# New 2-Aryl-9-methyl-*β*-carbolinium salts as Potential Acetylcholinesterase Inhibitor agents: Synthesis, Bioactivity and Structure–Activity Relationship

**DOI:** 10.1038/s41598-018-19999-3

**Published:** 2018-01-24

**Authors:** Bohang Zhou, Bingyu Zhang, Xingqiang Li, Xiuxiu Liu, Hui Li, Ding Li, Zhiming Cui, Huiling Geng, Le Zhou

**Affiliations:** College of Chemistry & Pharmacy, Northwest A&F University, Yangling, 712100 Shaanxi Province People’s Republic of China

## Abstract

A series of 2-aryl-9-methyl-*β*-carbolinium bromides (B) were synthesized and explored for anti-acetylcholinesterase (AChE) activities *in vitro*, action mechanism and structure-activity relationship. All the compounds B along with their respective 3,4-dihydro intermediates (A) presented anti-AChE activity at 10 μM. Thirteen compounds B showed the excellent activity with IC_50_ values of 0.11**–**0.76 μM and high selectivity toward AChE relative to butyrylcholinesterase (BChE), superior to galantamine (IC_50_ = 0.79 μM), a selective AChE inhibitor drug. Kinetic analysis showed that the action mechanisms of both compounds B and A are a competitive inhibition model. Structure-activity relationship analyses showed that the C = N^+^ moiety is a determinant for the activity. Substituents at 6, 7 or 4′ site, the indole-N-alkyl and the aromatization of the C-ring can significantly improve the activity. Molecular docking studies showed that the compounds could combine with the active site of AChE by the π-π or cation-π action between the carboline ring and the phenyl rings of the residues, and the β-carboline moiety is embedded in a cavity surrounded by four aromatic residues of Trp86, Tyr337, Trp439 and Tyr449. The present results strongly suggest that the para-position of the D-ring should be a preferred modification site for further structural optimization design. Thus, 2-aryl-9-methyl-*β*-carboliniums emerged as novel and promising tool compounds for the development of new AChE inhibitor agents.

## Introduction

As the most common form of adult onset dementia, Alzheimer’s disease (AD) is an age-related irreversible neurodegenerative disorder characterized by a progressive memory loss, a decline in language skills and other cognitive impairments^1^. It was estimated that 47 million people suffered from dementia worldwide in 2016, and this number is predicted to increase to more than 131 million by 2050. The total estimated worldwide cost of dementia is US$818 billion, and it will become a trillion dollar disease by 2030^2^. Therefore, given the aging of the population, AD is becoming one of the main public health issues we have to face.

AD is a complicated disease involving different molecular events. Its pathological features include tau-protein aggregation in nerve cells, *β*-amyloid protein aggregation in the spaces between nerve cells, oxidative stress and lowered levels of acetylcholine in the brain^3^. Among them, cholinergic hypothesis is the most popular explanation of mechanism of AD development^4^. Neuropathological evidence has proved that the memory impairment and behavioral abnormalities in patients with AD results from low level of acetylcholine (ACh) in different areas of the central nervous system, mainly the cerebral cortex and the hippocampus. Therefore, AChE inhibitors has been the leading strategy for the development of AD drugs, which can elevate the level of acetylcholine in the synapses between cholinergic neurons and enhance cholinergic function^5^. In addition, AChE inhibitors are also used for the treatment of senile dementia, ataxia, myasthenia gravis and Parkinson’s disease^6^, or as insecticides in modern agricultural procedures^7^.

Acetylcholinesterase and butyrylcholinesterase (BuChE) are two different types of cholinesterases present in the human brain, which catalyze the hydrolysis of choline-based esters to terminate cholinergic signal transmission. The two enzymes are distinguished on the basis of substrate specificities, tissue distribution and sensitivity to inhibitors^8,9^. AChE mainly exist in the synapses of the central and peripheral nervous systems and in the membranes of erythrocytes, while BuChE is primarily present in the blood and glial cells or neurons^10,11^. AChE hydrolyzes acetylcholine (ACh) more quickly whereas BuChE hydrolyzes butyrylcholine (BuCh) more quickly. Under normal conditions, acetylcholine (ACh) is dominantly decomposed by AChE instead of BuChE although both AChE and BuChE can hydrolyze ACh^12,13^. Therefore, AChE has been considered the main targeting cholinesterase in the AD scenario. Current clinical therapeutic strategy for mild-to-moderate AD is mainly to improve cholinergic neurotransmission by using AChE inhibitors^14^. However, it is worth mentioning that BuChE was also found to have a critical role for ACh hydrolysis in AD, especially in the late stage of AD^15,16^. In progressed AD, level of AChE in brain declines while BuChE increases, leading to ratio of BuChE/AChE shifting from 0.6 to 1.1^17,18^. At this point, BuChE can compensate for AChE loss by hydrolyzing ACh in cholinergic transmission. Therefore, to avoid the adverse effects caused by suppression of AChE, exploits of BuChE inhibitors for AD treatment have also aroused a worldwide popularity. A lot of effective and selective BuChE inhibitors have been discovered^19^. Furthermore, it was found that the two enzymes are also related with the formation of amyloid protein plaques, which is encouraging the development of dual- or multi-functional ChE inhibitors^20^.

At present, there are four FDA-approved AChE inhibitor-type drugs (tacrine, donepezil, galantamine and rivastigmine) for treatment of cognitive dysfunction and memory loss associated with mild-to-moderate AD^21^. Among them, donepezil and galantamine are reversible and selective AChE inhibitors that compete with acetylcholine for AChE binding^22^. Recent researches showed that AChE inhibitors not only alleviate the cognitive defect of AD patients by elevating acetylcholine (ACh) levels, but also act as disease modifying agents by preventing the early step of AD, the assembly of *β*-amyloid peptide (Aβ) into amyloid plaque^23,24^. Nevertheless, some obvious adverse effects including nausea and vomiting, decreased appetite, weight loss, hepatotoxicity or problems associated with bioavailability were also reported for these drugs^25–28^. Additionally, these drugs are only focused on the symptomatic aspects but cannot prevent, halt or reverse the progression of the disease^14^. Therefore, the search of central selective AChEIs devoid of adverse effects is still a challenging research topic. It is very necessary to find better AChE inhibitor agents and effective therapeutics for AD desease^29^.

To date, a lot of highly active AChE and/or BuChE inhibitors had been found, including synthetic, semi-synthetic and natural compounds^30^. It is worth noting that most of the compounds are nitrogen-containing compounds or alkaloids. Among them, *β*-carboline (pyrido[3,4-b]indole) compounds are one important type. *β*-Carboline alkaloids, also referred to as harman alkaloids, were widely distributed in organisms including plants, animals, halobios and human being^31^. In addition, *β*-carbolines occur in foods and cigarette smoke, suggesting human uptake and exposure to these compounds^32^. In the past decades, *β*-carbolines have attracted lots of attention from researchers due to their diverse biological activities such as antimicrobial^33^, antitumor^34^, antiviral^35^ and antiparasitic^36^, anti-inflammatory^37^, vasorelaxant^38^, antioxidant^39^, neuroactive or neurotoxic actions^40^. Especially interesting for us is that some natural or synthetic quaternary 2-methyl-*β*-carboline salts were also found to have strong AChE inhibitory activity^41,42^. The results suggest that *β*-carboline is a promising molecular framework for development of new anti-AChE agents.

**Figure 1:**
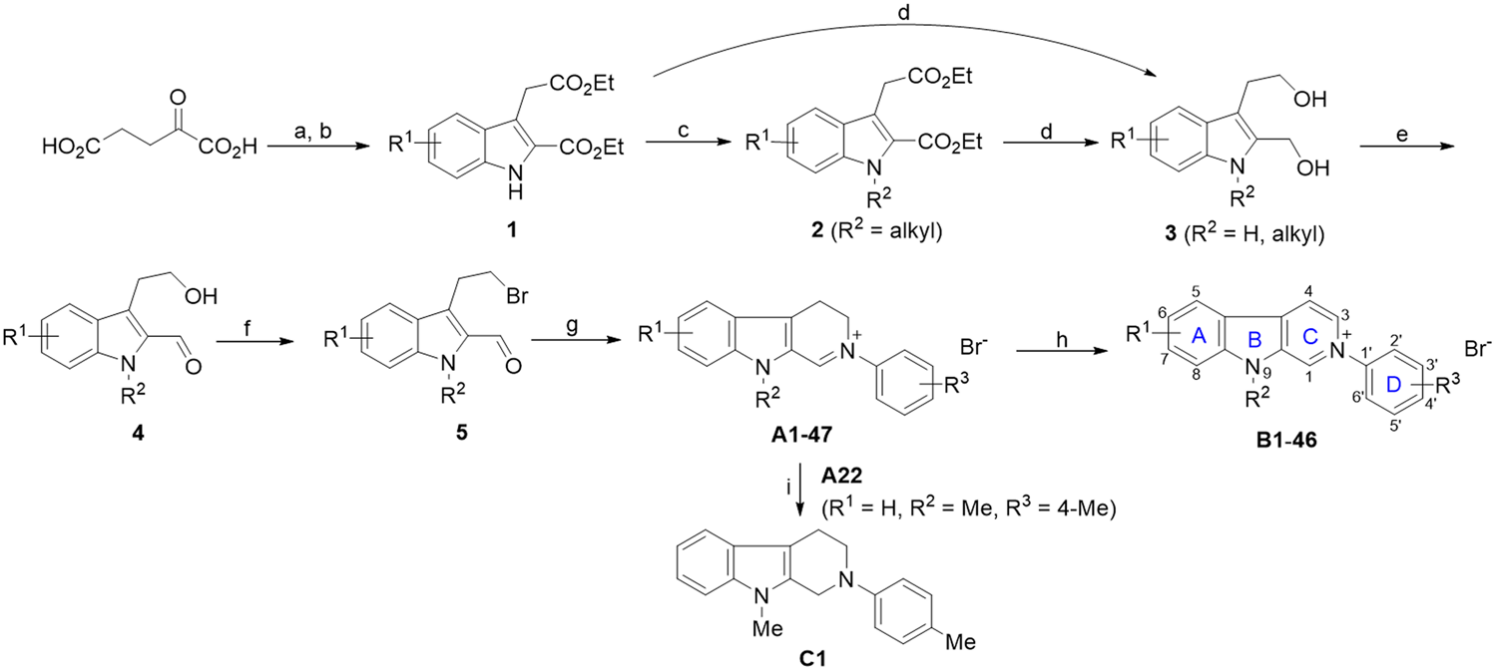
Synthetic route of target compounds B1**-**B34 and C1. Reagents and conditions: (**a**) phenylhydrazine-HCl, H_2_O, 12 h at r.t.; (**b**) EtOH, con. H_2_SO_4_, reflux for 12 h; (**c**) R^2^I or R^2^Br, NaH, dry DMF, 0 °C; (**d**) LiAlH_4_, dry THF, 0 °C to r.t.; (**e**) active MnO_2_, CHCl_3_, r.t.; (**f**) MsCl, Et_3_N, LiBr, dry THF, 0 °C to r.t.; (**g**) R-PhNH_2_, TsOH·H_2_O, EtOH, r.t.; (**h**) Pd/C, acetonitrile, reflux; (**i**) NaBH_4_, EtOH, r.t.

With the aim of finding more potent *β*-carboline-type AChE inhibitors for treatment of AD, herein, a series of new *β*-carboline derivatives (Fig. 1) were designed and explored for AChE inhibition activity, action mechanism as well as structure-activity relationship (SAR). These compounds are characterized by a quaternary 2-phenyl-9-methyl-*β*-carbolinium skeleton with various substituents on the 2-aryl ring.

## Results and Discussion

### Chemistry

Based on the consideration of SAR and structural diversity, we designed a series of 2-aryl-9-mehtyl-*β*-carboline salts (**B1**-**B33**) with various substituents on the D-ring (Fig. 1) in order to get an insight into the effect of the substitution pattern of the D ring on anti-AChE activity. The substituents include both electron-withdrawing groups like halogen atom, CN, CF_3_, NO_2_, and electron-donating groups like CH_3_, OCH_3_ and OH. The substitution position involves the 2′, 3′ or 4′ site of the D-ring. In addition, we also designed one the indole-N-H instead of N-Me derivative (**B34**) and one 1,2,3,4-tetrahydro derivative (**C1**) of compounds **B** in order to explore the effect of the indole-N-substituents and the C-ring aromatization on the activity. The substitution patterns of target compounds are depicted in Table 1.

**Table 1:** Structures and inhibitory activity of compounds A and intermediates B against AChE. ^*a*^The test concentration of the compound is 10 μM. The differences between data with the different lowercases within a column are significant (*p* < 0.05).

Compound				Inhibition rate (%)^*a*^	Compound				Inhibition rate (%)^*a*^
No.	R^1^	R^2^	R^3^		No.	R^1^	R^2^	R^3^	
**B1**	H	Me	H	71.5 ± 0.3 l	**A1**	H	Me	H	**40.9** ± **2.1 ij**
**B2**	H	Me	2′-F	59.8 ± 3.2 n	**A2**	H	Me	2′-F	15.1 ± 2.8 mnop
**B3**	H	Me	3′-F	70.5 ± 2.2 l	**A3**	H	Me	3′-F	22.4 ± 1.1 l
**B4**	H	Me	4′-F	84.3 ± 1.5 hi	**A4**	H	Me	4′-F	**49.3** ± **2.2 fgh**
**B5**	H	Me	2′-Cl	38.5 ± 1.9 st	**A5**	H	Me	2′-Cl	2.4 ± 8.0 stu
**B6**	H	Me	3′-Cl	56.4 ± 2.5 no	**A6**	H	Me	3′-Cl	19.3 ± 4.0 lm
**B7**	H	Me	4′-Cl	91.2 ± 1.5 cde	**A7**	H	Me	4′-Cl	**60.1** **±** **2.9 d**
**B8**	H	Me	2′-Br	32.1 ± 0.9 u	**A8**	H	Me	2′-Br	5.9 ± 1.0 qrs
**B9**	H	Me	3′-Br	48.8 ± 6.6 q	**A9**	H	Me	3′-Br	16.2 ± 3.3 mno
**B10**	H	Me	4′-Br	95.4 ± 0.8 ab	**A10**	H	Me	4′-Br	**60.3** ± **1.6 d**
**B11**	H	Me	2′-I	43.3 ± 1.8 r	**A11**	H	Me	2′-I	−10.6 ± 3.0 w
**B12**	H	Me	3′-I	56.4 ± 5.9 no	**A12**	H	Me	3′-I	10.1 ± 2.8 pqr
**B13**	H	Me	4′-I	93.8 ± 0.4 abcd	**A13**	H	Me	4′-I	**54.2** ± **3.2 ef**
**B14**	H	Me	2′-OH	23.9 ± 4.3 v	**A14**	H	Me	2′-OH	23.2 ± 2.5 kl
**B15**	H	Me	3′-OH	72.1 ± 1.8 l	**A15**	H	Me	3′-OH	**51.8** ± **9.1 fg**
**B16**	H	Me	4′-OH	82.1 ± 2.5 ij	**A16**	H	Me	4′-OH	**64.3** ± **0.3 cd**
**B17**	H	Me	2′-OMe	41.0 ± 2.9 rs	**A17**	H	Me	2′-OMe	5.8 ± 2.4 qrs
**B18**	H	Me	3′-OMe	53.5 ± 0.4 op	**A18**	H	Me	3′-OMe	4.7 ± 1.5 rst
**B19**	H	Me	4′-OMe	90.7 ± 0.8 def	**A19**	H	Me	4′-OMe	66.1 ± 0.5 c
**B20**	H	Me	2′-Me	59.6 ± 3.2 n	**A20**	H	Me	2′-Me	44.3 ± 1.7 j
**B21**	H	Me	3′-Me	72.4 ± 3.9 l	**A21**	H	Me	3′-Me	28.6 ± 1.7 k
**B22**	H	Me	4′-Me	95.2 ± 0.5 abc	**A22**	H	Me	4′-Me	**77.6** ± **1.5 a**
**B23**	H	Me	2′-CN	68.7 ± 0.7 lm	**A23**	H	Me	2′-CN	10.1 ± 1.6 pqr
**B24**	H	Me	3′-CN	50.7 ± 2.2 pq	**A24**	H	Me	3′-CN	18.9 ± 0.3 lmn
**B25**	H	Me	4′-CN	36.8 ± 0.8 t	**A25**	H	Me	4′-CN	15.6 ± 1.8 mnop
**B26**	H	Me	3′-CF_3_	41.0 ± 1.9 rs	**A26**	H	Me	3′-CF_3_	12.8 ± 2.1 kln
**B27**	H	Me	4′-CF_3_	91.4 ± 0.3 bcde	**A27**	H	Me	4′-CF_3_	28.4 ± 1.0 k
**B28**	H	Me	3′-NO_2_	34.9 ± 1.8 tu	**A28**	H	Me	3′-NO_2_	14.5 ± 3.9 mnop
**B29**	H	Me	2′,6′-diF	48.5 ± 3.1 pq	**A29**	H	Me	2′,6′-diF	−3.3 ± 2.0 uv
**B30**	H	Me	2′,4′-diCl	90.6 ± 1.0 def	**A30**	H	Me	2′,4′-diCl	−0.5 ± 2.4 tu
**B31**	H	Me	3′,5′-diCl	18.9 ± 2.5 v	**A31**	H	Me	3′,5′-diCl	0.7 ± 0.5 stu
**B32**	H	Me	2′-F-4′-Br	92.8 ± 1.0 abc	**A32**	H	Me	2′-F-4′-Br	11.1 ± 2.8 opq
**B33**	H	Me	2′,4′-diBr	96.7 ± 0.05 a	**A33**	H	Me	2′,4′-diBr	−7.0 ± 8.3 vw
**B34**	6-OMe	Me	H	85.7 ± 2.5 ghi	**A34**	6-OMe	Me	H	68.4 ± 2.8 c
**B35**	6-Me	Me	H	88.6 ± 2.5 efg	**A35**	6-Me	Me	H	70.3 ± 2.2 bc
**B36**	7-F	Me	H	76.2 ± 1.1 k	**A36**	7-F	Me	H	59.3 ± 2.1 de
**B37**	7-Cl	Me	H	89.9 ± 0.7 def	**A37**	7-Cl	Me	H	66.6 ± 4.7 c
**B38**	H	Et	H	87.8 ± 1.1 efgh	**A38**	H	Et	H	76.6 ± 3.7 a
**B39**	H	Pr	H	78.8 ± 0.9 jk	**A39**	H	Pr	H	67.1 ± 2.4 c
**B40**	H	iPr	H	86.9 ± 0.8 fgh	**A40**	H	iPr	H	68.9 ± 5.1 bc
**B41**	H	allyl	H	78.2 ± 1.6 k	**A41**	H	allyl	H	74.4 ± 1.1 ab
**B42**	H	Bu	H	65.7 ± 0.6 m	**A42**	H	Bu	H	53.9 ± 1.5 ef
**B43**	H	iBu	H	84.1 ± 0.6 hi	**A43**	H	iBu	H	46.5 ± 1.9 ghi
**B44**	H	Bn	H	47.8 ± 2.0 q	**A44**	H	Bn	H	31.2 ± 4.4 ef
**B45**	H	CH_2_CO_2_Et	H	42.0 ± 0.7 rs	**A45**	H	CH_2_CO_2_Et	H	38.8 ± 4.6 j
**B46**	H	H	H	43.0 ± 1.8 r	**A46**	H	H	H	41.3 ± 1.4 ij
**C1**	H	Me	4′-Me	−2.3 ± 3.2 w	**A47**	H	H	4′-Br	16.0 ± 1.7 mnop
Galantamine			91.2 ± 1.1 cde						

The synthetic route of target compounds is shown in Fig. 1, in which phenylhydrazine–HCl and α-ketoglutaric acid were used as the starting materials. According to our reported method^33^, key intermediates **A1**-**A35** were synthesized *via* 6–7 steps. In sequence, the intermediates **A1**-**A34** were dehydrogenated by Pd/C in acetonitrile or toluene to provide the final compounds **B1-B34**. Compound **A1** was reduced with NaBH_4_ to obtain its 1,2,3,4-tetrahydro derivative **C1**.

Compounds **B** include 32 new compounds (**B2-B33**) and 2 known compounds (**B1**, **B34**). The known compounds **B1**, **B34** and intermediates **A1-A35** were confirmed by comparison of NMR and MS data and those reported in literature^33^. New compounds **B2-B33** were elucidated by ^1^H NMR, ^13^C NMR and HRMS analyses. All compounds **B** showed some similar spectroscopic characteristics because of the structural similarity. Each compound showed a characteristic ion peak at *m*/*z* [M-Br]^+^ in positive ESI-HRMS spectra. The presence of bromide anion was confirmed by ion peaks at *m*/*z* 79 and 81 in negative ESI-MS spectra. In ^1^H and ^13^C NMR spectra, each compound **B** revealed one signal of H-1 at *δ*_H_ ca. 10.0 (1 H, s or d, *J* = ca 1.2 Hz) and one signal of C-1 in the range of *δ*_C_ 152**–**163, one doublet signal of H-4 at *δ*_H_ ca. 9.02 (1 H, d, *J* = ca 6.5 Hz), one doublet or double doublet signal of H-3 at *δ*_H_ ca. 9.00 (1 H, d, *J* = ca. 6.5 Hz, or dd, J = ca. 6.5, ca. 1.2 Hz), one signal of C-4 at *δ*_C_ ca. 130 and one signal of C-3 at *δ*_C_ ca.133. The NMR spectra of **C1** were similar to that of **B22** except for three additional CH_2_ signals at *δ*_H_ 4.38 (2 H, s, H-1)/*δ*_C_ 46.6 (C-1), 3.60 (2 H, t, *J* = 5.6 Hz, H-3)/*δ*_C_ 48.7 (C-3), 2.93 (2 H, t, *J* = 5.6 Hz, H-4)/*δ*_C_ 21.8 (C-4) and fewer signals of one HC=N^+^ unit and one AX system [C(3)H=C(4)H].

### AChE inhibition activity

According to Ellman’s coupled enzyme assay^43^, compounds **B1-B34**, **C1** along with intermediates **A1-A35** were screened for inhibition activity on AChE at 10 μM. Galantamine, a selective and competitive AChE inhibitor drug for treatment of AD, was used as a reference control. The results are shown in Table 1.

The data in Table 1 showed that all compounds **B** and the majority of intermediates **A** presented some anti-AChE activity at 10 μM. However, compared with compounds **A**, except for **B14**, all compounds **B** showed the much higher activity. Among compounds **B**, thirty-two compounds displayed inhibition rates of >50%, and nine compounds (**B7**, **B10**, **B13**, **B19**, **B22**, **B27**, **B30**, **B32** and **B33**) showed the inhibition rates of 90.6% to 96.7%, higher or equal to galantamine (91.2%). **B33** (R = 2′,4′-diBr, R′ = Me) showed the highest activity. In contrast to compounds **B**, only the minority of compounds **A** (**A1**, **A4**, **A7**, **A10**, **A13**, **A15**, **A16**, **A19**, **A22**, **A34-A43** and **A46**) showed the moderate inhibition rates of 40% to 78%.

In order to explore anti-AChE potential more detail, the compounds with the higher initial activity in Table 1 were further determined for median inhibition concentrations (IC_50_) on AChE. Galantamine was used as a reference standard. The results are listed in Table 2. Gratifyingly, thirteen compounds **B** (**B7, B10, B13, B19, B22, B27, B30, B32**, **B33**, **B35**, **B37**, **B38** and **B40**) showed the excellent activity with IC_50_ values of ≤0.76 μM, higher or approximately equal to that of galantamine (IC_50_ = 0.79 μM), agreement with that showed in Table 1. Among them, **B33** (IC_50_ = 0.11 μM) was most active, superior to rivastigmine (IC_50_ = 9.94 μM)^44^ and tacrine (IC_50_ = 0.25 μM)^45^ but inferior to donepezil (IC_50_ = 0.03 μM)^46^. In contrast to compounds **B**, all the compounds **A** in Table 2 showed the low to the moderate activity (IC_50_ values > 1.4 μM).

**Table 2:** Median inhibition concentrations of part of compounds B and intermediates A against AChE and BuChE. ^*a*^SI: selectivity index, the ratio value of IC_50_ (BuChE)/IC_50_ (AChE); ^*b*^Estimated values based on the results in Table 1; ^*c*^ND: no determination. ^*d*^NA: no inhibition activity at 10 μM; ^*e*^The data are cited from ref.^44^; ^*f*^The data are cited from ref.^45^. ^*g*^The data are cited from ref.^46^.

Compound	IC_50_ ± S.D (μM)		SI^*a*^	Compound	IC_50_ ± S.D (μM)		SI^*a*^
	AChE	BuChE			AChE	BuChE	
**B1**	3.18 ± 0.12	124.9 ± 7.8	39.3	**A1**	18.6 ± 2.93	10.1 ± 1.66	0.54
**B2**	6.65 ± 1.07	23.0 ± 0.53	3.49	**A2**	>10^*b*^	ND	
**B3**	4.73 ± 0.33	72.4 ± 4.25	15.3	**A3**	>10^*b*^	ND	
**B4**	1.46 ± 0.12	51.6 ± 2.43	35.3	**A4**	14.1 ± 1.41	59.5 ± 2.72	4.22
**B5**	>10^*b*^	ND^*c*^		**A5**	≫10^*b*^	ND	
**B6**	9.65 ± 0.78	33.1 ± 1.07	3.40	**A6**	>10^*b*^	ND	
**B7**	**0.56** ± 0.07	60.0 ± 1.52	107.1	**A7**	7.58 ± 1.29	42.2 ± 0.86	5.57
**B8**	>10^*b*^	ND		**A8**	≫10^*b*^	ND	
**B9**	≈10^*b*^	ND		**A9**	>10^*b*^	ND	
**B10**	**0.43** ± 0.04	55.4 ± 1.73	128.8	**A10**	6.79 ± 2.29	76.2 ± 11.0	11.2
**B11**	>10^*b*^	ND		**A11**	NA	ND	
**B12**	11.1 ± 1.34	21.1 ± 0.46	1.90	**A12**	>10^*b*^	ND	
**B13**	**0.24** ± 0.03	40.4 ± 1.83	168.3	**A13**	5.50 ± 0.37	53.8 ± 2.51	9.80
**B14**	>10^*b*^	ND		**A14**	>10^*b*^	ND	
**B15**	3.92 ± 0.30	29.6 ± 2.48	7.60	**A15**	6.12 ± 0.61	10.0 ± 0.59	1.63
**B16**	1.50 ± 0.19	67.9 ± 11.5	45.3	**A16**	2.81 ± 0.22	28.0 ± 2.88	9.96
**B17**	>10^*b*^	ND		**A17**	≫10^*b*^	ND	
**B18**	9.69 ± 0.80	62.2 ± 3.62	6.42	**A18**	≫10^*b*^	ND	
**B19**	**0.47** ± 0.04	34.0 ± 2.00	72.3	**A19**	2.92 ± 0.28	137.0 ± 0.79	46.9
**B20**	9.71 ± 0.55	154.0 ± 9.1	15.9	**A20**	>10^*b*^	ND	
**B21**	5.05 ± 0.26	68.6 ± 8.06	13.6	**A21**	>10^*b*^	ND	
**B22**	**0.29** ± 0.03	32.2 ± 1.65	111.6	**A22**	1.45 ± 0.21	80.1 ± 5.27	55.2
**B23**	4.41 ± 0.29	195.6 ± 13.4	44.4	**A23**	>10^*b*^	ND	
**B24**	10.3 ± 0.60	212.2 ± 17.4	20.6	**A24**	>10^*b*^	ND	
**B25**	>10^*b*^	ND		**A25**	>10^*b*^	ND	
**B26**	>10^*b*^	ND		**A26**	>10^*b*^	ND	
**B27**	**0.70** ± 0.11	77.9 ± 4.97	111.3	**A27**	>10^*b*^	ND	
**B28**	>10^*b*^	ND		**A28**	>10^*b*^	ND	
**B29**	≈10^*b*^	ND		**A29**	NA	ND	
**B30**	**0.55** ± 0.07	132.6 ± 26.3	241.1	**A30**	NA	ND	
**B31**	>10^*b*^	ND		**A31**	NA	ND	
**B32**	**0.30** ± 0.08	115.5 ± 18.2	385.0	**A32**	>10^*b*^	ND	
**B33**	**0.11** ± 0.07	30.6 ± 2.31	278.2	**A33**	NA	ND	
**B34**	0.94 ± 0.23	33.3 ± 2.34	35.4	**A34**	2.04 ± 0.33	37.6 ± 2.84	18.4
**B35**	**0.42** ± 0.15	17.2 ± 1.86	41.0	**A35**	2.66 ± 0.37	10.6 ± 0.58	3.98
**B36**	2.61 ± 0.51	27.0 ± 1.14	10.3	**A36**	4.74 ± 0.90	14.2 ± 0.44	3.00
**B37**	**0.57** ± 0.08	14.4 ± 0.84	25.3	**A37**	4.02 ± 0.95	11.7 ± 0.50	2.91
**B38**	**0.76** ± 0.21	17.1 ± 0.78	22.5	**A38**	3.69 ± 0.40	10.4 ± 0.40	2.82
**B39**	3.08 ± 0.66	37.0 ± 5.40	12.0	**A39**	5.72 ± 0.66	13.0 ± 0.62	2.27
**B40**	**0.54** ± 0.16	16.5 ± 2.37	30.6	**A40**	3.73 ± 0.34	16.3 ± 0.56	4.37
**B41**	1.18 ± 0.30	16.2 ± 1.80	13.7	**A41**	3.91 ± 0.36	6.83 ± 0.20	1.75
**B42**	2.91 ± 0.33	22.8 ± 3.18	7.84	**A42**	9.79 ± 3.55	10.0 ± 0.68	1.02
**B43**	0.98 ± 0.03	6.53 ± 0.61	6.66	**A43**	>10^*b*^	ND	
**B44**	11.2 ± 2.54	24.1 ± 2.23	2.15	**A44**	>10^*b*^	ND	
**B45**	>10^*b*^	ND		**A45**	>10^*b*^	ND	
**B46**	18.0 ± 2.42	18.7 ± 2.80	1.04	**A46**	18.2 ± 1.97	3.90 ± 0.53	0.21
**C1**	NA^*d*^	ND		**A47**	>10^*b*^	ND	
Galantamine	0.79 ± 0.05	13.7 ± 0.71	18.0	Tacrine	0.25 ± 0.01^*e*^	0.05 ± 0.00^*e*^	0.22 ^*e*^
Rivastigmine	9.94 ± 0.83^*f*^	2.86 ± 0.22^*f*^	0.29^*f*^	Donepezil	0.03 ± 0.01^*g*^	5.40 ± 0.27^*g*^	180^*g*^

There is increasing recognition that BuChE may play an important role in cholinergic transmission^15,16^. While the level of AChE decreases dramatically in the advanced stage of AD, BuChE increases in brain regions involved in cognitive functions^17,18^. At this point, BuChE is able to compensate for AChE loss by hydrolyzing ACh. These observations lead one to believe that inhibition of both AChE and BuChE may contribute to improvement of current symptomatic treatments of AD. Therefore, the compounds in Table 2 were also evaluated for BuChE inhibition activity by means of Ellman’s method^43^. Galantamine was used as a reference standard. The results in Table 2 showed that the compounds also exhibited some inhibition activity on BuChE with IC_50_ values of 3.90 to 212.2 μM, but compared with the anti-AChE activity of each the compound, its anti-BuChE activity is obviously lower except for **A1** and **A34**. The results above showed that both the compounds **B** and **A** had the higher selectivity for AChE than BuChE. Compared with galantamine, thirteen compounds **B** (**B7, B10, B13, B19, B22, B27, B30, B32, B33, B34**, **B35**, **B38** and **B40**) also showed the higher selectivity for AChE in addition to the higher anti-AChE activity. Among them, the selectivity indexes (SI) of compounds **B30, B32** and **B33** reached up to >240, comparable with that of donepezil (SI = 180)^46^, a selective AChE inhibitor drug. Relative to compounds **B**, compounds **A** in Table 2 showed the lower selectivity to AChE. Unexpectedly, compounds **A1** and **A46** (SI < 1.0) showed the higher selectivity to BuChE but not AChE.

As the representative compounds, compound **B33** with the highest anti-AChE activity among compounds **B** and compound **A22** with the highest anti-AChE activity among compounds **A** were further evaluated for cytotoxic activities on both mouse neuroblastoma N2a cells and primary cultured porcine fetal kidney cells. Meanwhile, in order to compare the cytotoxicity of compounds A and B, compound **B22** was subjected to the assay. The results in Table 3 showed that both **B33** and **B22** showed the much lower cytotoxicity with IC_50_ values of 10.6 and 19.2 μM on mouse neuroblastoma N2a cells compared with their respective anti-AChE activity (IC_50_ = 0.11, 0.29 μM). Similarly, both **B33** and **B22** also showed the much lower cytotoxicity on primary cultured porcine fetal kidney cells (IC_50_ = >20, 18.1 μM). Compared with **B22**, compound **A22** showed the higher cytotoxicity with IC_50_ values of 2.13 and 5.18 μM on the two strains of cells although its cytotoxicities are also lower than its anti-AChE activity (IC_50_ = 1.45 μM).

**Table 3:** Cytotoxicity of the compounds on mouse neuroblastoma N2a cells and primary cultured porcine fetal kidney cells (48 h). ^*a*^95% CI: the confidence interval of IC_50_ at 95% probability.

Compound	Mouse neuroblastoma N2a cells		Primary cultured porcine fetal kidney cells	
	IC_50_ (μM)	95% CI (μM)	IC_50_ (μM)	95% CI (μM)
**B33**	10.6	8.26–14.4	>20	
**B22**	19.2	13.0–33.2	18.1	13.5–27.2
**A22**	2.13	1.88–2.41	5.18	4.72–5.68

### Mechanism of AChE inhibition

**Figure 2:**
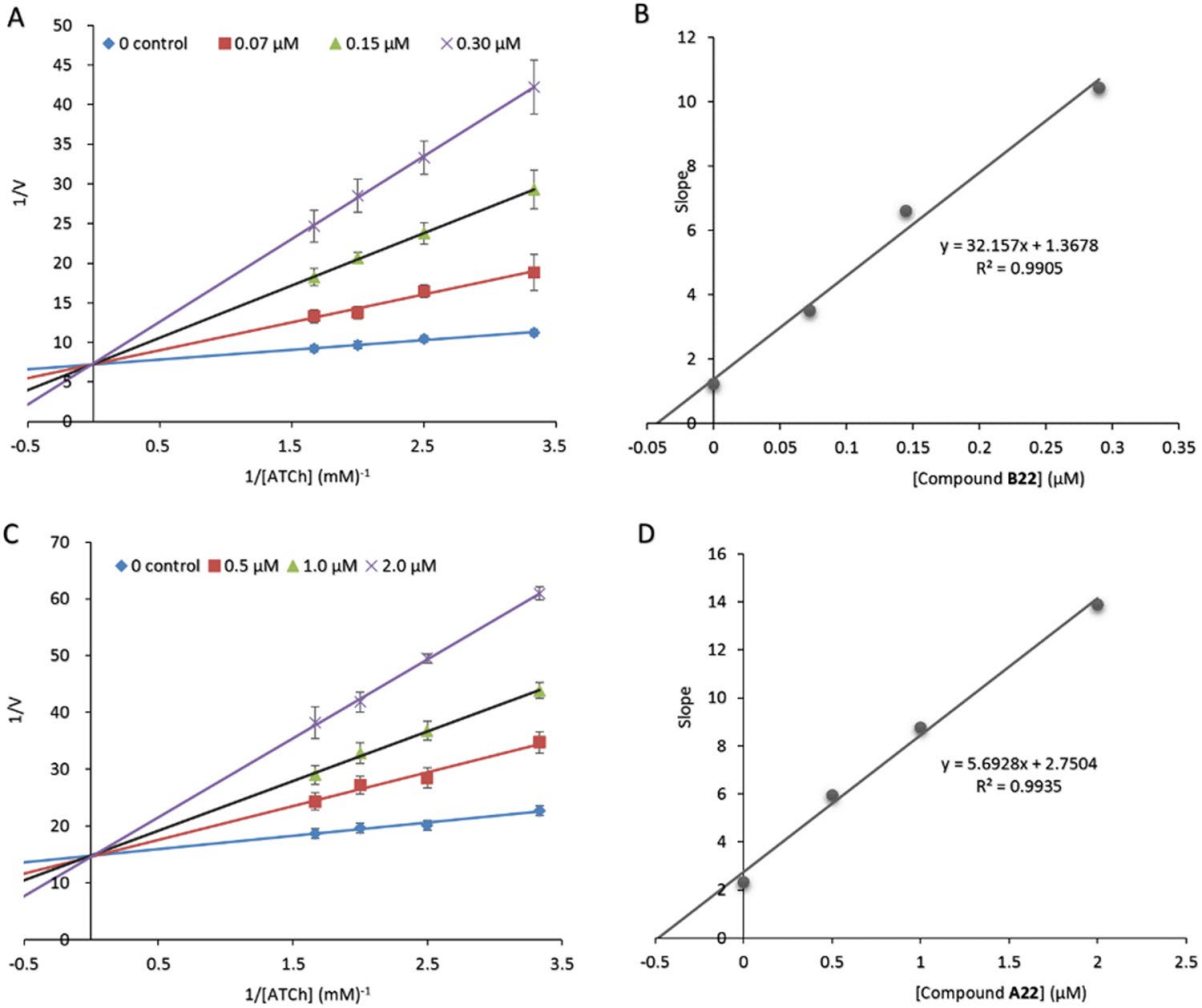
The Mechanism of AChE inhibition by compound B22 (**A**) and intermediate A22 (**C**) respective to ATCh, and their *K*_i_ determination (**B,D**). (**A** and **B**) The reciprocals of the initial reaction rates and substrate concentrations are plotted. (**C** and **D**) The slope values of the lines from graph **A** or **C** are plotted versus the inhibitor concentration, affording an equation of linear regression. When *y* is 0, the equations give *K*_i_ values of 4.25 × 10^−8^ M for compound B22 and 4.83 × 10^−7^ M for compound A22.

In order to know the mechanism of AChE inhibition, compounds **B22** and **A22** as two representative compounds were conducted for kinetic analysis of AChE inhibition. The graphical analysis of the inhibition data for **B22** and **A22** in comparison with galantamine is shown in Fig. 2. The results in Fig. 2A and C clearly showed that both **B22** and **A22** inhibited AChE in a competitive manner with the substrate acetylthiocholine iodide (ATCh). The estimates of the inhibition constants *K*_i_ of **B22** and **A22** for AChE were 4.25 × 10^−8^ M and 4.83 × 10^−7^ M, respectively, while the *K*_m_ value of acetylthiocholine iodide (ATCh) for AChE was 1.63 × 10^−4^ M. The results showed that the binding capacity of **B22** with AChE is approximately 11-fold that of **A22** and 3835-fold that of the substrate ATCh.

### Structure-activity relationships

By comparison of the activity and structures of the compounds in Tables 1 and 2, we can find some clear and regular structure-activity relationships for compounds **B** and **A** (Tables 4, 5 and 6).

**Table 4:** Effect of substitution patterns of the D-ring on the activity of compounds **B** and **A**. ^*a*^Arrows outside of parentheses represent compounds **B**; arrows inside parentheses represent compounds **A**. ^*b*^↑significantly increasing the activity relative to R = H; ↓significantly decreasing the activity; ±, slight change of the activity. ^*c*^“nd” means no determination.

Substituent (R)	Anti-AChE			Selectivity toward AChE			Anti-BChE		
	2′-R	3′-R	4′-R	2′-R	3′-R	4′-R	2′-R	3′-R	4′-R
F	↓ (↓)	± (↓)	↑ (↑)	↓ (nd)	↓ (nd)	↓ (↑)	↑ (nd)	↑ (nd)	↑ (↓)
Cl	↓ (↓)	↓ (↓)	↑ (↑)	nd	↓ (nd)	↑ (↑)	nd	↑ (nd)	↑ (↓)
Br	↓ (↓)	↓ (↓)	↑ (↑)	nd	nd	↑ (↑)	nd	nd	↑ (↓)
I	↓ (↓)	↓ (↓)	↑ (↑)	nd	↓ (nd)	↑ (↑)	nd	↑ (nd)	↑ (↓)
OH	↓ (↓)	± (↑)	↑ (↑)	nd	↓ (↑)	↑ (↑)	nd	↑ (±)	↑ (↓)
OMe	↓ (↓)	↓ (↓)	↑ (↑)	nd	↓ (nd)	↑ (↑)	nd	↑ (nd)	↑ (↓)
Me	↓ (±)	± (↓)	↑ (↑)	↓(nd)	↓ (nd)	↑ (↑)	↓ (nd)	↑ (nd)	↑ (↓)
CN	± (↓)	↓ (↓)	↓ (↓)	↑ (nd)	↓ (nd)	nd	↓ (nd)	↓ (nd)	nd
CF_3_	nd	↓ (↓)	↑ (↓)	Nd	nd	↑(nd)	nd	nd	↑ (nd)
NO_2_	nd	↓ (↓)	nd	Nd	nd	nd	nd	nd	nd

**Table 5:** Effect of substitution patterns of the D-ring on the activity of compounds B and A. ^*a*^↑significantly increasing the activity relative to R = H; ↓significantly decreasing the activity; ±, slight change of the activity. ^*b*^“nd” means no determination.

Dihalogenation	Anti-AChE		Selectivity toward AChE		Anti-BChE	
	Compound B	Compound A	Compound B	Compound A	Compound B	Compound A
2′,4′-diCl	↑	↓	↑	nd	↓	nd
2′,4′-diBr	↑	↓	↑	nd	↑	nd
2′-F-4′-Br	↑	↓	↑	nd	↑	nd
2′,6′-diF	↓	↓	nd	nd	nd	nd
3′,5′-diCl	↓	↓	nd	nd	nd	nd

**Table 6:** Effect of the indole-N-alkyl and substitution patterns of the A-ring on the activity of compounds B and A. ^*a*^↑, significantly increasing the activity relative to R = H; ↓, significantly decreasing the activity; ±, slight change of the activity. ^*b*^“nd” means no determination.

Substituent	Anti-AChE		Selectivity toward AChE		Anti-BChE	
	Compound B	Compound A	Compound B	Compound A	Compound B	Compound A
6-OMe	↑	↓	↓	↑	↑	↓
6-Me	↑	↓	±	↑	↑	±
7-F	↑	↓	↓	↑	↑	↓
7-Cl	↑	↓	↓	↑	↑	±
(N)Me	↑	±	↑	↑	↓	↓
(N)Et	↑	↑	↑	↑	±	↓
(N)Pr	↑	↑	↑	↑	↓	↓
(N)iPr	↑	↑	↑	↑	±	↓
(N)Allyl	↑	↑	↑	↑	±	↓
(N)Bu	↑	↑	↑	↑	↓	↓
(N)iBu	↑	±	↑	nd	↑	nd
(N)Bn	↑	↓	↑	nd	↓	nd
(N)CH_2_CO_2_Et	±	↓	nd		nd	nd

Firstly, the substitution pattern of the D-ring can dramatically influence the activity. For compounds **B**, the *p*-substitution of all the substituents except cyano group (**B25**, **A25**) can significantly increase the anti-AChE activity (**B4**, **B7**, **B10**, **B13**, **B16**, **B19**, **B22**, **B27** *vs* **B1**) whereas the *o*- or *m*-substitution generally leads to decrease or slight change of the activity. A similar trend was also found in compounds **A** (**A4**, **A7**, **A10**, **A13**, **A16**, **A19**, **A22** *vs* **A1**). The substituents above not only include electron-donating groups like OH, OMe or Me and electron-withdrawing groups like halogen atoms or CF_3_ but also involve hydrogen-bond acceptors (OMe) and hydrogen-bond donors (OH). Therefore, we speculated that the effect of the *p*-substituents on the activity should be mainly steric effect but not electron effect or hydrogen-bond effect. For *p*-mono-substituted compounds **B**, the order of the anti-AChE activity is the iodide (**B13**) ≈ the Me-substituted compound (**B22**) > the bromide (**B10**) ≈ the MeO-substituted compound (**B19**) > the chloride (**B7**) > the CF_3_-substituted compound (**B27**) > the fluoride (**B4**) ≈ the OH-substituted compound (**B16**). The results above strongly suggest that the 4′ site of the D-ring should be one important modifiable position for further structure optimization.

For BuChE, the effect of substituents on the D-ring on the activity strongly depends on the structure of the C-ring. For compounds **B** with the aromatic C-ring, the introduction of substituents to the D-ring causes increase of the anti-BuChE activity in most cases. However, the opposite was observed for compounds **A** with a nonaromatic C-ring. Nevertheless, 4′-substituents (Cl, Br, I, Me, OMe, OH, CF_3_) can significantly increase the selectivity of both compounds **B** and **A** to AChE at the same time.

For the dihalides (**B29**‒**33**), the presence of 2′,4′-dihalogen atoms (**B30**, **32**, **33**) can significantly improve both the anti-AChE activity and selectivity, whereas the presence of 2′,6′-difluoro or 3′,5′-dichloro leads to dramatic decrease of the activity (**B29**, **B31** *vs* **B1**). Compared the corresponding 4′-mono-halogenated compounds, 2′,4′-dihalogenated compounds showed the slight improvement of the anti-AChE activity although they exhibited the much higher selectivity for AChE (**B30** *vs* **B7**; **B32** or **B33** *vs* **B10**). The results above show that for the 2′,4′-halogenated compounds, 2′-halogen atom can significantly increase the selectivity to AChE but not greatly influences the anti-AChE activity (**B30** *vs* **B7**; **B32** or **B33** *vs* **B10**). In other words, there may be a synergistic effect on the anti-AChE selectivity between 2′-Cl and 4′-Cl, 2′-F and 4′-Br or 2′-Br and 4′-Br. The selectivity indexes for AChE of the 2′,4′-dibromo compound (**B33**), the 2′-fluoro-4′-bromo compound (**B32**), 2′,4′-dichloro compound (**B30**) reach up to 278, 385 and 241, respectively.

As strong electron-withdrawing substituents, cyano, nitro or trifluoromethyl group on the D-ring generally causes decrease of the activity of compounds **B** (**B24-26**, **B28** *vs* **B1**). The exception is that 4′-CF_3_ is able to dramatically improve the activity (**B27** *vs* **B1**). Unlike compounds **B**, the above substituents or double halogen atoms on the D-ring always leads to decrease of the activity of compounds **A** (**A22**-**33** *vs* **A1**).

Secondly, the C=N^+^ moiety of the compounds should be a determinant for the activity because transformation of the 3,4-dihydro-β-cabolinium (**A22**) or aromatic β-cabolinium (**B22**) to its 1,2,3,4-tetrahydro-derivative **C1** leads to complete loss of the activity. A similar case was also observed in some tertiary aromatic β-carbolines and their 1,2,3,4-tetrahydro-β-carbolines, where only quaternary aromatic 2-methyl-β-carboliniums showed the higher activity, whereas its corresponding tertiary aromatic β-carboline derivatives without 2-methyl, 2-methyl-1,2,3,4-tetrahydro-β-carbolines or quaternary 2,2-dimethyl-1,2,3,4-tetrahydro-β-carboline salts only exhibited very weak or no activity^41,42^.

Thirdly, compared with **B46** (the indole-N-H), the indole-N-alkylation can significantly improve the anti-AChE activity of compounds **B**. This effect varies with the type and steric hindrance of the alkyl groups. A similar case was also observed for compounds **A**. Among all the substituents, iso-propyl (**B40**) and ethyl (**B38**) can give the best improvement of the anti-AChE activity followed by iso-butyl (**B43**) and allyl (**B41**) while benzyl has the weakest effect on the activity. Methyl, propyl or butyl cause the moderate effect. The only exception is that —CH_2_CO_2_Et has almost no effect on the activity (**B45** *vs* **B46**, Table 1). On the contrary, for anti-BuChE activity, whether for compounds **B** or **A**, the indole-N-alkylation always causes decrease of the activity compared with **B46** or **A46** (the indole-N-H). The above SARs are obviously different from that of 2-methyl-*β*-carbolinium salts, where the indole-N-methylation is not able to significantly increase the anti-AChE activity^41^, even decrease the activity^42^. The results above suggest that the effect of the indole-N-alkylation on the activity of the aromatic 2-substituted-*β*-carboliniums is also related with the type of the pyridine-N-substituents.

It is worth noting that for compounds **A**, the effect of the indole-N-alkylation on the activity varies with the substituents on the D-ring. When the D-ring is phenyl (R=H), the indole-N-methylation hardly impact the activity (**A1** *vs* **A46**). However, when the D-ring is 4′-bromrophenyl, the indole-N-methylation causes the significant increase of the activity (**A10** *vs* **A47**, Table 1). We assume that a similar case might also exist in compounds **B**.

Fourthly, comparison of **B34-B37** and **B1** or **A34-A37** and **A1** suggests that substitution patterns of the A-ring can also dramatically influence the activity. The introduction of 6-OMe, 6-Me, 7-F or 7-Cl to the A-ring leads to obvious increase of the anti-AChE activity of both compounds **B** and **A**. However, different cases exist in aspect of anti-BuChE activity. The substituents on the A-ring can also improve the anti-BuChE activity of compounds **B** (**B37-B37** vs **B1**) but decrease that of compounds **A** (**A37-A37** vs **A1**). Since the substituents above involve different electron effect, we speculated that the effect of substituents on the A-ring might mainly be steric effect but not electron effect.

Finally, besides the substituents on the A- and D-ring and the indole-N-substituents, the aromatization of the C-ring can also intensely influence the anti-AChE activity. Among these factors, there is an interaction effect on the activity. The aromatization of the C-ring dramatically enhances the activity of the indole-N-alkyl compounds (**B1**‒**B45** *vs* **A1**‒**A45**), but hardly influences the activity of the indole-N-H compounds (**A45** *vs* **B45**), indicating that the influence of the aromatization of the C-ring on the activity is related with the indole-N-substituents. Compound **A33** was no activity at 10 μM whereas **B33** showed the highest activity; compound **A7** was more active than compound **A4**, but **B7** was less active than **B4**. This result shows that the influence of the aromatization of the C-ring on the activity also closely varies with the substituents on the D-ring. Furthermore, the presence of 4′-Br can improve the activity of the indole-N-Me compounds (**A10** *vs* **A1**, **B10** *vs* **B1**) but decrease the activity of the indole-N-H compounds (**A47** *vs* **A46**, Table 1), suggesting that an interaction effect on the activity also exists between the substituents on the D-ring and the indole-N-substituents.

For BuChE, a similar interaction effect on the activity also exists among the four factors. For example, for the N_9_-Me compounds, the C-ring aromatization improves that activity of the 4′-F, 4′-Br, 4′-I, 4′-OMe or 4′-Me compounds (**B4** *vs* **A4**, **B10** *vs* **A10**, **B13** *vs* **A13**, **B19** *vs* **A19**, **B22** *vs* **A22**) but decreases the activity of the 4′-Cl, 3′-OH or 4′-OH compounds (**B7** *vs* **A7**, **B15** *vs* **A15**, **B16** *vs* **A16**). For the indole-N-H compound, the C-ring aromatization also causes decrease of the activity (**B46** *vs* **A46**). Interestingly, whether for the indole-N-alkyl compounds or for the indole-N-H compounds, the C-ring aromatization always leads to increase of the selectivity toward AChE (**B1** *vs* **A1**; **B4** *vs* **A4**; **B7** *vs* **A7**; **B10** *vs* **A10**; **B15** *vs* **A15**; **B16** *vs* **A16**; **B19** *vs* **A19**; **B22** *vs* **A22**; **B34**-**45** *vs* **A34**-**45**; **B46** *vs* **A46**) (Table 2).

### Molecular docking of compounds with AChE and BuChE

**Figure 3:**
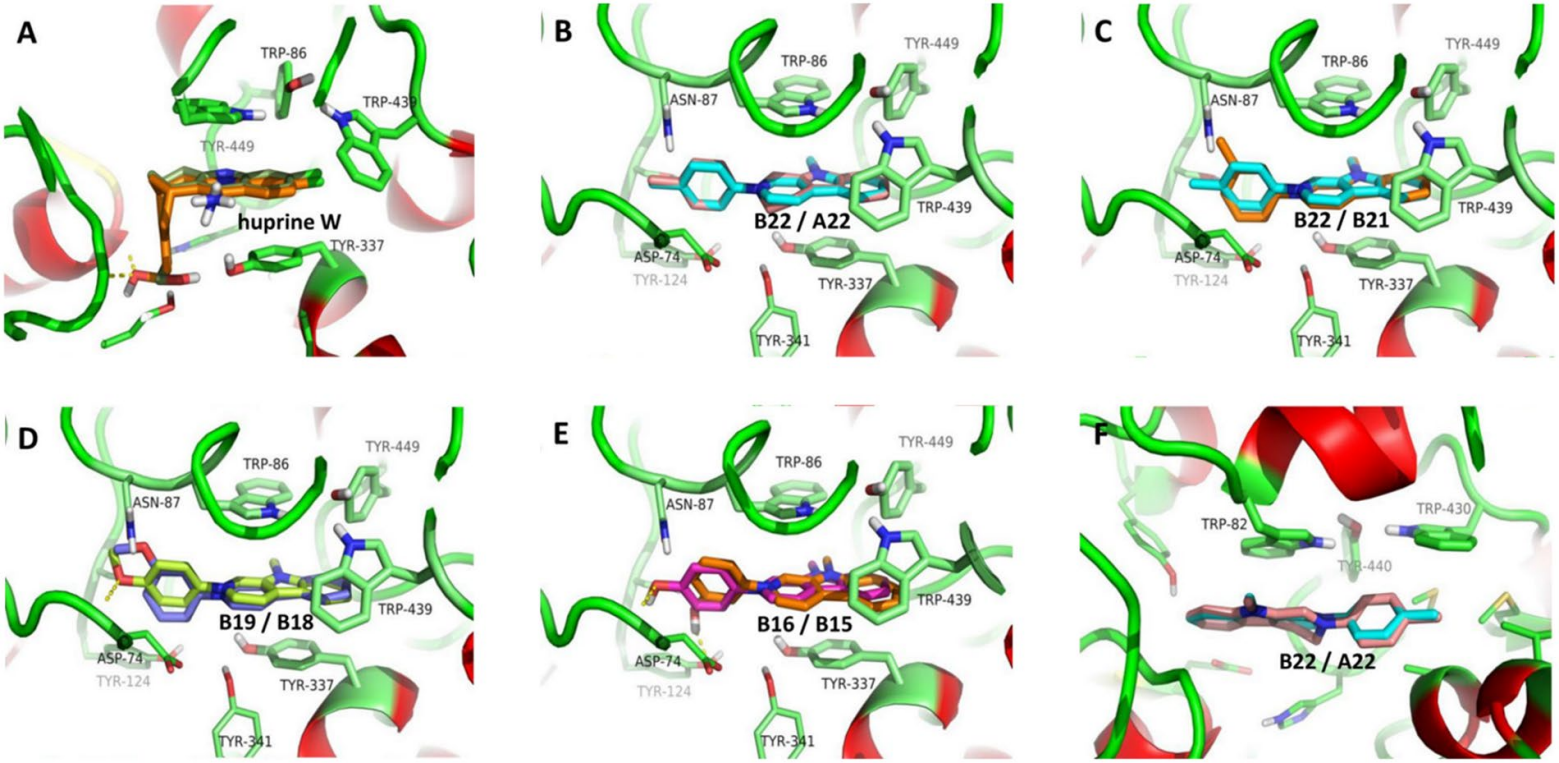
The estimated binding modes of the compounds into the active site of AChE and BuChE. The protein structures are show in Ribbon style with coil in green, helix in red and strand in yellow. The binding site residues are displayed using a stick model with carbon in green. The potential hydrogen contacts between compounds and protein were highlighted by yellow dashed lines. (**A**) Superposition of huprine W in the X-ray crystallographic structure (PDB code 4BDT; orange) and in docking resultant complex structure of the AChE (dark green). (**B**–**E**) The binding modes of the compounds (in stick model with carbon) into the active site of AChE: (**B**) **B22** (cyan) and **A22** (pink); (**C**) **B22** (cyan) and **B21** (goldenrod); (**D**) **B19** (chartreuse) and **B18** (medium blue); (**E**) **B16** (purple) and **B15** (orange red). (**F**) The binding modes of compounds **B22** (cyan) and **A22** (pink) into the active site of BuChE. Other atoms were colored as follows: nitrogen, blue; oxygen, red; hydrogen, gray.

To gain insight into binding interactions of the compounds in the hydrolytic active site of AChE and BuChE, molecular docking studies were performed for the representative compounds **B15**, **B16**, **B18**, **B19**, **B21**, **B22** and **A22**. These studies were performed into an AChE structure (PDB 4BDT) and BuChE (PDB 5K5E). The results are shown in Figure 3 and Table 7. Figure 3A revealed that the binding modes of **huprine W** and **6QS** obtained by AutodDock are almost identical to the crystallographic structure, which proves the feasibility of the docking protocol. The data in Table 5 show that the binding free energies (<−9.3 kcal/mol) of compounds **A** or **B** with the catalytic site of AChE are larger than that (−7.2 to −8.3 kcal/mol) with BuChE, indicating that compounds **A** or **B** have more inhibition potential to AChE than BuChE, agreement with the measured inhibition activities. The results of molecular docking showed that the compounds have very similar binding modes (Fig. 1B–E and F). Figure 3B–E showed that the compounds could be stabilized in the active site of AChE by interactions similar to those described for **huprine W**. The β-carboline moiety is embedded in a remarkable group of aromatic rings including Trp86, Tyr337, Trp439 and Tyr449. The B-ring of the β-carboline is facing Tyr337 while the C-ring is facing Trp86 (Fig. 3B). Obviously, the full aromatic β-carboline moiety should be more beneficial than its 3,4-dihydro structure for enhancing the binding affinity of the ligands for AChE due to the π-π action between the carboline ring and the indole ring of the residue Trp86. The speculation was further supported by the compounds **B** having larger binding free energy with the catalytic site of AChE than compounds **A** (Table 7, **B13** *vs* **A13**, **B15** *vs* **A15**, **B19** *vs* **A19**, **B22** *vs* **A22**).

**Table 7:** The binding free energies of compounds with AChE and BuChE. ^*a*^The inhibitor co-crystals with AChE in the crystal structure of the protein complex (PDB code: 4BDT). ^*b*^The inhibitor co-crystals with BuChE in the crystal structure of the protein complex (PDB code: 5K5E).

Compound	IC_50_ (μM)		FBE (kcal/mol)	
	AChE	BuChE	AChE	BuChE
**A13**	5.5	53.8	−10.38	−7.51
**A15**	6.12	10	−9.38	−7.7
**A16**	2.81	28	−9.48	−7.69
**A19**	2.92	137	−10.04	−7.25
**A22**	1.45	80.1	−9.46	−7.52
**B12**	11.1	21.1	−9.78	−8.26
**B13**	0.24	40.4	−10.46	−7.46
**B15**	3.92	29.6	−9.57	−7.8
**B16**	1.5	67.9	−9.43	−7.76
**B18**	9.69	62.2	−9.46	−7.65
**B19**	0.47	34	−10.11	−7.65
**B21**	5.05	68.6	−9.37	−7.82
**B22**	0.29	32.2	−9.86	−7.38
**B33**	0.11	30.6	−10.28	−7.97
**huprine W** ^***a***^	0.0011		−10.23	/
**6QS** ^***b***^		0.443	/	−11.49

The binding models of **B22**/**21** (Fig. 3C) or **B19**/**18** (Fig. 3D) showed that the 3′-substituents may bring some inappropriate clash penetration by the ligand into the enzyme, whereas 4′-substituents generally increase the binding free energy of AChE-the compound. Similar situations arise in other compounds, such as **B16/B15** (Fig. 3E), **B13/B12**, **A16**/**A15**, *et al*. Interestingly, Fig. 3E showed that the hydrogen atom of 3′-OH group and the phenolic oxygen atom of Tyr124 could form an H-bond (Fig. 3E), which compensates the inappropriate clash penetration, resulting in the comparable binding free energy for **A16** *vs* **A15** or **B16** *vs* **B15**. These results support our presumption above that the effect of the *p*-substituents on the activity should be mainly steric effect but not electron effect or hydrogen-bond effect.

All of the current marketed AChEIs are nitrogen-contained compounds, which are partly protonated at physiological pH. In the acid-base equilibrium, the cations formed by protonation can enhance the overall affinity of AChE for the ligands by additional cation–π interactions with aromatic residues at the active site of AChE, whereas their neutral can cross the blood‒brain barrier (BBB) due to their good lipophilicity^47^. In the present study, ionic compounds **A22** and **B22** showed the higher activity against AChE, but their neutral derivative **C1** lost the activity (Table 2). Obviously, the positive charge of compounds **A** and **B** should be a crucial factor for the activity. Molecular docking showed that the β-carboline moiety locates in the cavity consisting of four aromatic residues of Trp-86, Trp-439, Tyr-337 and Tyr-449 at the active site of AChE (Fig. 3B–E). All the aromatic rings of these residues are rich in π-electrons. Therefore, the positively charged β-carboline moiety with the π-electron poor property can form stronger π-π action with the residues to improve the overall affinity of AChE for the compounds or the activity of the compounds.

**Figure 4:**
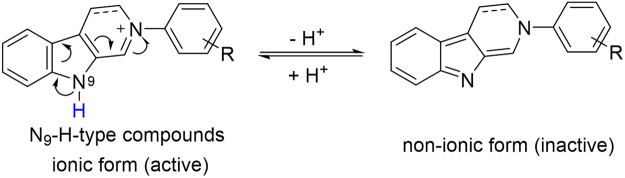
Possible existing forms of N_9_-H-type compounds in an aqueous solution.

Similarly, the low activity of the indole-N-H compounds may also be explained by loss of the positive charge. Theoretically, the indole-N-H has stronger acidity because of the presence of the C=N^+^ moiety adjacent to the N_9_ site. Therefore, the indole-N-H compounds such as **B34** and **A35** can partly release their protons in aqueous solution to form inactive or low active neutral forms (Fig. 4). In other word, the indole-N-H compounds can co-exist in two forms of ion and non-ion in aqueous solution, but only their ionic forms have the good activity. Unlike the indole-N-H compounds, the indole-N-Me compounds always exist in ionic forms. This may be why the indole-N-methylation can increase the activity of compounds **B** (**B1** *vs* **B34**, Table 2) or **A** (**A10** *vs* **A35**, Table 1). Additionally, the similar case was not observed for 2-methyl-*β*-carbolinium salts^41,42^, where their indole-N-substiutents did not significantly influent the activity against AChE. The reason is that the non-ionic form formed by release of the indole-N-H of 2-methyl-*β*-carbolinium salts is unstable because of the lack of conjugation action of the D-aryl ring.

**Figure 5:**
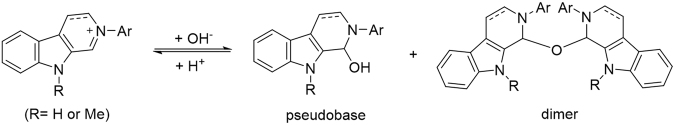
Plausible existing forms of compounds A or B in an aqueous solution.

Sanguinarine and chelerythrine are two quaternary benzo[c]phenanthridine alkaloids, which contain a C=N^+^ moiety with high chemical activity. Studies showed that the two can react with OH^−^ or H_2_O to yield the corresponding pseudobase or dimer by its C=N^+^ moiety, and mainly exist in its dimer in pH 7.0 aqueous solution^48,49^. Therefore, both sanguinarine and chelerythrine can easily cross the membrane of cells to quickly enter living cells although they are ionic compounds^50^. Additionally, we also found the similar chemical transformation for 2-aryl-3,4-dihydroisoquinoliniums containing a C=N^+^ moiety (unpublished). Based on the results above and the structural similarity, we speculated that compounds **A** or **B** could also co-exist in two forms of ion and their corresponding pseudobases or dimers in human blood (pH 7.35–7.45) due to the presence of a C=N^+^ moiety with high chemical activity (Fig. 5). The resulting pseudobase or dimer forms can allow compounds **A** or **B** to cross the blood brain barrier (BBB) due to its good lipophilicity. At present, the investigation on BBB permeability of the compounds is ongoing in our lab.

Besides higher anti-AChE activity and higher selectivity to AChE, compound **B22** also showed the lower cytotoxicity than **A22**, which should be attributed to the aromatization of the C-ring. A similar case was observed for the antifungal activity of compounds **A**, where the aromatization of the C-ring causes reduction of the activity^33^. This similarity might be related with both cytotoxicity and antifungal activity involving inhibition of cell proliferation. Obviously, for potent and high selective β-carboline-type AChE inhibitors for treatment of AD, the full aromatic 9-alkyl-β-carboline skeleton is crucial whereas for anticancer or antimicrobial, the 9-alkyl-3,4-dihydro-β-carboline skeleton is favorable.

In conclusion, a series of new 2-aryl-9-methyl-*β*-carboline bromides (**B**) were synthesized in the present study. All compounds **B** along with most of their 3,4-dihydro intermediates (**A**) showed anti-AChE activities *in vitro* at 10 μM. Among them, nine compounds **B** showed higher anti-AChE activity and selectivity relative to BChE than galantamine, a selective AChE inhibitor drug, which showed great potential to be developed as new selective AChE inhibitor agents. Kinetic analysis proved that both compounds **B** and **A** are able to inhibit AChE in a competitive manner, and the binding capacity of **B22** with AChE reaches up to 3835-fold that of substrate acetylthiocholine iodide (ATCh). In aspect of SAR, the C=N^+^ moiety was proved to be a determinant for the activity, and 4′-substituents of the D-ring, the indole-N-methyl and C-ring aromatization can dramatically increase the anti-AChE activity and selectivity by independent or synergistic effect. These findings will be of great importance for further structural optimization design. Thus, we succeeded in discovering a class of new and promising tool compounds for development of new AChE inhibitor agents.

## Methods

### Chemicals

Acetylthiocholine iodide (ATCh), butyrylthiocholine iodide (BuTCh), 5,5′-dithio-bis(2-nitrobenzoic acid) (DTNB), and galantamine were purchased from Sigma Chemicals Co., Ltd. (Shanghai, China). Phenylhydrazine-HCl and α-ketoglutaric acid were purchased from Aladdin Industrial Inc. (Shanghai, China). Other reagents and solvents were obtained locally and of analytical grade or purified according to standard methods before use. The water used was redistilled and ion-free.

### Enzymes

AChE (E.C. 3.1.1.7) (BR, 200 u/mg) from fly’s head and BuChE (E.C. 3.1.1.8) (BR, 20 u/mg) from horse serum were purchased from Shanghai Yuanye biological technology Co. Ltd (Shanghai, China).

### Instruments

Melting points were determined on a digital melting point apparatus. Nuclear magnetic resonance spectra (NMR) were performed on an Avance III 500 MHz instrument (Bruker, Karlsruhe, Germany). Chemical shifts (*δ* values) and coupling constants (*J* values) are given in parts per million and hertz, respectively. High-resolution mass spectra (HR-MS) were carried out with a microTOFQ II instrument (Bruker). Low-resolution mass spectra were carried out with a LCQ Fleet instrument (Thermo Fisher, Shanghai, China).

### Synthesis

#### General procedure for the synthesis of compounds A1-A47

According to our previous method^33^, intermediates **A1-A47** were prepared *via* 5 or 6 steps starting from phenylhydrazine-HCl and α-ketoglutaric acid. The compounds were confirmed by ^1^H NMR, ^13^C NMR and MS analyses, and their spectral data were agreement with those in literature^23^.

#### General procedure for the synthesis of compounds B1-B46

To a solution of compounds **A** (0.2 mmol) in acetonitrile (40 mL) was added 5% Pd/C (wetted with ca. 55% water) (0.17 mmol, 80 mg). The mixture was refluxed at 80 °C for about 3 days to complete the reaction. The Pd/C powders in the reaction solution was filtered off through a sand core funnel and completely washed with methanol. The combined solution was evaporated under vacuum to yield the desired compounds.

*9-Methyl-2-phenyl-β-carboline bromide (****B1****)*: Yield 78%; yellow powders; mp 271.2**–**272.8 °C; The ^1^H NMR, ^13^C NMR and MS data are consistent with those in the literature^33^.

*9-Methyl-2-(2-fluorophenyl)-β-carboline bromide (****B2****)*: Yield, 29%; golden yellow powders; mp 247.6–249.0 °C; ^1^H NMR (500 MHz, DMSO-*d*_6_) *δ*: 9.99 (1 H, s, H-1), 9.07 (1 H, d, *J* = 6.5 Hz, H-4), 9.00 (1 H, d-like, *J* = 6.5 Hz, H-3), 8.67 (1 H, d, *J* = 8.0 Hz, H-5), 8.04 (1 H, 2 × t, *J* = 7.9, 1.7 Hz, H-6′), 8.01–7.95 (2 H, m, H-7, H-8), 7.84 (1 H, m, H-4′), 7.75 (1 H, t-like, *J* = 9.4, 8.5, 1.3 Hz, H-3′), 7.63 (1 H, t, *J* = 7.7 Hz, H-5′), 7.58 (1 H, 2 × t, *J* = 7.9, 1.5 Hz, H-6), 4.17 (3 H, s, NCH_3_); ^13^C NMR (125 MHz, DMSO-*d*_6_) *δ* 155.4 (d, *J* = 252.1 Hz, C-2′), 146.0 (C-1), 136.4 (C-8a), 134.5 (C-8a), 133.7 (d, *J* = 7.9 Hz, C-4′), 133.5 (C-3), 133.1 (C-9a), 131.5 (d, *J* = 11.7 Hz, C-1′), 131.0 (C-4b), 128.9 (C-6′), 126.3 (d, *J* = 3.8 Hz, C-5′), 124.7 (C-7), 122.7 (C-5), 119.4 (C-6), 118.1 (C-8), 117.7 (d, *J* = 18.8 Hz, C-3′), 112.0 (C-4a), 31.2 (NCH_3_); HR-ESI-MS [M-Br]^+^: Calcd for C_18_H_14_FN_2_^+^, 277.1136, found 277.1170.

*9-Methyl-2-(3-fluorophenyl)-β-carboline bromide (****B3****)*: Yield, 50%; orangered powders; mp 143.0–144.0 °C; ^1^H NMR (500 MHz, DMSO-*d*_6_) *δ* 9.97 (1 H, d, *J* = 1.1 Hz, H-1), 9.04 (2 H, s, H-3, H-4), 8.67 (1 H, d, *J* = 8.0 Hz, H-5), 8.06 (1 H, 2 × t, *J* = 9.5, 2.3 Hz, H-2′), 8.01–7.93 (2 H, m, H-7, H-8), 7.92–7.82 (2 H, m, H-5′, H-6′), 7.66 (1 H, t-like, *J* = 8.4 Hz, H-4′), 7.57 (1 H, 2 × t, *J* = 8.0, 1.4 Hz, H-6), 4.20 (3 H, s, NCH_3_); ^13^C NMR (126 MHz, DMSO-*d*_6_) *δ* 162.5 (d, *J* = 247.1 Hz, H-3′), 145.9 (C-1), 145.0 (d, *J* = 10.3 Hz, H-1′), 136.4 (C-8a), 133.32 (C-3), 133.26 (C-4b), 132.9 (C-9a), 132.5 (d, *J* = 8.8 Hz, C-5′), 129.8 (C-4), 124.7 (C-7), 122.7 (C-5), 122.1 (d, *J* = 3.2 Hz, C-6′), 119.4 (C-6), 118.23 (d, *J* = 21.1 Hz, C-2′), 118.19 (C-8), 113.8 (d, *J* = 26.3 Hz, C-4′), 112.0 (C-4a), 31.0 (NCH_3_); HR-ESI-MS [M-Br]^+^: Calcd for C_18_H_14_FN_2_^+^, 277.1136, found 277.1170.

*9-Methyl-2-(4-fluorophenyl)-β-carboline bromide (****B4****)*: Yield, 76%; yellow powders; mp 296.8–297.8 °C; ^1^H NMR (500 MHz, DMSO-*d*_6_) *δ* 9.95 (1 H, s, H-1), 9.03 (1 H, d, *J* = 6.5 Hz, H-3), 8.99 (1 H, dd, *J* = 6.5, 1.3 Hz, H-2), 8.67 (1 H, d, *J* = 8.0 Hz, H-5), 8.08 (2 H, dd, *J* = 8.8, 4.5 Hz, H-2′, H-6′), 8.01–7.93 (2 H, m, H-7, H-8), 7.67 (2 H, t, *J* = 8.7 Hz, H-3′, H-5′), 7.57 (1 H, 2 × t, *J* = 8.0, 1.3 Hz, H-6), 4.19 (3 H, s, NCH_3_). ^13^C NMR (126 MHz, DMSO-*d*_6_) *δ* 162.9 (d, *J* = 249.0 Hz, C-4′), 145.2 (C-1), 140.0 (d, *J* = 2.9 Hz, C-1′), 136.0 (C-8a), 132.9 (C-3), 132.6 (C-9a), 132.1 (C-4b), 129.4 (C-4), 127.7 (d, *J* = 9.4 Hz, C-2′, C-6′), 124.1 (C-7), 122.1 (C-5), 118.9 (C-6), 117.6 (C-8), 116.9 (d, *J* = 23.6 Hz, C-3′, C-5′), 111.4 (C-4a), 30.5 (NCH_3_); HR-ESI-MS [M-Br]^+^: Calcd for C_18_H_14_FN_2_^+^, 277.1136, found 277.1118.

*9-Methyl-2-(2-chlorophenyl)-β-carboline bromide (****B5****)*: Yield, 36%; golden yellow powders; mp 206.9–207.7 °C; ^1^H NMR (500 MHz, DMSO-*d*_6_) *δ* 9.99 (1 H, d, *J* = 1.1 Hz, H-1), 9.10 (1 H, d, *J* = 6.5 Hz, H-4), 8.96 (1 H, dd-like, *J* = 6.5, 1.4 Hz, H-4), 8.69 (1 H, d, *J* = 8.0 Hz, H-5), 8.04 (dd, *J* = 7.8, 1.4 Hz, H-6′), 8.02–7.96 (2 H, m, H-7, H-8), 7.94 (1 H, d, *J* = 8.0, 1.4 Hz, H-3′), 7.82 (1 H, 2 × t, *J* = 7.8, 1.6 Hz, H-4′), 7.77 (1 H, 2 × t, *J* = 7.7, 1.5 Hz, H-5′), 7.59 (1 H, dt-like, *J* = 8.0, 1.5 Hz, H-6), 4.17 (3 H, s, NCH_3_); ^13^C NMR (126 MHz, DMSO-*d*_6_) *δ* 145.4 (C-1), 140.6 (C-1′), 135.8 (C-8a), 134.0 (C-2′), 133.0 (C-3), 132.8 (C-9a), 132.6 (C-4b), 130.6 (C-4), 130.4 (C-4′), 128.9 (C-3′), 128.8 (C-5′), 128.6 (C-6′), 124.3 (C-7), 122.2 (C-5), 119.0 (C-6), 117.7 (C-8), 111.6 (C-4a), 30.7 (NCH_3_). HR-ESI-MS [M-Br]^+^: Calcd for C_18_H_14_ClN_2_^+^, 293.0840, found 293.0848.

*9-Methyl-2-(3-chlorophenyl)-β-carboline bromide (****B6****)*: Yield, 60%; orangered powders; mp 206.7–207.5 °C; ^1^H NMR (500 MHz, DMSO-*d*_6_) *δ* 9.97 (1 H, d, *J* = 1.1 Hz, H-1), 9.04 (2 H, broad s, H-3, H-4), 8.67 (1 H, d, *J* = 8.0 Hz, H-5), 8.23 (1 H, t, *J* = 2.1 Hz, H-2′), 8.01–7.94 (3 H, m, H-7, H-8, H-6′), 7.86 (1 H, 2 × t, *J* = 8.2, 1.6 Hz, H-4′), 7.83 (1 H, t, *J* = 7.9 Hz, H-5′), 7.57 (1 H, t, *J* = 6.6 Hz, H-6), 4.19 (3 H, s, NCH_3_); ^13^C NMR (126 MHz, DMSO-*d*_6_) *δ* 145.3 (C-1), 144.4 (C-1′), 140.1 (C-3′), 135.9 (C-8a), 134.1 (C-5′), 132.8 (C-3), 132.3 (C-9a), 131.6 (C-4b), 130.6 (C-2′), 129.2 (C-4), 125.5 (C-4′), 124.2 (C-7), 124.1 (C-6′), 122.1 (C-5), 118.8 (C-6), 117.6 (C-8), 111.4 (C-4a), 30.5 (NCH_3_); HR-ESI-MS [M-Br]^+^: Calcd for C_18_H_14_ClN_2_^+^, 293.0840, found 293.0847.

*9-Methyl-2-(4-chlorophenyl)-β-carboline bromide (****B7****)*: Yield, 89%; golden yellow powders; mp 265.5–266.5 °C; ^1^H NMR (500 MHz, DMSO-*d*_6_) *δ* 9.94 (1 H, d, *J* = 1.2 Hz, H-1), 9.03 (1 H, d, *J* = 6.5 Hz, H-4), 9.00 (1 H, dd, *J* = 6.5, 1.2 Hz, H-3), 8.66 (1 H, d, *J* = 8.0 Hz, H-5), 8.05 (2 H, d, *J* = 8.7 Hz, H-3′, H-5′H-2′, H-6′), 8.01–7.92 (2 H, m, H-7, H-8), 7.90 (2 H, d, *J* = 8.7 Hz, H-2′, H-6′), 7.56 (1 H, t, *J* = 7.3 Hz, H-6), 4.19 (3 H, s, NCH_3_). ^13^C NMR (126 MHz, DMSO-*d*_6_) *δ* 145.2 (C-1), 142.3 (C-1′), 136.0 (C-8a), 135.5 (C-4′), 132.8 (C-3), 132.7 (C-3′, C-5′), 132.2 (C-9a), 130.0 (C-4b), 129.2 (C-4), 127.2 (C-2′, C-6′), 124.1 (C-7), 122.1 (C-5), 118.9 (C-6), 117.7 (C-8), 111.5 (C-4a), 30.6 (NCH_3_); HR-ESI-MS [M-Br]^+^: Calcd for C_18_H_14_ClN_2_^+^, 293.0840, found 293.0933; Negative ESI-MS *m*/*z*: 78.68 [^79^Br^−^], 80.70 [^81^Br^−^].

*9-Methyl-2-(2-bromophenyl)-β-carboline bromide (****B8****)*: Yield, 32%; golden yellow powders; mp 212.6–213.5 °C; ^1^H NMR (500 MHz, DMSO-*d*_6_) *δ* 10.00 (1 H, d, *J* = 1.3 Hz, H-1), 9.12 (1 H, d, *J* = 6.4 Hz, H-4), 8.93 (1 H, dd, *J* = 6.4, 1.4 Hz, H-3), 8.70 (1 H, d, *J* = 8.1 Hz, H-5), 8.07 (1 H, dd, *J* = 8.1, 1.4 Hz, H-6′), 8.03 (1 H, dd, *J* = 8.0, 1.6 Hz, H-2′), 8.02–7.96 (2 H, m, H-7, H-8), 7.80 (1 H, 2 × t, *J* = 7.8, 1.4 Hz, H-4′), 7.74 (1 H, 2 × t, *J* = 7.8, 1.6 H, H-5′), 7.59 (1 H, 2 × t, *J* = 8.0, 1.4 Hz, H-6), 4.17(3 H, s, NCH_3_); ^13^C NMR (126 MHz, DMSO-*d*_6_) *δ* 145.9 (C-1), 142.7 (C-1′), 136.3 (C-8a), 134.6 (C-4′), 134.2 (C-3′), 133.5 (C-3), 133.4 (C-5′), 133.2 (C-9a), 130.8 (C-4b), 129.9 (C-4), 129.2 (C-6′), 124.8 (C-7), 122.8 (C-5), 119.4 (C-2′), 119.3 (C-6), 118.1 (C-8), 112.1 (C-4a), 31.1 (NCH_3_); HR-ESI-MS [M-Br]^+^: Calcd for C_18_H_14_BrN_2_^+^, 337.0335 (^79^Br), 339.0314 (^81^Br), found 337.0340, 339.0329.

*9-Methyl-2-(3-bromophenyl)-β-carboline bromide (****B9****)*: Yield, 53%; yellow powders; mp 219.2–220.2 °C; ^1^H NMR (500 MHz, DMSO-*d*_6_) *δ* 9.96 (1 H, s, H-1), 9.03 (2 H, s, H-3, H-4), 8.66 (1 H, d, *J* = 8.0 Hz, H-5), 8.33 (1 H, d, *J* = 2.1 Hz, H-2′), 8.02 (1 H, dd, *J* = 8.2, 2.1 Hz, H-4′), 8.00–7.94 (3 H, m, H-7, H-8, H-6′), 7.75 (1 H, t, *J* = 8.1 Hz, H-5), 7.57 (1 H, t, *J* = 7.3 Hz, H-6), 4.19 (3 H, s, NCH_3_); ^13^C NMR (125 MHz, DMSO-*d*_6_) *δ* 145.8 (C-1), 145.0 (C-1′), 136.4 (C-8a), 134.1 (C-6′), 133.32 (C-5′), 133.30 (C-3), 132.8 (C-9a), 132.4 (C-4b), 129.8 (C-4), 128.7 (C-4′), 125.1 (C-3′), 124.6 (C-7), 122.8 (C-2′), 122.7 (C-5), 119.4 (C-6), 118.1 (C-8), 112.0 (C-4a), 31.0 (NCH_3_). HR-ESI-MS [M-Br]^+^: Calcd for C_18_H_14_BrN_2_^+^, 337.0335 (^79^Br), 339.0314 (^81^Br), found 337.0342, 339.0323.

*9-Methyl-2-(4-bromophenyl)-β-carboline bromide (****B10****)*: Yield, 80%; faint yellow powders; mp 260.0–261.1 °C; ^1^H NMR (500 MHz, DMSO-*d*_6_) *δ* 9.93 (1 H, d, *J* = 1.4 Hz, H-1), 9.03 (1 H, d, *J* = 6.5 Hz, H-3), 9.00 (1 H, dd, *J* = 6.5, 1.4 Hz, H-4), 8.66 (1 H, d, *J* = 8.0 Hz, H-5), 8.03 (2 H, d, *J* = 8.8 Hz, H-2′, H-6′), 7.97 (2 H, d, *J* = 8.8 Hz, H-3′, H-5′), 7.99–7.93 (2 H, m, H-7, H-8), 7.57 (1 H, 2 × t, *J* = 8.0, 1.4 Hz, H-6), 4.18 (3 H, s, NCH_3_); ^13^C NMR (126 MHz, DMSO-*d*_6_) *δ* 145.3 (C-1), 142.7 (C-1′), 136.0 (C-8a), 133.0 (C-3), 132.8 (C-3′), 132.7 (C-5′), 132.2 (C-9a), 131.3 (C-4b), 129.2 (C-4), 127.8 (C-3′), 127.4 (C-5′), 124.2 (C-2′), 124.1 (C-6′), 122.2 (C-5), 118.9 (C-6), 117.7 (C-8), 111.5 (C-4a), 30.6 (NCH_3_). HR-ESI-MS [M-Br]^+^: Calcd for C_18_H_14_BrN_2_^+^, 337.0335 (^79^Br), 339.0314 (^81^Br), found 337.0337, 339.0316.

*9-Methyl-2-(2-iodohenyl)-β-carboline bromide (****B11****)*: Yield, 34%; golden yellow powders; mp 253.6–254.4 °C; ^1^H NMR (500 MHz, DMSO-*d*_6_) *δ* 9.92 (1 H, d, *J* = 1.2 Hz, H-1), 9.09 (1 H, d, *J* = 6.4 Hz, H-4), 8.87 (1 H, dd, *J* = 6.4, 1.4 Hz, H-3), 8.68 (1 H, d, *J* = 8.1 Hz, H-5), 8.23 (1 H, dd, *J* = 7.9, 1.4 Hz, H-6′), 8.02–7.96 (2 H, m, H-7, H-8), 7.93 (1 H, dd, *J* = 7.9, 1.5 Hz, H-2′), 7.78 (1 H, td, *J* = 7.7, 1.4 Hz, H-4′), 7.60 (1 H, td, *J* = 8.0, 2.0 Hz, H-6), 7.53 (1 H, td, *J* = 7.8, 1.5 Hz, H-5′), 4.17 (3 H, s, NCH_3_); ^13^C NMR (126 MHz, DMSO-*d*_6_) *δ* 146.4 (C-1′), 145.9 (C-1), 140.2 (C-3′), 136.3 (C-8a), 134.6 (C-3), 133.5 (C-9a), 133.15 (C-4′), 133.12 (C-4b), 130.6 (C-5′), 130.4 (C-4), 128.2 (C-6′), 124.7 (C-7), 122.8 (C-5), 119.4 (C-6), 118.2 (C-8), 112.1 (C-4a), 96.7 (C-2′), 31.1 (NCH_3_); HR-ESI-MS [M-Br]^+^: Calcd for C_18_H_14_IN_2_^+^, 385.0196, found 385.0198.

*9-Methyl-2-(3-iodohenyl)-β-carboline bromide (****B12****)*: Yield, 60%; brown red powders; mp 243.7–244.0 °C; ^1^H NMR (500 MHz, DMSO-*d*_6_) *δ* 9.93 (1 H, d, *J* = 1.1 Hz, H-1), 9.01(1 H, d, *J* = 6.5 Hz, H-4), 9.00 (1 H, dd, *J* = 6.5, 1.1 Hz, H-3), 8.66 (1 H, d, *J* = 8.0 Hz, H-5), 8.42 (1 H, t, *J* = 1.9 Hz, H-2′), 8.13 (1 H, d, *J* = 8.2 Hz, H-6′), 8.01 (1 H, dd, *J* = 8.1, 2.2 Hz, H-4′), 8.00–7.93 (2 H, m, H-7, H-8), 7.60–7.54 (2 H, m, H-6, H-5′), 4.19 (3 H, s, NCH_3_); ^13^C NMR (126 MHz, DMSO-*d*_6_) *δ* 145.8 (C-1′), 144.8 (C-1), 139.9 (C-4′), 136.4 (C-8a), 134.0 (C-2′), 133.3 (C-5′), 133.2 (C-9a), 132.7 (C-3), 132.2 (C-4b), 129.7 (C-4), 125.3 (C-6′), 124.6 (C-7), 122.6 (C-5), 119.4 (C-6), 118.1 (C-8), 112.0 (C-4a), 96.0 (C-3′), 31.0 (NCH_3_); HR-ESI-MS [M-Br]^+^: Calcd for C_18_H_14_IN_2_^+^, 385.0196, found 385.0188.

*9-Methyl-2-(4-iodohenyl)-β-carboline bromide (****B13****)*: Yield, 92%; orangered powders; mp 281.6–282.8 °C; ^1^H NMR (500 MHz, DMSO-*d*_6_) *δ* 9.92 (1 H, d, *J* = 1.3 Hz, H-1), 9.02 (1 H, d, *J* = 6.5 Hz, H-4), 8.98 (1 H, dd, *J* = 6.5, 1.4 Hz, H-3), 8.65 (1 H, dd, *J* = 8.0, 1.0 Hz, H-5), 8.18 (2 H, d, *J* = 8.7 Hz, H-3′, H-5′), 7.99–7.93 (2 H, m, H-7, H-8), 7.80 (2 H, d, *J* = 8.7 Hz, H-2′, H-6′), 7.56 (1 H, 2 × t, *J* = 8.0, 1.4 Hz, H-6), 4.18 (3 H, s, NCH_3_); ^13^C NMR (126 MHz, DMSO-*d*_6_) *δ* 145.2 (C-1), 143.2 (C-1′), 138.8 (C-3′, H-5′), 136.0 (C-8a), 132.7 (C-3), 132.5 (C-9a), 132.1 (C-4b), 129.0 (C-4), 127.2, 124.1 (C-7), 122.1 (C-5), 118.9 (C-6), 117.7 (C-8), 111.4 (C-4a), 97.7 (C-4′), 30.5 (NCH_3_); HR-ESI-MS [M-Br]^+^: Calcd for C_18_H_14_IN_2_^+^, 385.0196, found 385.0150; Negative ESI-MS *m*/*z*: 78.69 [^79^Br^−^], 80.68 [^81^Br^−^].

*9-Methyl-2-(2-hydroxyhenyl)-β-carboline bromide (****B14****)*: Yield, 33%; red brown powders; mp 189.7–191.2 °C; ^1^H NMR (500 MHz, DMSO-*d*_6_) *δ* 9.72 (1 H, s, H-1), 8.82 (1 H, d, *J* = 6.4 Hz, H-4), 8.76 (1 H, d, *J* = 6.5 Hz, H-3), 8.59 (1 H, d, *J* = 8.0 Hz, H-5), 7.94 (1 H, d, *J* = 8.4 Hz, H-8), 7.89 (1 H, 2 × t, *J* = 8.4, 2.1 Hz, H-7), 7.56–7.48 (2 H, m, H-4′), 7.33 (1 H, dd, *J* = 7.6, 2.0 Hz, H-6′), 7.15 (1 H, d, *J* = 7.9 Hz, H-3′), 7.07 (1 H, 2 × t, *J* = 8.0, 1.8 Hz, H-5′), 6.58 (1 H, 2 × broad s, OH), 4.14 (s, 3 H, NCH_3_); ^13^C NMR (126 MHz, DMSO-*d*_6_) *δ* 145.1 (C-2′), 136.1 (C-1), 134.1 (C-8a), 131.9 (C-3), 131.4 (C-9a), 130.8 (C-4b), 129.6 (C-4′), 128.4 (C-3), 125.9 (C-6′), 125.5 (C-1′), 124.6 (C-7), 124.0 (C-5′), 121.9 (C-5), 119.6 (C-6), 117.0 (C-8), 112.4 (C-3′), 111.5 (C-4a), 30.6 (NCH_3_). HR-ESI-MS [M-Br]^+^: Calcd for C_18_H_15_N_2_O^+^, 275.1179, found 275.1178.

*9-Methyl-2-(3-hydroxyhenyl)-β-carboline bromide (****B15****)*: Yield, 69%; red brown powders; mp 233.8–235.1 °C; ^1^H NMR (500 MHz, DMSO-*d*_6_) *δ* 10.42 (1 H, s, OH), 9.89 (1 H, s, H-1), 8.99 (1 H, d, *J* = 6.5 Hz, H-4), 8.96 (1 H, dd, *J* = 6.5, 1.3 Hz, H-3), 8.65 (1 H, d, *J* = 8.1 Hz, H-5), 8.04–7.89 (2 H, m, H-7, H-8), 7.58 (1 H, t, *J* = 8.0 Hz, H-5′), 7.54 (1 H, 2 × t, *J* = 8.0, 1.4 Hz, H-6), 7.38 (1 H, dd, *J* = 7.8, 2.1 Hz, H-6′), 7.35 (1 H, t, *J* = 2.3 Hz, H-2′), 7.17 (1 H, dd, *J* = 8.2, 2.2 Hz, H-4′), 4.18 (3 H, s, NCH_3_); ^13^C NMR (126 MHz, DMSO-*d*_6_) *δ* 158.5 (C-3′), 145.1 (C-1), 144.5 (C-1′), 136.0 (C-8a), 132.52 (C-9a), 132.48 (C-3), 132.0 (C-4b), 130.9 (C-5′), 128.8 (C-4), 124.0 (C-7), 121.9 (C-5), 118.8 (C-6), 117.6 (C-8), 117.5 (C-6′), 115.3 (C-4′), 112.1 (C-4a), 111.3 (C-2′), 30.4 (NCH_3_); HR-ESI-MS [M-Br]^+^: Calcd for C_18_H_15_N_2_O^+^, 275.1179, found 275.1190.

*9-Methyl-2-(4-hydroxyhenyl)-β-carboline bromide (****B16****)*: Yield, 93%; red brown powders; mp 267.9–268.5 °C; ^1^H NMR (500 MHz, DMSO-*d*_6_) *δ* 9.72 (1 H, s, H-1), 8.88 (1 H, d, *J* = 6.6 Hz, H-4), 8.86 (1 H, d, *J* = 6.6 Hz, H-3), 8.60 (1 H, d, *J* = 8.0 Hz, H-5), 7.95–7.88 (2 H, m, H-7, H-8), 7.56 (2 H, d, *J* = 8.1 Hz, H-2′, H-6′), 7.53 (1 H, t, *J* = 7.3 Hz, H-6), 6.71 (2 H, d, *J* = 8.1 Hz, H-3′, H-5′), 4.18 (1 H, b s, OH), 4.18 (3 H, s, NCH_3_). ^13^C NMR (126 MHz, DMSO-*d*_6_) *δ* 144.7 (C-1), 136.3 (C-4′), 132.1 (C-1′), 132.0 (C-8a), 131.8 (C-3), 130.4 (C-9a), 127.5 (C-4), 125.4 (C-4b), 125.33 (C-2′), 125.31 (C-6′), 123.7 (C-7), 121.7 (C-5), 119.0 (C-6), 118.0 (C-3′), 117.9 (C-5′), 117.6 (C-8), 111.1 (C-4a), 30.4 (NCH_3_); HR-ESI-MS [M-Br]^+^: Calcd for C_18_H_15_N_2_O^+^, 275.1179, found 275.1185.

*9-Methyl-2-(2-methoxylphenyl)-β-carboline bromide (****B17****)*: Yield, 46%; orange powders; mp 181.7–182.4 °C; ^1^H NMR (500 MHz, DMSO-*d*_6_) *δ* 9.85 (1 H, d, *J* = 1.3 Hz, H-1), 8.99 (1 H, d, *J* = 6.4 Hz, H-4), 8.84 (1 H, dd, *J* = 6.5, 1.3 Hz, H-3), 8.64 (1 H, d, *J* = 8.1 Hz, H-5), 8.00–7.94 (2 H, m, H-7, H-8), 7.81 (1 H, dd, *J* = 7.8, 1.6 Hz, H-6′), 7.74 (1 H, 2 × t, *J* = 8.0, 1.7 Hz, H-4′), 7.57 (1 H, 2 × t, *J* = 7.2, 1.7 Hz, H-6), 7.49 (1 H, dd, *J* = 8.5, 1.2 Hz, H-5′), 7.31 (1 H, 2 × t, *J* = 7.7, 1.2 Hz, H-3′), 4.15 (3 H, s, OCH_3_), 3.86 (3 H, s, NCH_3_); ^13^C NMR (125 MHz, DMSO-*d*_6_) *δ* 152.9 (C-2′), 145.7 (C-1), 136.4 (C-8a), 135.0 (C-4′), 133.2 (C-3), 133.0 (C-9a), 132.7 (C-4b), 131.1 (C-4), 127.9 (C-6′), 124.6 (C-7, C-1′), 122.5 (C-5), 121.6 (C-5′), 119.4 (C-6), 117.7 (C-8), 113.9 (C-3′), 111.9 (C-4a), 57.0 (OCH_3_), 31.0 (NCH_3_); HR-ESI-MS [M-Br]^+^: Calcd for C_19_H_17_N_2_O^+^, 289.1335, found 289.1367.

*9-Methyl-2-(3-methoxylphenyl)-β-carboline bromide (****B18****)*: Yield, 70%; yellow powders; mp 204.0–205.1 °C; ^11^H NMR (500 MHz, DMSO-*d*_6_) *δ* 9.93 (1 H, s, H-1), 9.01 (2 H, s, H-3, H-4), 8.66 (1 H, d, *J* = 8.0 Hz, H-5), 8.03–7.92 (2 H, m, H-7, H-8), 7.70 (1 H, t, *J* = 8.2 Hz, H-5′), 7.63 (1 H, t, *J* = 2.2 Hz, H-2′), 7.61–7.52 (2 H, m, H-6, H-6′), 7.33 (1 H, dd, *J* = 8.4, 2.4 Hz, H-4′), 4.19 (3 H, s, OCH_3_), 3.93 (3 H, s, NCH_3_); ^13^C NMR (126 MHz, DMSO-*d*_6_) *δ* 160.6 (C-3′), 145.7 (C-1), 145.1 (C-1′), 136.5 (C-8a), 133.3 (C-5′), 133.2 (C-9a), 132.7 (C-3), 131.5 (C-4b), 129.6 (C-4), 124.6 (C-7), 122.6 (C-5), 119.4 (C-6′), 118.2 (C-6), 117.6 (C-8), 116.9 (C-2′), 111.9 (C-4′), 111.7(C-4a), 56.5 (OCH_3_), 31.1 (NCH_3_); HR-ESI-MS [M-Br]^+^: Calcd for C_19_H_17_N_2_O^+^, 289.1335, found 289.1340.

*9-Methyl-2-(4-methoxylphenyl)-β-carboline bromide (****B19****)*: Yield, 93%; faint yellow powders; mp 225.5–226.8 °C; ^11^H NMR (500 MHz, DMSO-*d*_6_) *δ* 9.87 (1 H, d, *J* = 1.3 Hz, H-1), 8.99 (1 H, d, *J* = 6.5 Hz, H-4), 8.95 (1 H, dd, *J* = 6.5, 1.4 Hz, H-3), 8.65 (1 H, d, *J* = 8.0 Hz, H-5), 7.99–7.92 (2 H, m, H-7, H-8), 7.93 (2 H, d, *J* = 9.0 Hz, H-2′, H-6′), 7.55 (1 H, 2 × t, *J* = 8.0, 1.2 Hz, H-6), 7.32 (2 H, d, *J* = 9.0 Hz, H-3′, H-5′), 4.18 (3 H, s, OCH_3_), 3.92 (3 H, s, NCH_3_); ^13^C NMR (126 MHz, DMSO-*d*_6_) *δ* 160.7 (C-4′), 145.1 (C-1), 136.8 (C-8a), 136.1 (C-1′), 132.8 (C-9a), 132.4 (C-3), 131.7 (C-4b), 129.0 (C-4), 126.4 (C-2′, C-6′), 124.0 (C-7), 122.0 (C-5), 118.9 (C-6), 117.7 (C-8), 115.1 (C-3′, C-5′), 111.3 (C-4a), 55.9 (OCH_3_), 30.5 (NCH_3_); HR-ESI-MS [M-Br]^+^: Calcd for C_19_H_17_N_2_O^+^, 289.1335, found 289.1342; Negative ESI-MS *m*/*z*: 78.68 [^79^Br^−^], 80.67 [^81^Br^−^].

*9-Methyl-2-(2-methylphenyl)-β-carboline bromide (****B20****)*: Yield, 43%; yellow powders; mp 122.6–130.5 °C; ^1^H NMR (500 MHz, DMSO-*d*_6_) *δ* 9.83 (1 H, s, H-1), 9.03 (1 H, d, *J* = 6.3 Hz, H-4), 8.85 (1 H, d, *J* = 6.3 Hz, H-3), 8.68 (1 H, d, *J* = 8.0 Hz, H-5), 8.02–7.93 (2 H, m, H-7, H-8), 7.74 (1 H, d, *J* = 7.8 Hz, H-6′), 7.67 (1 H, t, *J = *8.0 Hz, H-4′), 7.64 (1 H, t, *J* = 8.0 Hz, H-5′), 7.58 (2 H, m, H-6, H-3′), 4.16 (3 H, s, NCH_3_), 2.17 (3 H, s, CH_3_); ^13^C NMR (126 MHz, DMSO-*d*_6_) *δ* 145.2 (C-1), 143.0 (C-1′), 136.0 (C-8a), 133.6 (C-9a), 133.0 (C-3), 132.6 (C-2′), 132.2 (C-4b), 131.5 (C-4), 130.9 (C-3′), 130.0 (C-4′), 127.4 (C-5′), 126.5 (C-6′), 124.1 (C-7), 122.0 (C-5), 119.0 (C-6), 117.6 (C-8), 111.4 (C-4a), 30.5 (NCH_3_), 16.6 (CH_3_); HR-ESI-MS [M-Br]^+^: Calcd for C_19_H_17_N_2_^+^, 273.1386, found 273.1392.

*9-Methyl-2-(3-methylphenyl)-β-carboline bromide (****B21****)*: Yield, 68%; luminous yellow powders; mp 227.9–229.3 °C; ^1^H NMR (500 MHz, DMSO-*d*_6_) *δ* 9.96 (1 H, s, H-1), 9.05 (1 H, d, *J* = 6.5 Hz, H-4), 9.03 (1 H, d, *J* = 6.7 Hz, H-3), 8.69 (1 H, d, *J* = 8.0 Hz, H-5), 8.06–7.94 (2 H, m, H-7, H-8), 7.87 (1 H, s, H-2′), 7.82 (1 H, d, *J* = 8.0 Hz, H-6′), 7.70 (1 H, t, *J* = 7.8 Hz, H-5′), 7.63–7.56 (2 H, m, H-6, H-4′), 4.23 (3 H, s, NCH_3_), 2.54 (3 H, s, CH_3_); ^13^C NMR (126 MHz, DMSO-*d*_6_) *δ* 145.7 (C-1), 144.1 (C-1′), 140.6 (C-3′), 136.6 (C-8a), 133.2 (C-3), 133.1 (C-9a), 132.6 (C-4b), 131.7 (C-2′), 130.4 (C-4′), 129.5 (C-4), 126.0 (C-5′), 124.6 (C-7), 122.7 (C-6′), 122.6 (C-5), 119.4 (C-6), 118.2 (C-8), 111.9 (C-4a), 31.1 (NCH_3_), 21.4 (CH_3_); HR-ESI-MS [M-Br]^+^: Calcd for C_19_H_17_N_2_^+^, 273.1386, found 273.1416.

*9-Methyl-2-(4-methylphenyl)-β-carboline bromide (****B22****)*: Yield, 82%; orange powders; mp 290.2–290.9 °C; ^1^H NMR (500 MHz, DMSO-*d*_6_) *δ* 9.89 (1 H, s, H-1), 9.00 (1 H, d, *J* = 6.5 Hz, H-4), 8.97 (1 H, dd, *J* = 6.5, 1.4 Hz, H-3), 8.65 (1 H, d, *J* = 8.0 Hz, H-5), 8.00–7.92 (2 H, m, H-7, H-8), 7.88 (2 H, d, *J* = 8.3 Hz, H-2′, H-6′), 7.59 (2 H, d, *J* = 8.3 Hz, H-3′, H-5′), 7.56 (1 H, t, *J* = 8.1 Hz, H-6), 4.18 (3 H, s, NCH_3_), 2.49 (3 H, s, CH_3_); ^13^C NMR (126 MHz, DMSO-*d*_6_) *δ* 145.1 (C-1), 141.3 (C-4′), 140.7 (C-1′), 136.1 (C-8a), 132.7 (C-9a), 132.5 (C-3), 131.9 (C-4b), 130.4 (C-3′, C-5′), 128.9 (C-4), 124.8 (C-2′, C-6′), 124.0 (C-7), 122.0 (C-5), 118.9 (C-6), 117.7 (C-8), 111.4 (C-4a), 30.5 (NCH_3_), 20.6 (CH_3_); HR-ESI-MS [M-Br]^+^: Calcd for C_19_H_17_N_2_^+^, 273.1386, found 273.1390; Negative ESI-MS *m*/*z*: 78.72 [^79^Br^−^], 80.72 [^81^Br^−^].

*9-Methyl-2-(2-cyanophenyl)-β-carboline bromide (****B23****)*: Yield, 57%; brown yellow powders; mp 179.5–179.8 °C; ^1^H NMR (500 MHz, DMSO-*d*_6_) *δ* 10.08 (1 H, s, H-1), 9.14 (1 H, d, *J* = 6.5 Hz, H-4), 9.12 (1 H, d, *J* = 6.5 Hz, H-3), 8.68 (1 H, d, *J* = 8.0 Hz, H-5), 8.34 (1 H, d, *J* = 7.8 Hz, H-6′), 8.21–8.12 (2 H, m, H-3′, H-4′), 8.06–7.96 (3 H, m, H-7, H-8, H-5′), 7.61 (1 H, 2 × t-like, *J* = 8.0, 1.6 Hz, H-6), 4.18 (3 H, s, NCH_3_); ^13^C NMR (126 MHz, DMSO-*d*_6_) *δ* 145.6 (C-1), 144.3 (C-1′), 135.7 (C-8a), 135.0 (C-5′), 134.2 (C-3′), 133.5 (C-4′), 133.2 (C-9a), 132.9 (C-3), 131.8 (C-4b), 130.2 (C-3), 128.1 (C-6′), 124.3 (C-7), 122.4 (C-5), 118.9 (C-6), 117.6 (C-8), 114.8 (C≡N), 111.6 (C-4a), 109.4 (C-2′), 30.6 (NCH_3_); HR-ESI-MS [M-Br]^+^: Calcd for C_19_H_14_N_3_^+^, 284.1182, found 284.1193.

*9-Methyl-2-(3-cyanophenyl)-β-carboline bromide (****B24****)*: Yield, 79%; orange-yellow powders; mp 271.0–272.8 °C; ^1^H NMR (500 MHz, DMSO-*d*_6_) *δ* 10.0 (1 H, s, H-1), 9.08 (1 H, s, H-4), 8.67 (1 H, d, *J* = 8.0 Hz, H-5), 8.63 (1 H, br s, H-3), 8.37 (1 H, dd, *J* = 8.2, 2.3 Hz, H-6′), 8.27 (1 H, d, *J* = 7.9 Hz, H-4′), 8.03 (1 H, d, *J* = 7.9 Hz, H-4′), 8.01–7.95 (2 H, m, H-7, H-8), 7.99 (1 H, s, H-3′), 7.58 (1 H, 2 × t, *J* = 8.0, 1.4 Hz, H-6), 4.20 (3 H, s, NCH_3_); ^13^C NMR (126 MHz, DMSO-*d*_6_) *δ* 147.0 (C-1′), 145.3 (C-1), 143.6 (C-4′), 135. 9 (C-8a), 134.3 (C-5′), 132.9 (C-9a), 132.7 (C-3), 132.4 (C-4b), 131.4 (C-6′), 130.3 (C-4), 129.3 (C-2′), 124.2 (C-7), 122.2 (C-5), 118.8 (c-6), 117.7 (C-8), 117.5 (C≡N), 112.7 (C-3′), 111.5 (C-4a), 30.5 (NCH_3_); HR-ESI-MS [M-Br]^+^: Calcd for C_19_H_14_N_3_^+^, 284.1182, found 284.1187.

*9-Methyl-2-(4-cyanophenyl)-β-carboline bromide (****B25****)*: Yield, 74%; orange-yellow powders; mp 188.6–189.9 °C; ^1^H NMR (500 MHz, DMSO-*d*_6_) *δ* 10.0 (1 H, d, *J* = 1.2 Hz, H-1), 9.07 (1 H, d-like, *J* = 6.5 Hz, H-4), 9.04 (1 H, dd-like, *J* = 6.5, 1.2 Hz, H-3), 8.67 (1 H, d, *J* = 8.0 Hz, H-5), 8.34 (2 H, d, *J* = 8.7 Hz, H-2′, H-6′), 8.24 (2 H, d, *J* = 8.7 Hz, H-3′, H-5′), 8.03–7.94 (2 H, m, H-7, H-8), 7.58 (1 H, 2 × t, *J* = 8.0, 1.4 Hz, H-6), 4.20 (3 H, s, NCH_3_); ^13^C NMR (126 MHz, DMSO-*d*_6_) *δ* 146.4 (C-1′), 145.4 (C-1), 135.9 (C-8a), 134.2 (C-3′, C-5′), 132.9 (C-3), 132.6 (C-9a), 132.5 (C-4b), 129.2 (C-4), 126.6 (C-2′, C-6′), 124.2 (C-7), 122.2 (C-5), 118.9 (C-6), 117.74 (C-8), 117.71 (C-4′), 113.5 (C≡N), 111.5 (C-4a), 30.6 (NCH_3_); HR-ESI-MS [M-Br]^+^: Calcd for C_19_H_14_N_3_^+^, 284.1182, found 284.1180.

*9-Methyl-2-(3-trifluoromethylphenyl)-β-carboline bromide (****B26****)*: Yield, 82%; orange-yellow powders; mp 125.4–126.1 °C; ^1^H NMR (500 MHz, DMSO-*d*_6_) *δ* 10.0 (1 H, s, H-1), 9.08 (1 H, dd, *J* = 6.5, 1.3 Hz, H-3), 9.06 (1 H, d, *J* = 6.5 Hz, H-4), 8.68 (1 H, d, *J* = 8.0 Hz, H-5), 8.48 (1 H, s, H-2′), 8.33 (1 H, dd, *J* = 8.0, 2.2 Hz, H-6′), 8.16 (1 H, d, *J* = 7.9 Hz, H-4′), 8.05 (1 H, t, *J* = 8.0 Hz, H-5′), 8.02–7.94 (2 H, m, H-7, H-8), 7.58 (1 H, 2 × t, *J* = 8.0, 1.3 Hz, H-6), 4.20 (3 H, s, NCH_3_); ^13^C NMR (126 MHz, DMSO-*d*_6_) *δ* 145.3 (C-1), 143.9 (C-1′), 135.9 (C-8a), 133.0 (C-3), 132.8 (C-9a), 132.3 (C-4b), 131.4 (C-4), 130.4 (q, *J* = 33.0 Hz, C-3′), 129.7 (C-5′), 129.5 (C-6′), 127.5 (q, *J* = 3.7 Hz, C-4′), 124.2 (C-7), 123.4 (q, *J* = 273.3 Hz, CF_3_), 122.7 (q, *J* = 4.0 Hz, C-2′), 122.2 (C-5), 118.9 (C-6), 117.6 (C-8), 111.5 (C-4a), 30.6 (NCH_3_); HR-ESI-MS [M-Br]^+^: Calcd for C_19_H_14_F_3_N_2_^+^, 327.1104, found 327.1081.

*9-Methyl-2-(4-trifluoromethylphenyl)-β-carboline bromide (****B27****)*: Yield, 86%; yellow powders; mp 152.7–153.8 °C; ^1^H NMR (500 MHz, DMSO-*d*_6_) *δ* 10.0 (1 H, s, H-1), 9.07 (2 H, s, H-3, H-4), 8.67 (1 H, d, *J* = 8.0 Hz, H-5), 8.26 (2 H, d, *J* = 8.6 Hz, H-2′, H-6′), 8.22 (2 H, d, *J* = 8.6 Hz, H-3′, H-5′), 8.03–7.94 (2 H, m, H-7, H-8), 7.58 (1 H, 2 × t, *J* = 8.0, 1.4 Hz, H-6), 4.20 (3 H, s, NCH_3_); ^13^C NMR (126 MHz, DMSO-*d*_6_) δ 146.3 (d-like, *J* = 4.5 Hz, H-1′), 145.3 (C-1), 135.9 (C-8a), 132.9 (C-3), 132.7 (C-9a), 132.4 (C-4b), 130.8 (q, *J* = 32.3 Hz, C-4′), 129.3 (C-4), 127.3 (q, *J* = 3.8 Hz, C-3′, C-5′), 126.6 (C-2′, C-6′), 123.6 (q, *J* = 273.5 Hz, CF_3_), 124.2 (C-7), 122.2 (C-5), 118.9 (C-6), 117.7 (C-8), 111.5 (C-4a), 30.6 (NCH_3_); HR-ESI-MS [M-Br]^+^: Calcd for C_19_H_14_F_3_N_2_^+^, 327.1104, found 327.1130.

*9-Methyl-2-(3-nitrophenyl)-β-carboline bromide (****B28****)*: Yield, 79%; orange-yellow powders; mp 182.2–183.5 °C; ^1^H NMR (500 MHz, DMSO-*d*_6_) *δ* 10.1 (1 H, d, *J* = 1.3 Hz, H-1), 9.09 (1 H, dd, *J* = 6.5, 1.4 Hz, H-3), 9.07 (1 H, d, *J* = 6.5 Hz, H-4), 8.96 (1 H, t, *J* = 2.3 Hz, H-2′), 8.68 (1 H, d, *J* = 8.1 Hz, H-5), 8.61 (1 H, dd, *J* = 8.2, 2.1 Hz, H-6′), 8.48 (1 H, dd, *J* = 7.8, 2.2 Hz, H-4′), 8.10 (1 H, t, *J* = 8.2 Hz, H-5′), 8.04–7.95 (2 H, m, H-7, H-8), 7.58 (1 H, dt-like, *J* = 8.0, 1.3 Hz, H-6), 4.21 (3 H, s, NCH_3_); ^3^C NMR (126 MHz, DMSO-*d*_6_) *δ* 148.2 (C-3′), 145.4 (C-1), 143.8 (C-1′), 135.9 (C-8a), 132.92 (C-3), 132.89 (C-9a), 132.5 (C-4b), 132.0 (C-5′), 131.6 (C-6′), 129.6 (C-4), 125.4 (C-4′), 124.2 (C-7), 122.2 (C-5), 121.0 (C-2′), 118.9 (C-6), 117.7 (C-8), 111.5 (C-4a), 30.6 (NCH_3_); HR-ESI-MS [M-Br]^+^: Calcd for C_18_H_14_N_3_O_2_^+^, 304.1081, found 304.1089.

*9-Methyl-2-(2,6-difluorophenyl)-β-carboline bromide (****B29****)*: Yield, 68%; yellow powders; mp 259.5–261.0 °C; ^1^H NMR (500 MHz, DMSO-*d*_6_) *δ* 10.10 (1 H, s, H-1), 9.15 (1 H, d, *J* = 6.5 Hz, H-4), 9.06 (1 H, d, *J* = 6.4 Hz, H-3), 8.67 (1 H, d, *J* = 8.1 Hz, H-5), 8.03–7.98 (2 H, m, H-7, H-8), 7.92 (1 H, nonet, *J* = 8.7, 6.3 Hz, H-4′), 7.66 (2 H, t, *J* = 8.8 Hz, H-3′, H-5′), 7.60 (1 H, dt-like, *J* = 8.0, 2.2 Hz, H-6), 4.14 (3 H, s, NCH_3_); ^13^C NMR (126 MHz, DMSO-*d*_6_) *δ* 156.1 (d, *J = *254.2 Hz, C-2′, C-6′), 145.8 (C-1), 136.0 (C-8a), 134.4 (C-3), 133.8 (C-9a), 133.5 (C-4b), 133.2 (C-4), 131.2 (C-4′), 124.4 (C-7), 122.4 (C-5), 119.0 (C-6), 118.0 (C-8), 113.6 (d, *J = *12.3 Hz, C-1′), 113.2 (dd, *J* = 18.8, 3.2 Hz, C-3′, C-5′), 111.7 (C-4a), 30.6 (NCH_3_); HR-ESI-MS [M-Br]^+^: Calcd for C_18_H_13_F_2_N_2_^+^, 295.1041, found 295.1049.

*9-Methyl-2-(2,4-dichlorophenyl)-β-carboline bromide (****B30****)*: Yield, 63%; brown powders; mp 215.6–216.8 °C; ^1^H NMR (500 MHz, DMSO-*d*_6_) *δ* 9.97 (1 H, d, *J* = 1.3 Hz, H-1), 9.10 (1 H, d, *J* = 6.4 Hz, H-4), 8.94 (1 H, dd, *J* = 6.4, 1.3 Hz, H-3), 8.67 (1 H, d, *J* = 8.0 Hz, H-5), 8.21 (1 H, d, *J* = 2.3 Hz, H-3′), 8.07 (1 H, d, *J* = 8.5 Hz, H-6′), 8.05–7.95 (2 H, m, H-7, H-8), 7.91 (1 H, dd, *J* = 8.5, 2.3 Hz, H-5′), 7.59 (1 H, dt-like, *J* = 8.0, 2.2 Hz, H-6), 4.15 (3 H, s, NCH_3_); ^13^C NMR (126 MHz, DMSO-*d*_6_) *δ* 145.5 (C-1), 139.5 (C-1′), 136.8 (C-4′), 136.6 (C-2′), 135.8 (C-8a), 133.9 (C-3), 133.1 (C-9a), 132.8 (C-4b), 130.4 (C-3′), 130.2 (C-5′), 130.1 (C-4), 128.9 (C-6′), 124.2 (C-7), 122.3 (C-5), 118.9 (C-6), 117.6 (C-8), 111.6 (C-4a), 30.6 (NCH_3_); HR-ESI-MS [M-Br]^+^: Calcd for C_18_H_13_Cl_2_N_2_^+^, 327.0450 (^35^Cl), 329.0421 (^37^Cl) found 327.0458, 329.0428.

*9-Methyl-2-(3,5-dichlorophenyl)-β-carboline bromide (****B31****)*: Yield, 73%; yellow powders; mp 272.6–273.8 °C; ^1^H NMR (500 MHz, DMSO-*d*_6_) *δ* 10.01 (1 H, s, H-1), 9.08 (2 H, t-like, *J* = 6.0 Hz, H-3, H-4), 8.69 (1 H, d, *J* = 8.0 Hz, H-5), 8.27 (2 H, d, *J* = 1.8 Hz, H-2′, H-6′), 8.13 (1 H, t, *J* = 1.8 Hz, H-4′), 8.08–7.94 (2 H, m, H-7, H-8), 7.61 (1 H, dt-like, *J* = 8.0, 1.6 Hz, H-6), 4.22 (3 H, s, NCH_3_). ^13^C NMR (126 MHz, DMSO-*d*_6_) *δ* 145.4 (C-1), 144.8 (C-1′), 135.8 (C-8a), 135.0 (C-3′, C-5′), 133.0 (C-3), 132.8 (C-9a), 132.5 (C-4b), 130.3 (C-4′), 129.3 (C-4), 124.8 (C-7), 124.2 (C-2′, C-6′), 122.2 (C-5), 118.8 (C-6), 117.6 (C-8), 111.5 (C-4a), 30.6 (NCH_3_); HR-ESI-MS [M-Br]^+^: Calcd for C_18_H_13_Cl_2_N_2_^+^, 327.0450 (^35^Cl), 329.0421 (^37^Cl) found 327.0429, 329.0399.

*9-Methyl-2-(2-fluoro-4-bromophenyl)-β-carboline bromide (****B32****)*: Yield, 56%; orange-yellow powders; mp 211.2–212.9 °C; ^1^H NMR (500 MHz, DMSO-*d*_6_) *δ* 9.97 (1 H, s, H-1), 9.07 (1 H, d, *J* = 6.5 Hz, H-4), 8.98 (1 H, dt, *J* = 6.4, 1.5 Hz, H-3), 8.66 (1 H, d, *J* = 8.0 Hz, H-5), 8.18 (1 H, dd, *J* = 9.9, 2.1 Hz, H-6′), 8.04–7.96 (3 H, m, H-7, H-8, H-5′), 7.89 (1 H, dt, *J* = 8.5, 1.5 Hz, H-5′), 7.59 (1 H, dt-like, *J* = 8.0, 1.7 Hz, H-6), 4.16 (3 H, s, NCH_3_); ^13^C NMR (126 MHz, DMSO-*d*_6_) *δ* 145.5 (C-1), 139.5 (C-1′), 136.8 (C-4′), 136.6 (C-2′), 135.8 (C-8a), 133.9 (C-3), 133.1 (C-9a), 132.8 (C-4b), 130.4 (C-3′), 130.2 (C-5′), 130.1 (C-4), 128.9 (C-6′), 124.2, 122.3, 118.9, 117.6 (C-8), 111.6 (C-4a), 30.6 (NCH_3_); HR-ESI-MS [M-Br]^+^: Calcd for C_18_H_13_BrFN_2_^+^, 355.0241 (^79^Br), 357.0220 (^81^Br) found 355.0244, 357.0223.

*9-Methyl-2-(2,4-dibromophenyl)-β-carboline bromide (****B33****)*: Yield, 55%; yellow powders; mp 258.4–258.7 °C; ^1^H NMR (500 MHz, DMSO-*d*_6_) *δ* 9.97 (1 H, d, *J* = 1.3 Hz, H-1), 9.10 (1 H, d, *J* = 6.4 Hz, H-4), 8.92 (1 H, dd, *J* = 6.4, 1.3 Hz, H-4), 8.68 (1 H, d, *J* = 8.1 Hz, H-5), 8.41 (1 H, d, *J* = 2.0 Hz, H-3′), 8.06 (1 H, dd, *J* = 8.5, 2.2 Hz, H-5′), 8.03–7.94 (3 H, m, H-7, H-8, H-6′), 7.59 (1 H, dt-like, *J* = 8.0, 1.7 Hz, H-6), 4.16 (3 H, s, NCH_3_); ^13^C NMR (126 MHz, DMSO-*d*_6_) *δ* 145.5 (C-1), 141.5 (C-1′), 135.7 (C-3′), 135.6 (C-8a), 133.9 (C-3), 133.1 (C-9a), 132.7 (C-4b), 132.3 (C-5′), 130.2 (C-6′), 130.1 (C-4), 125.1 (C-2′), 124.2 (C-7), 122.3 (C-5), 120.4 (C-4′), 118.9 (C-6), 117.6 (C-8), 111.6 (C-4a), 30.6 (NCH_3_); HR-ESI-MS [M-Br]^+^: Calcd for C_18_H_13_Br_2_N_2_^+^, 414.9440 (2 × ^79^Br), 416.9420 (^79^Br + ^81^Br), 418.9399 (2 × ^81^Br) found 414.9439, 416.9418, 418.9398.

*6-Methoxy-9-methyl-2-phenyl-β-carboline bromide (****B34****)*: Yield, 88%; orange-yellow powders; ^1^H NMR (500 MHz, MeOD) *δ* 9.64 (1 H, s, H-1), 8.82 (1 H, d, *J* = 6.5 Hz, H-4), 8.73 (1 H, d, *J* = 6.5 Hz, H-3), 8.00 (1 H, d, *J* = 2.6 Hz, H-5), 7.91 (2 H, d-like, *J* = 6.6 Hz), 7.82 (1 H, d, *J* = 9.2 Hz), 7.78–7.74 (3 H, m), 7.58 (1 H, dd, *J* = 9.2, 2.7 Hz), 4.17 (NCH_3_), 3.99 (OCH_3_); ^13^C NMR (126 MHz, CD_3_OD) *δ* 157.5 (C-6), 145.6 (C-1), 142.8 (C-1′), 138.0, 133.8, 132.9, 132.1, 131.6 (C-3′, C-5′), 129.5, 126.1 (C-2′, C-6′), 125.8, 121.2, 118.7, 113.3, 104.6, 56.6 (OCH_3_), 30.9 (NCH_3_); HR-ESI-MS [M-Br]^+^: Calcd for C_21_H_21_N_2_^+^, 289.1335 found 289.1342.

*6,9-Dimethyl-2-phenyl-β-carboline bromide (****B35****)*: Yield, 90%; orange-yellow powders; ^1^H NMR (500 MHz, MeOD) *δ* 9.66 (1 H, s, H-1), 8.81(1 H, d, *J* = 6.5 Hz, H-4), 8.79 (1 H, d, *J* = 6.5 Hz, H-3), 8.34 (1 H, s, H-5), 7.94 (2 H, d, *J* = 6.9 Hz, H-2′, H-6′), 7.79–7.82 (5 H, m), 4.19 (3 H, NCH_3_), 2.63 (3 H, CH_3_); ^13^C NMR (126 MHz, MeOD) *δ* 145.9 (C-1), 145.6 (C-1′), 138.1, 136.1, 134.2, 133.9, 133.5, 132.1, 131.6 (C-3′, C-5′), 129.3, 126.1 (C-2′, C-6′), 124.0, 120.9, 118.6, 111.9, 30.9 (NCH_3_), 21.4 (CH_3_); HR-ESI-MS [M-Br]^+^: Calcd for C_19_H_17_N_2_^+^, 273.1386 found 273.1393.

*7-Fluoro-9-methyl-2-phenyl-β-carboline bromide (****B36****)*: Yield, 93%; orange-yellow powders; ^1^H NMR (500 MHz, CD_3_Cl) *δ* 9.94 (1 H, s, H-1), 9.06 (1 H, dd, *J* = 6.5, 1.2 Hz, H-3), 9.02 (1 H, d, *J* = 6.5 Hz, H-4), 8.75 (1 H, dd, *J* = 8.5, 5.5 Hz, H-5), 8.01 (2 H, d-like, *J* = 8.0 Hz, H-8), 7.94 (1 H, dd, *J* = 10.0, 2.0 Hz, H-8), 7.84–7.78 (3 H, m), 7.50–7.47 (1 H, m), 4.19 (3 H, s, CH_3_); ^13^C NMR (126 MHz, DMSO-*d*_6_) *δ* 165.2 (d, *J* = 249.2 Hz, C-7), 146.6 (d, *J* = 13.7 Hz, C-1), 143.6 (C-1′), 136.9 (d, *J* = 1.2 Hz, C-8a), 133.5 (C-3), 132.1 (C-4′), 130.8 (C-9a), 130.2 (C-3′, C-5′), 129.1, 128.9, 126.6 (d, *J* = 11.4 Hz, C-5), 125.5 (C-4), 125.2 (C-2′, C-6′), 117.6 (C-4b), 115.8 (C-4a), 111.3 (d, *J* = 25.7 Hz, C-8), 98.2 (d, *J* = 27.5 Hz, C-6), 30.9 (NCH_3_); HR-ESI-MS [M-Br]^+^: Calcd for C_18_H_14_FN_2_^+^, 277.1136 found 277.1145.

*7-Chloro-9-methyl-2-phenyl-β-carboline bromide (****B37****)*: Yield, 93%; orange-yellow powders; ^1^H NMR (500 MHz, CD_3_Cl) *δ* 9.98 (1 H, s, H-1), 9.08–9.04 (2 H, m), 8.80 (1 H, d, *J* = 6.0 Hz, H-4), 8.70 (1 H, d, *J* = 8.5 Hz, H-5), 8.20 (1 H, d, *J* = 1.6 Hz, H-8), 8.03–8.01 (2 H, m), 7.84–7.78 (3 H, m), 7.63 (1 H, dd, *J = *8.5, 1.7 Hz, H-6), 7.48 (1 H,d, *J* = 8.0 Hz, H-4′), 7.1.2 (1 H, d, *J* = 7.4 Hz, H-6), 4.20 (3 H, s, CH_3_); ^13^C NMR (126 MHz, DMSO-*d*_6_) *δ* 145.7 (C-1), 143.6 (C-1′), 136.7 (C-8a), 133.5, 130.8, 130.2 (C-3′, C-5′), 129.6, 128.0, 125.8, 125.5, 125.2 (C-2′, C-6′), 122.7, 118.0, 118.2 (C-8), 111.7 (C-4a), 30.8 (NCH_3_); HR-ESI-MS [M-Br]^+^: Calcd for C_18_H_14_ClN_2_^+^, 293.0840 found 293.0851.

*9-Ethyl-2-phenyl-β-carboline bromide (****B38****)*: Yield, 90%; orange-yellow powders; ^1^H NMR (500 MHz, CD_3_Cl) *δ* 10.1 (1 H, s, H-1), 8.80 (1 H, d, *J* = 6.0 Hz, H-4), 8.71 (1 H, d, *J* = 6.0 Hz, H-3), 8.31 (1 H, d, *J* = 8.0 Hz, H-5), 8.06 (2 H, d, *J* = 7.5 Hz, H-2′, H-6′), 7.78 (1 H, t, *J* = 7.5 Hz), 7.61 (1 H, d, *J* = 8.4 Hz), 7.56 (2 H, t, *J* = 7.5 Hz, H-3′, H-5′), 7.48 (1 H, t, *J* = 7.5 Hz), 7.40 (1 H, t, *J* = 7.4 Hz, H-4′), 4.96 (2 H, q, *J* = 7.0 Hz, H-1″), 1.49 (3 H, t, *J* = 7.0 Hz, H-2″); ^13^C NMR (126 MHz, CD_3_Cl) *δ* 144.7 (C-1), 143.1 (C-1′), 135.5 (C-8a), 133.0 (C-3), 132.9 (C-9a), 131.9 (C-4′), 130.7 (C-3′, C-5′), 130.5 (C-4b), 127.8 (C-4), 124.8 (C-7), 124.0 (C-2′, C-6′), 122.4 (C-5), 119.5 (C-6), 118.3 (C-8), 110.8 (C-4a), 40.2 (C-1″), 14.5 (C-2″); HR-ESI-MS [M-Br]^+^: Calcd for C_19_H_17_N_2_^+^, 273.1386 found 273.1385.

*9-Propyl-2-phenyl-β-carboline bromide (****B39****)*: Yield, 86%; orange-yellow powders; ^1^H NMR (500 MHz, CD_3_Cl) *δ* 10.1 (1 H, s, H-1), 8.81 (1 H, d, *J* = 6.0 Hz, H-4), 8.72 (1 H, d, *J* = 6.0 Hz, H-3), 8.30 (1 H, d, *J* = 7.8 Hz, H-5), 8.05 (2 H, d, *J* = 7.5 Hz, H-2′, H-6′), 7.77 (1 H, t, *J* = 7.4 Hz), 7.59 (1 H, d, *J* = 8.3 Hz), 7.54 (2 H, t, *J* = 7.4 Hz, H-3′, H-5′), 7.46 (1 H, t, *J* = 6.9 Hz), 7.38 (1 H, t, *J* = 7.4 Hz, H-4′), 4.86 (2 H, t, *J* = 6.7 Hz, H-1″), 1.97–1.87 (2 H, m, H-2″), 0.94 (3 H, t, *J* = 7.0 Hz, H-3″); ^13^C NMR (126 MHz, CD_3_Cl) *δ* 145.2 (C-1), 143.1 (C-1′), 136.1, 132.92, 132.86, 132.0, 130.7, 130.4 (C-3′, C-5′), 127.9, 124.9 (C-2′, C-6′), 124.0, 122.4, 119.3, 118.4, 111.0, 46.5 (C-1″), 22.7 (C-2″), 11.5 (C-3″); HR-ESI-MS [M-Br]^+^: Calcd for C_20_H_19_N_2_^+^, 287.1543 found 287.1538.

*9-Iso-propyl-2-phenyl-β-carboline bromide (****B40****)*: Yield, 85%; orange-yellow powders; ^1^H NMR (500 MHz, CDCl_3_) *δ* 10.1 (1 H, s, H-1), 8.84 (1 H, d, *J* = 6.4 Hz, H-4), 8.76 (1 H, d, *J* = 6.4 Hz, H-3), 8.34 (1 H, d, *J* = 8.0 Hz, H-5), 8.06–8.04 (2 H, m), 7.82 (1 H, d, *J* = 8.5 Hz), 7.76 (1 H, t, *J* = 7.7 Hz), 7.55 (2 H, t, *J* = 7.7 Hz), 7.48 (1 H, t, *J* = 7.7 Hz), 7.40 (1 H, t, *J* = 7.5 Hz), 5.84 (1 H, p, *J* = 7.1 Hz, NCHMe_2_), 1.77 (6 H, d, *J* = 6.9 Hz, 2 × CH_3_); ^13^C NMR (126 MHz, CDCl_3_) *δ* 143.9 (C-1), 143.3 (C-1′), 135.5 (C-8a), 133.0 (C-3), 132.6, 132.0, 130.8, 130.5 (C-3′, C-5′), 128.1, 124.9 (C-2′, C-6′), 124.2, 122.2, 120.3, 118.3 (C-8), 113.2 (C-4a), 49.8 (NCHMe_2_), 21.3 (2 × CH_3_); HR-ESI-MS [M-Br]^+^: Calcd for C_20_H_19_N_2_^+^, 287.1543 found 287.1539.

*9-Allyl-2-phenyl-β-carboline bromide (****B41****)*: Yield, 74%; orange-yellow powders; ^1^H NMR (500 MHz, CD_3_Cl) *δ* 10.1 (1 H, s, H-1), 8.82 (1 H, d, *J* = 6.0 Hz, H-4), 8.80 (1 H, d, *J* = 6.0 Hz, H-3), 8.32 (1 H, d, *J* = 8.0 Hz, H-5), 8.13 (2 H, d, *J* = 7.6 Hz, H-2′, H-6′), 7.79 (1 H, t, *J* = 7.6 Hz), 7.61–7.55 (3 H, m), 7.50 (1 H, t, *J* = 7.2 Hz), 7.43 (1 H, t, *J* = 7.5 Hz), 6.09–6.03 (1 H, m, H-2″), 5.70 (2 H, br s, H-1″), 5.17 (1 H, d, *J* = 10.3 Hz, H-3″a), 5.06 (1 H, d, *J* = 17.1 Hz, H-3″b); ^13^C NMR (126 MHz, CD_3_Cl) *δ* 145.2 (C-1), 143.1 (C-1′), 135.8, 133.0, 132.04, 133.01, 131.3, 130.8, 130.5 (C-3′, C-5′), 128.4, 124.8 (C-2′, C-6′), 123.9, 122.6, 119.4, 118.4, 117.9 (C-3″), 111.2, 47.3 (C-1″); HR-ESI-MS [M-Br]^+^: Calcd for C_20_H_17_N_2_^+^, 285.1386 found 285.1383.

*9-Butyl-2-phenyl-β-carboline bromide (****B42****)*: Yield, 83%; orange-yellow powders; ^1^H NMR (500 MHz, CD_3_Cl) *δ* 10.1 (1 H, s, H-1), 8.81 (1 H, d, *J* = 5.9 Hz, H-4), 8.72 (1 H, d, *J* = 5.9 Hz, H-3), 8.30 (1 H, d, *J* = 7.8 Hz, H-5), 8.05 (2 H, d, *J* = 7.3 Hz, H-2′, H-6′), 7.77 (1 H, t, *J* = 7.3 Hz), 7.59–7.53 (3 H, m), 7.46 (1 H, t, *J* = 7.1 Hz), 7.38 (1 H, t, *J* = 7.1 Hz), 4.88 (2 H, t, *J* = 6.5 Hz, H-1″), 1.88–1.80 (2 H, m, H-2″), 1.34–1.31 (2 H, m, H-3″), 0.85 (3 H, t, *J* = 6.9 Hz, H-3″); ^13^C NMR (126 MHz, CD_3_Cl) *δ* 145.1 (C-1), 143.1 (C-1′), 135.92, 132.87, 132.0, 130.7, 130.5 (C-3′, C-5′), 127.8, 124.8 (C-2′, C-6′), 124.0, 122.4, 119.3, 118.4, 111.0, 57.9 (C-1″), 31.4 (C-2″), 20.3 (C-3″), 13.8 (C-4″); HR-ESI-MS [M-Br]^+^: Calcd for C_21_H_21_N_2_^+^, 301.1699 found 301.1696.

*9-Iso-butyl-2-phenyl-β-carboline bromide (****B43****)*: Yield, 80%; orange-yellow powders; ^1^H NMR (500 MHz, CD_3_Cl) *δ* 10.1 (1 H, s, H-1), 8.84 (1 H, d, *J* = 5.7 Hz, H-4), 8.76 (1 H, d, *J* = 5.7 Hz, H-3), 8.30 (1 H, d, *J* = 7.8 Hz, H-5), 8.13 (2 H, d, *J* = 7.3 Hz, H-2′, H-6′), 7.75 (1 H, t, *J* = 7.3 Hz), 7.57 (1 H, d, *J* = 8.3 Hz), 7.52 (2 H, *J* = 7.1 Hz, H-3′, H-5′), 7.43 (1 H, t, *J* = 7.1 Hz), 7.37 (1 H, t, *J* = 7.1 Hz), 4.74 (2 H, d, *J* = 7.1 Hz, H-1″), 2.28–2.36 (1 H, m, H-2″), 0.93 (6 H, d, *J* = 6.0 Hz, 2 × CH_3_); ^13^C NMR (126 MHz, CD_3_Cl) *δ* 145.5 (C-1), 143.1 (C-1′), 136.3, 132.9, 132.8, 132.2, 130.7, 130.4 (C-3′, C-5′), 128.1, 124.9 (C-2′, C-6′), 123.9, 122.4, 119.2, 118.5, 111.3, 51.9 (C-1″), 29.2 (C-2″), 20.2 (2 × CH_3_); HR-ESI-MS [M-Br]^+^: Calcd for C_21_H_21_N_2_^+^, 301.1699 found 301.1697.

*9-Benzyl-2-phenyl-β-carboline bromide (****B44****)*: Yield, 76%; orange-yellow powders; ^1^H NMR (500 MHz, CD_3_Cl) *δ* 10.2 (1 H, s, H-1), 8.69 (1 H, d, *J* = 6.3 Hz, H-3), 8.55 (1 H, d, *J* = 6.3 Hz, H-4), 8.37 (1 H, dd, *J* = 8.1 Hz), 8.16 (1 H, d, *J* = 7.7 Hz), 7.87 (1 H, d, *J* = 7.8 Hz), 7.74 (1 H, *J* = 8.6 Hz), 7.66 (2 H, t, *J* = 7.6 Hz), 7.60 (1 H, t, *J* = 7.4 Hz), 7.53 (1 H, t, *J* = 7.7 Hz), 7.34 (3 H, d-like, *J* = 7.1 Hz), 7.28 (3 H, d-like, *J* = 7.2 Hz), 6.43 (2 H, s, NCH_2_); ^13^C NMR (126 MHz, CD_3_Cl) *δ* 145.7 (C-1), 143.3 (C-1′), 136.0, 135.6, 133.3, 133.1, 131.4, 131.0, 130.6, 129.5, 129.0, 128.1, 127.0, 124.8, 123.7, 122.8, 119.4, 117.7, 111.4, 48.3 (NCH_2_); HR-ESI-MS [M-Br]^+^: Calcd for C_24_H_19_N_2_^+^, 335.1544 found 335.1543.

*9-(Ethoxy-2-oxyethyl)-2-phenyl-β-carboline bromide (****B45****)*: Yield, 75%; orange-yellow powders; ^1^H NMR (500 MHz, CD_3_Cl) *δ* 10.5 (1 H, s, H-1), 8.59 (1 H, d, *J* = 6.1 Hz, H-3), 8.48 (1 H, d, *J* = 6.3 Hz, H-4), 8.31 (1 H, d, *J* = 7.8 Hz), 8.16 (2 H, d, *J* = 7.4 Hz), 7.86 (1 H, t, *J* = 7.6 Hz), 7.66 (2 H, t-like, *J* = 7.3 Hz), 7.59 (2 H, t-like, *J* = 8.8 Hz), 7.50 (1 H, t, *J* = 7.6 Hz), 6.17 (2 H, s, N-CH_2_), 4.28 (2 H, q, *J* = 7.1 Hz, CH_2_CH_3_), 1.36 (3 H, t, *J* = 7.1 Hz, CH_3_); ^13^C NMR (126 MHz, CDCl_3_) *δ* 168.2 (C = O), 145.2 (C-1), 143.2 (C-1′), 136.7, 133.22, 133.17, 131.2, 131.0, 130.6, 130.5, 124.7, 123.6, 122.9, 119.6, 117.5, 110.7, 62.4 (OCH_2_), 47.0 (NCH_2_), 14.1 (CH_3_); HR-ESI-MS [M-Br]^+^: Calcd for C_21_H_19_N_2_O_2_^+^, 331.1450 found 331.1441.

*2-Phenyl-β-carboline bromide (****B46****)*: Yield 72%; yellow powders; mp 228.1–229.8 °C; The ^1^H NMR, ^13^C NMR and MS data are consistent with those in the literature^33^.

#### Synthesis of 9-methyl-2-p-toly-1,2,3,4-tetrahydro-β-carboline (C1)

To the solution of compound **A22** (6.1 mmol) in 50 mL ethanol was slowly added NaBH_4_ (0.347 g, 9.2 mmol) under stirring. After completion of the reaction by TLC, the solution was concentrated under reduced pressure. 100 mL water was added to the residue, extracted with EtOAc (2 × 60 mL), washed with brine and dried over anhydrous Na_2_SO_4_. After filtration, the solvent was removed under reduced pressure to yield compound **C1** as white powders in 98% yield. Mp 118.4–118.7 °C; ^1^H NMR (125 MHz, D_3_COD) *δ* 7.52 (1 H, d, *J* = 7.8 Hz, H-8), 7.30 (1 H, d, *J* = 8.2 Hz, H-5), 7.20 (1 H, 2 × t, *J* = 8.1, 1.0 Hz, H-6), 7.11 (2 H, d, *J* = 8.5 Hz, H-3′, H-5′), 7.10 (1 H, 2 × t, *J* = 7.8, 0.9 Hz, H-7), 7.00 (2 H, d, *J* = 8.5 Hz, H-2′, H-6′), 4.38 (2 H, s, H-1), 3.69 (3 H, s, NCH_3_), 3.60 (2 H, t, *J* = 5.6 Hz, H-3), 2.93 (2 H, t, *J* = 5.6 Hz, H-4), 2.30 (3 H, s, CH_3_); ^13^C NMR (500 MHz, D_3_COD) *δ* 149.1 (C-1′), 137.3 (C-8a), 133.2 (C-9a), 129.9 (C-4′), 129.3 (C-3′, C-5′), 126.8 (C-4b), 121.1 (C-7), 119.1 (C-6), 118.2 (C-5), 116.9 (C-8), 108.8 (C-2′, C-4′), 108.1 (C-4a), 48.7 (C-3), 46.6 (C-1), 29.4 (NCH_3_), 21.8 (C-4), 20.6 (CH_3_); HR-ESI-MS [M−H]^+^ calcd for C_19_H_19_N_2_, 275.1543, found 275.1536.

### Assay of AChE and BuChE

The inhibitory activities of the compounds against AChE were evaluated according to Ellman’s coupled enzyme assay^47^ with the following modifications. 90 μL potassium phosphate buffer (PBS, 0.1 M, pH 7.4), 10 μL the solution of 2 units/mL AChE in 0.1 M PBS (pH 7.4) and 10 μL the solution of 200 μM test compounds in the mixed solution of methanol-PBS (pH 7.4) (1:9, V/V) were added to each well of a 96-well plate. The methanol-PBS solution was used as a blank control. After incubation at room temperature for 10 min, 60 μL the solution of 10 mM DTNB in PBS was added into each the well. The plate was placed on crushed ices and cooled for 20 min. To each the well was added 30 μL the solution of 7.5 mM ATCh in PBS (pH 7.4). After incubation at 37 °C for 40 min, the initial rate of the enzyme was analyzed by measuring absorbance at wavelength of 412 nm with a microplate reader (Molecular Devices Co., Ltd.). Each test was performed in triplicate. Inhibition rates of the enzyme were calculated relative to a control sample. The inhibitory activities against BuChE were measured as described above for AChE, using 0.7 unit/mL BuChE and 4 mM BuTCh instead of AChE and ATCh as the enzyme and substrate, respectively.

The compounds with higher initial activities were further assayed for IC_50_ values according to the method described above. Based on the screening results, a series of test concentrations of the compound were set and tested for inhibition rate against AChE and BChE. The probit value of the average inhibition rate for each test concentration and the corresponding lg[concentration] were used to establish enzyme inhibition regression equation by the linear least-square fitting method. IC_50_ values were calculated from the equations.

Duncan multiple comparison test was used to evaluate significant difference between the average inhibition rates of various compounds at the same concentration by using PRISM software ver. 5.0 (GraphPad Software Inc., San Diego, CA, USA).

### Data

Data will be made available upon request to the corresponding authors.

## Electronic supplementary material

Supplementary Information
