# Supplementary material for: New 2-Aryl-9-methyl-β-carbolinium salts as Potential Acetylcholinesterase Inhibitor agents: Synthesis, Bioactivity and Structure–Activity Relationship
Source: Sci Rep. 2018 Jan 24;8:1559. doi: 10.1038/s41598-018-19999-3 (PMC5784158; doi:10.1038/s41598-018-19999-3)
Supplement: Supplementary file 1 — Supplementary Information [file 41598_2018_19999_MOESM1_ESM.pdf]

Supporting information for:

New 2-Aryl-9-methyl-*β*-carbolinium salts as Potential Acetylcholinesterase Inhibitor agents: Synthesis, Bioactivity and Structure–Activity Relationship

Bohang Zhou[†,§], Bingyu Zhang[†,§], Xingqiang Li[†], Xiuxiu Liu[†], Hui Li[†], Ding Li[†], Zhiming Cui[†], Huiling Geng[*,†] and Le Zhou[*,†]

[†]College of Chemistry & Pharmacy, Northwest A&F University, Yangling 712100, Shaanxi Province, People's Republic of China

[*]Corresponding author (Tel: +86-29-87092226; Fax: +86-29-87092226: E-mail: zhoulechem@nwsuaf.edu.cn (L. Zhou); genghuiling5@163.com (H.-L. Geng))

[§]These authors contributed equally to this work.

Contents

^{1}H and ^{13}C NMR spectra of new compounds **B2-B45, C1**......3

HRMS spectra of new compounds **B2-B45, C1**......48

Negative MS spectra of new compounds **B7, B13, B19 and B22**......70

Molecular docking studies......73

In vitro cytotoxicity assay......74

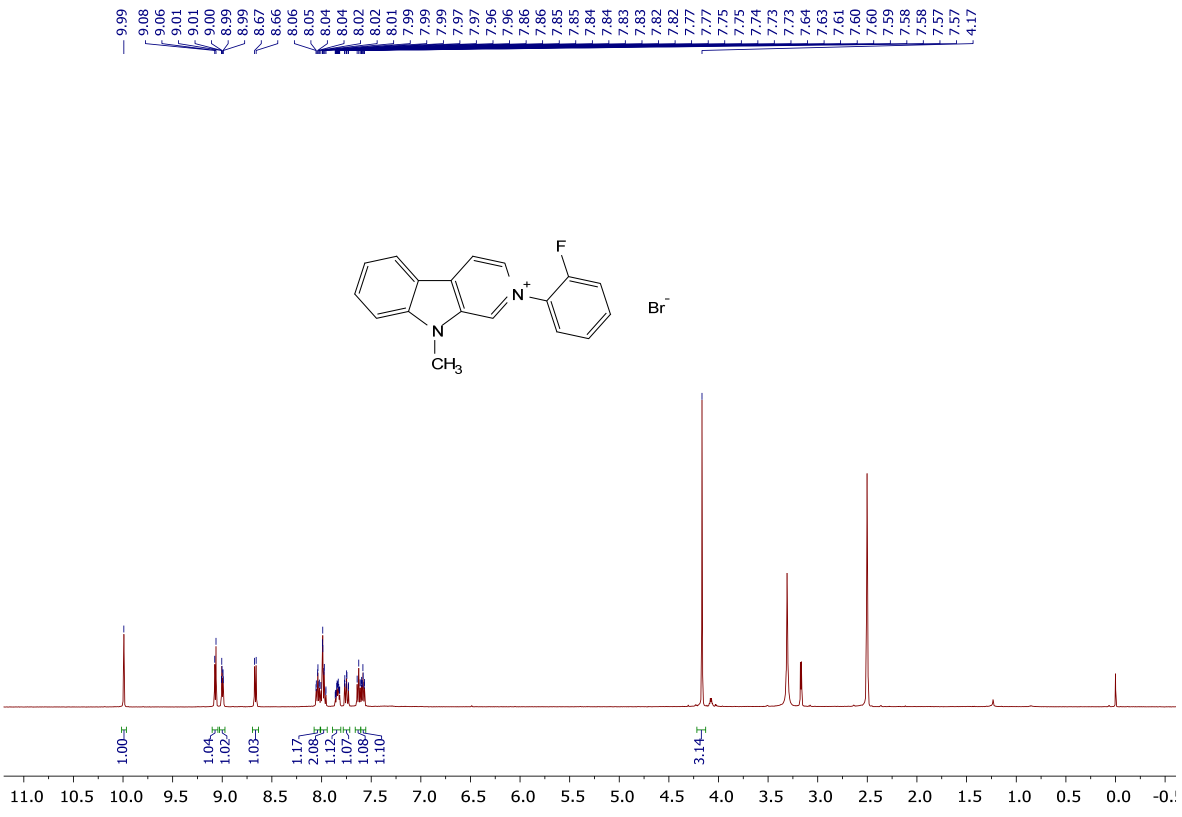

^{1}H NMR of Compound B**2**

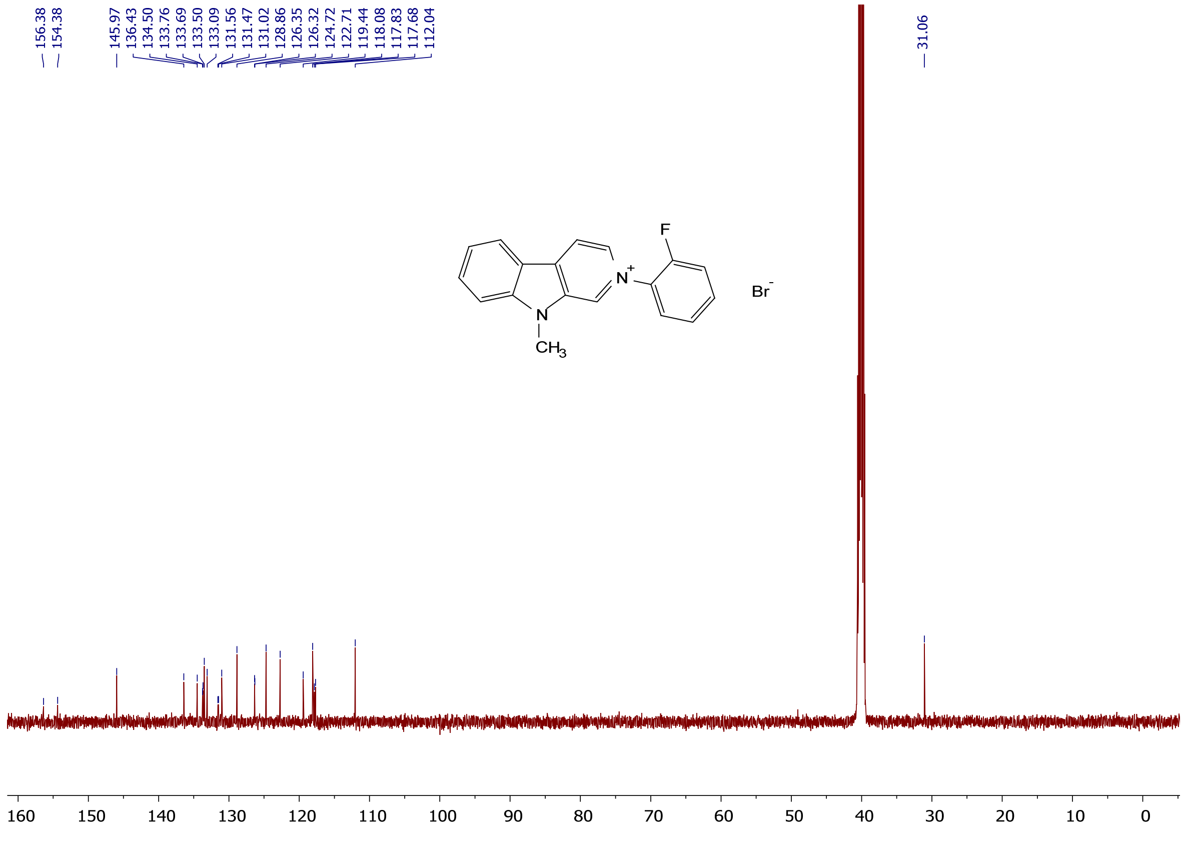

^{13}C NMR of Compound B**2**

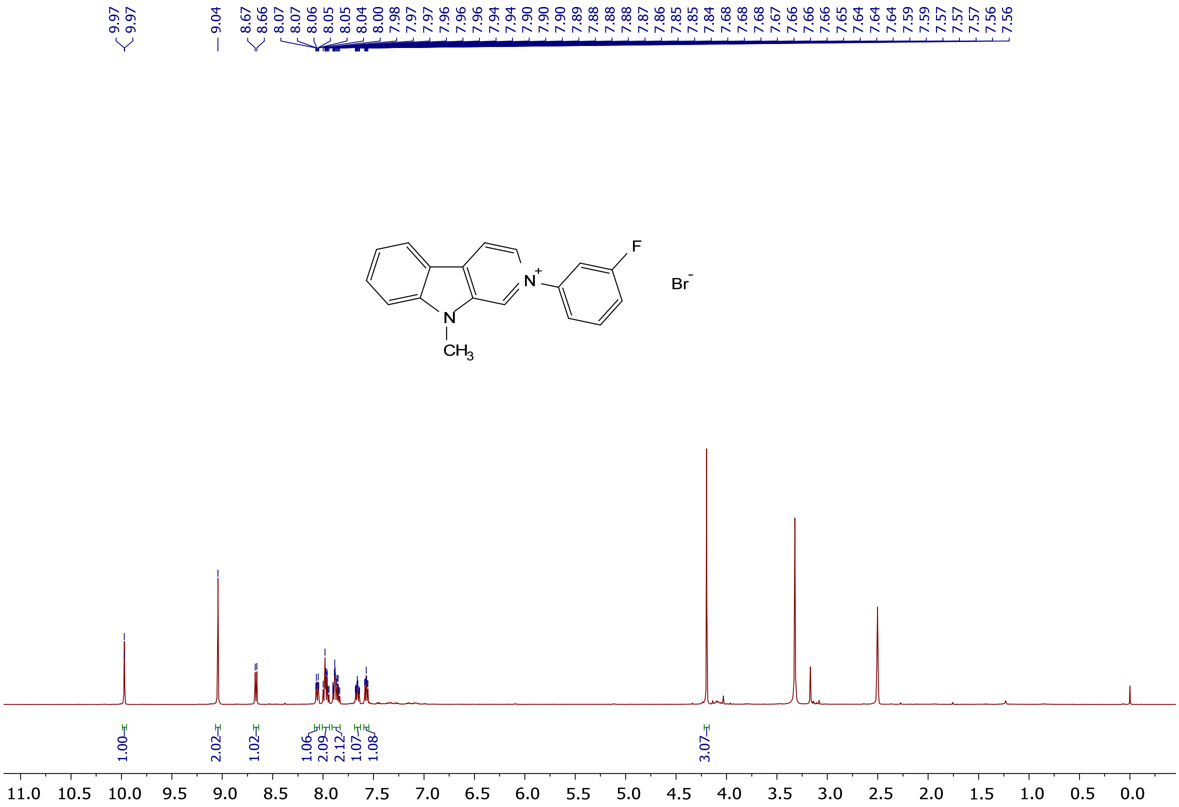

^{1}H NMR of Compound B**3**

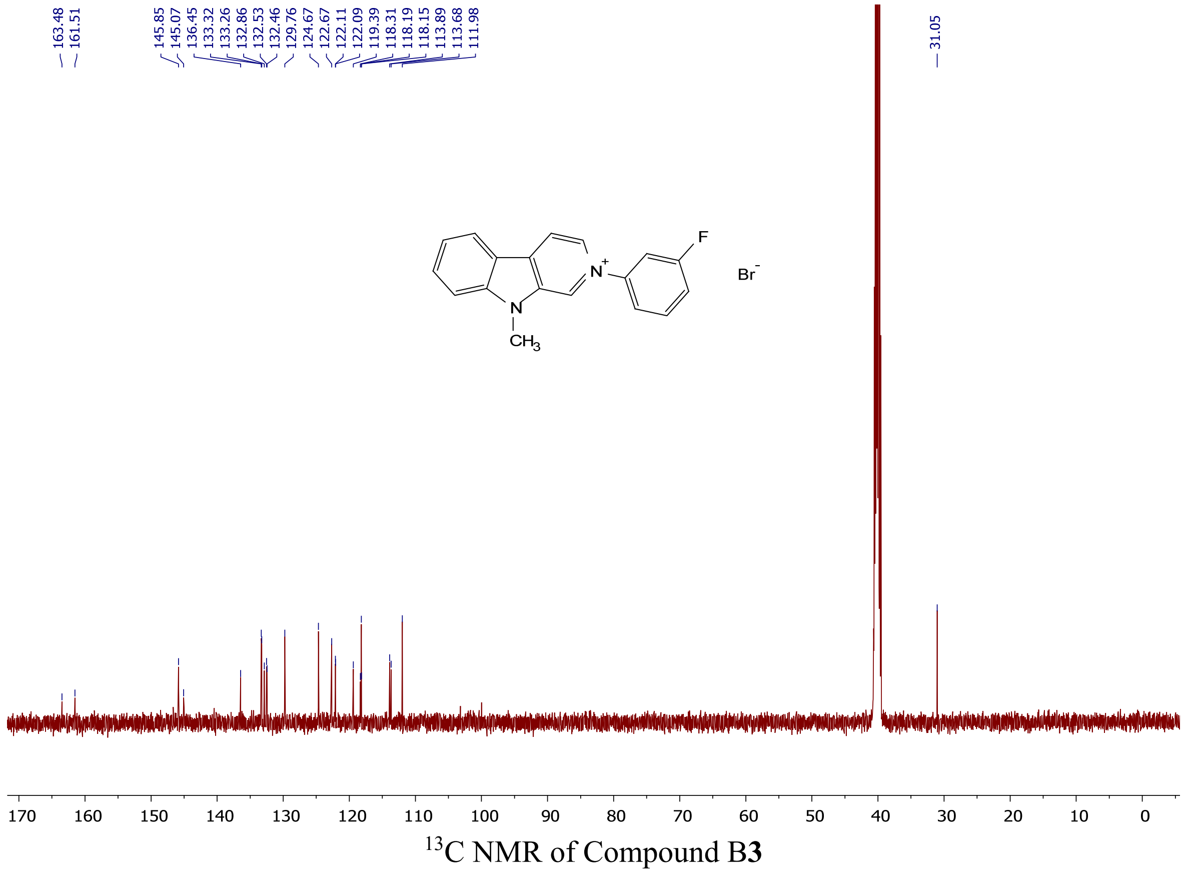

^{13}C NMR of Compound B**3**

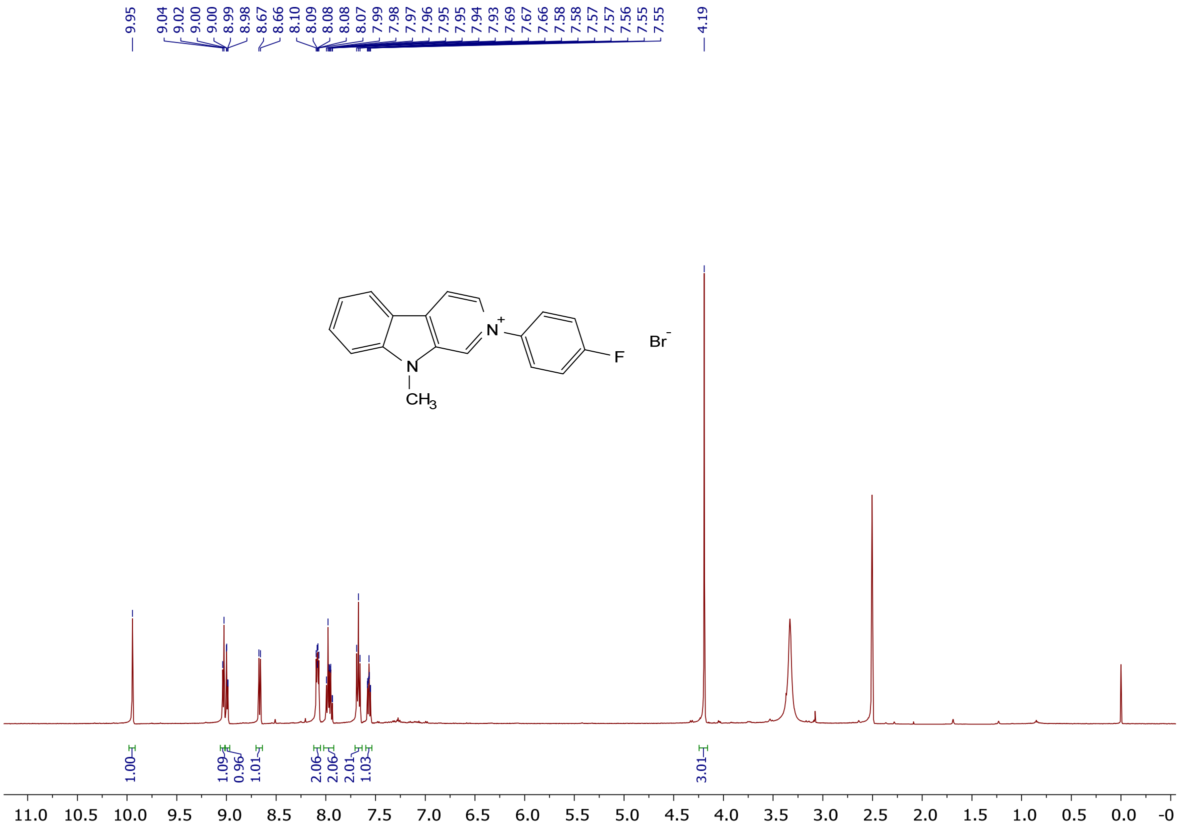

^{1}H NMR of Compound B**4**

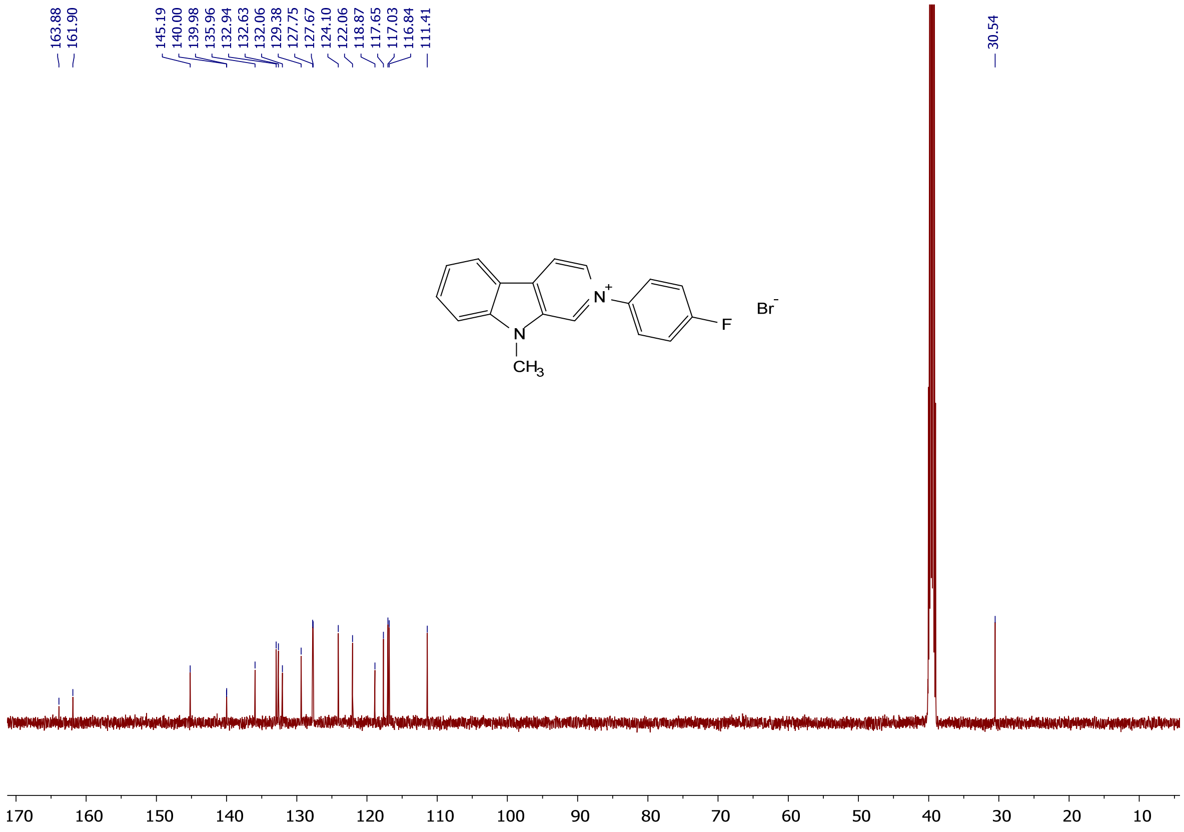

^{13}C NMR of Compound B**4**

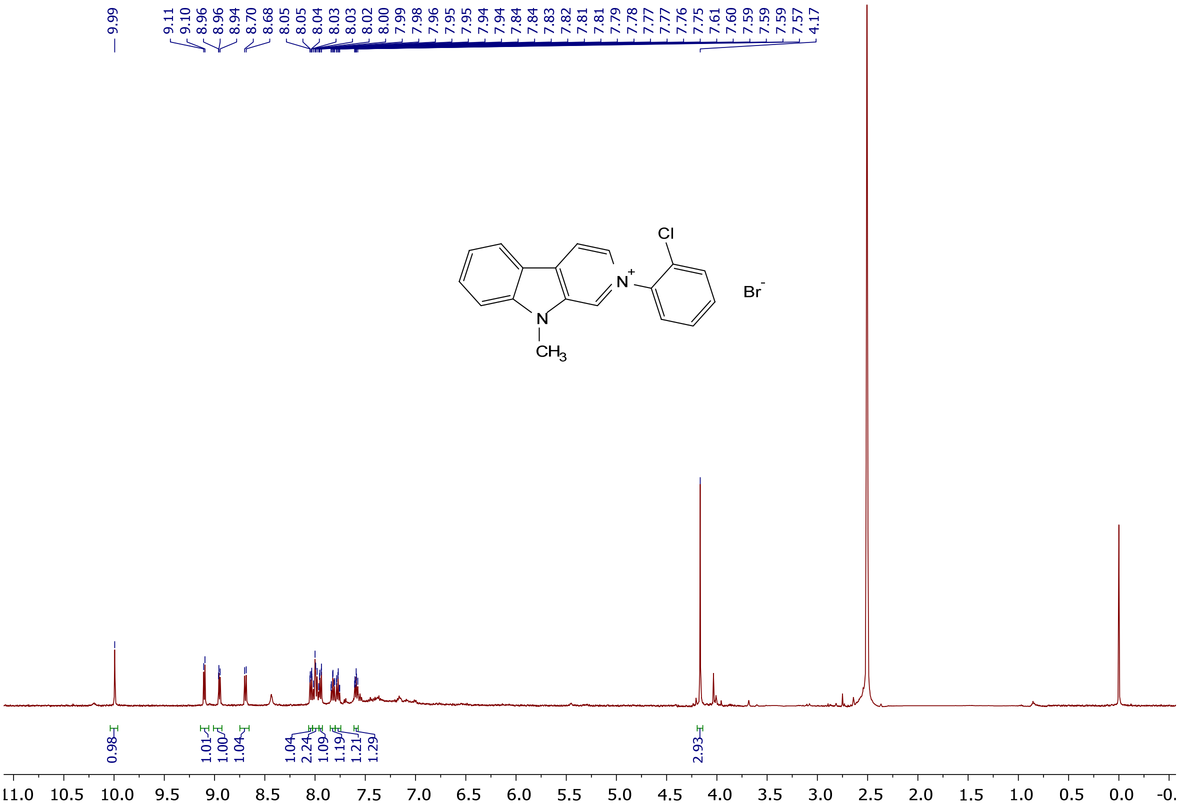

^{1}H NMR of Compound B**5**

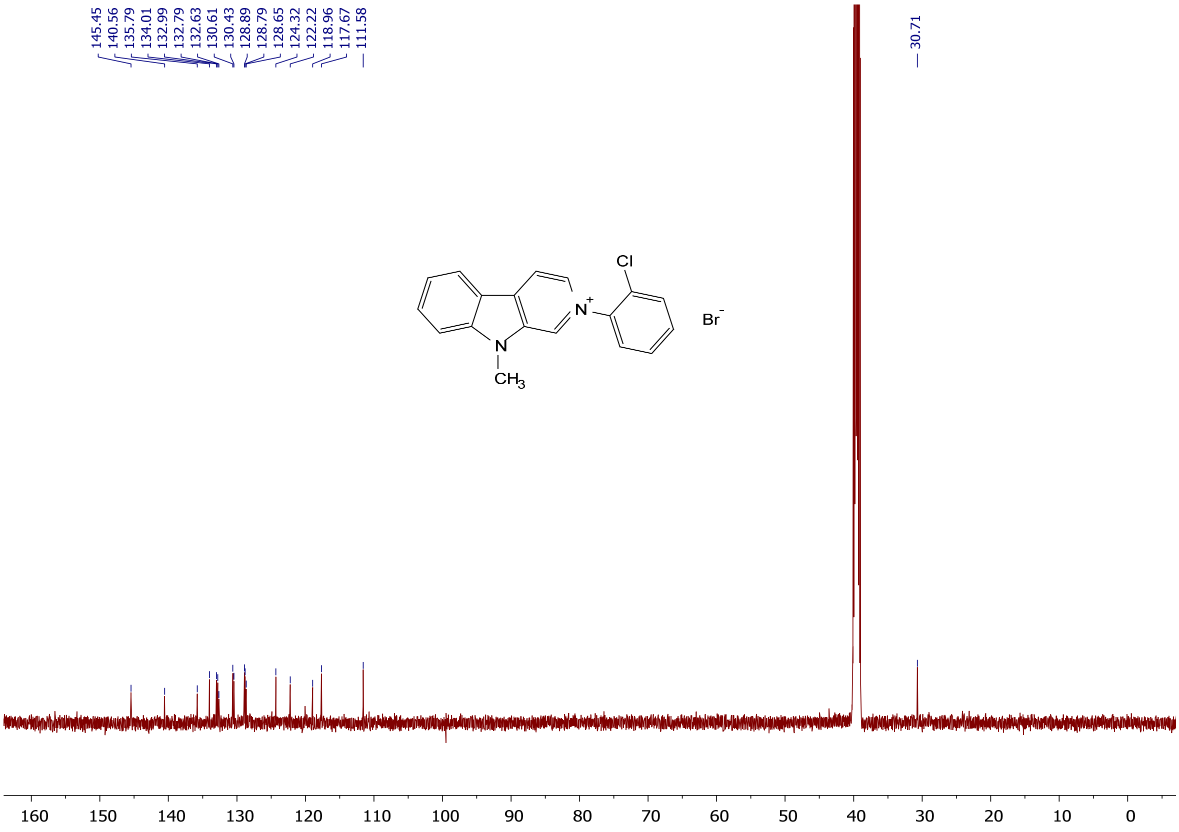

^{13}C NMR of Compound B**5**

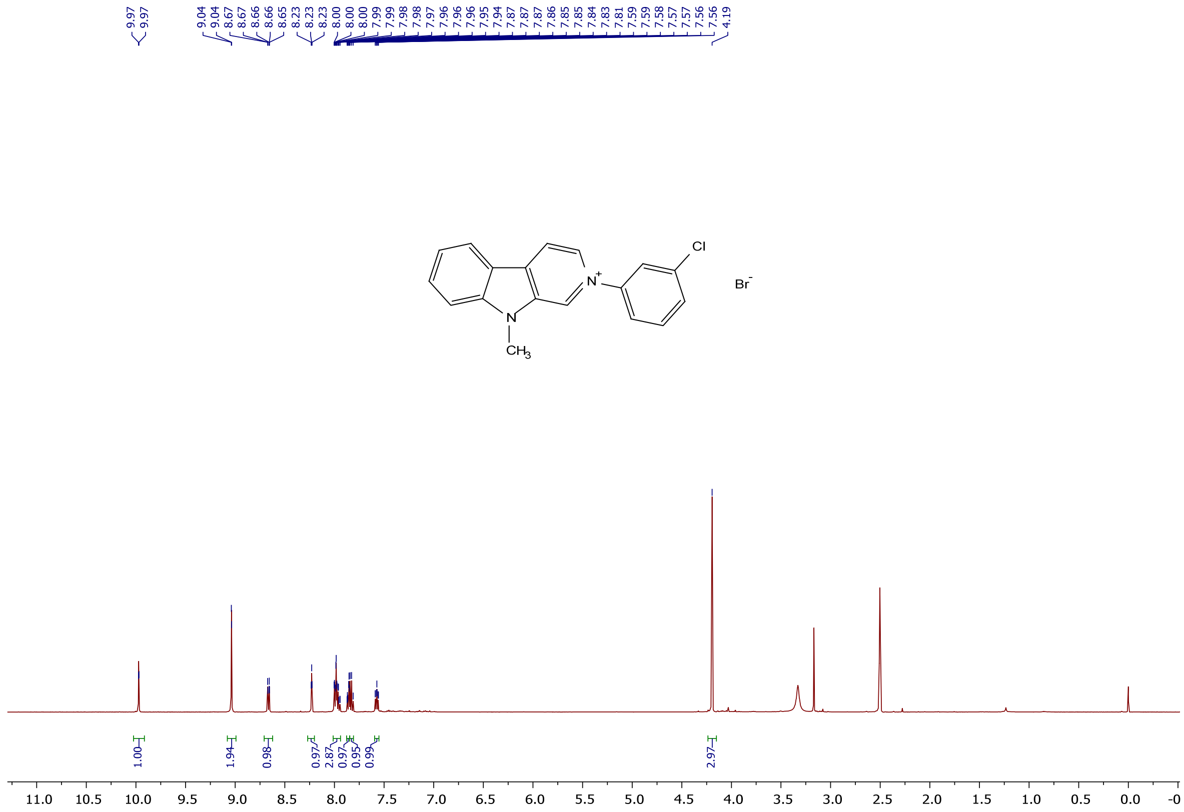

^{1}H NMR of Compound **B6**

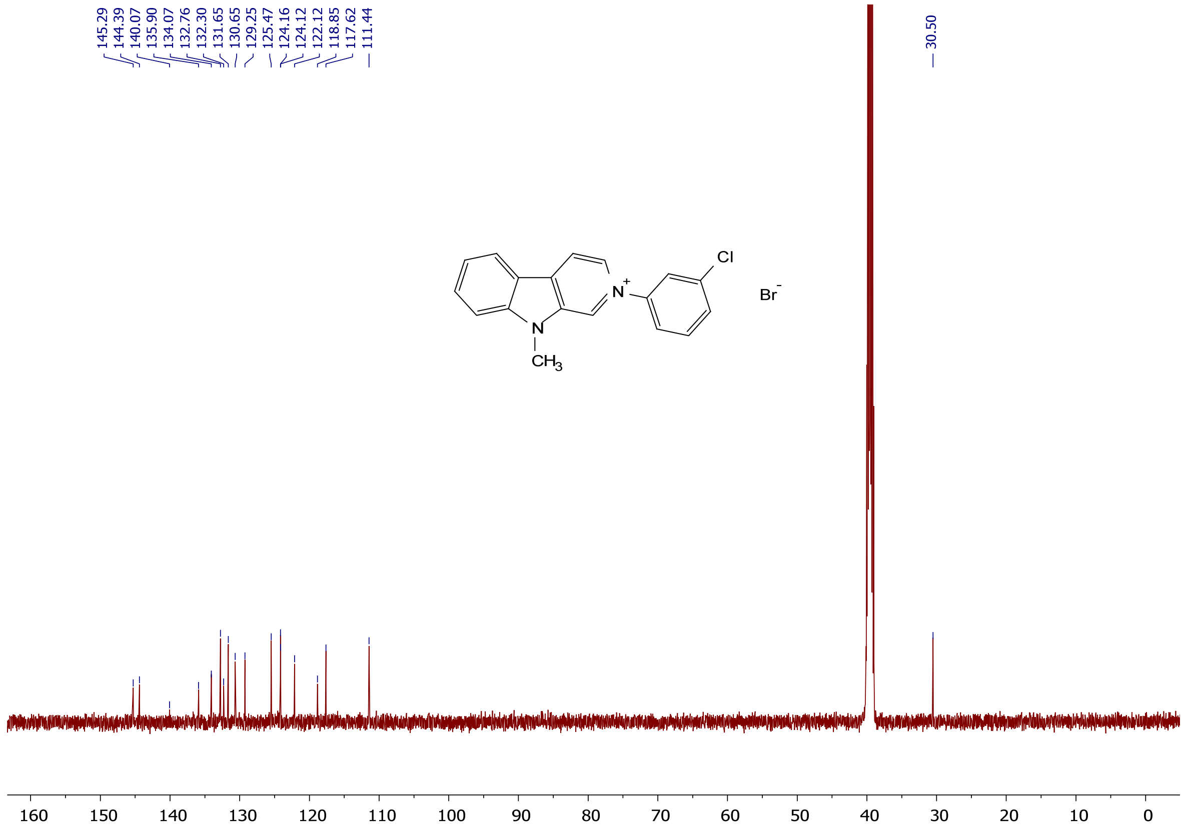

^{13}C NMR of Compound **B6**

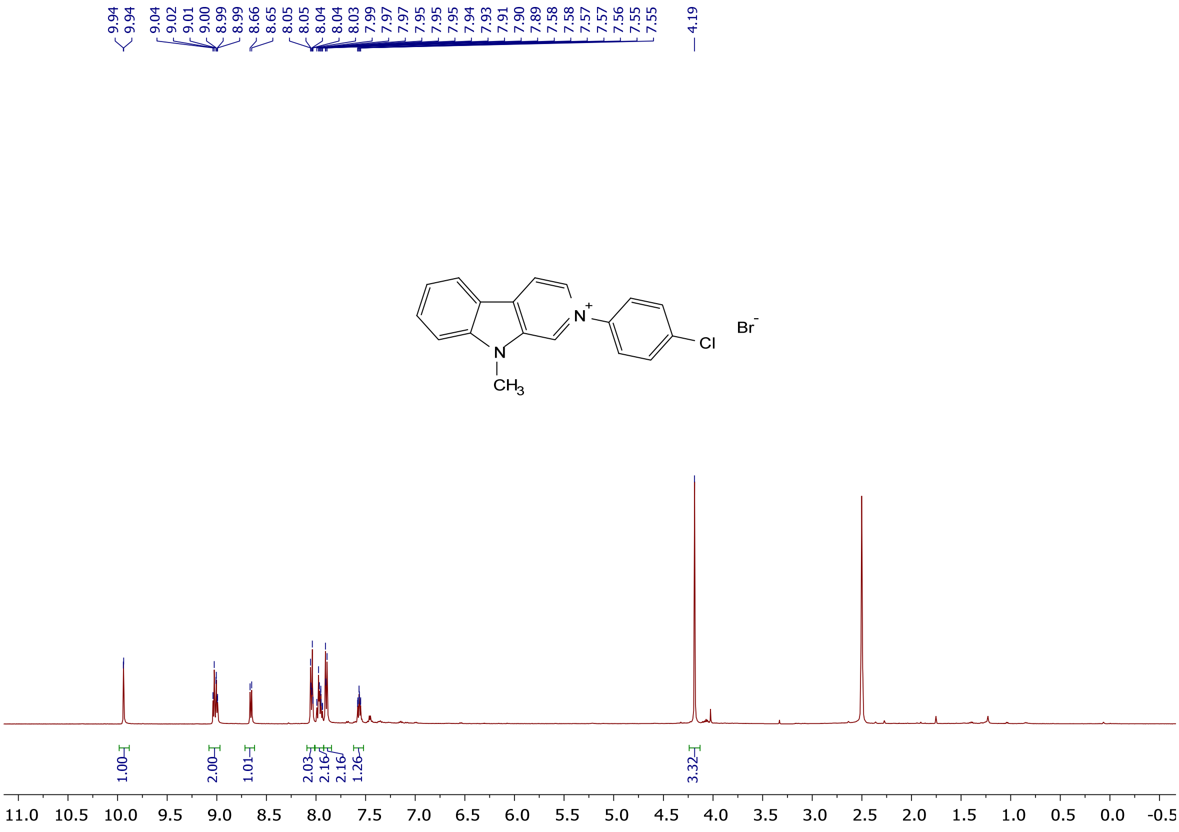

^{1}H NMR of Compound B**7**

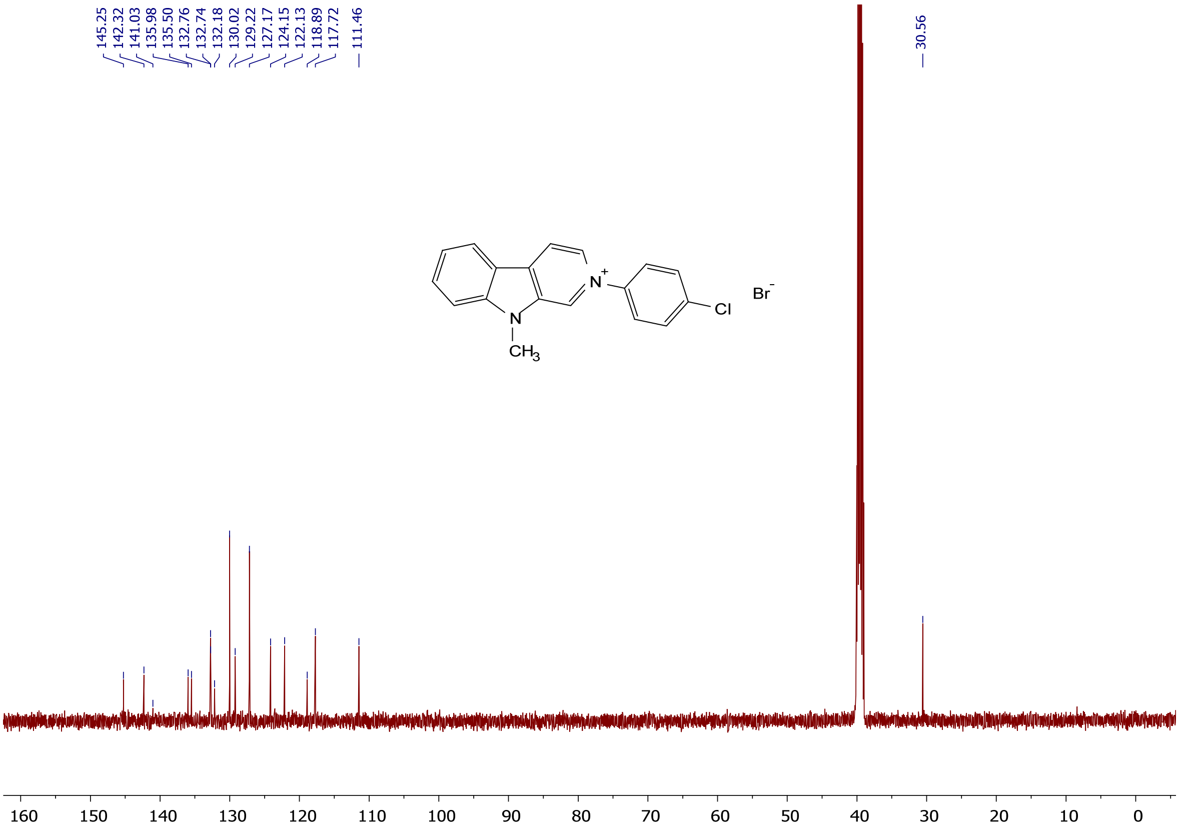

^{13}C NMR of Compound B**7**

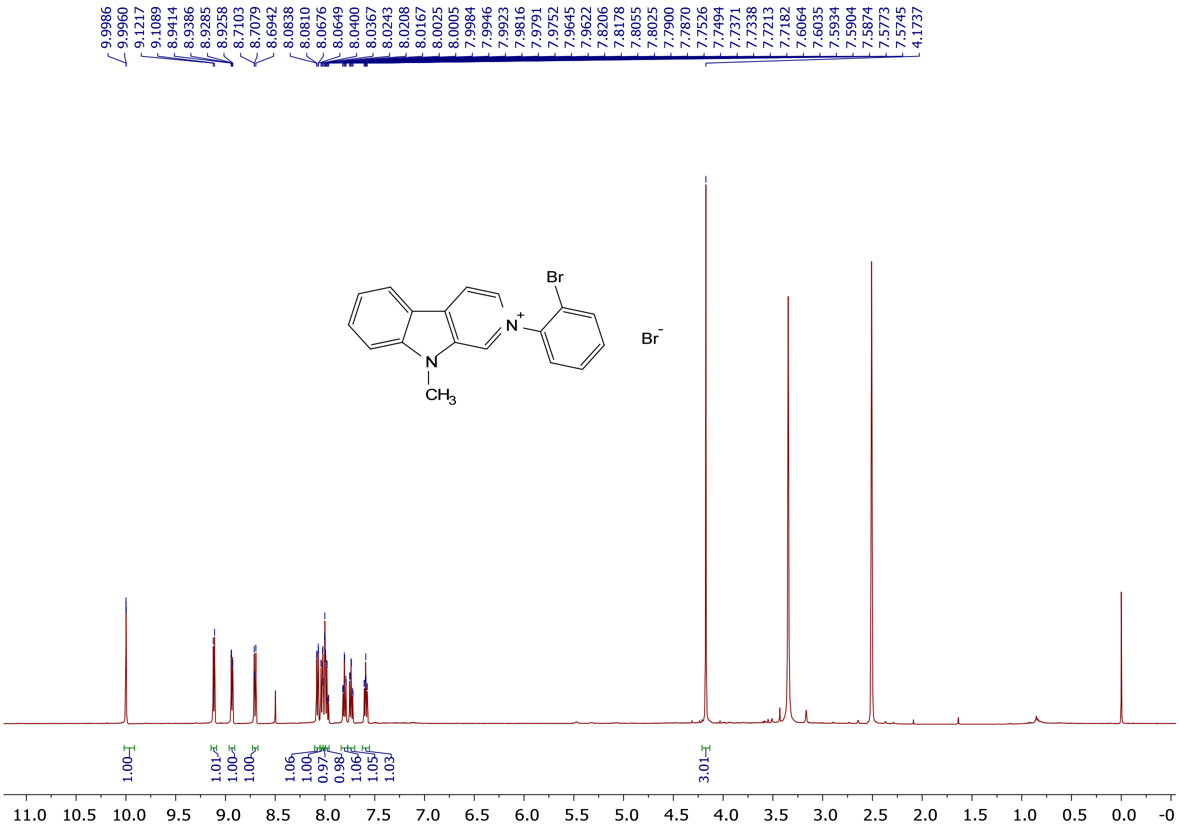

^{1}H NMR of Compound B**8**

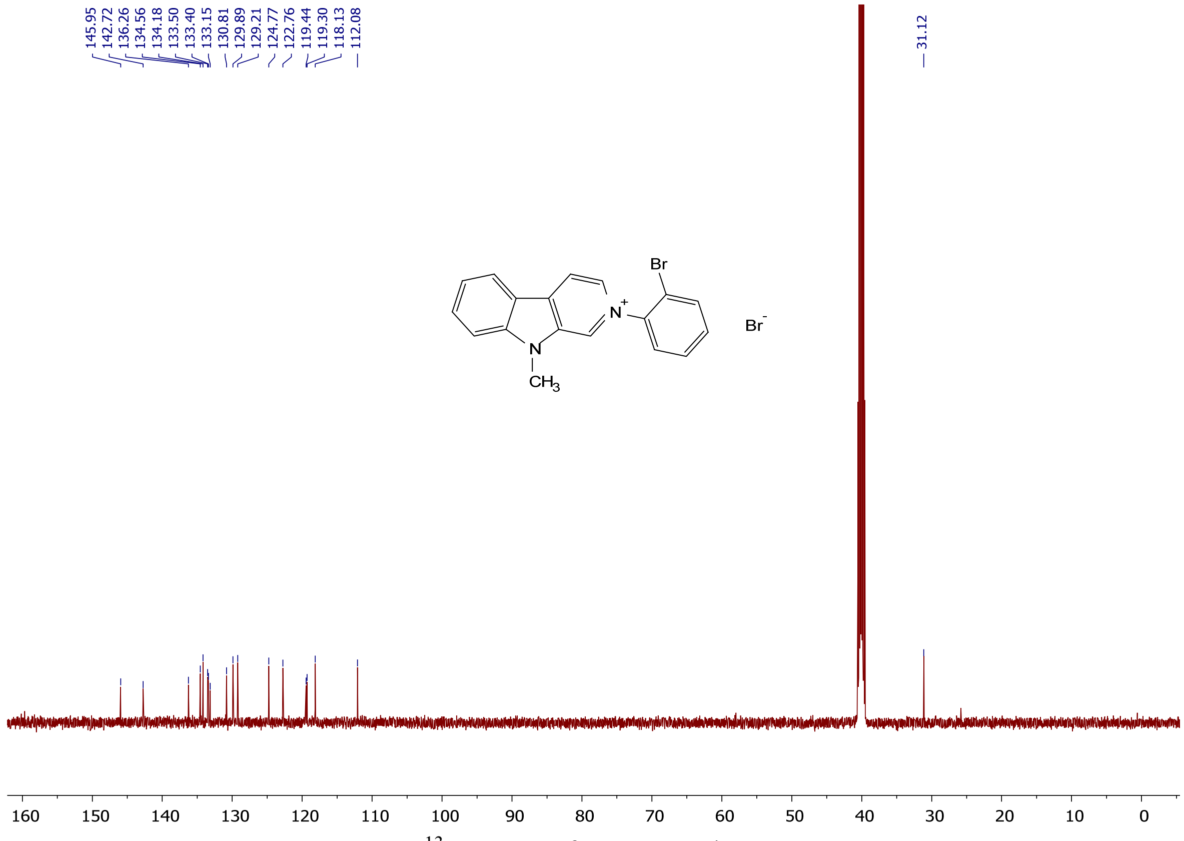

^{13}C NMR of Compound B**8**

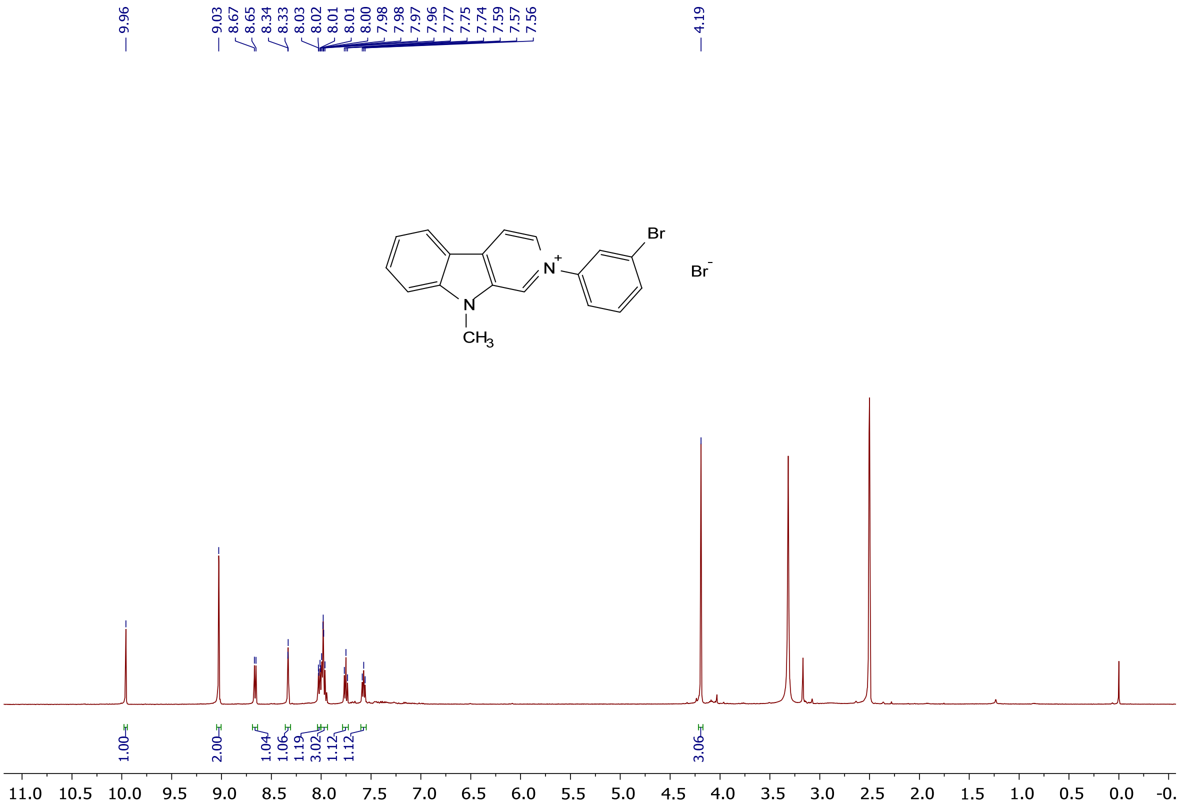

^{1}H NMR of Compound B**9**

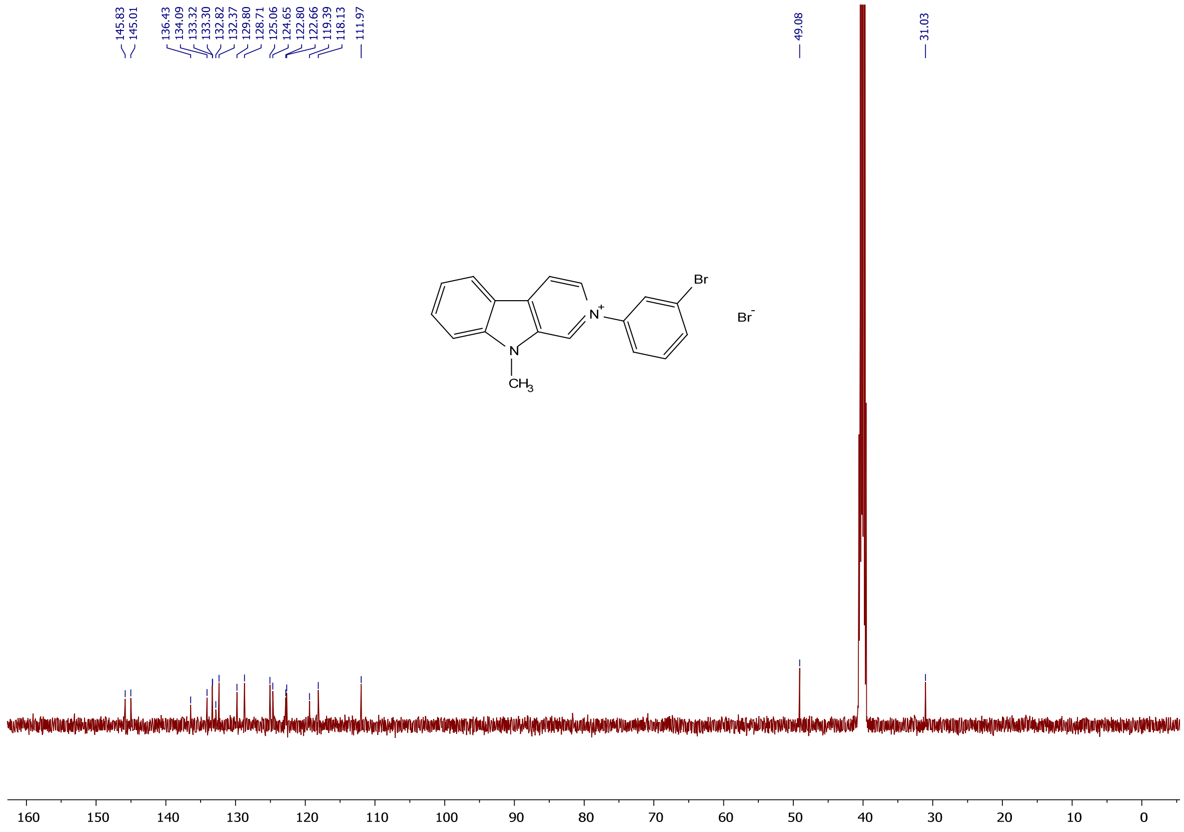

^{13}C NMR of Compound B**9**

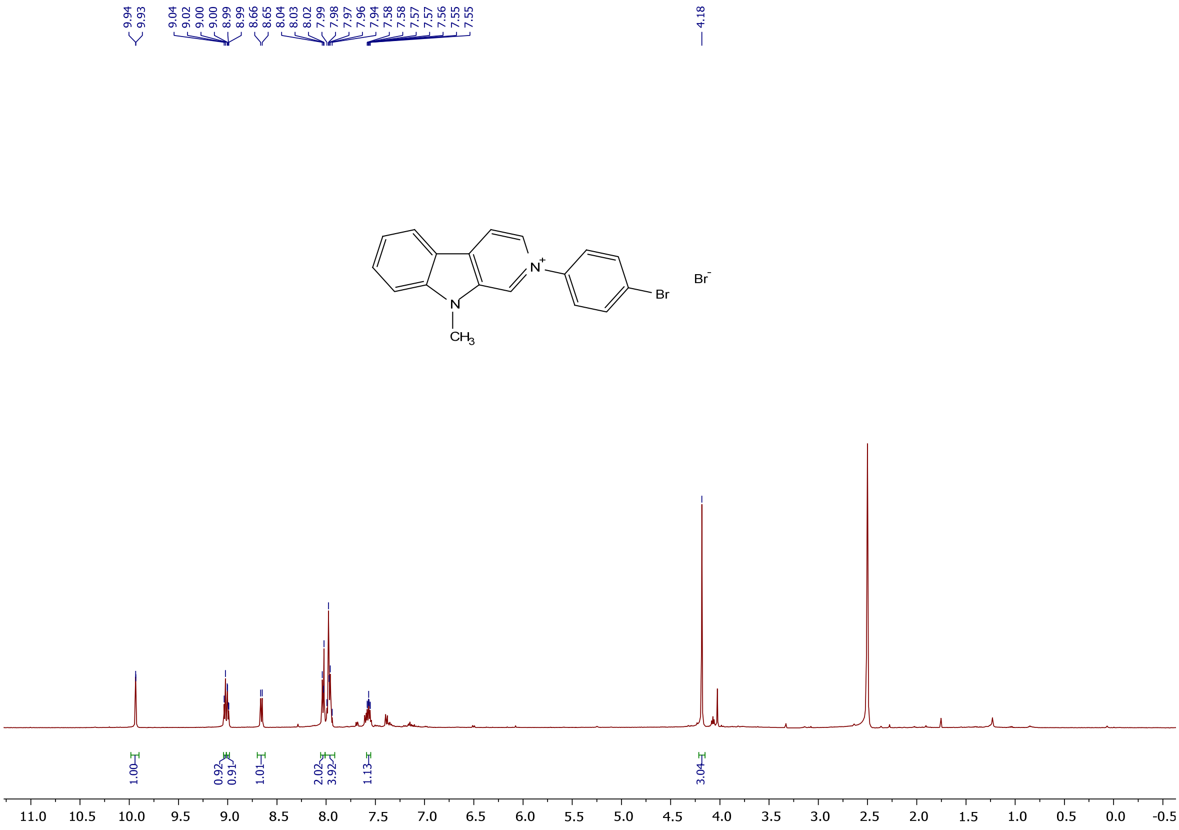

^{1}H NMR of Compound B**10**

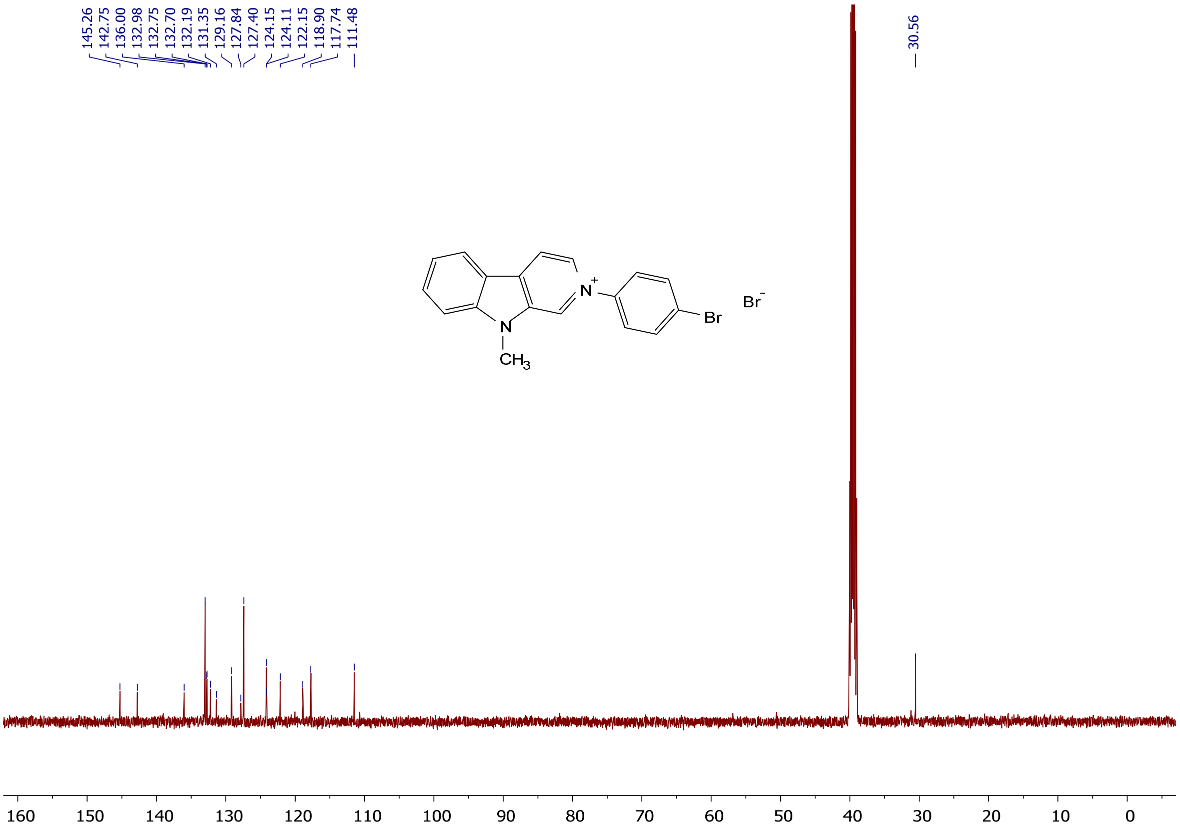

^{13}C NMR of Compound B**10**

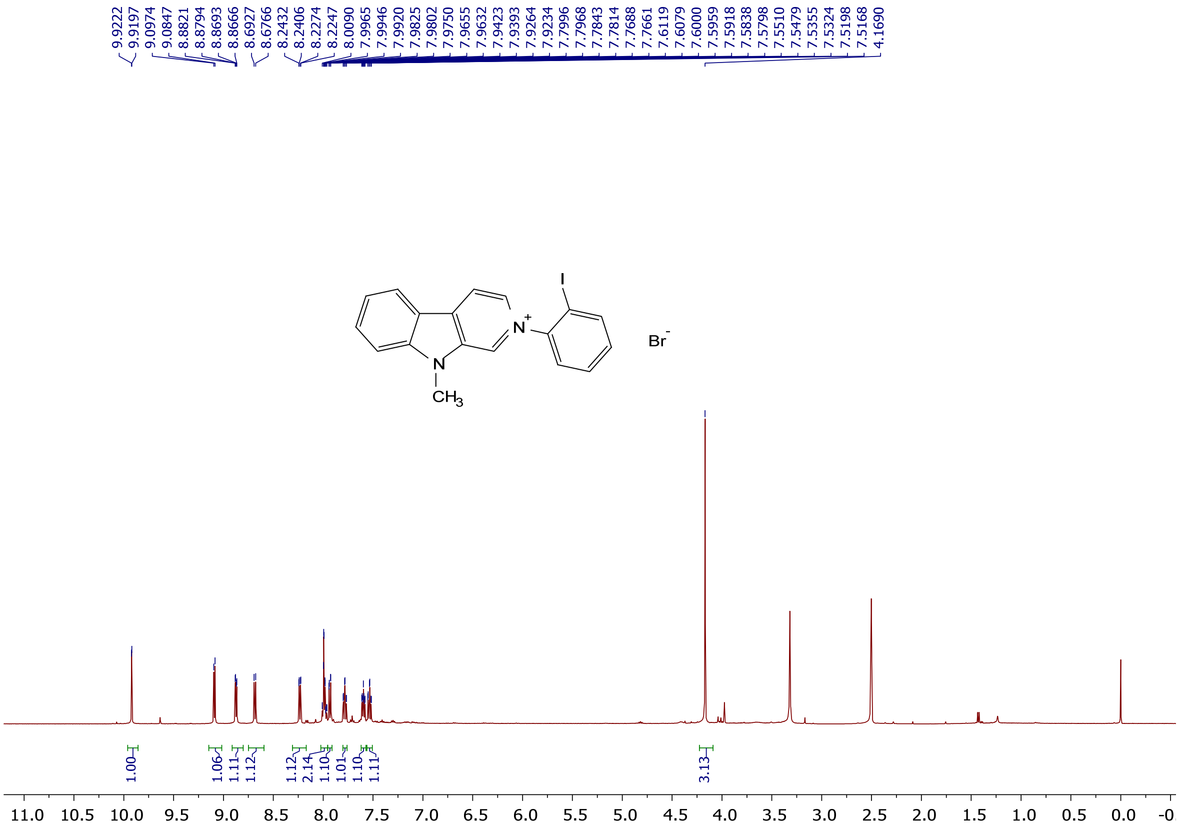

^{1}H NMR of Compound B**11**

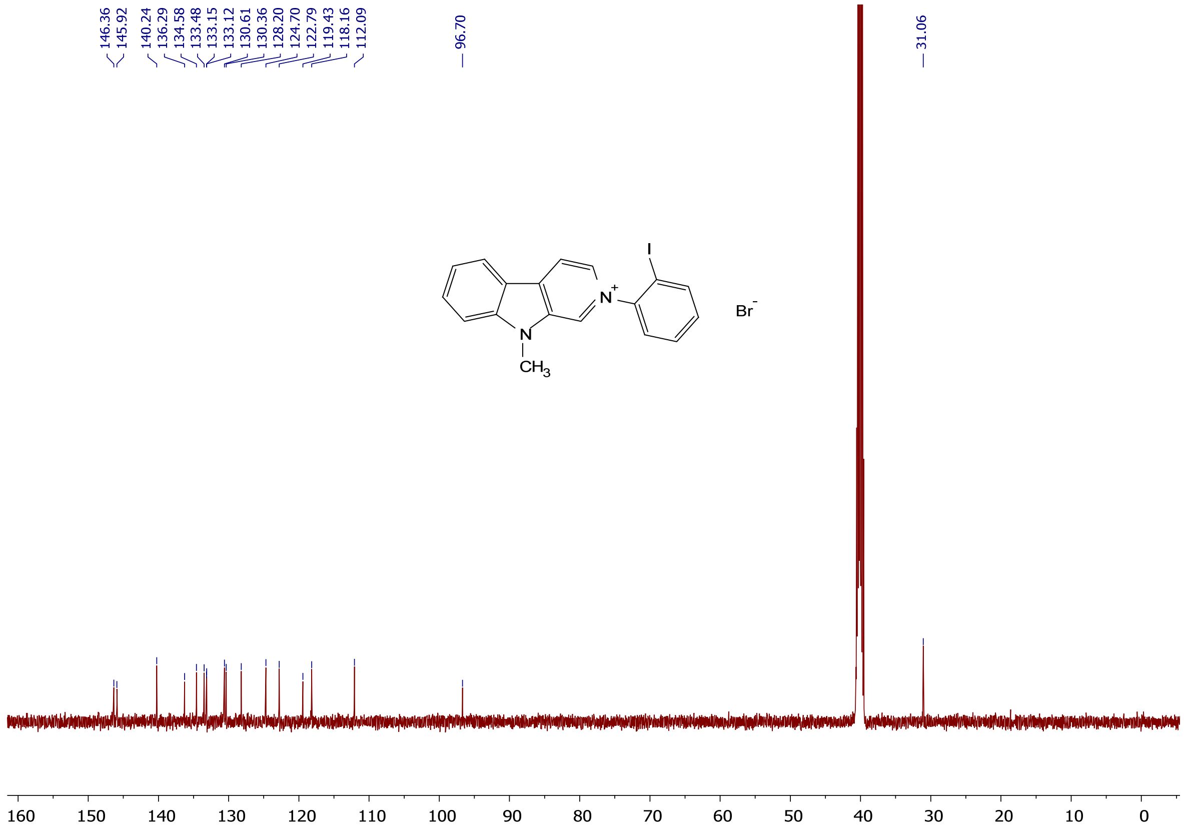

^{13}C NMR of Compound B**11**

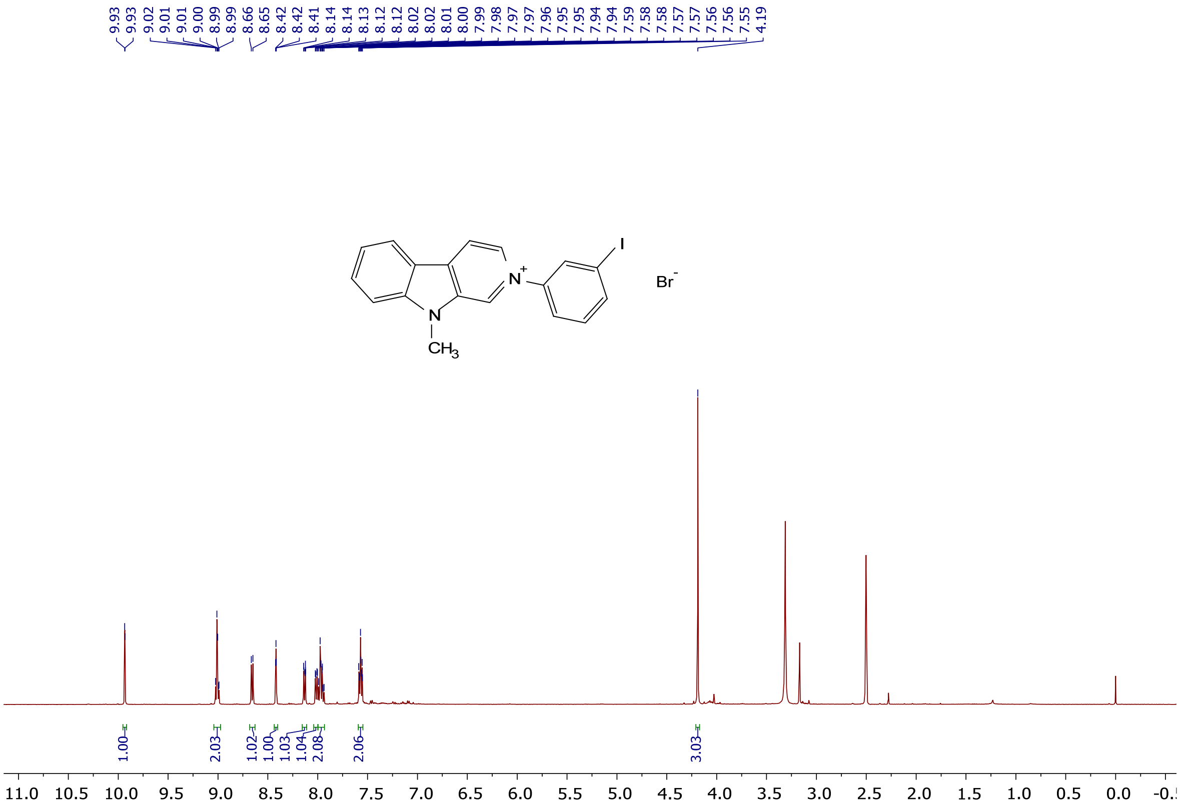

^{1}H NMR of Compound B**12**

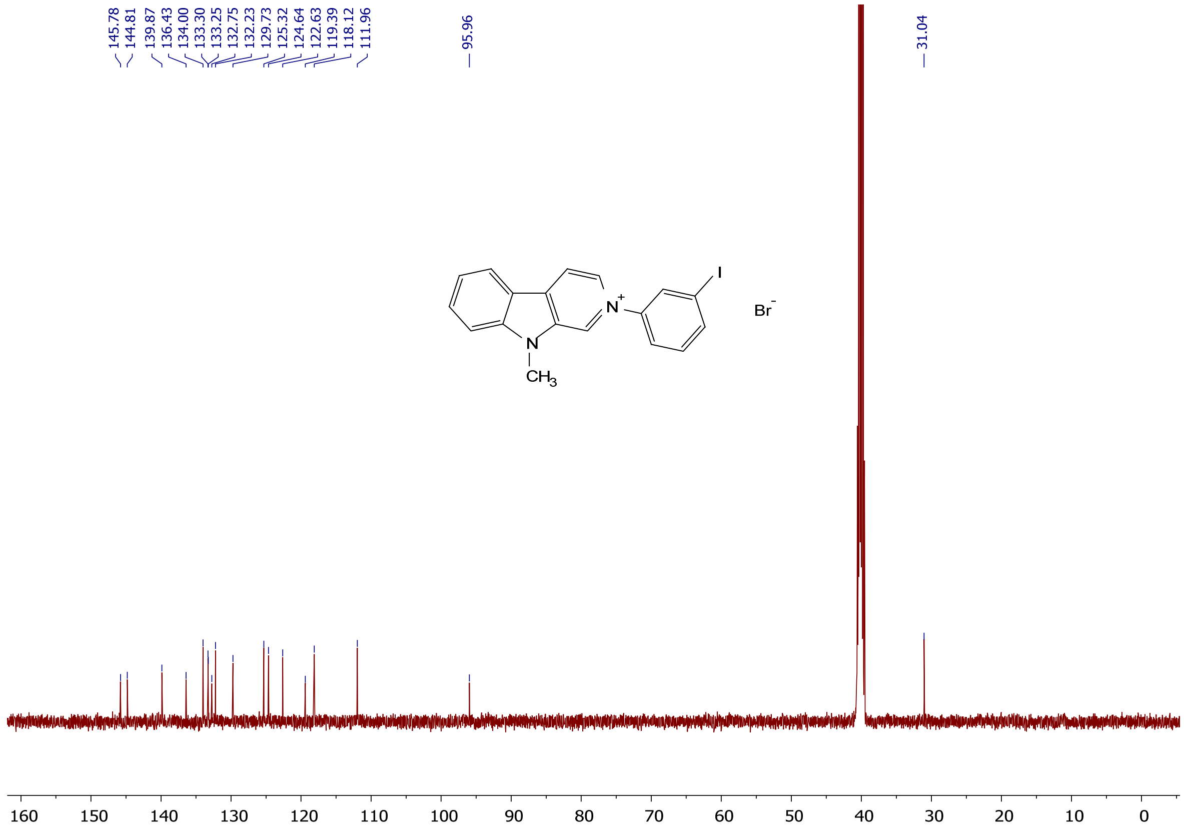

^{13}C NMR of Compound B**12**

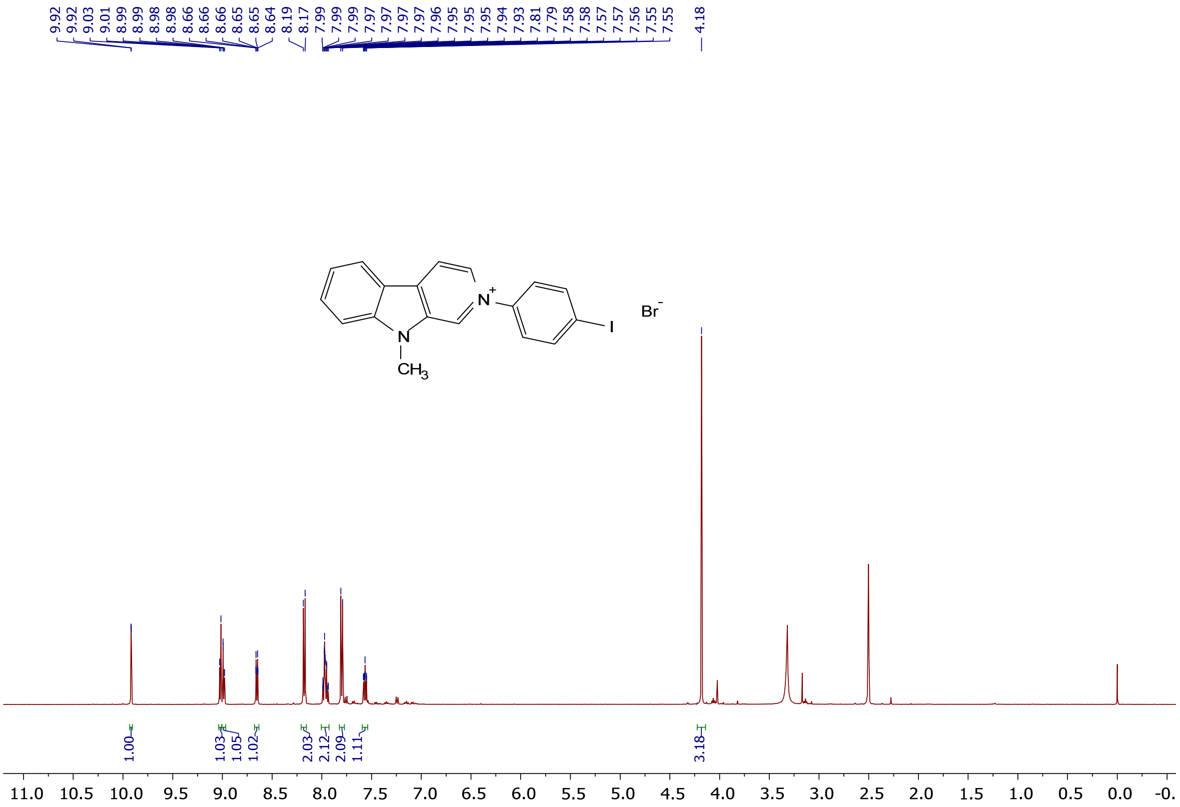

^{1}H NMR of Compound B**13**

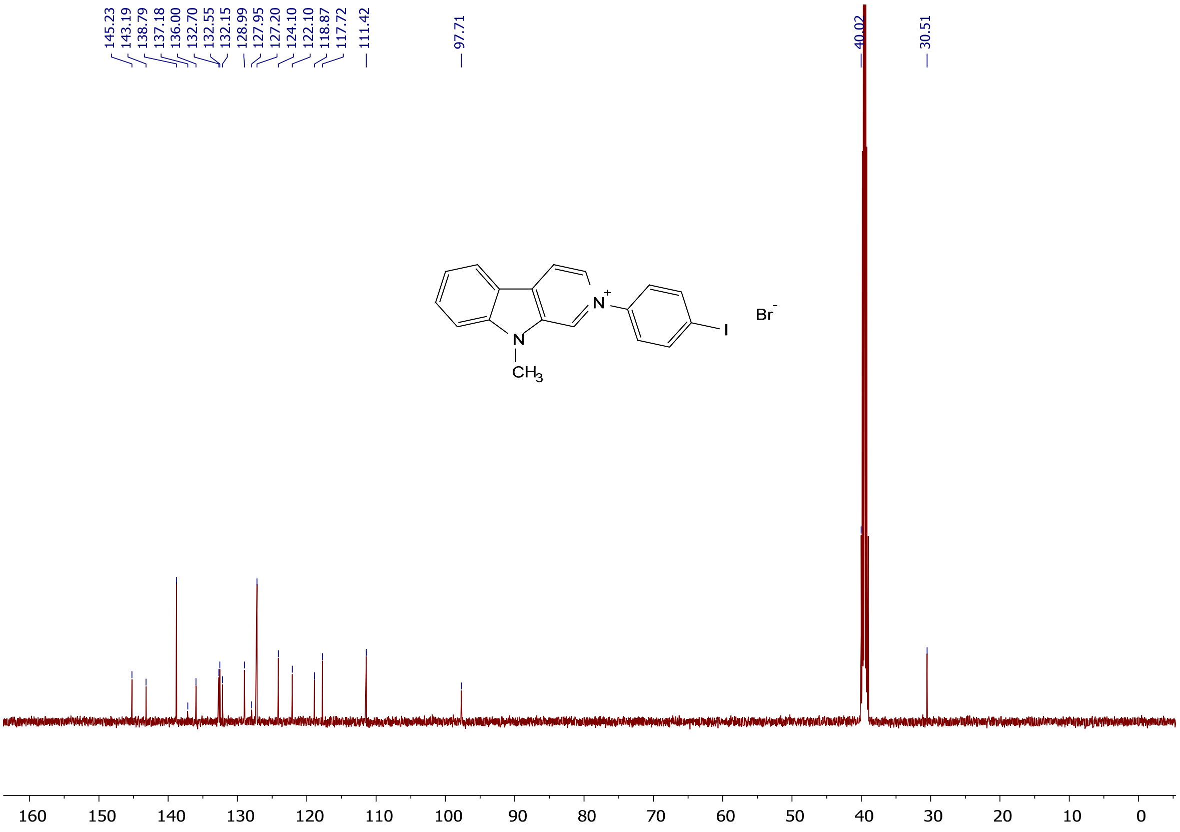

^{13}C NMR of Compound B**13**

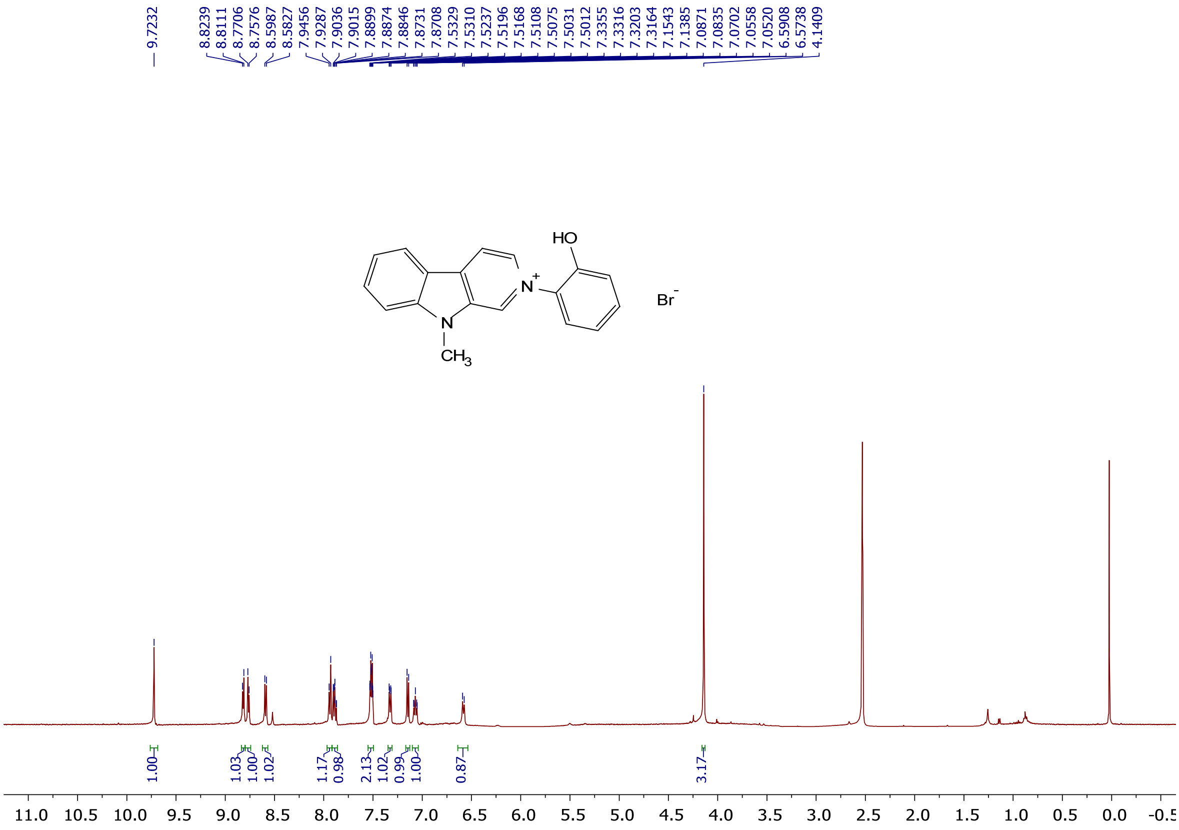

^{1}H NMR of Compound B**14**

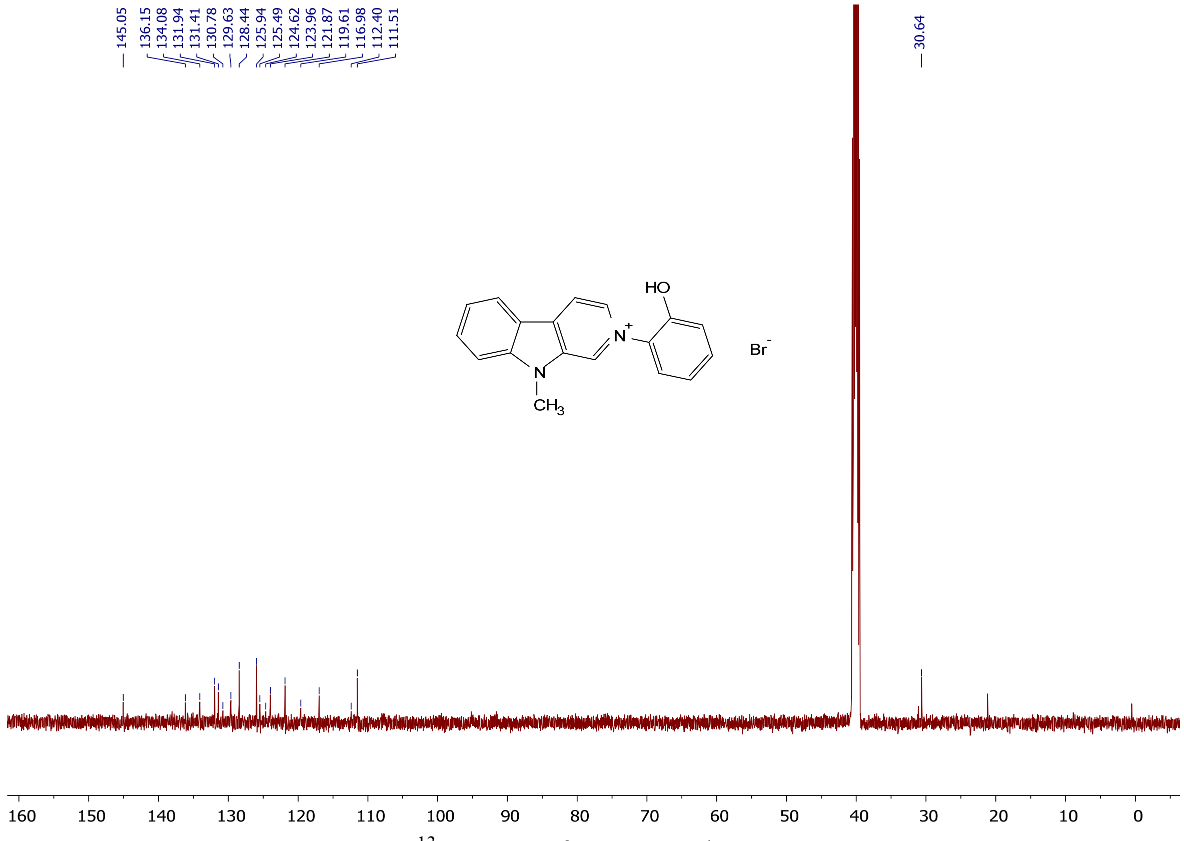

^{13}C NMR of Compound B**14**

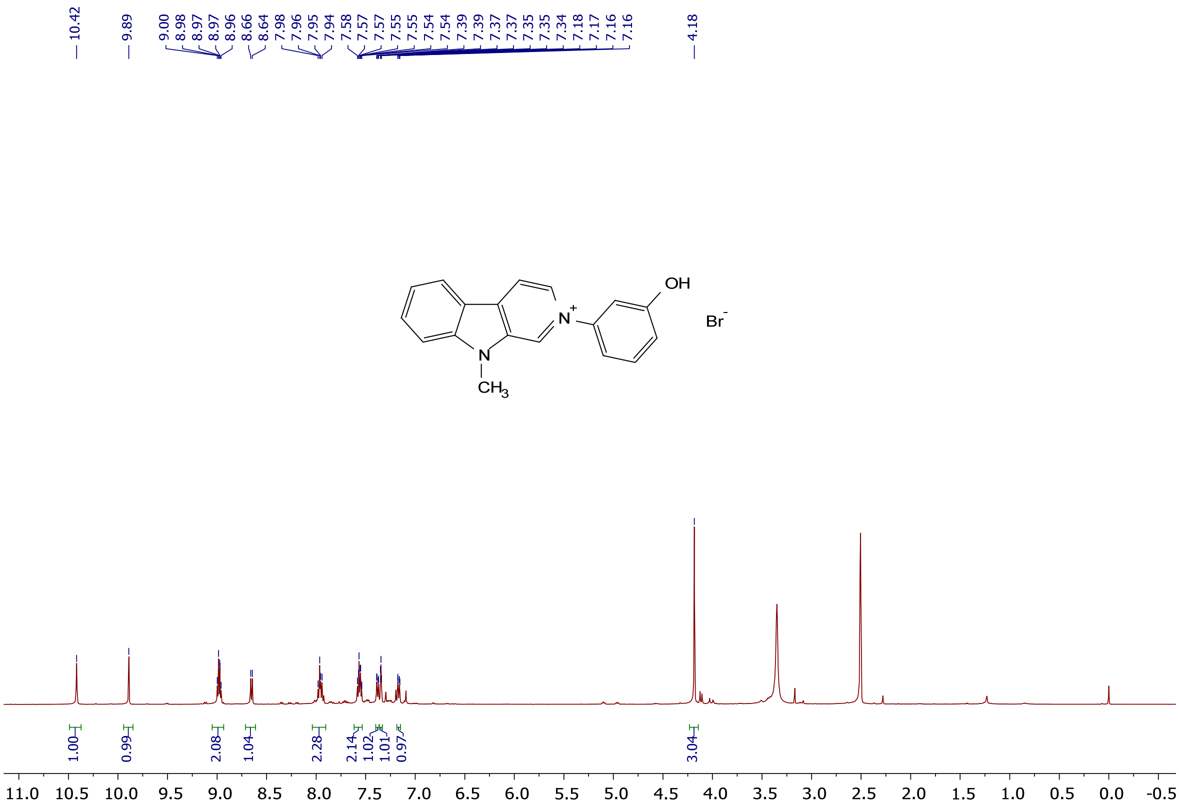

^{1}H NMR of Compound B**15**

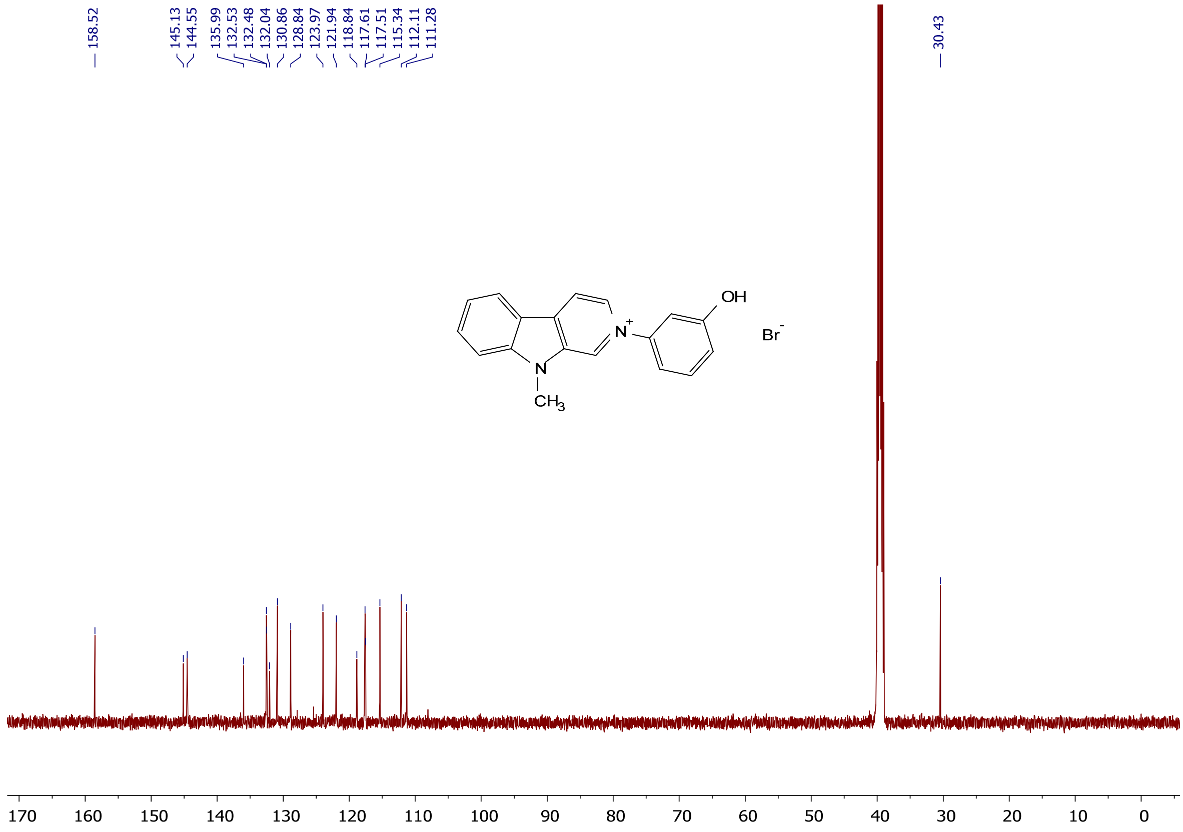

^{13}C NMR of Compound B**15**

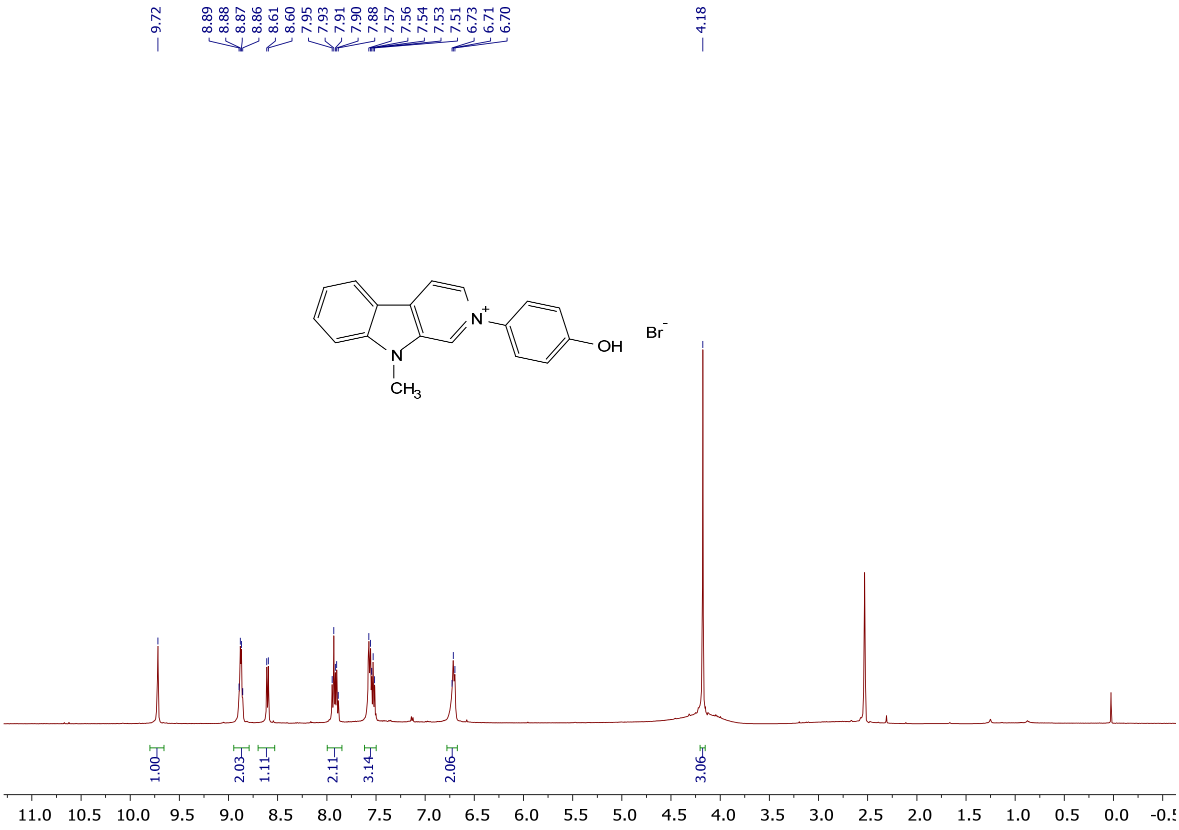

^{1}H NMR of Compound B**16**

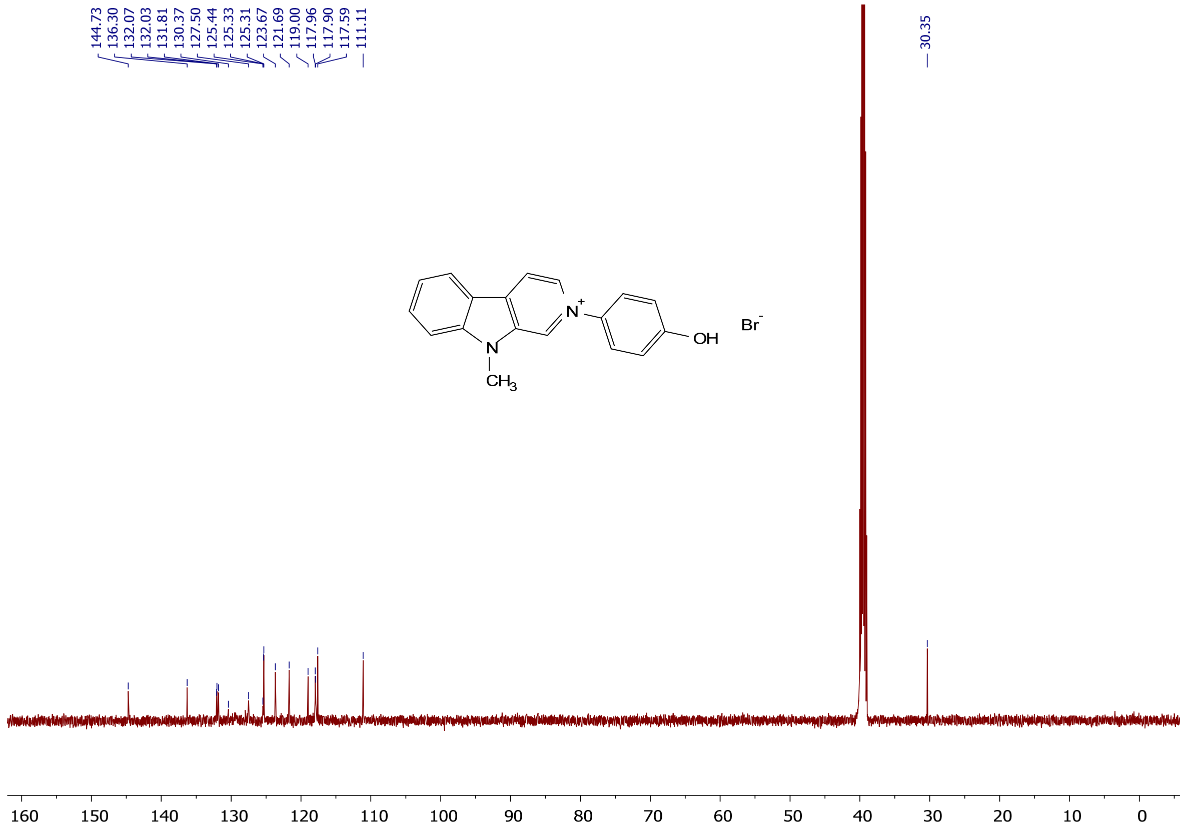

^{13}C NMR of Compound B**16**

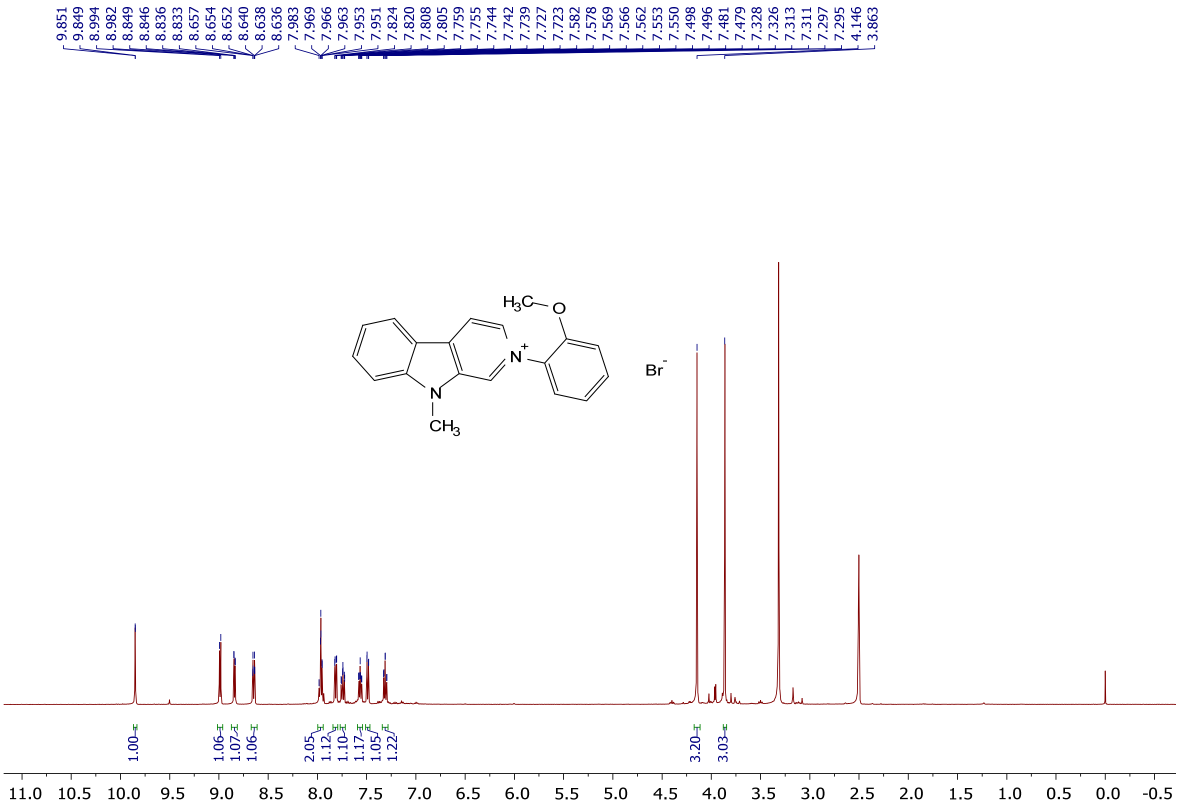

^{1}H NMR of Compound B**17**

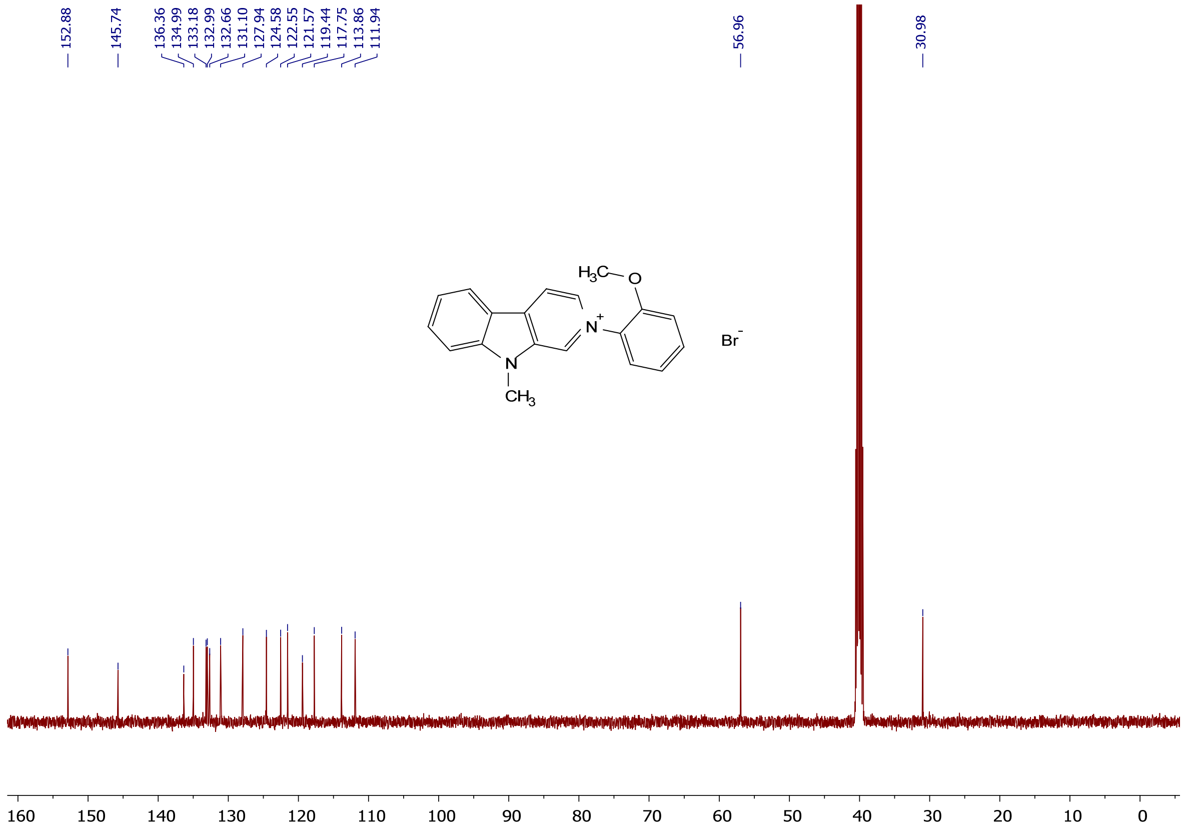

^{13}C NMR of Compound B**17**

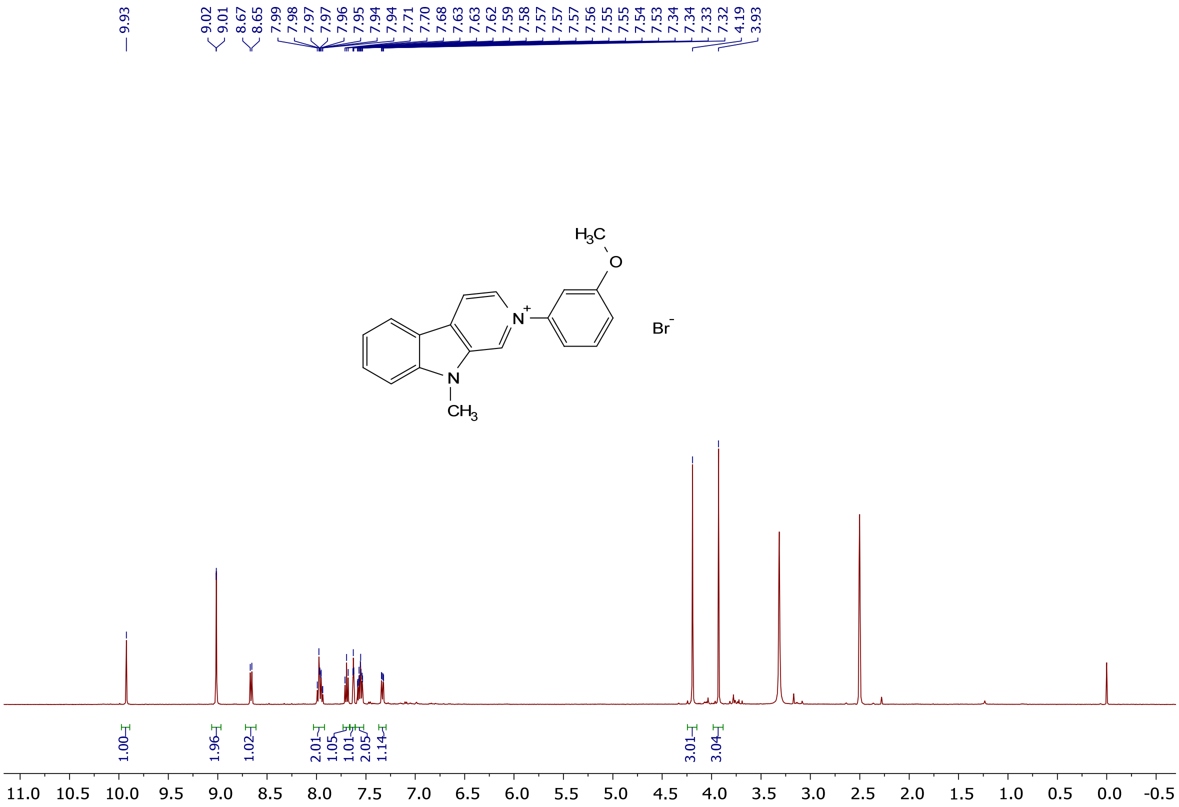

^{1}H NMR of Compound B**18**

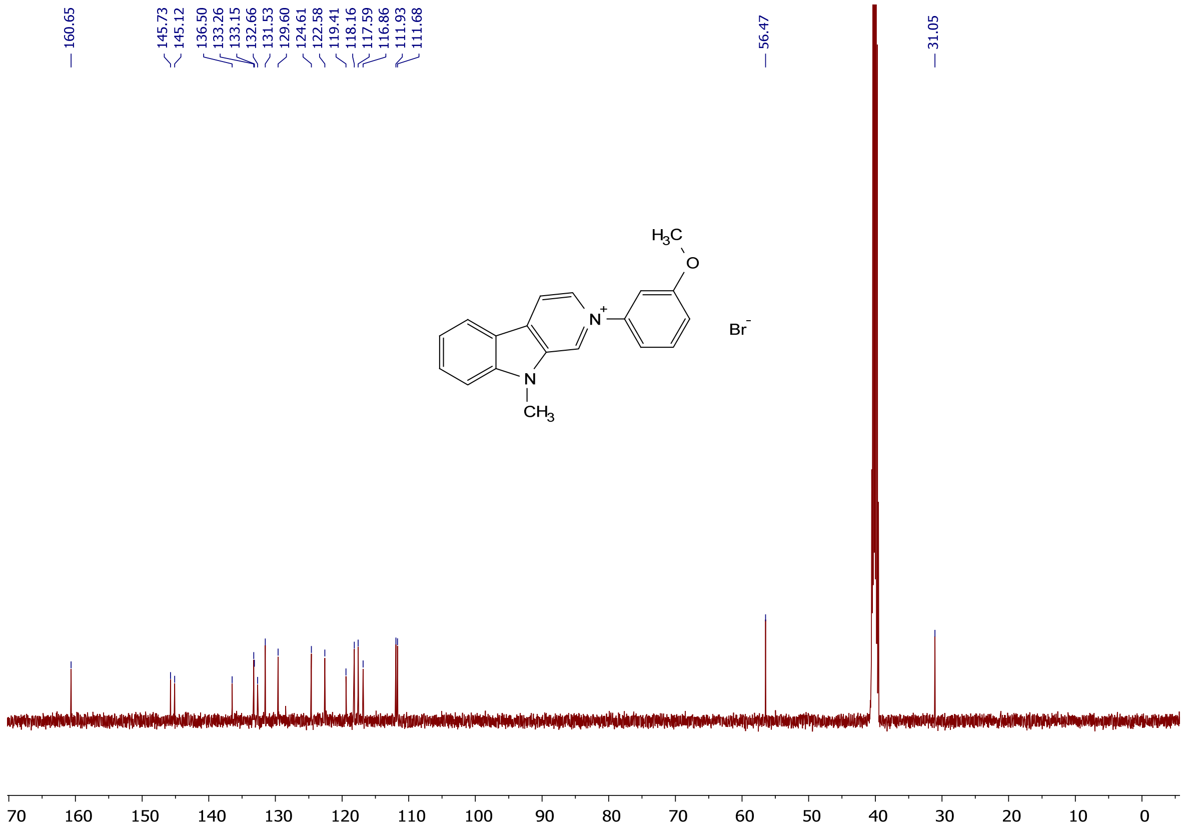

^{13}C NMR of Compound B**18**

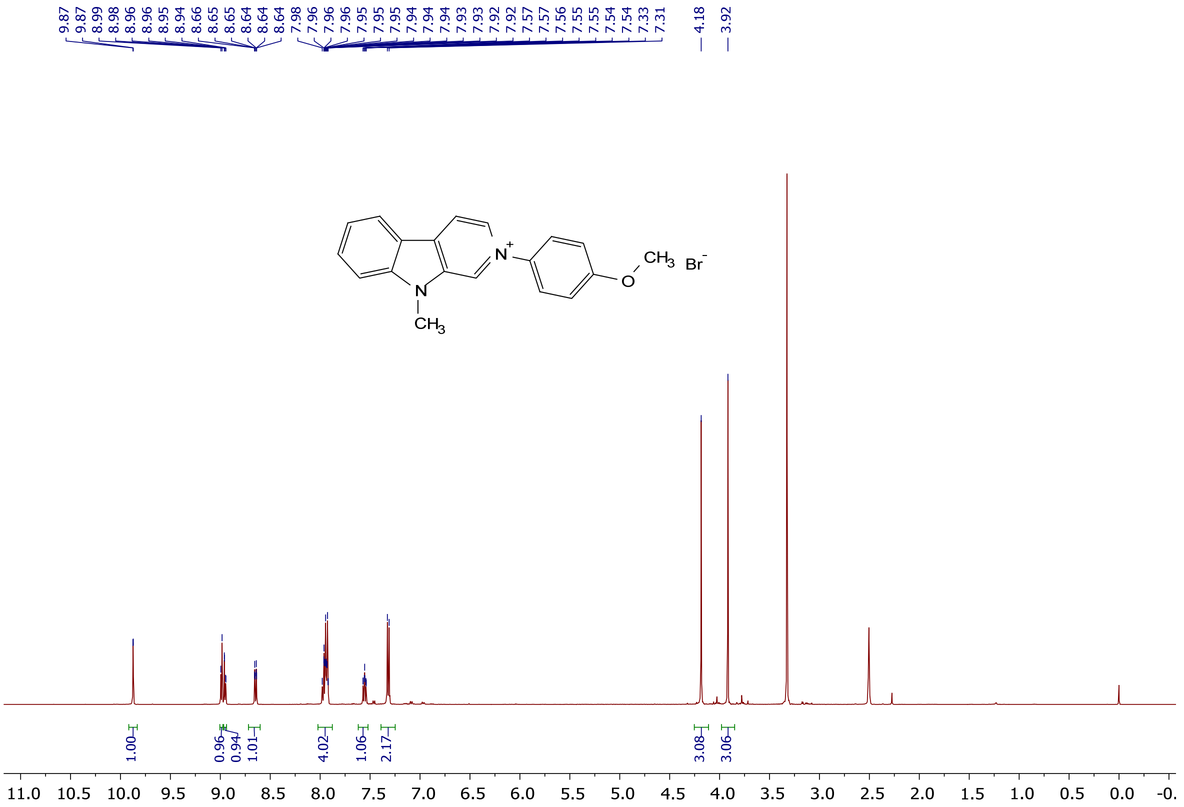

^{1}H NMR of Compound B**19**

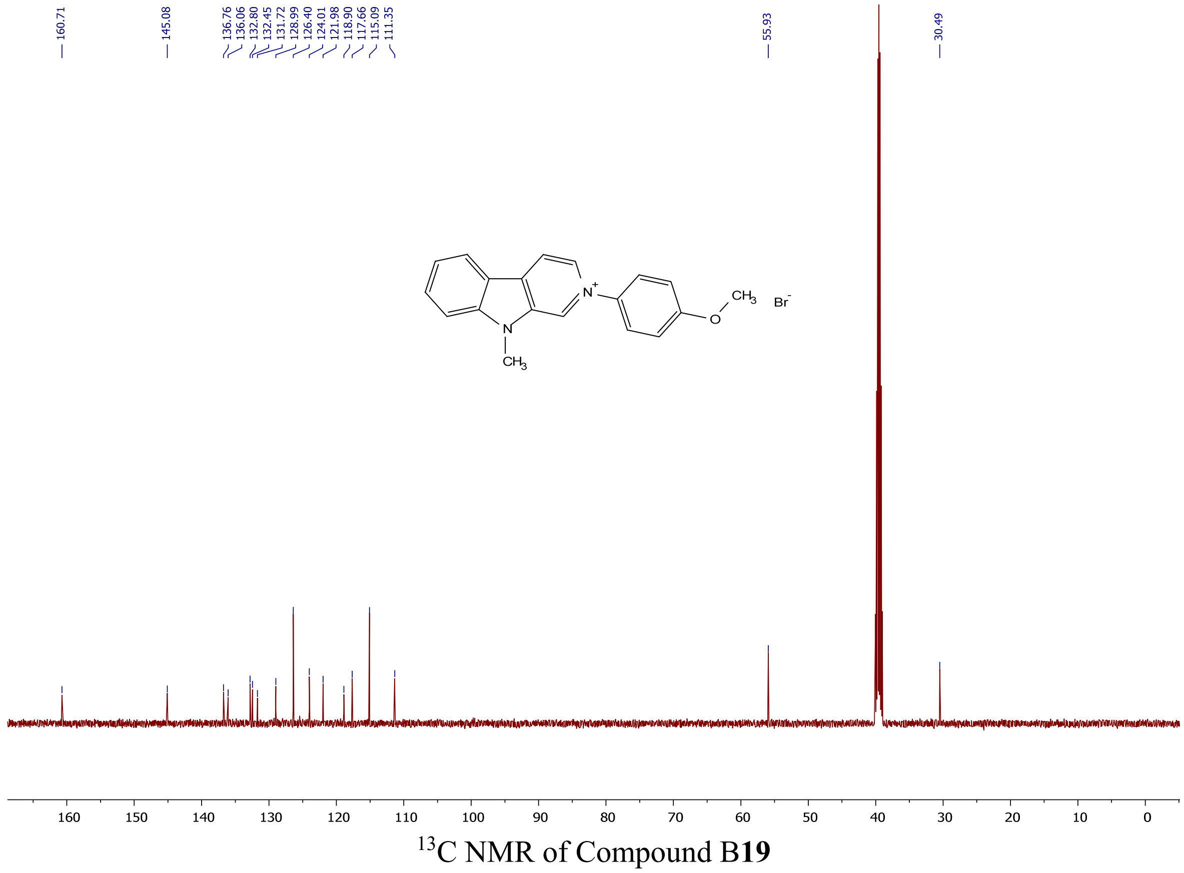

^{13}C NMR of Compound B**19**

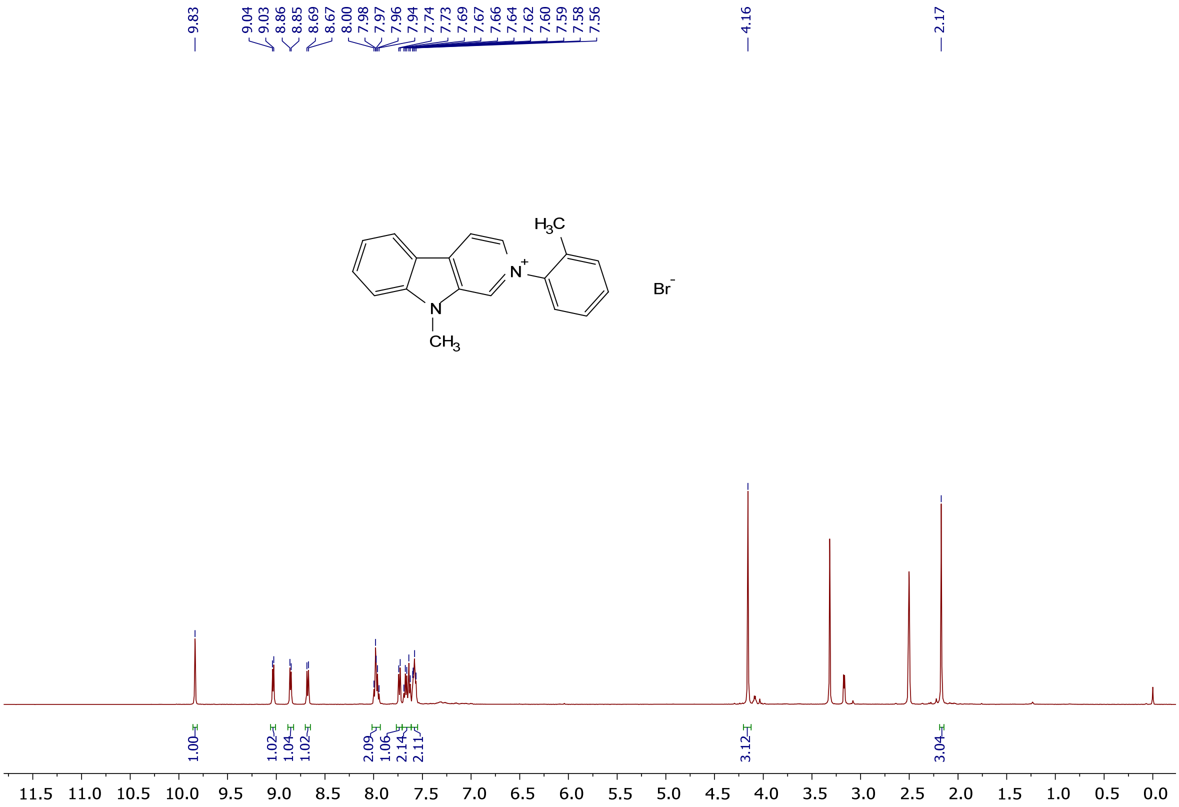

^{1}H NMR of Compound B**20**

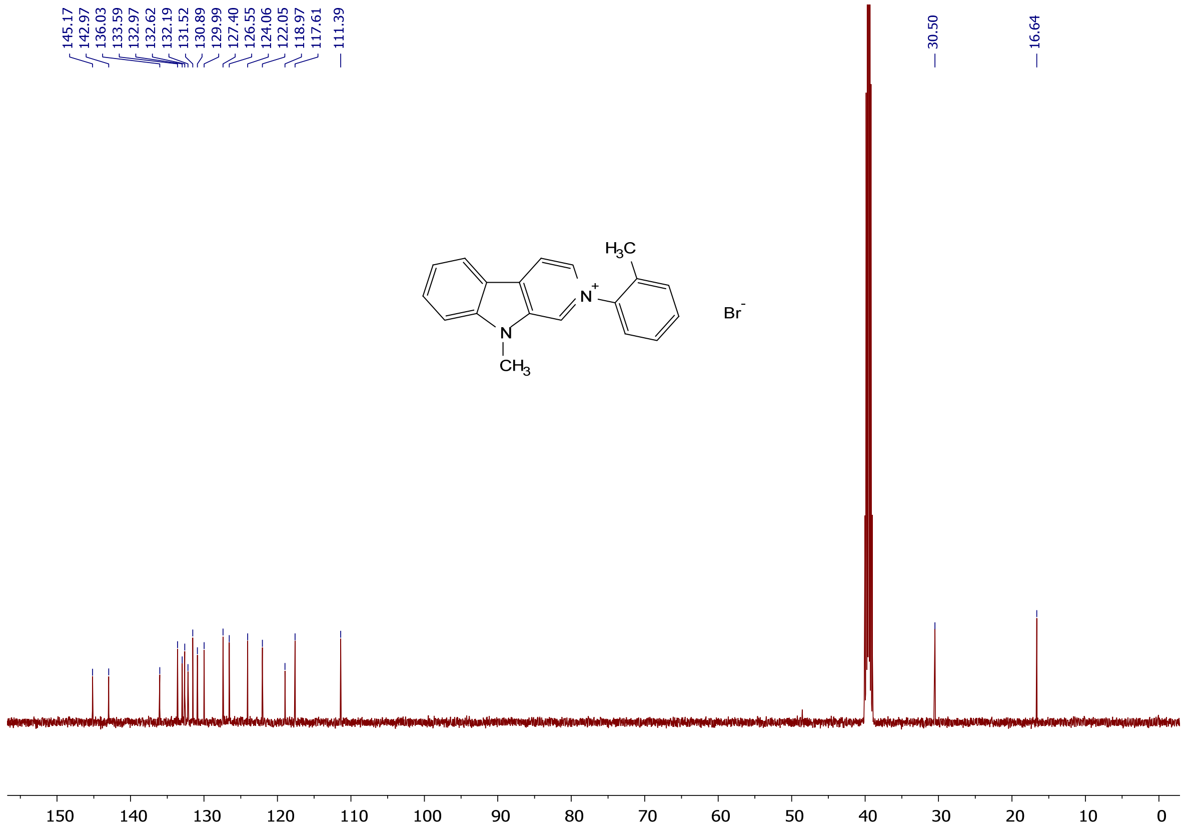

^{13}C NMR of Compound B**20**

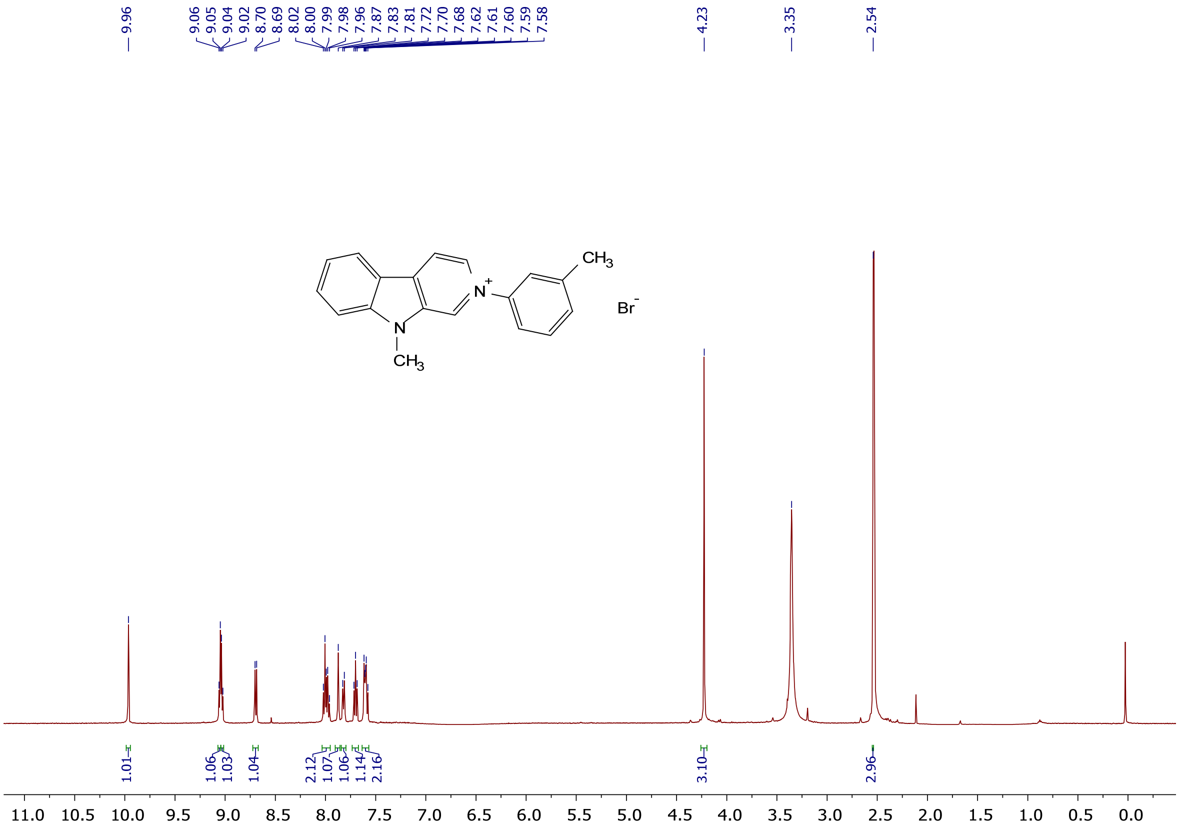

^{1}H NMR of Compound B**21**

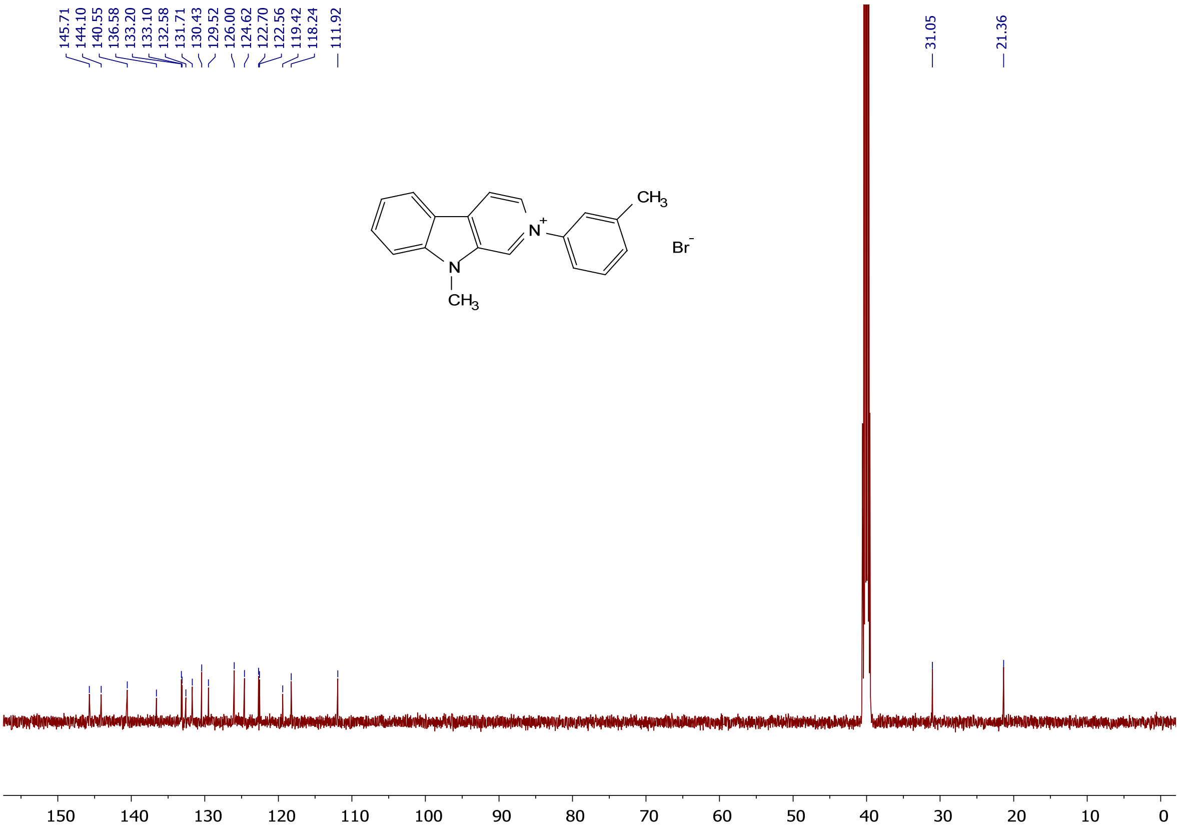

^{13}C NMR of Compound B**21**

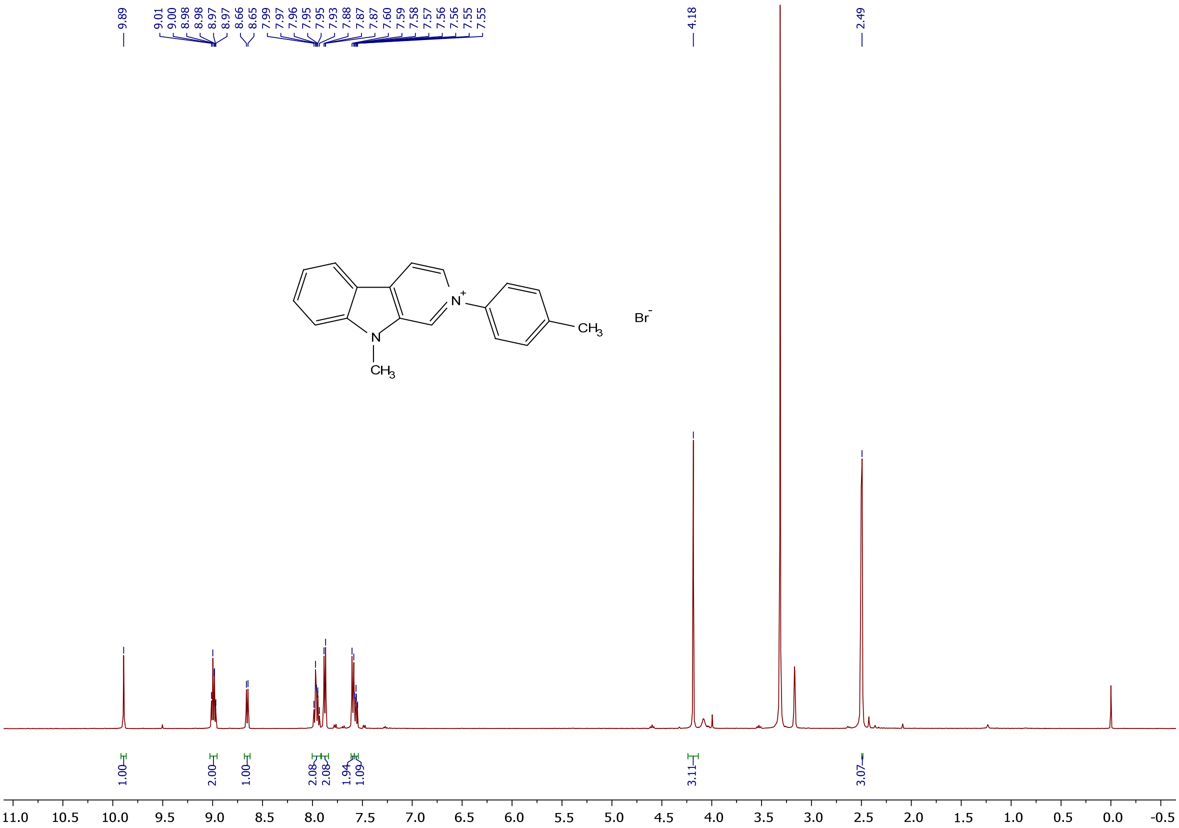

^{1}H NMR of Compound B**22**

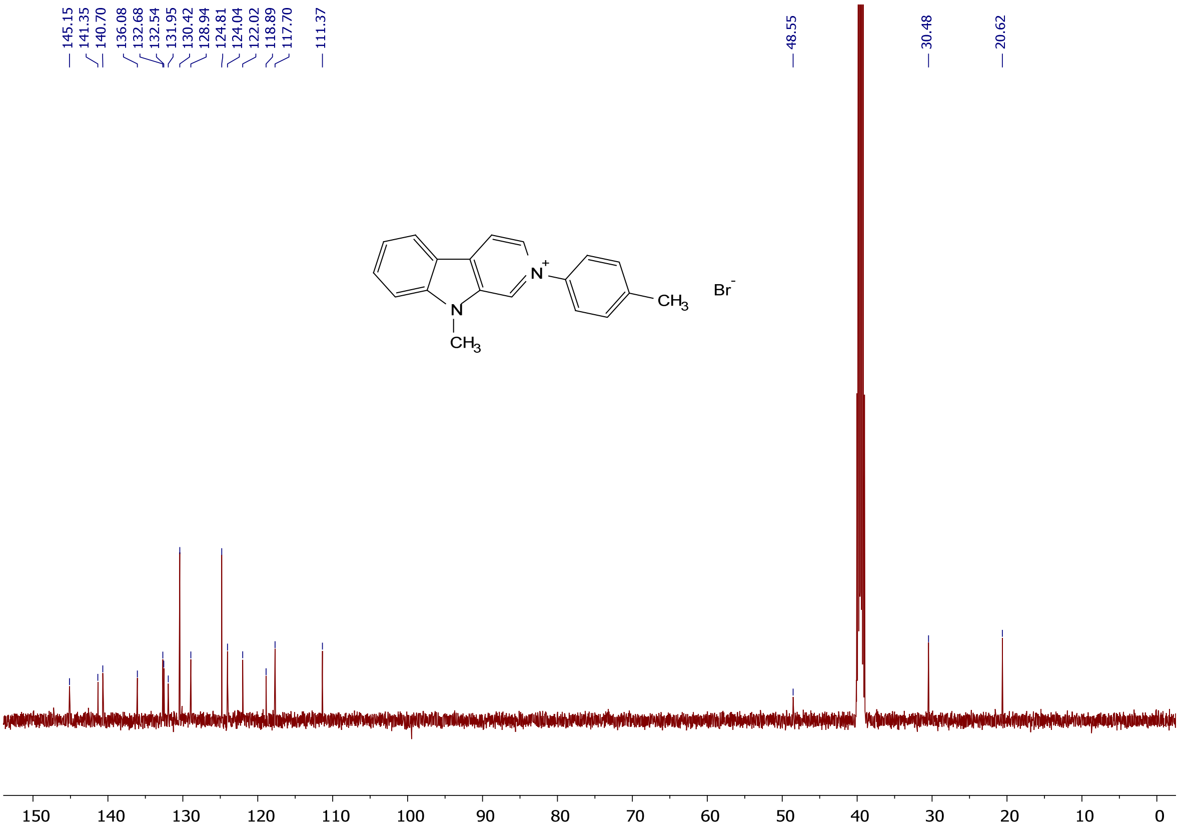

^{13}C NMR of Compound B**22**

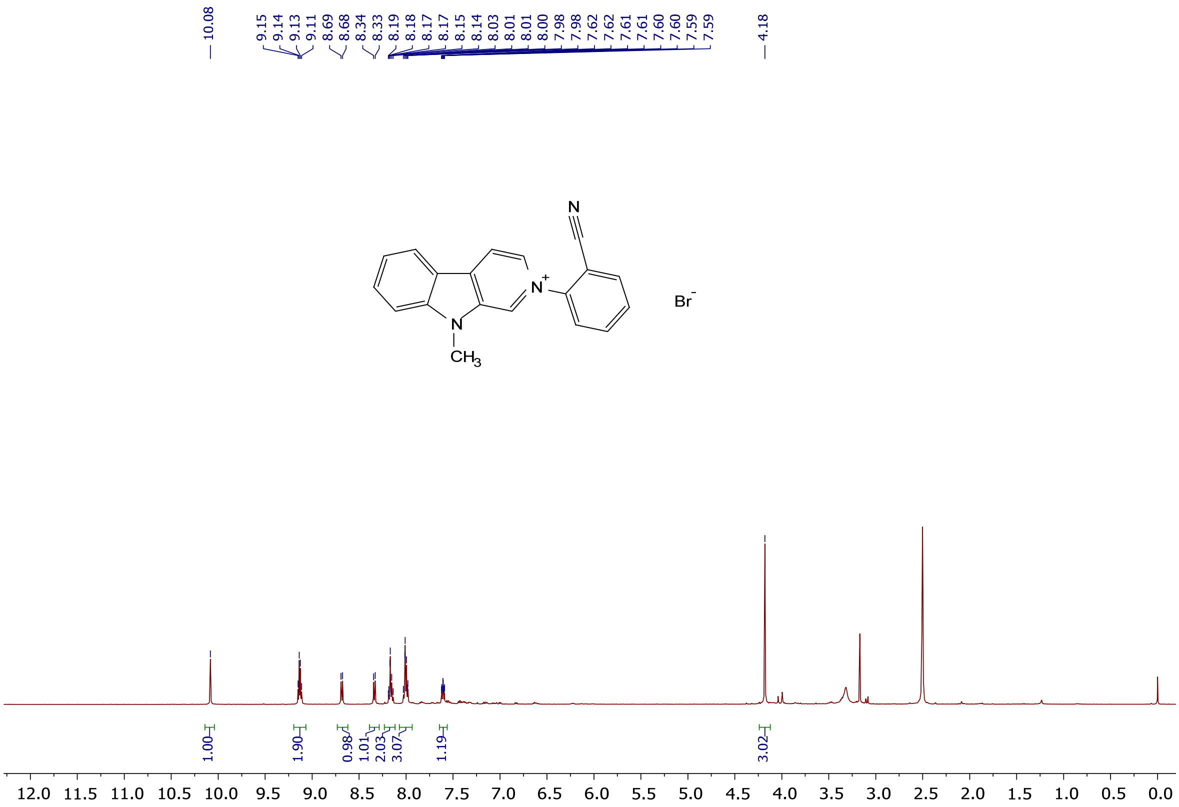

^{1}H NMR of Compound B**23**

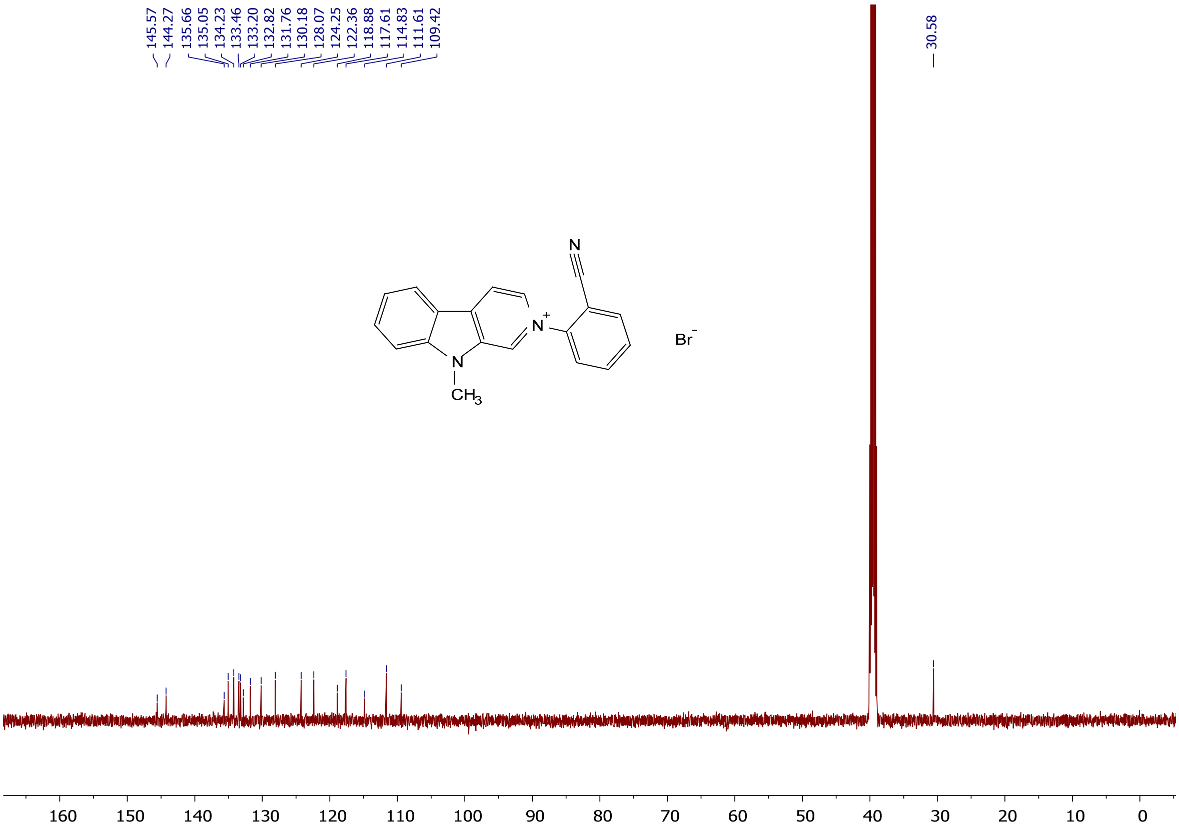

^{13}C NMR of Compound B**23**

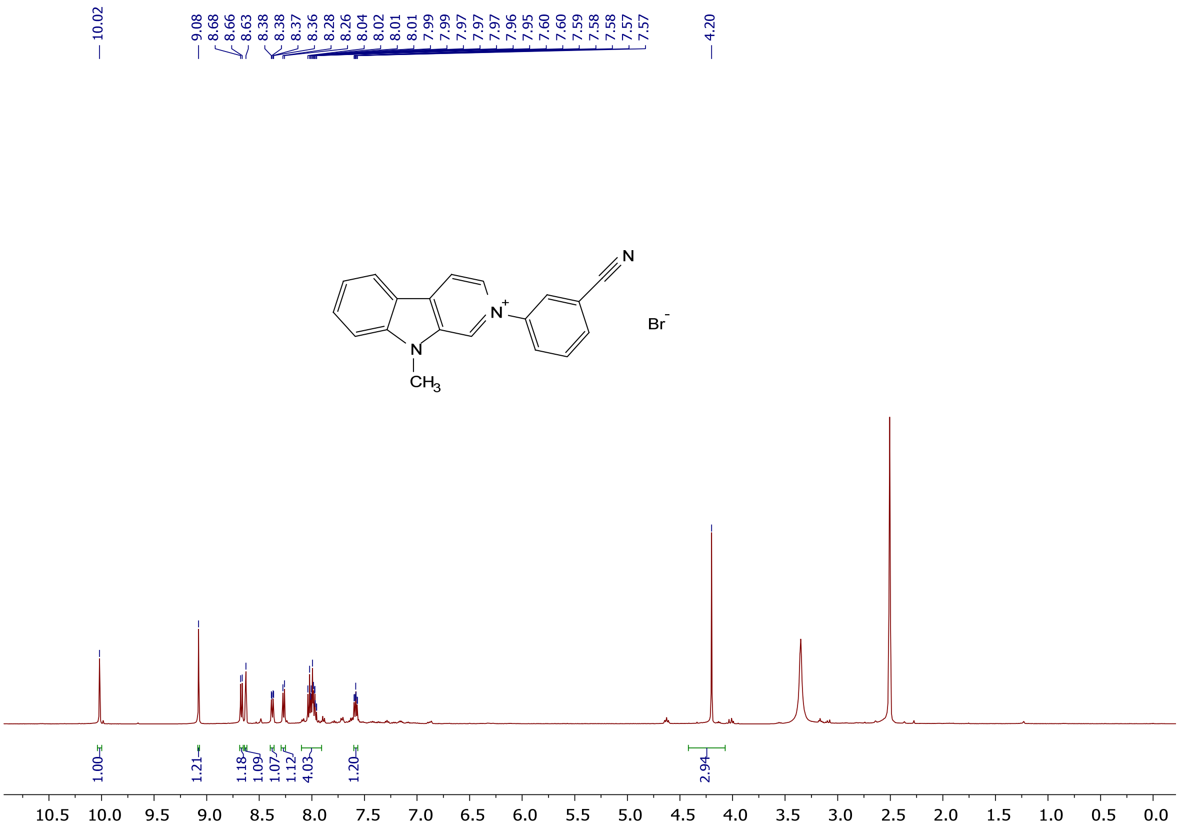

^{1}H NMR of Compound B**24**

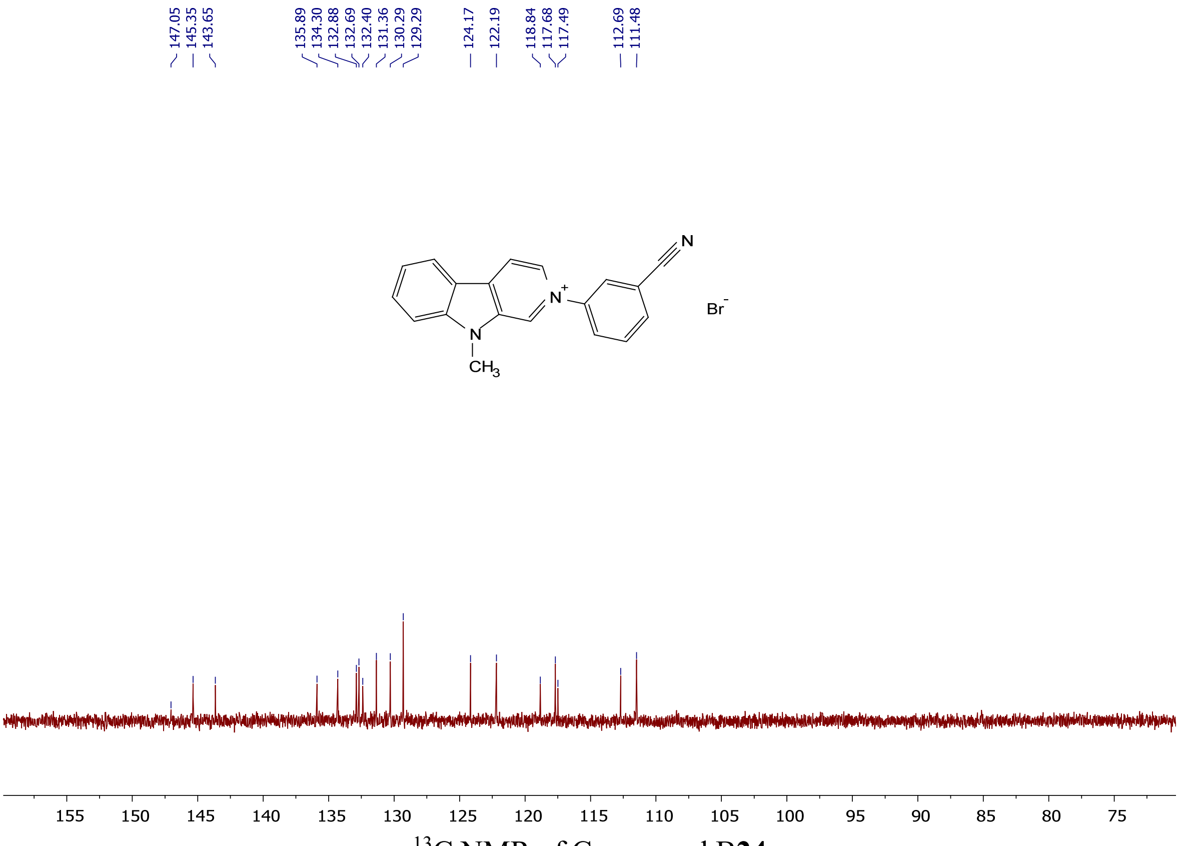

^{13}C NMR of Compound B**24**

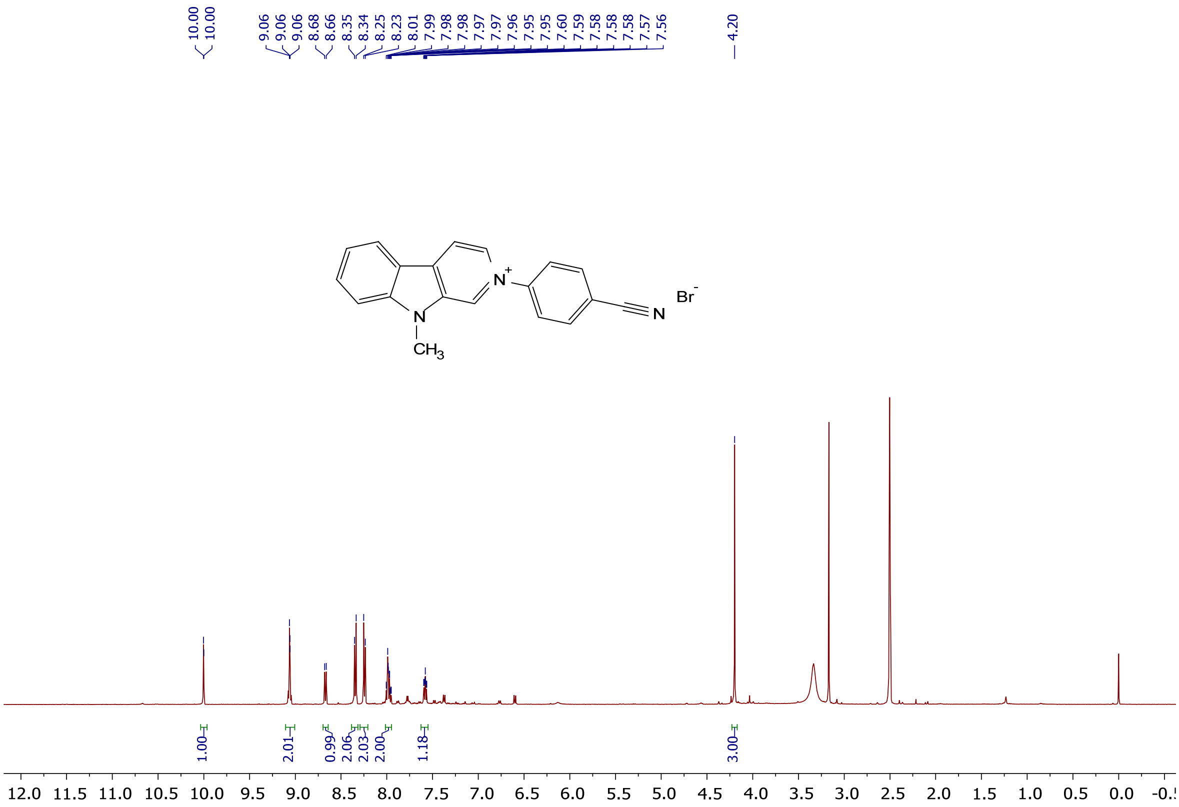

^{1}H NMR of Compound B**25**

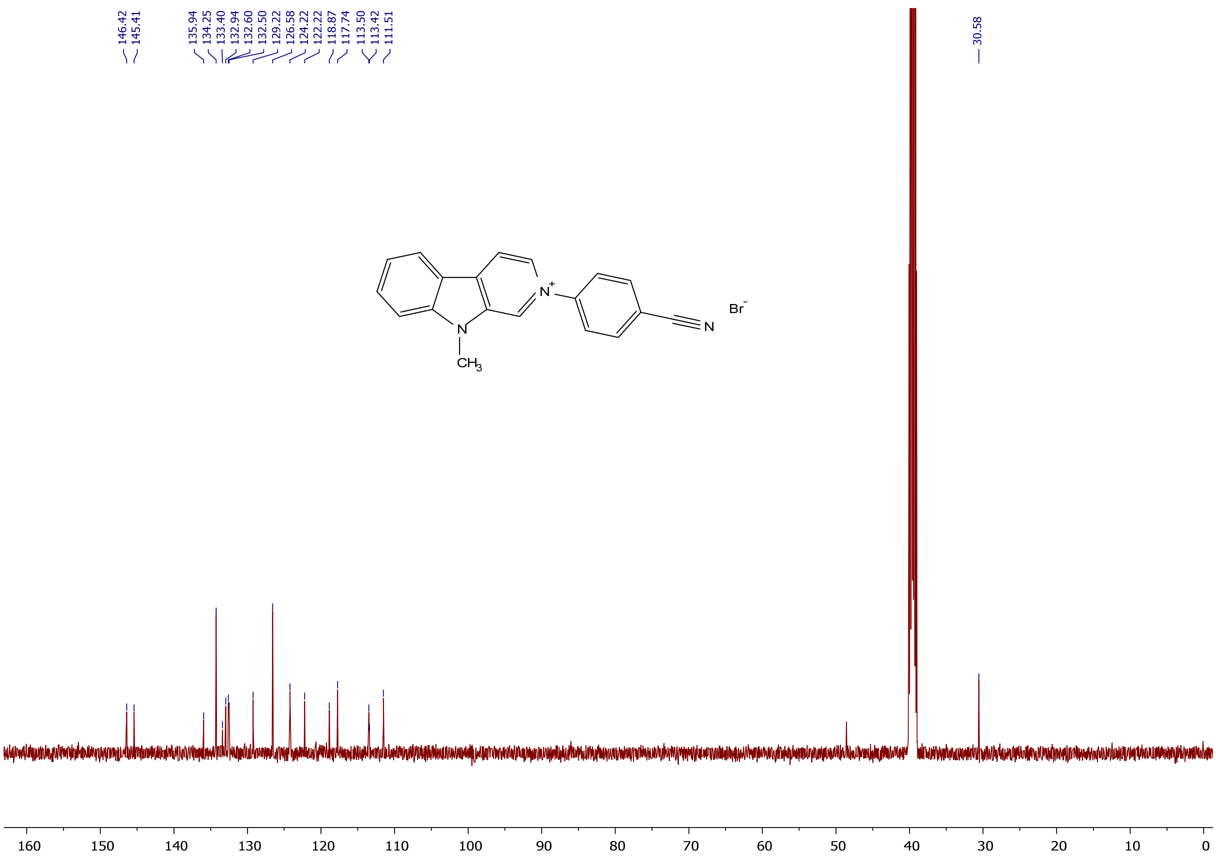

^{13}C NMR of Compound B**25**

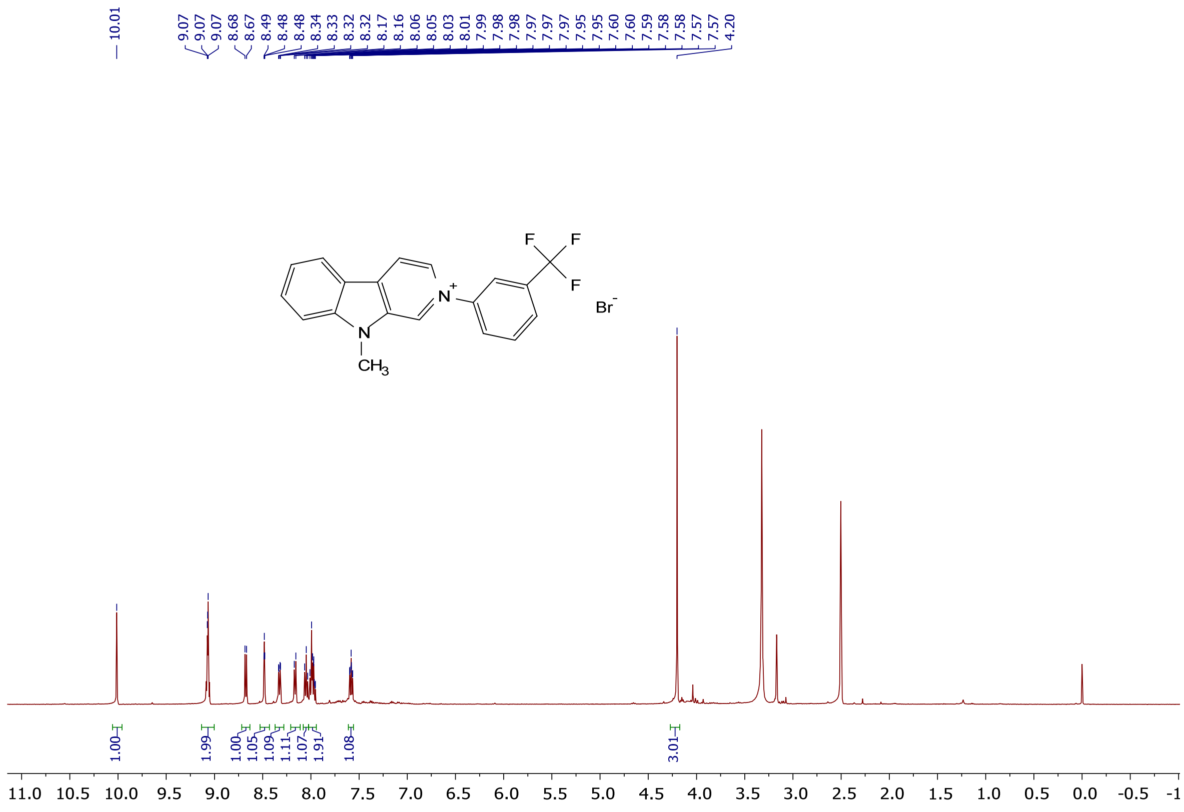

^{1}H NMR of Compound B**26**

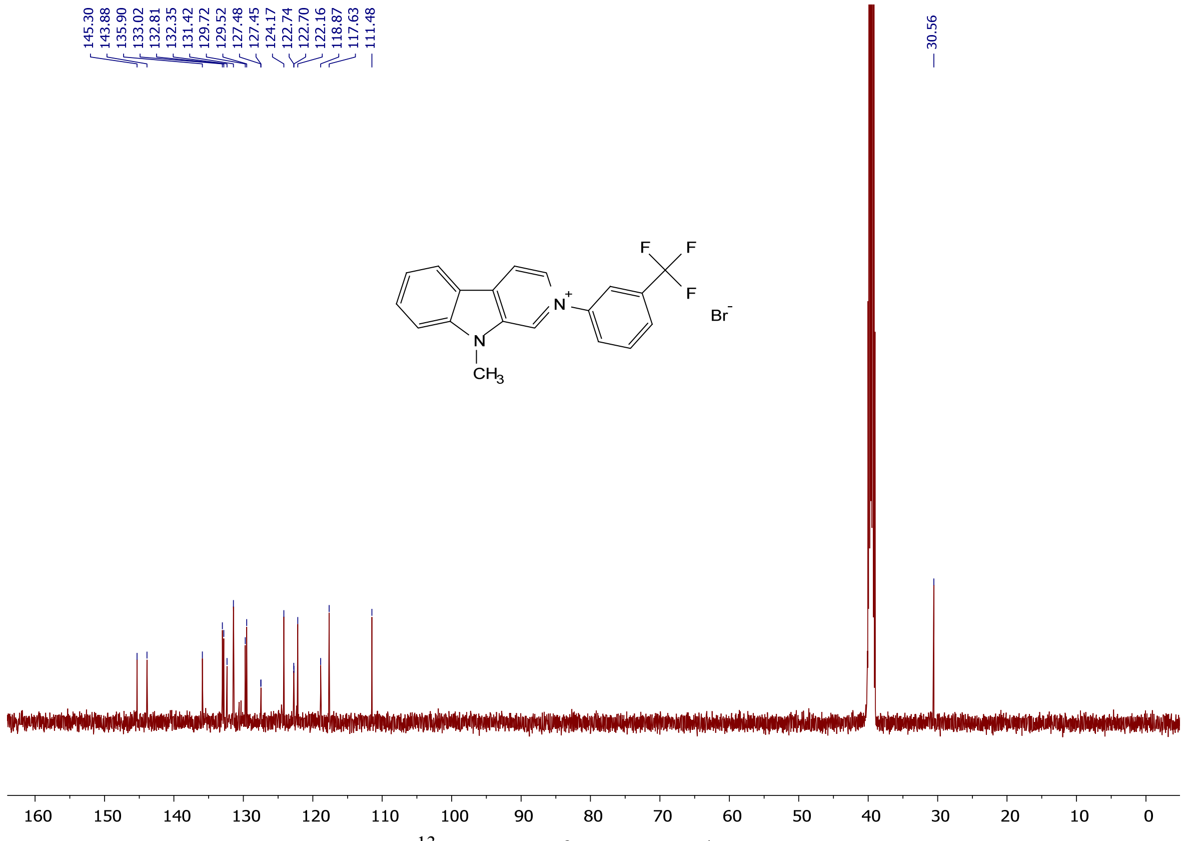

^{13}C NMR of Compound B**26**

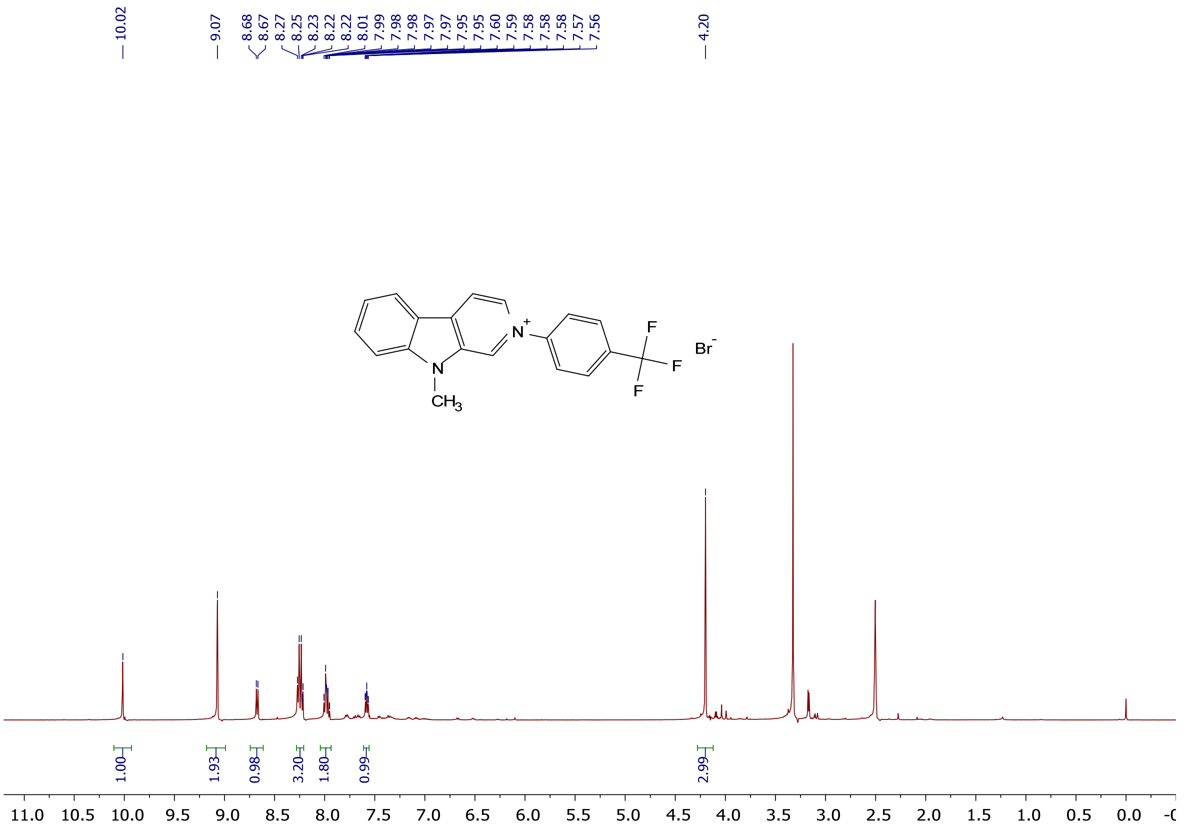

^{1}H NMR of Compound B**27**

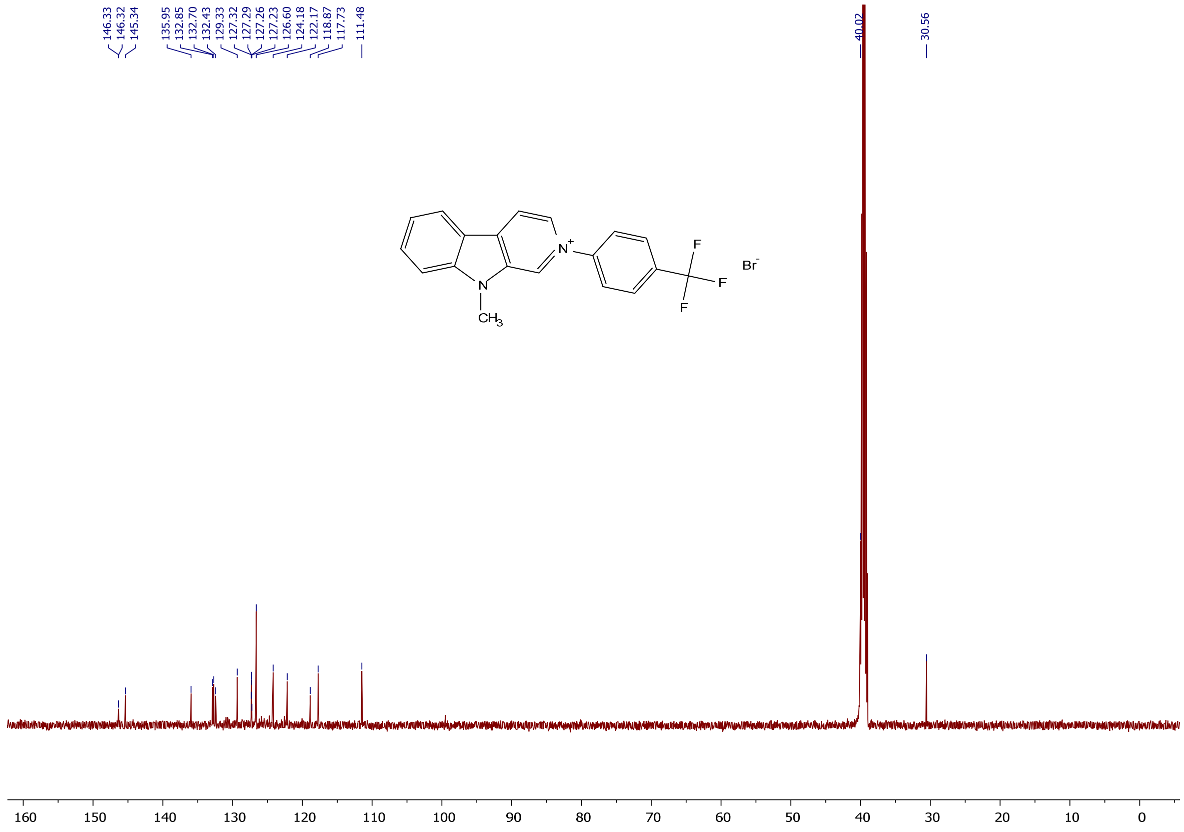

^{13}C NMR of Compound B**27**

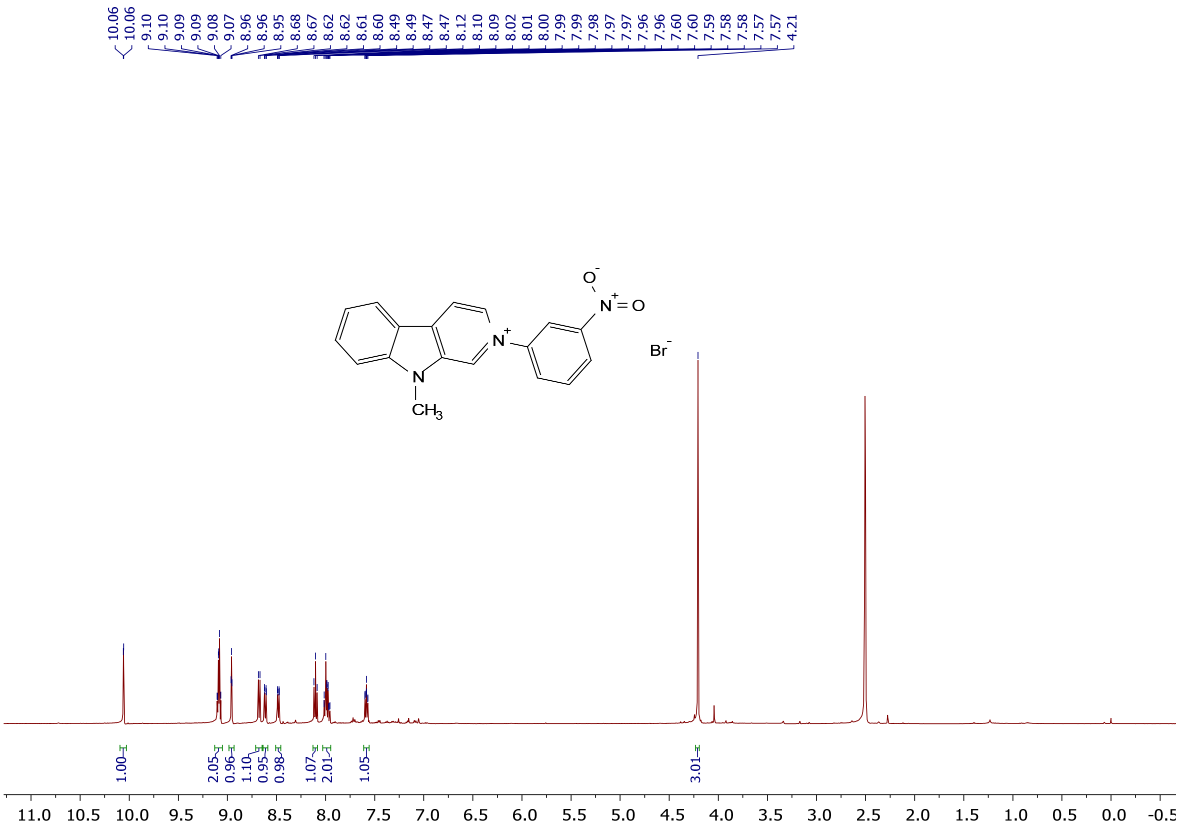

^{1}H NMR of Compound B**28**

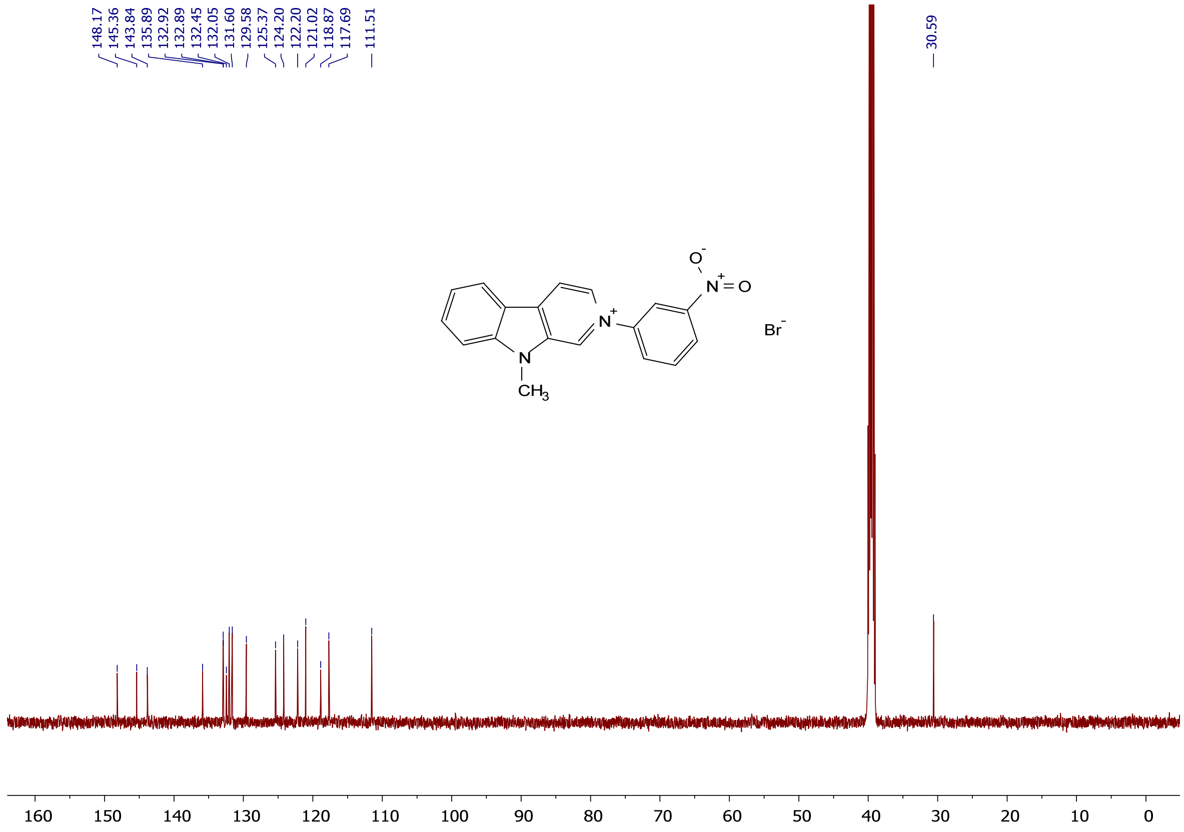

^{13}C NMR of Compound B**28**

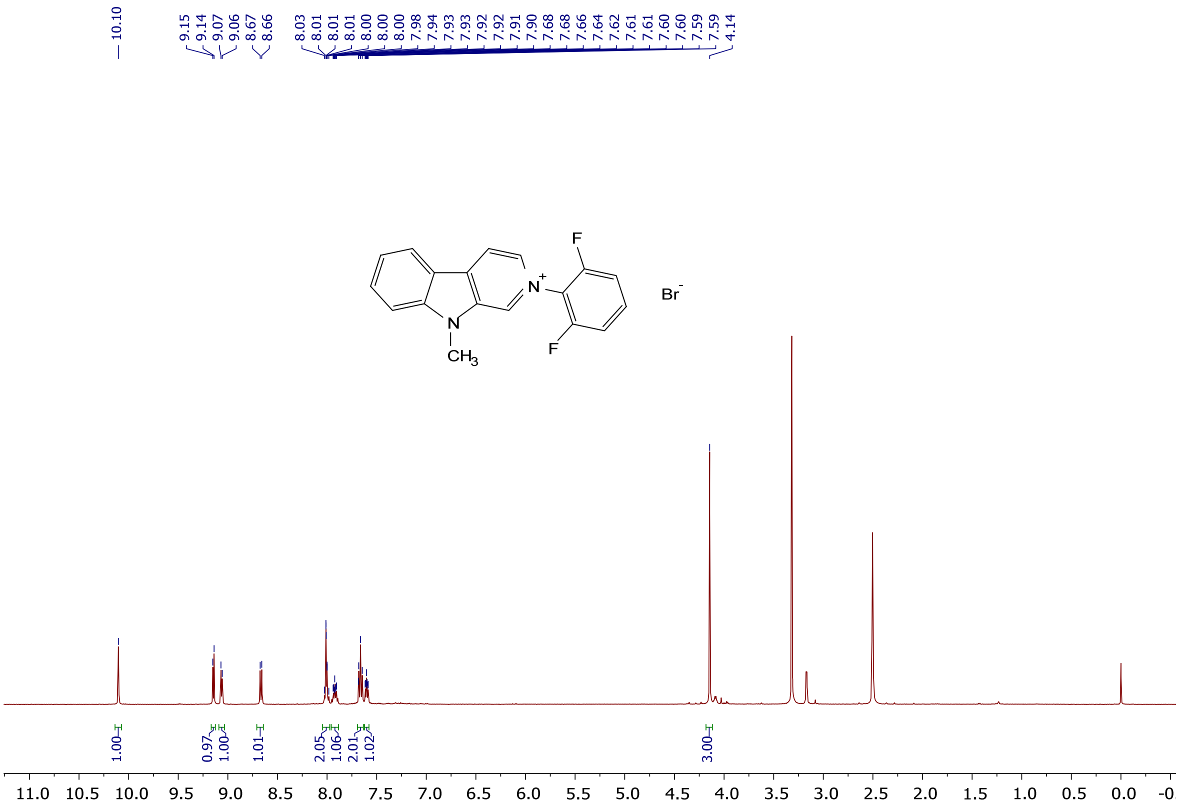

^{1}H NMR of Compound B**29**

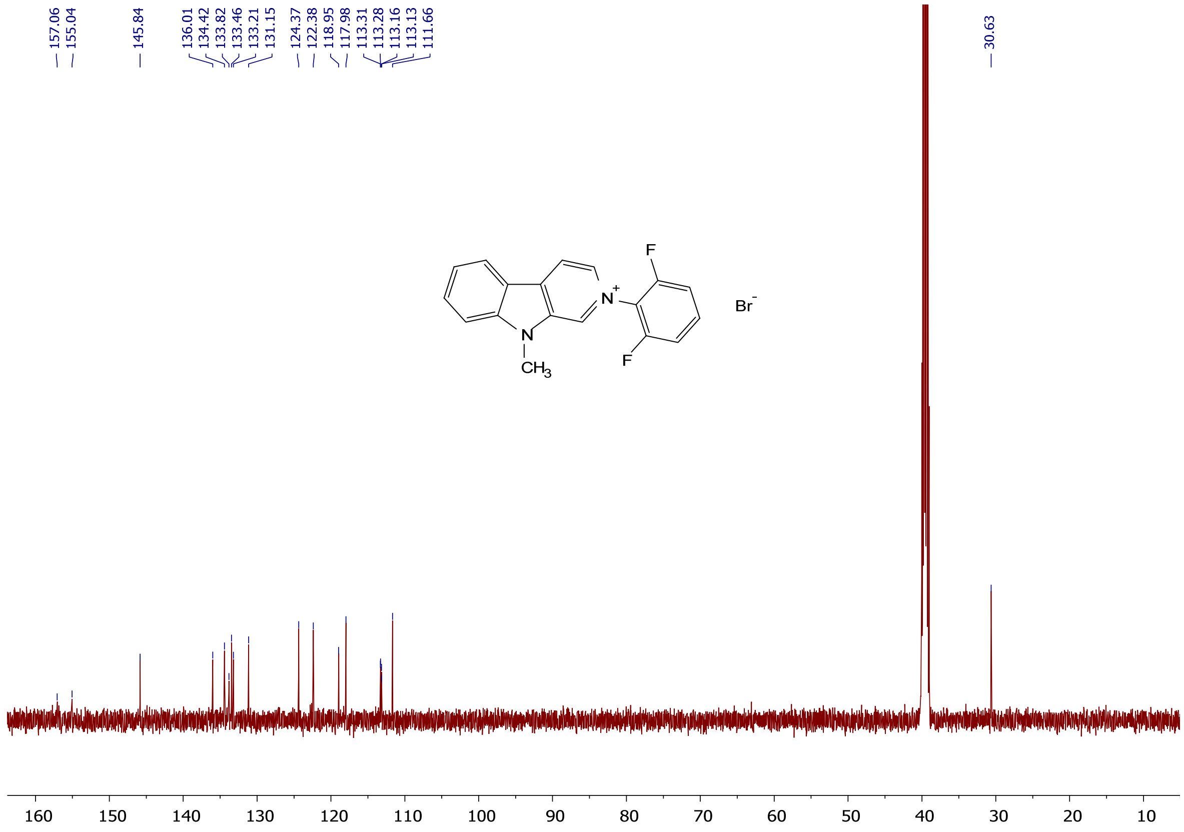

^{13}C NMR of Compound B**29**

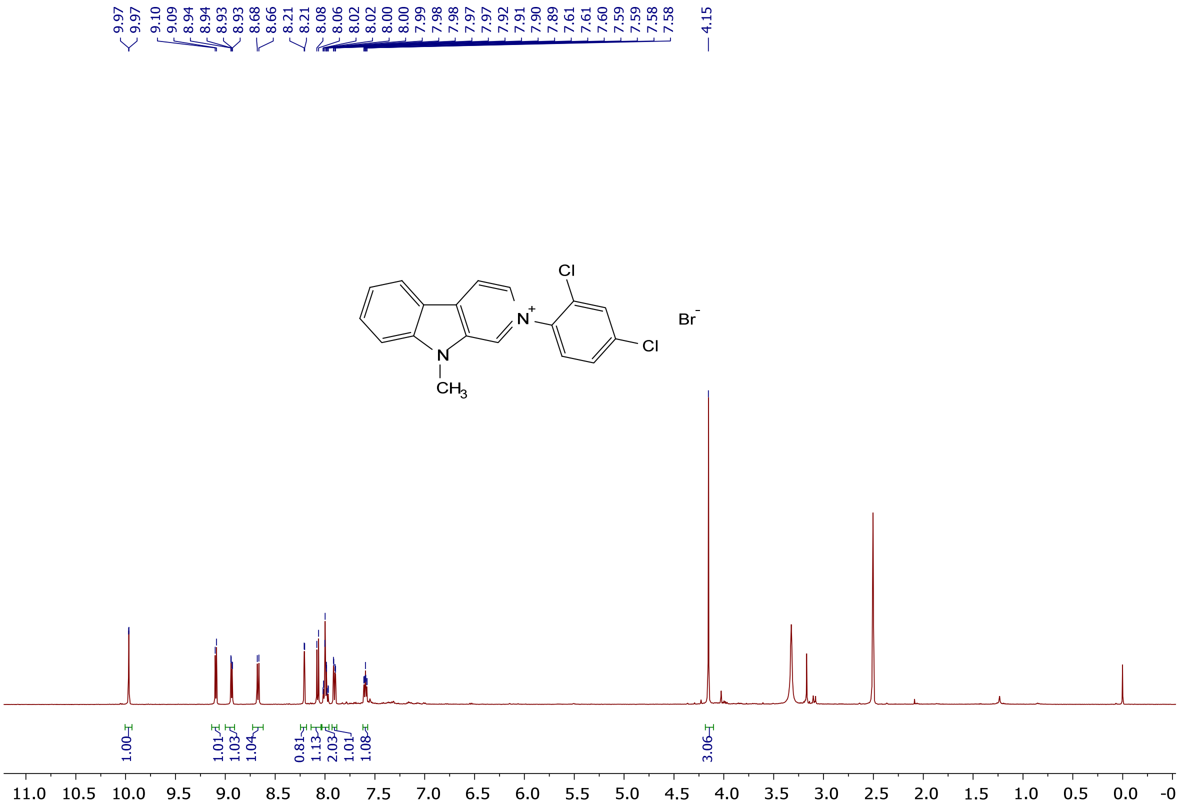

^{1}H NMR of Compound B**30**

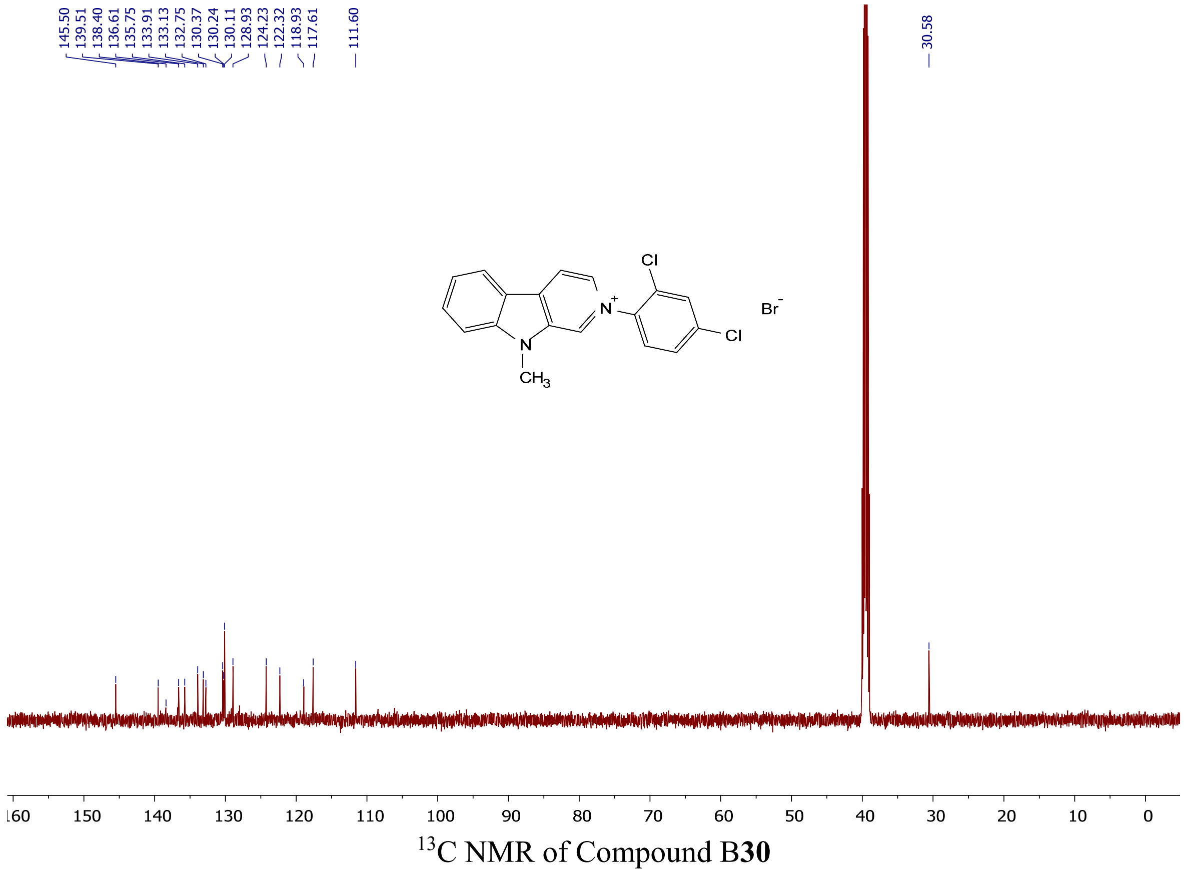

^{13}C NMR of Compound B**30**

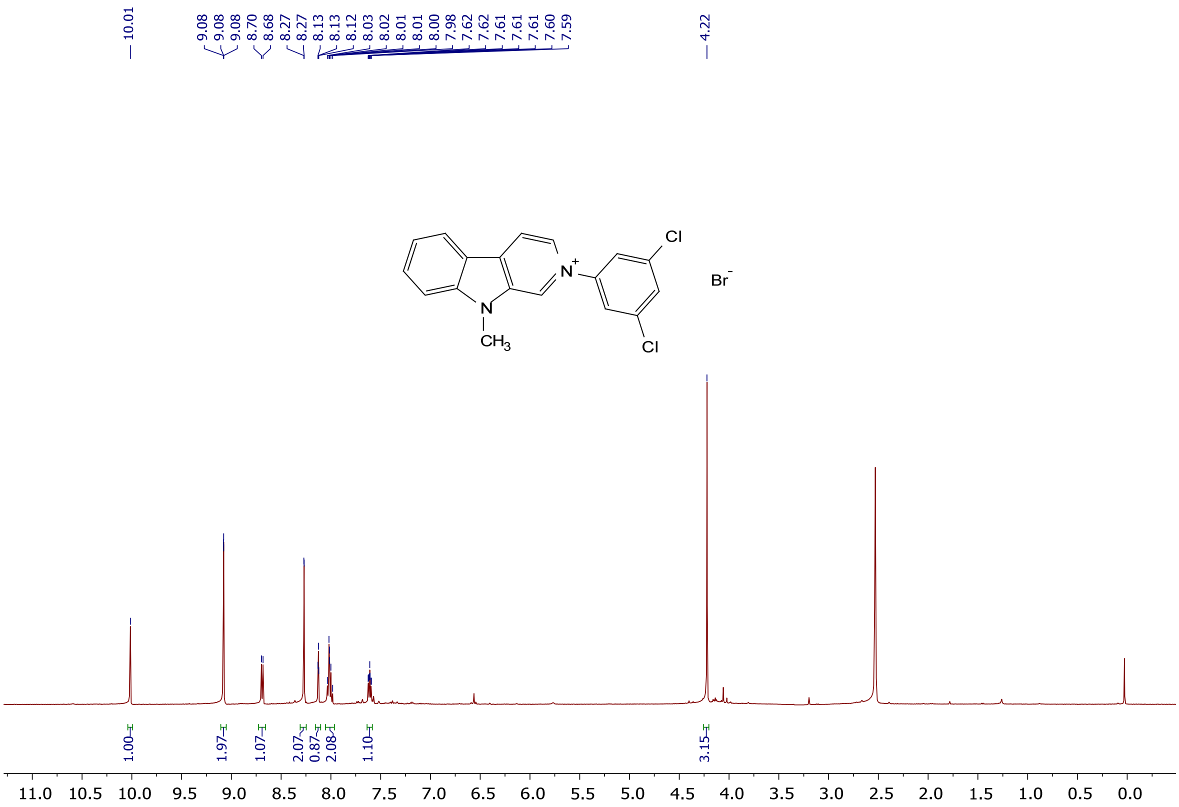

^{1}H NMR of Compound B**31**

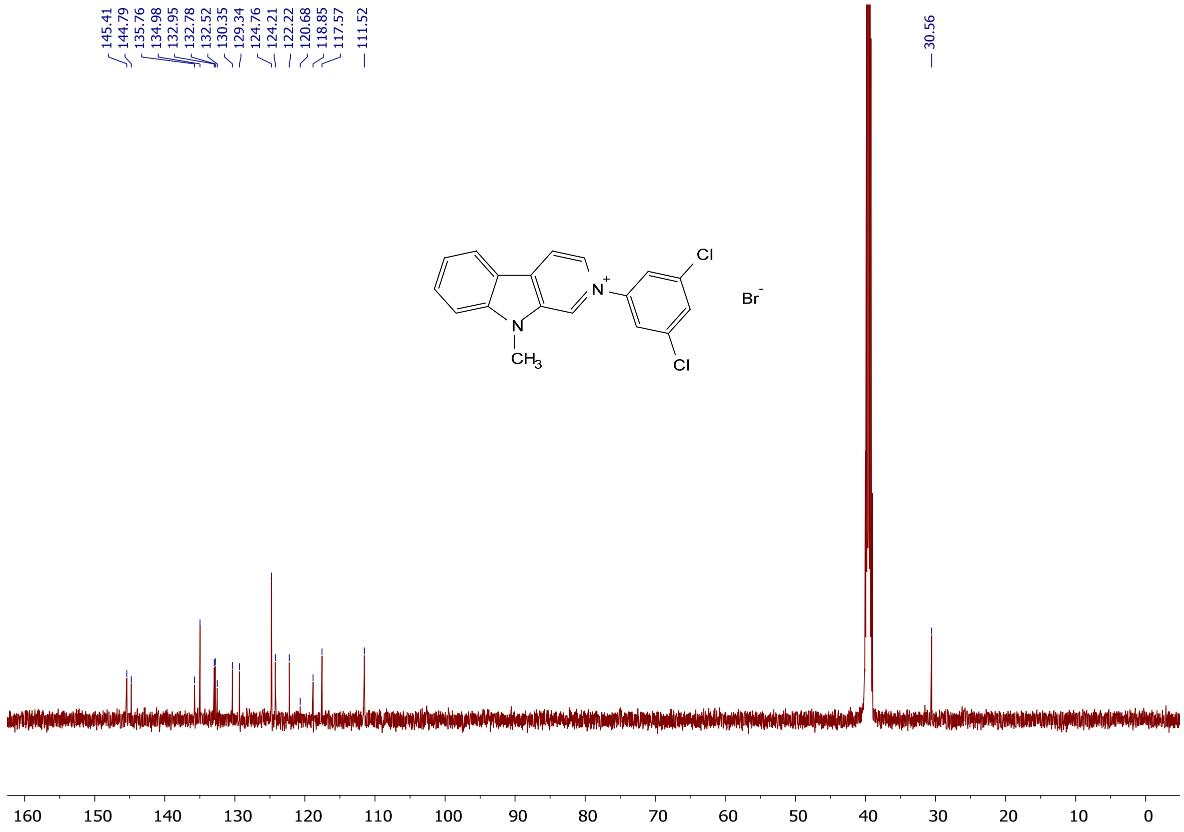

^{13}C NMR of Compound B**31**

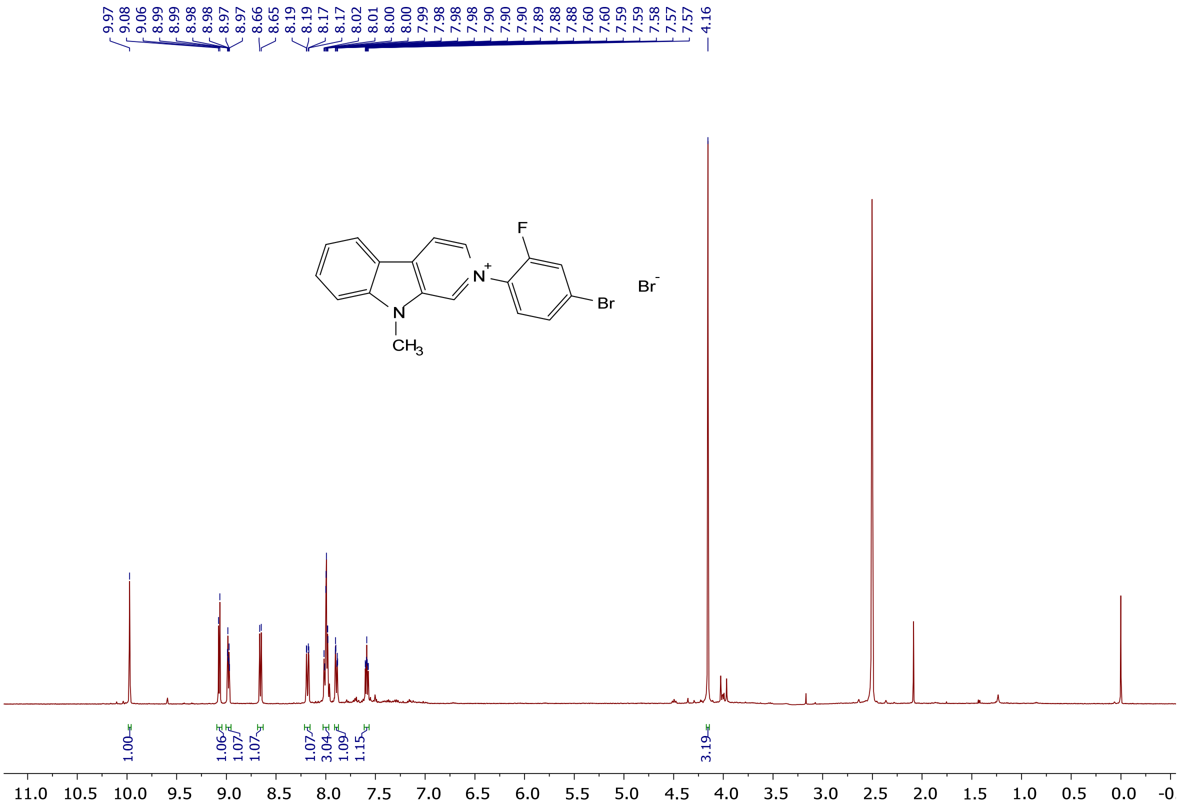

^{1}H NMR of Compound B**32**

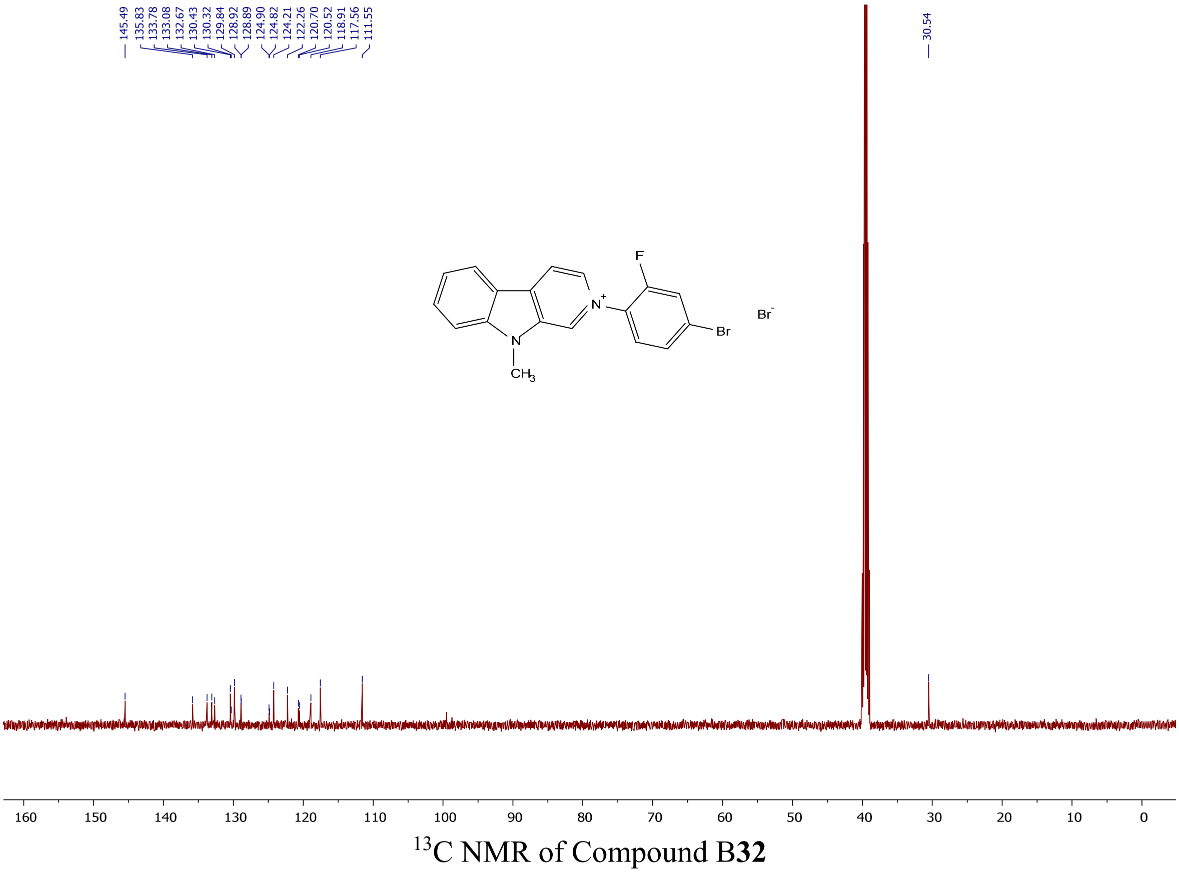

^{13}C NMR of Compound B**32**

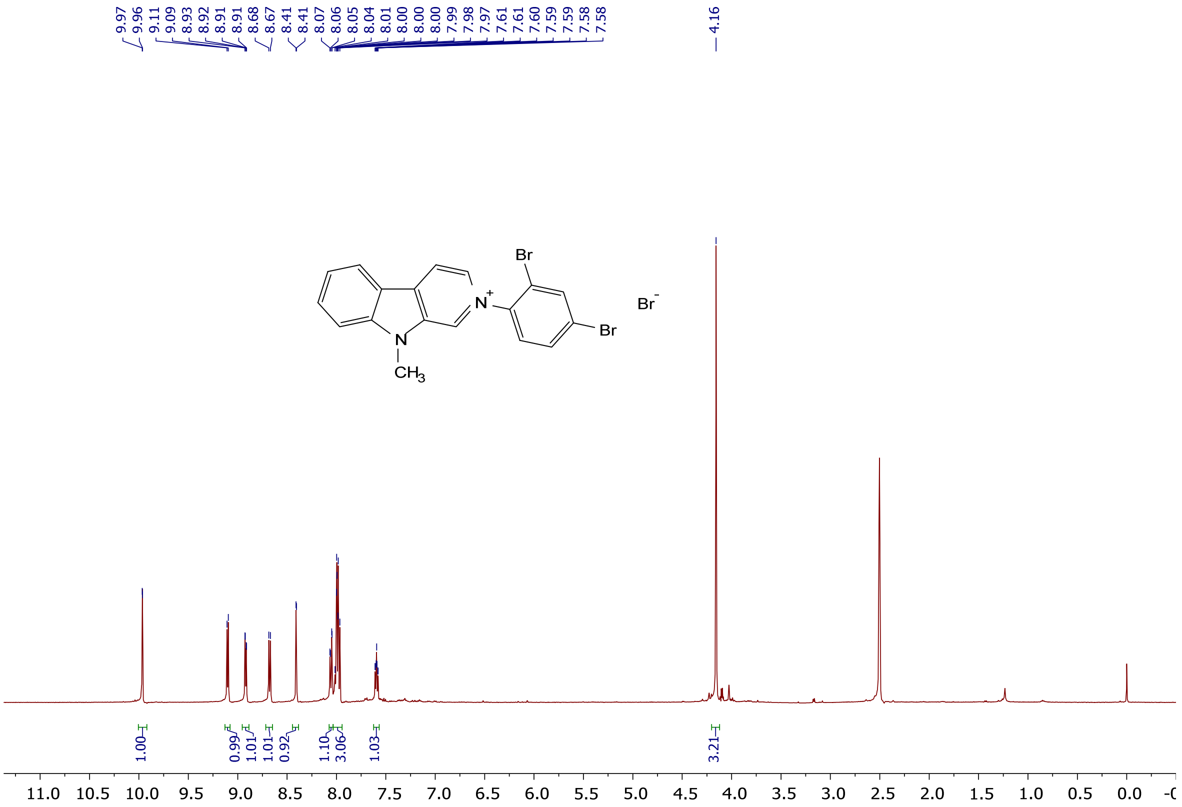

^{1}H NMR of Compound B**33**

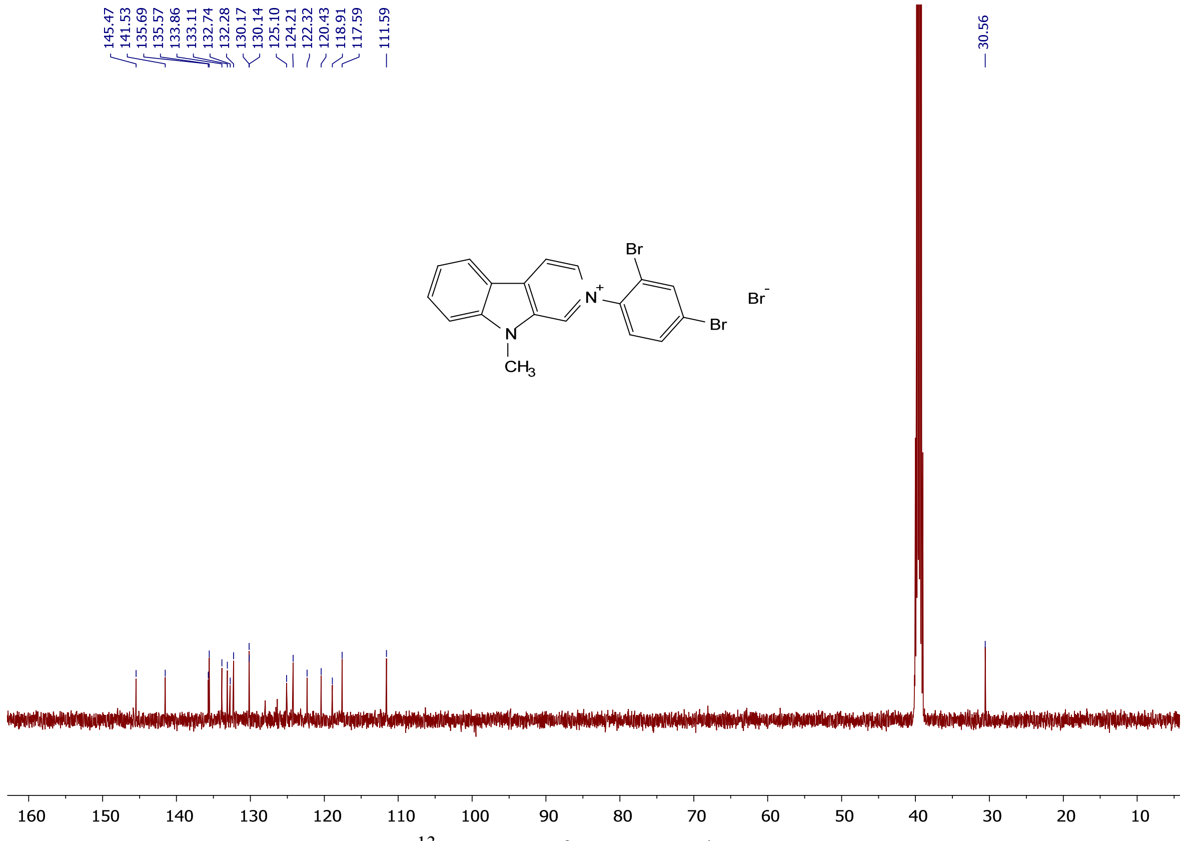

^{13}C NMR of Compound B**33**

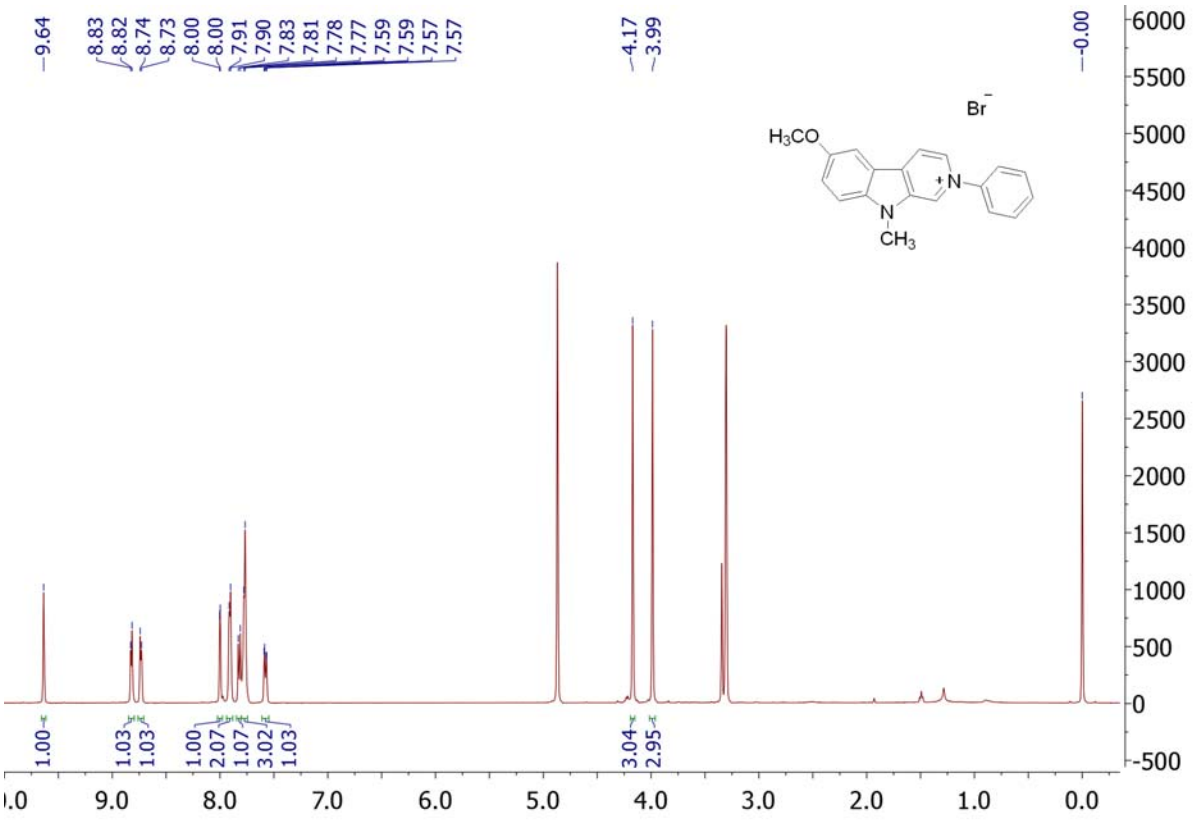

^{1}H NMR of Compound B**34**

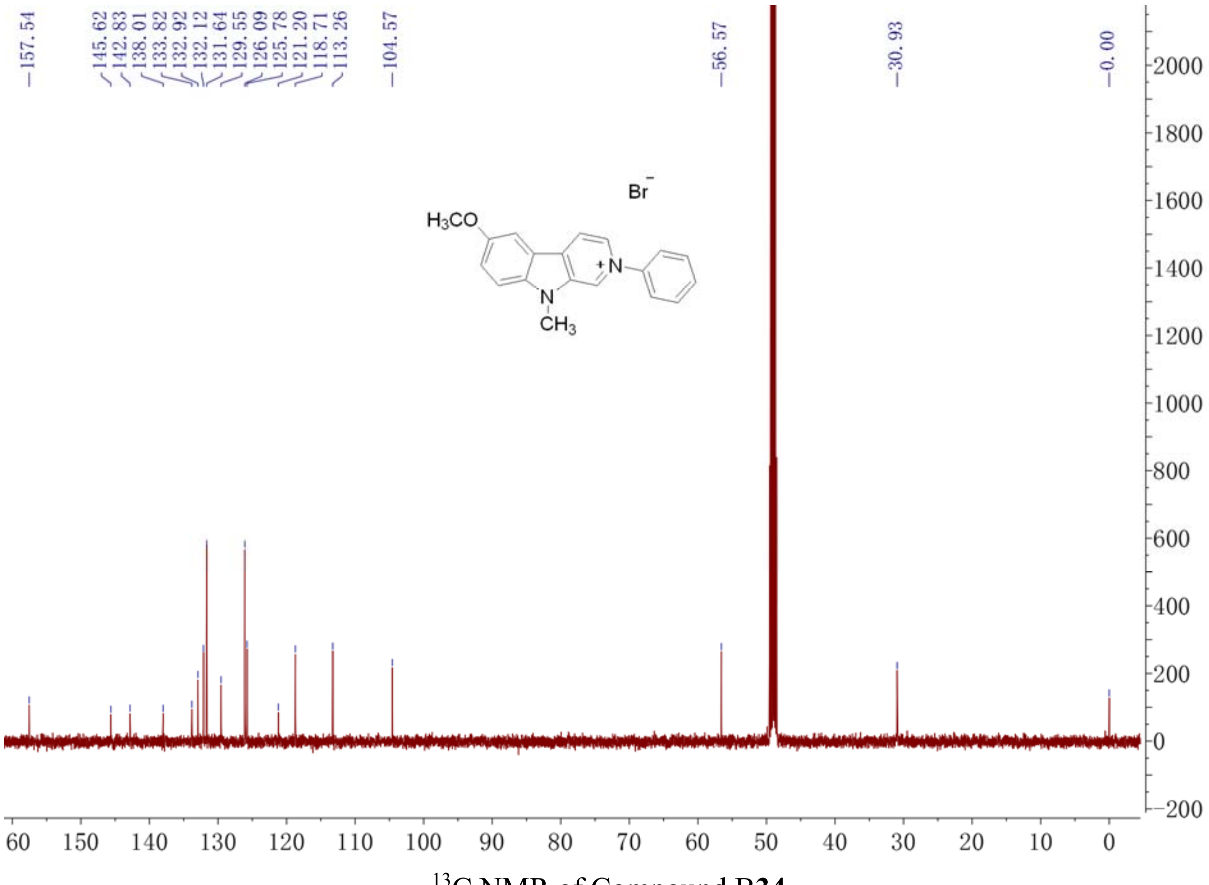

^{13}C NMR of Compound B**34**

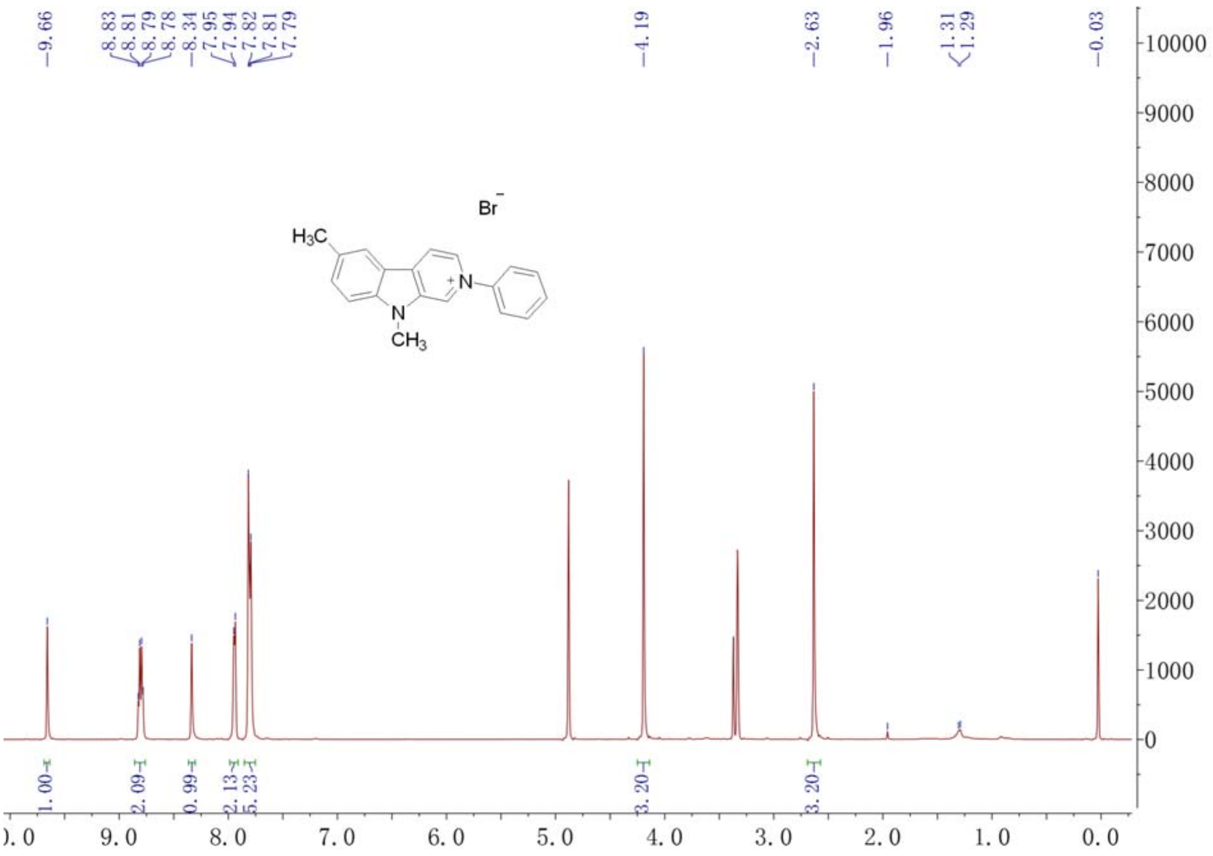

^{1}H NMR of Compound B**35**

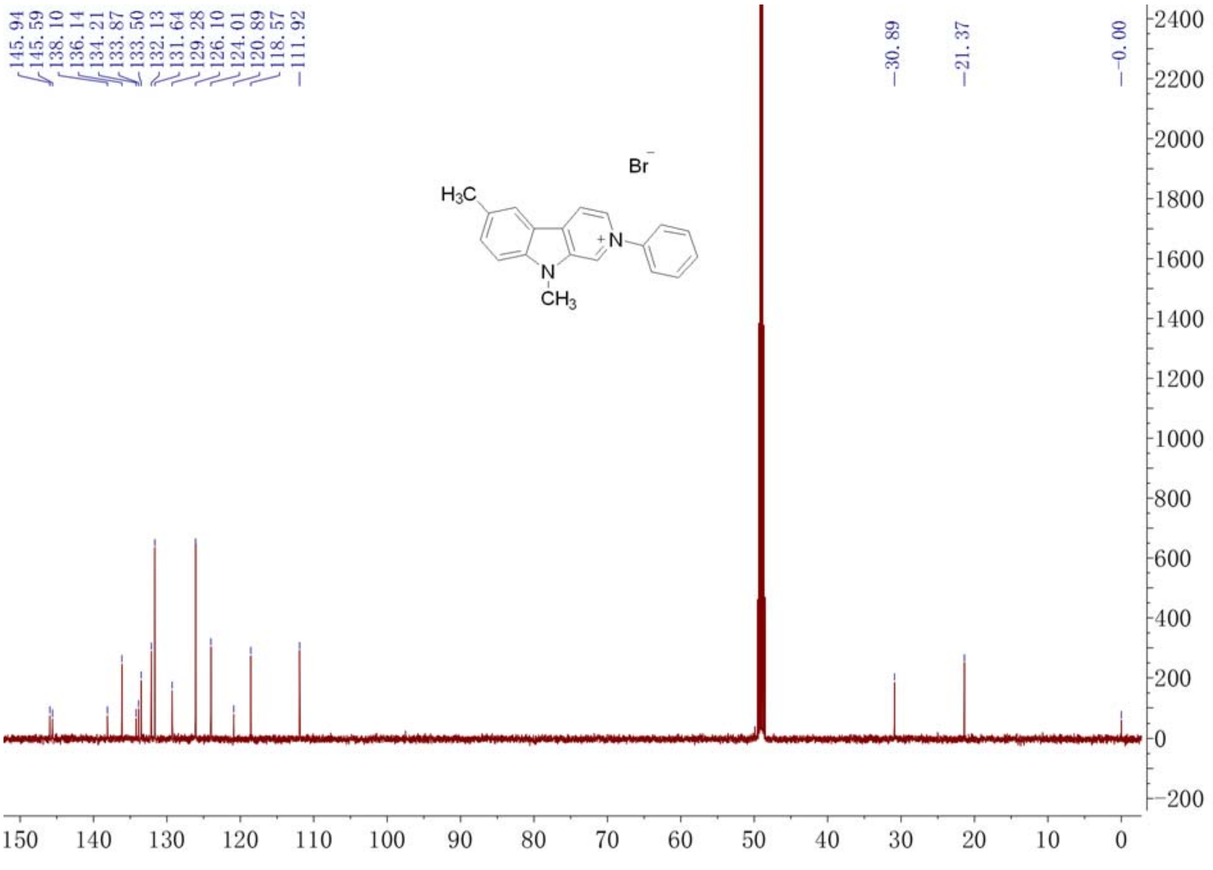

^{13}C NMR of Compound B**35**

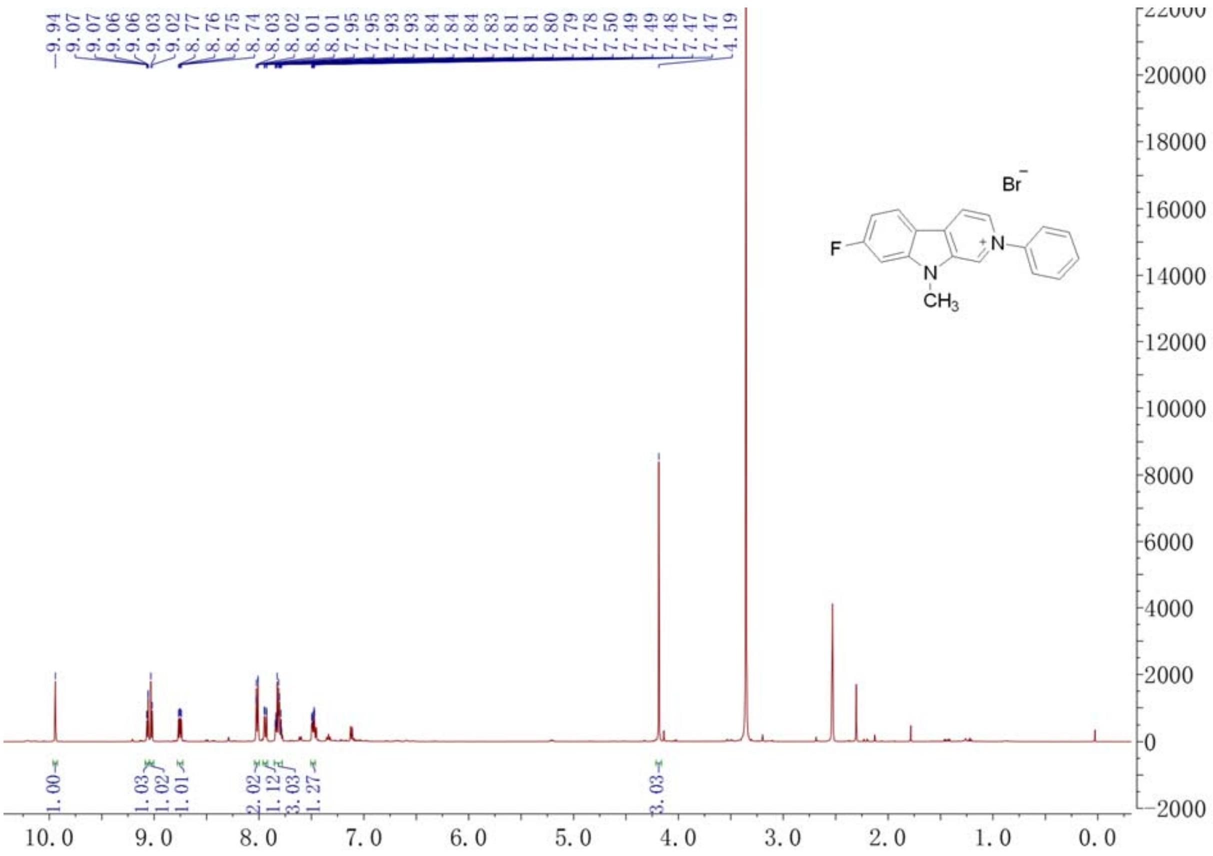

^1H NMR of Compound B**36**

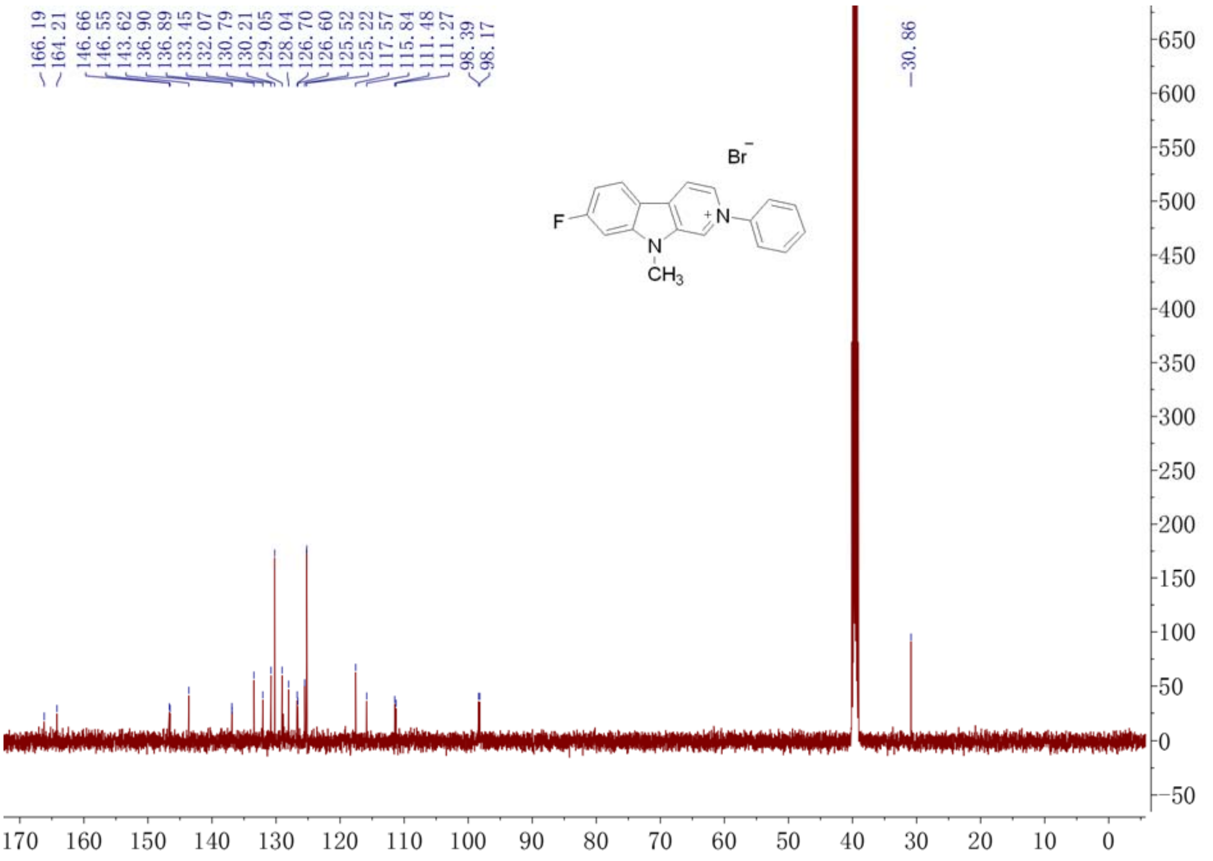

^{13}C NMR of Compound B**36**

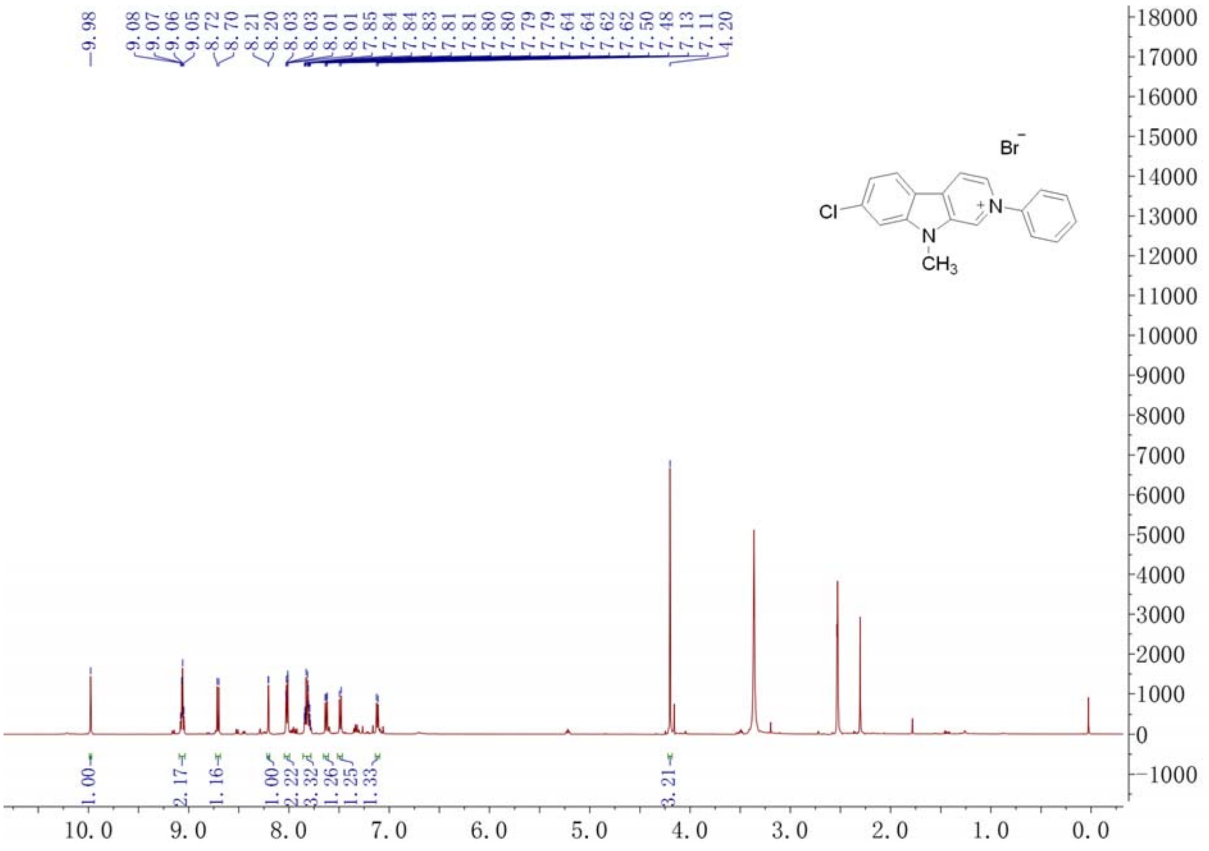

^{1}H NMR of Compound B**37**

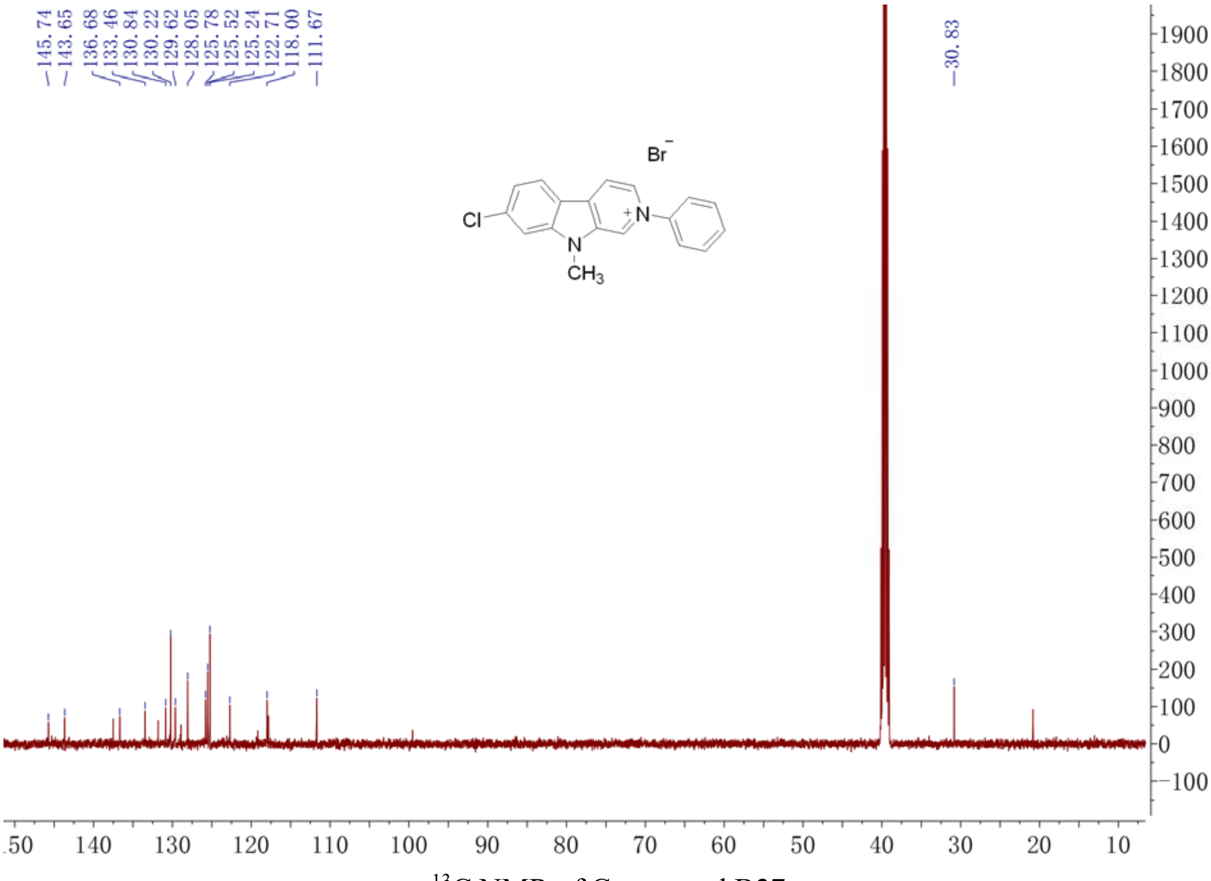

^{13}C NMR of Compound B**37**

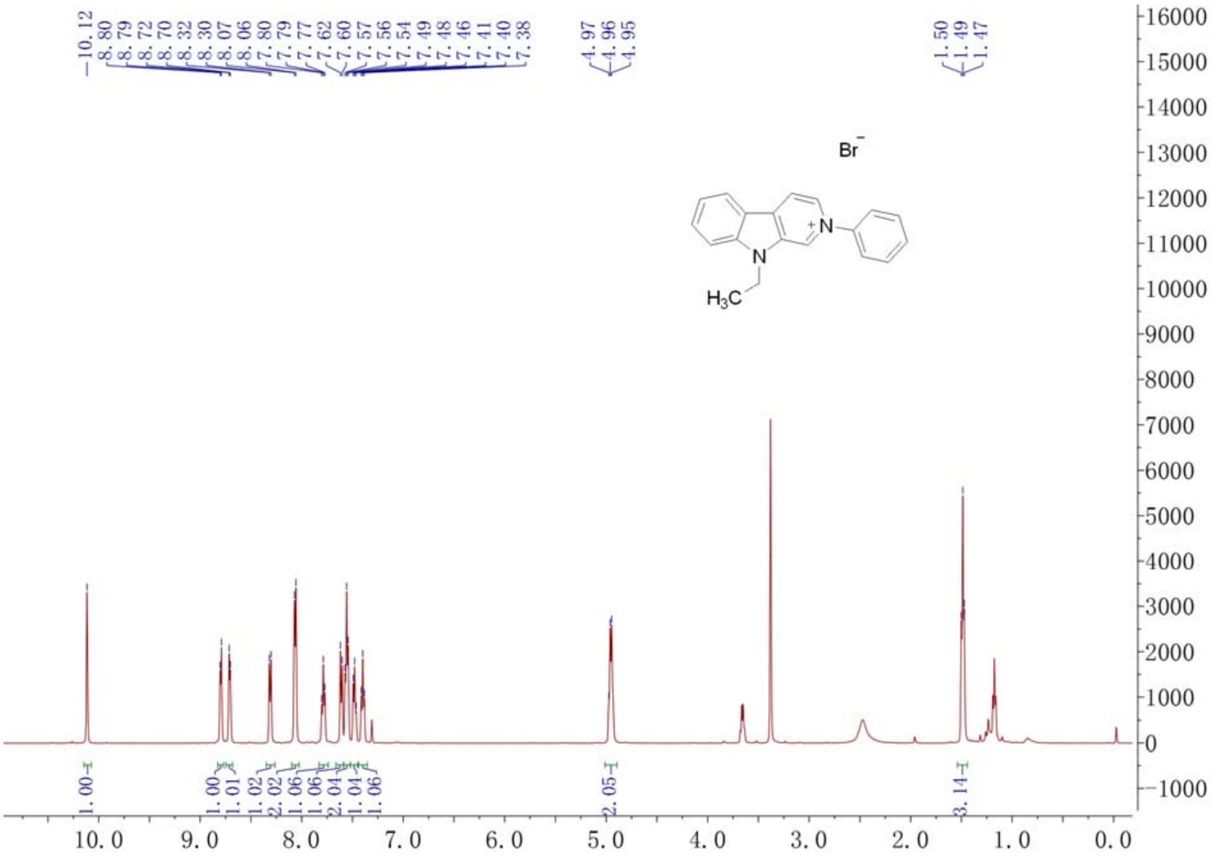

^{1}H NMR of Compound B**38**

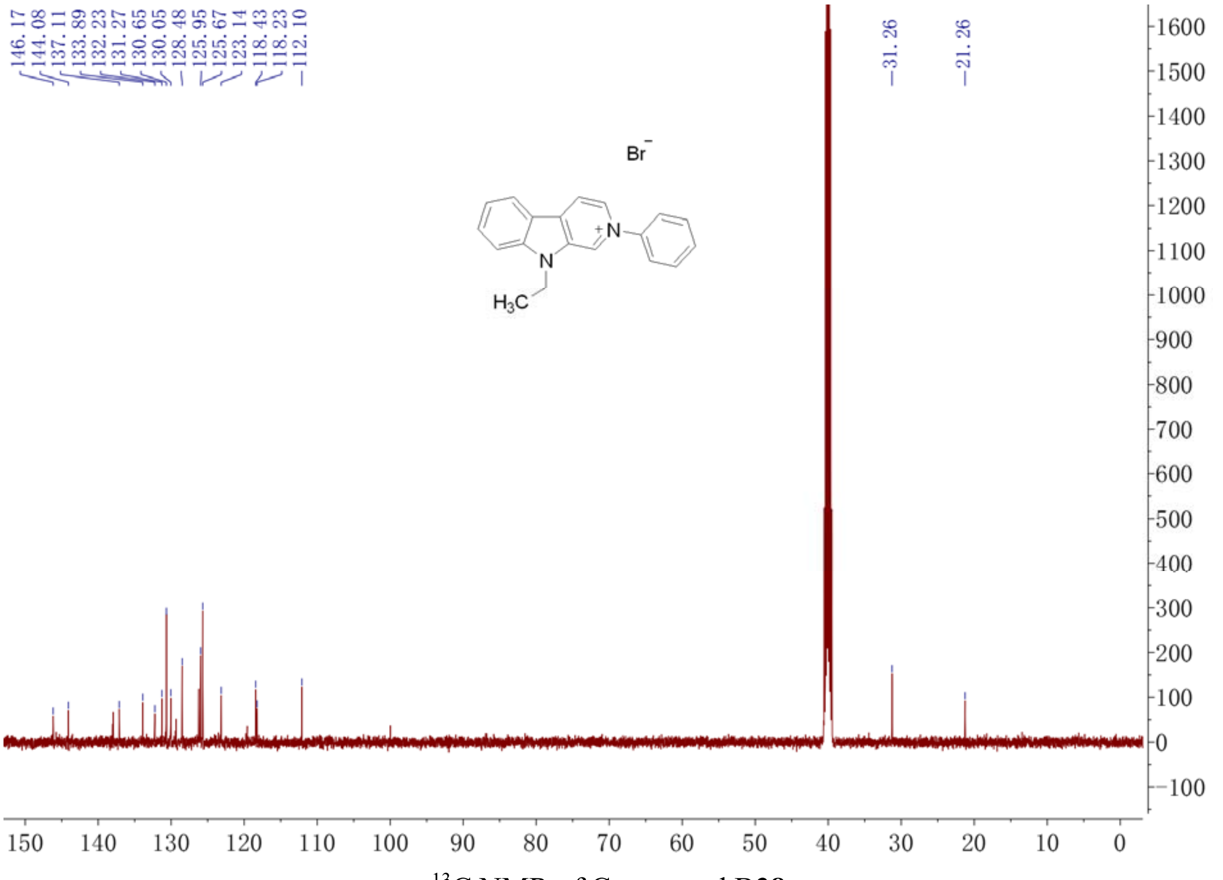

^{13}C NMR of Compound B**38**

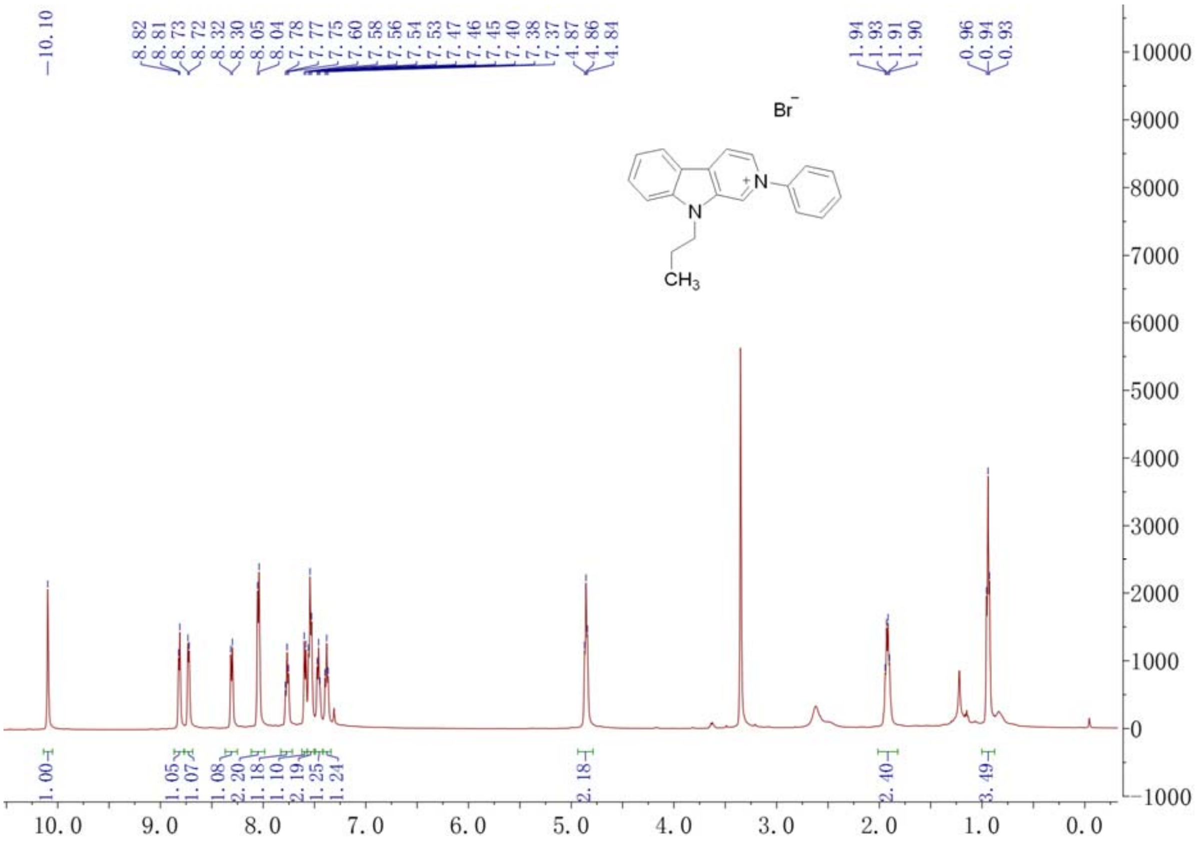

^{1}H NMR of Compound B**39**

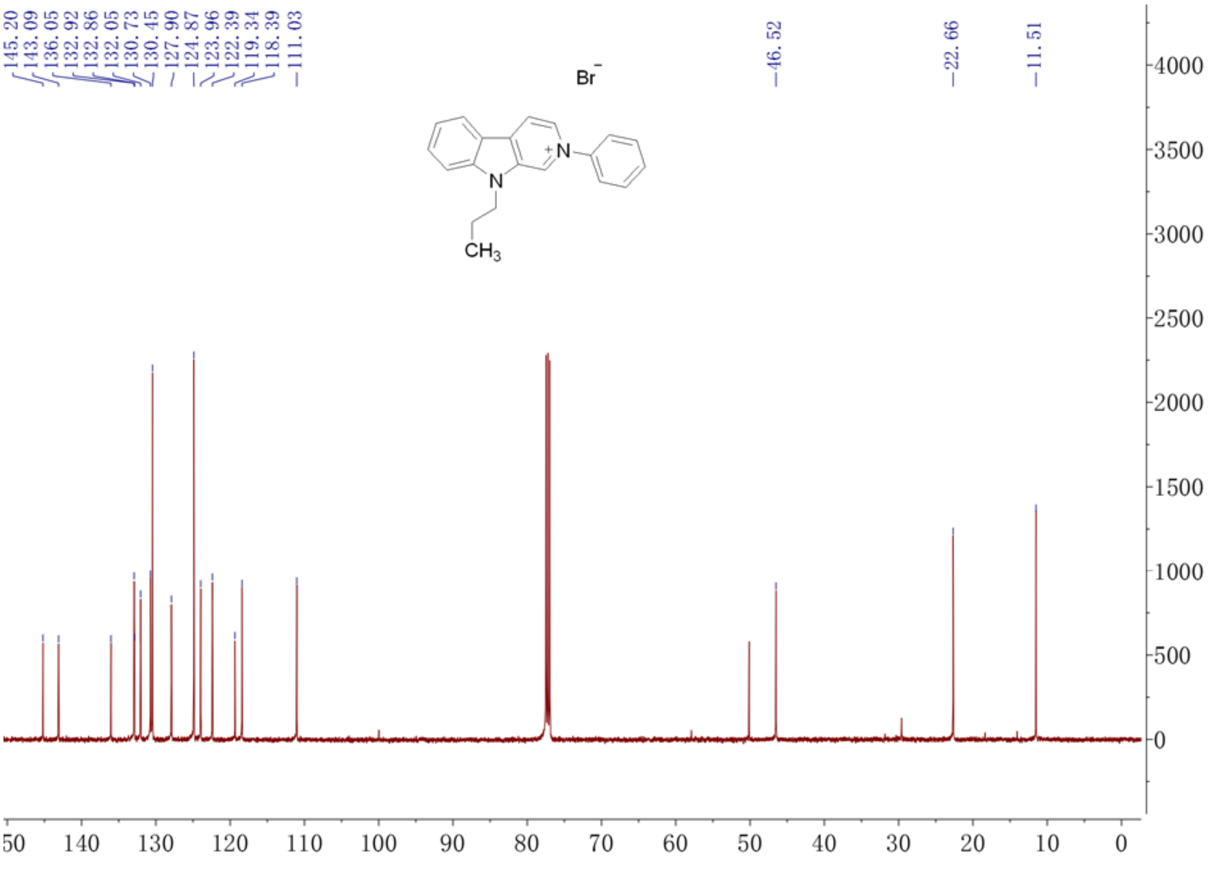

^{13}C NMR of Compound B**39**

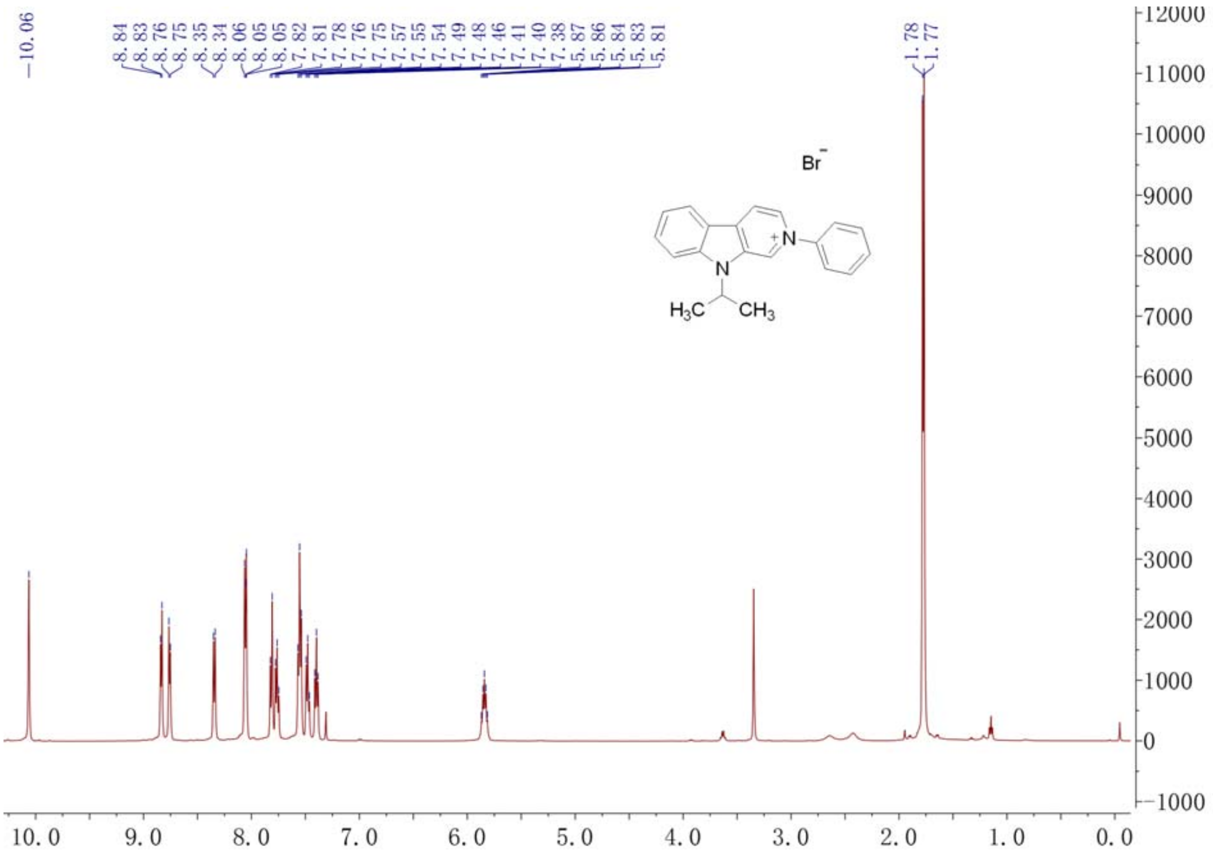

^{1}H NMR of Compound B**40**

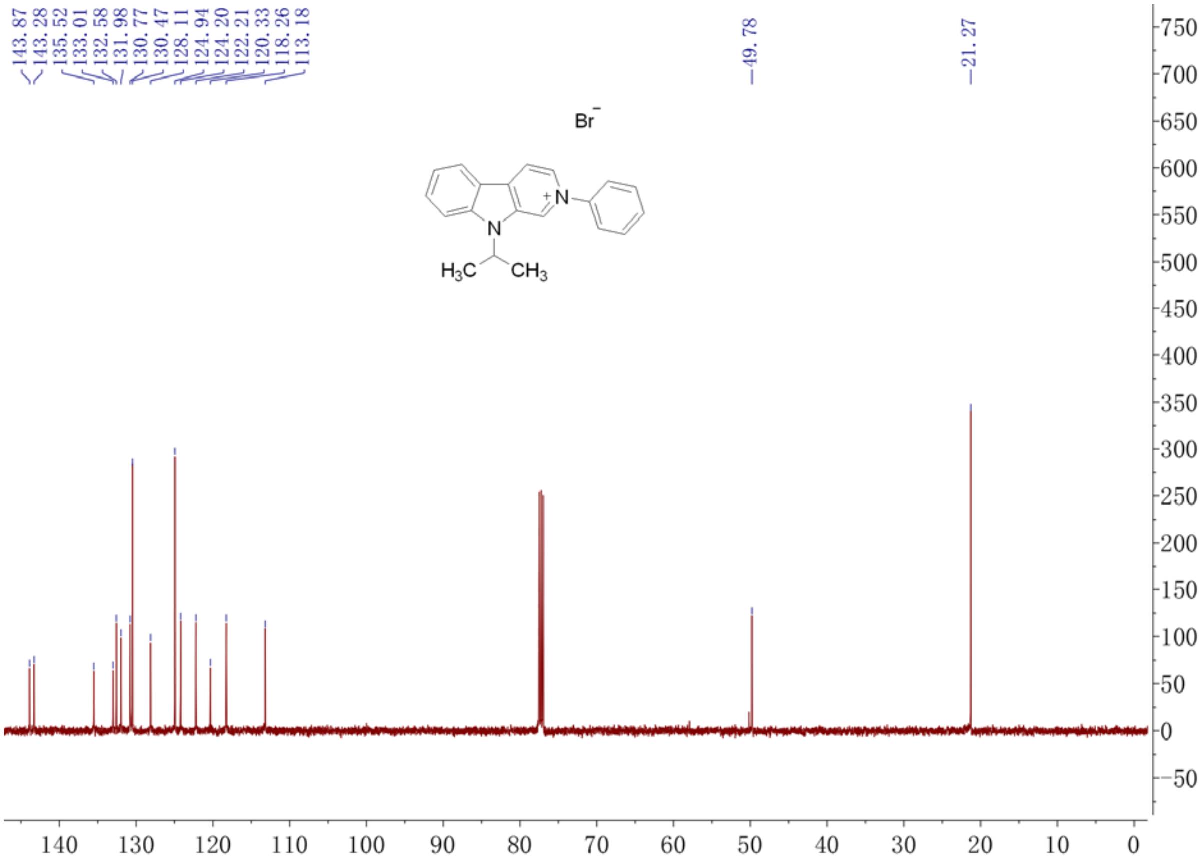

^{13}C NMR of Compound B**40**

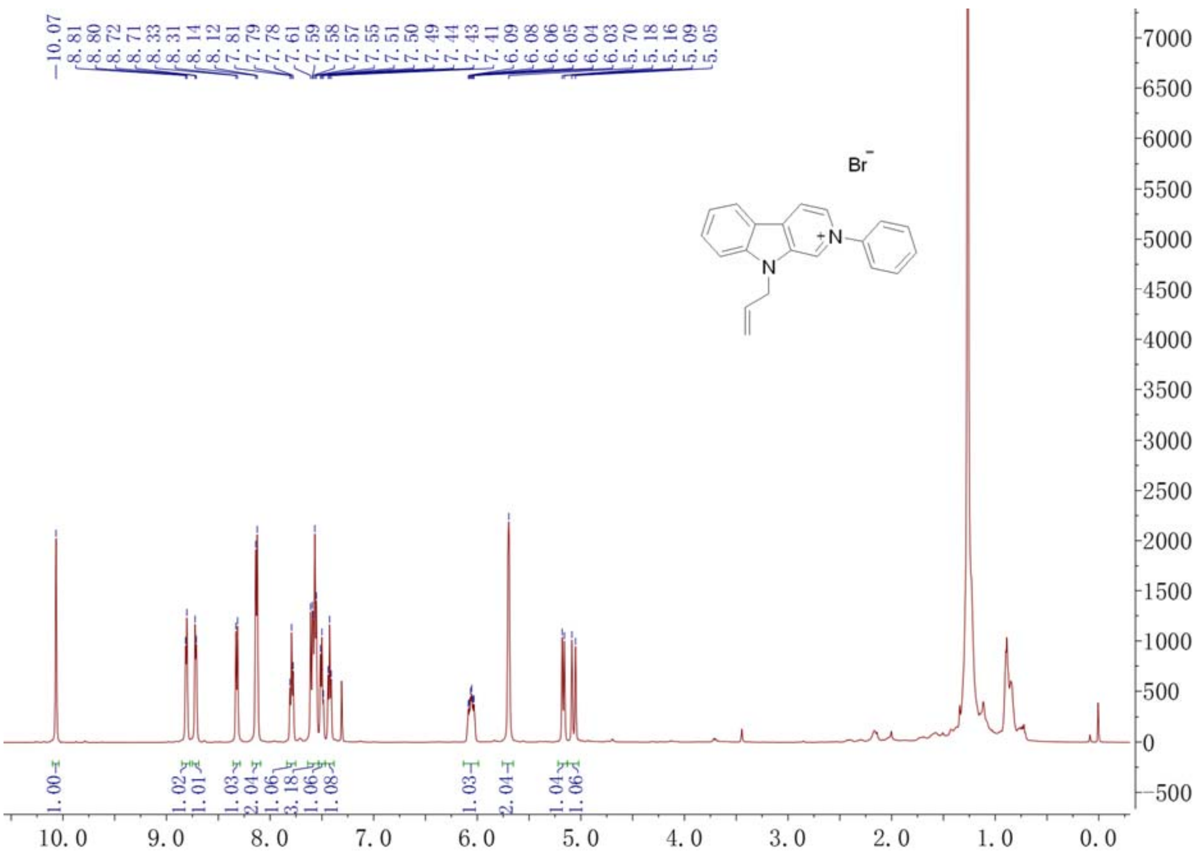

^{1}H NMR of Compound B**41**

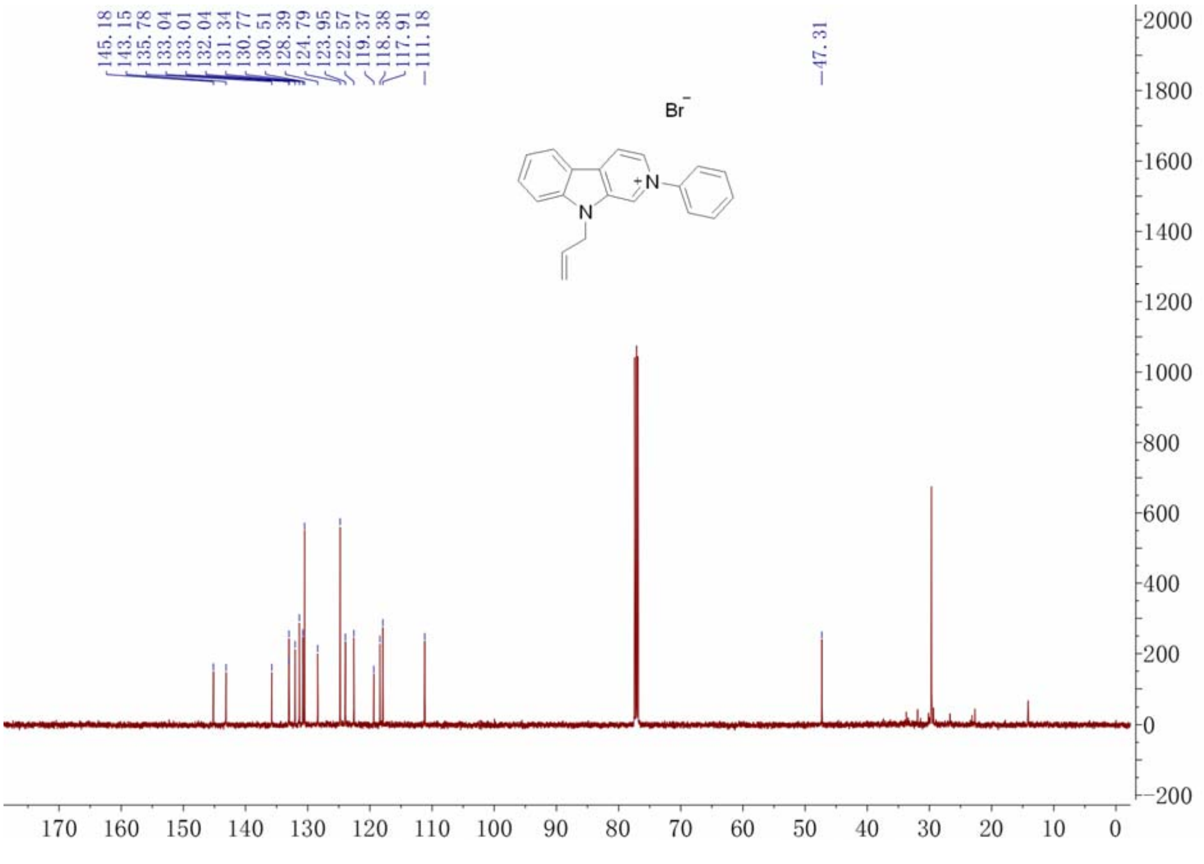

^{13}C NMR of Compound B**41**

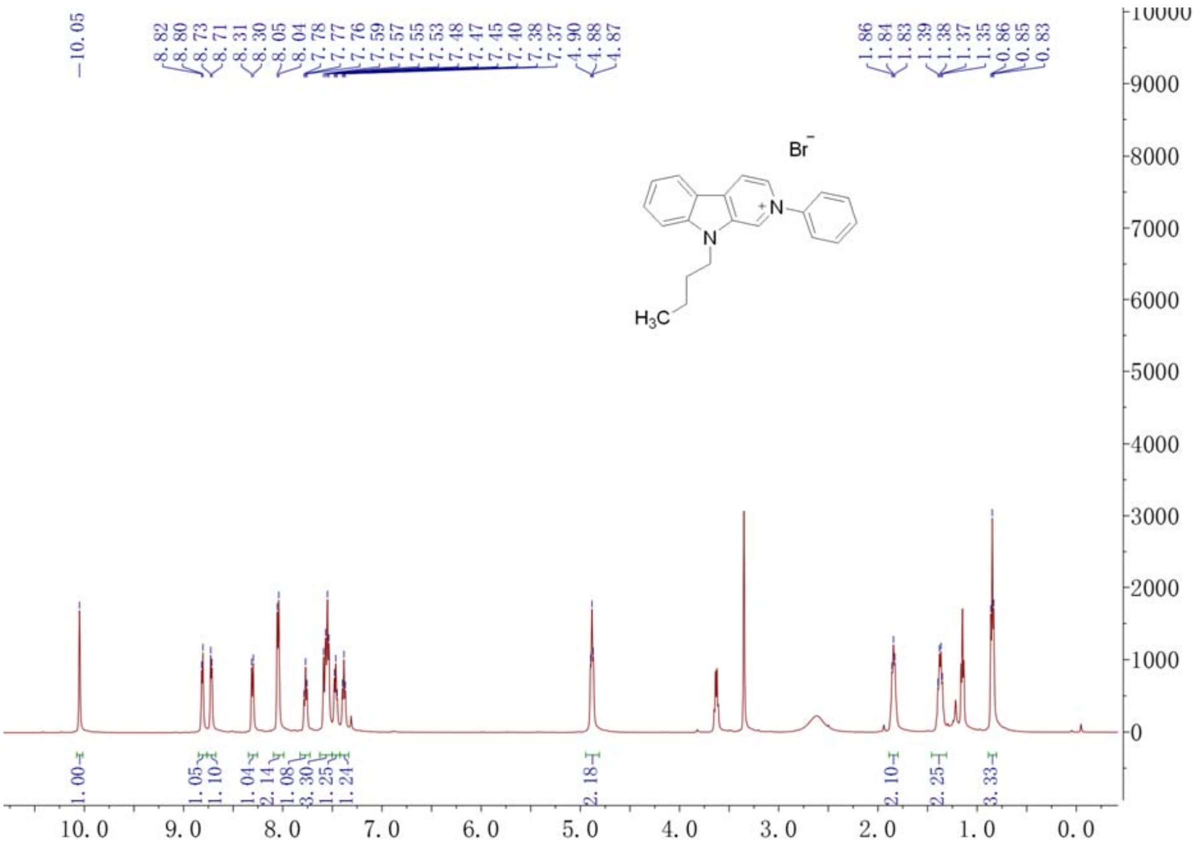

^{1}H NMR of Compound B**42**

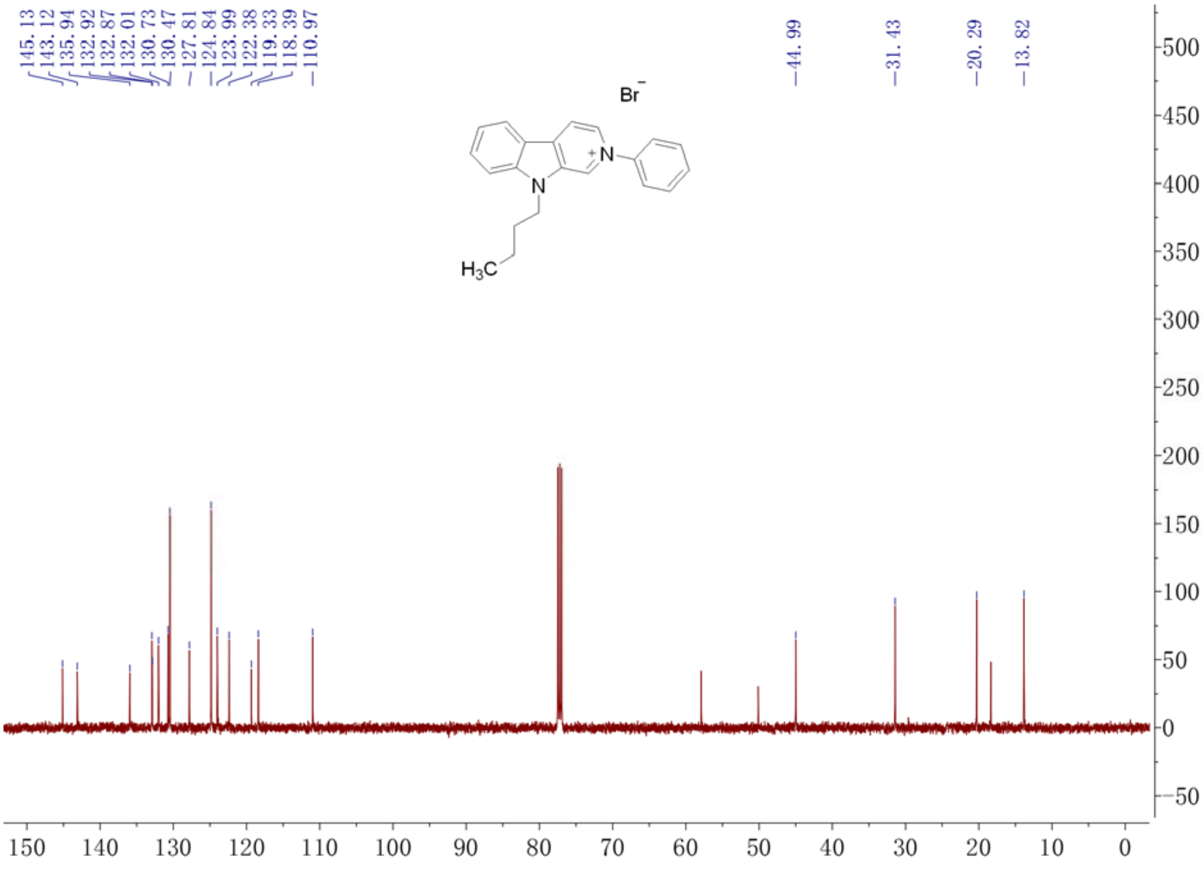

^{13}C NMR of Compound B**42**

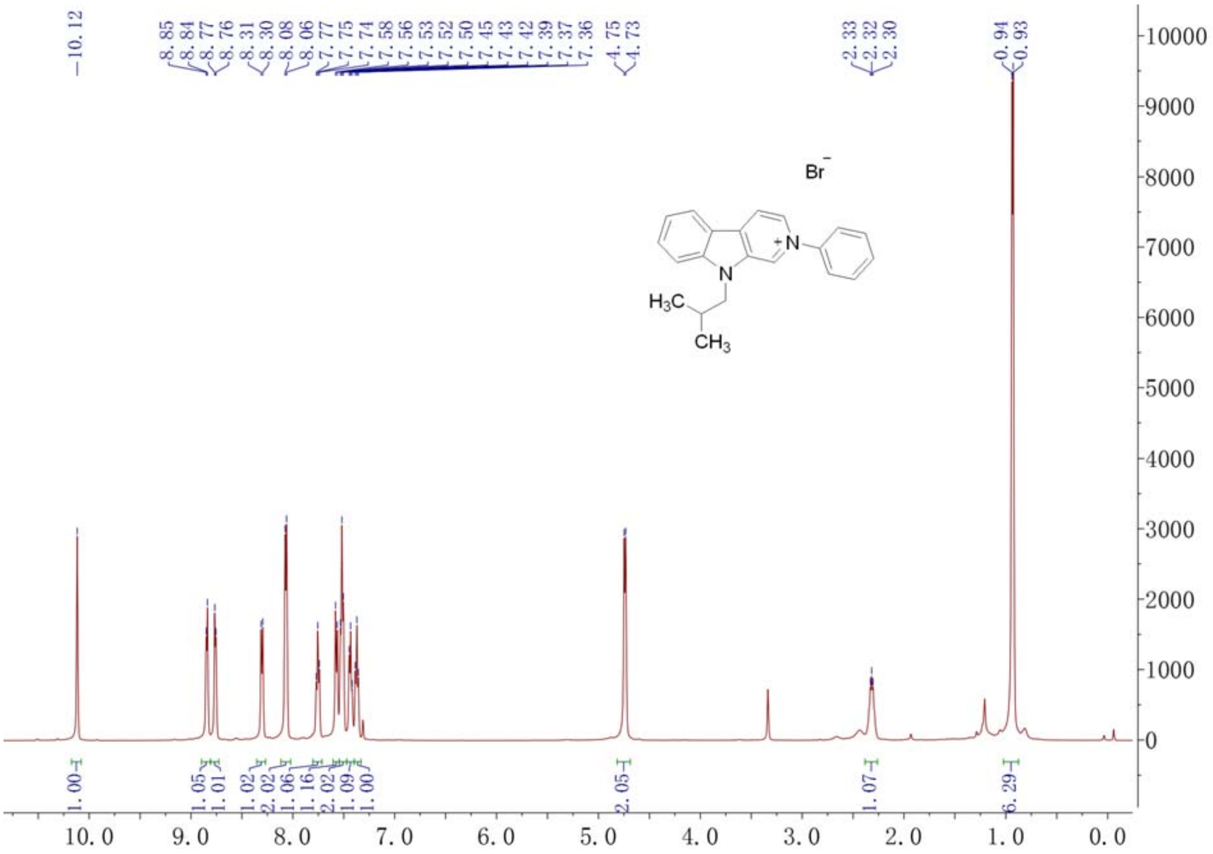

^{1}H NMR of Compound B**43**

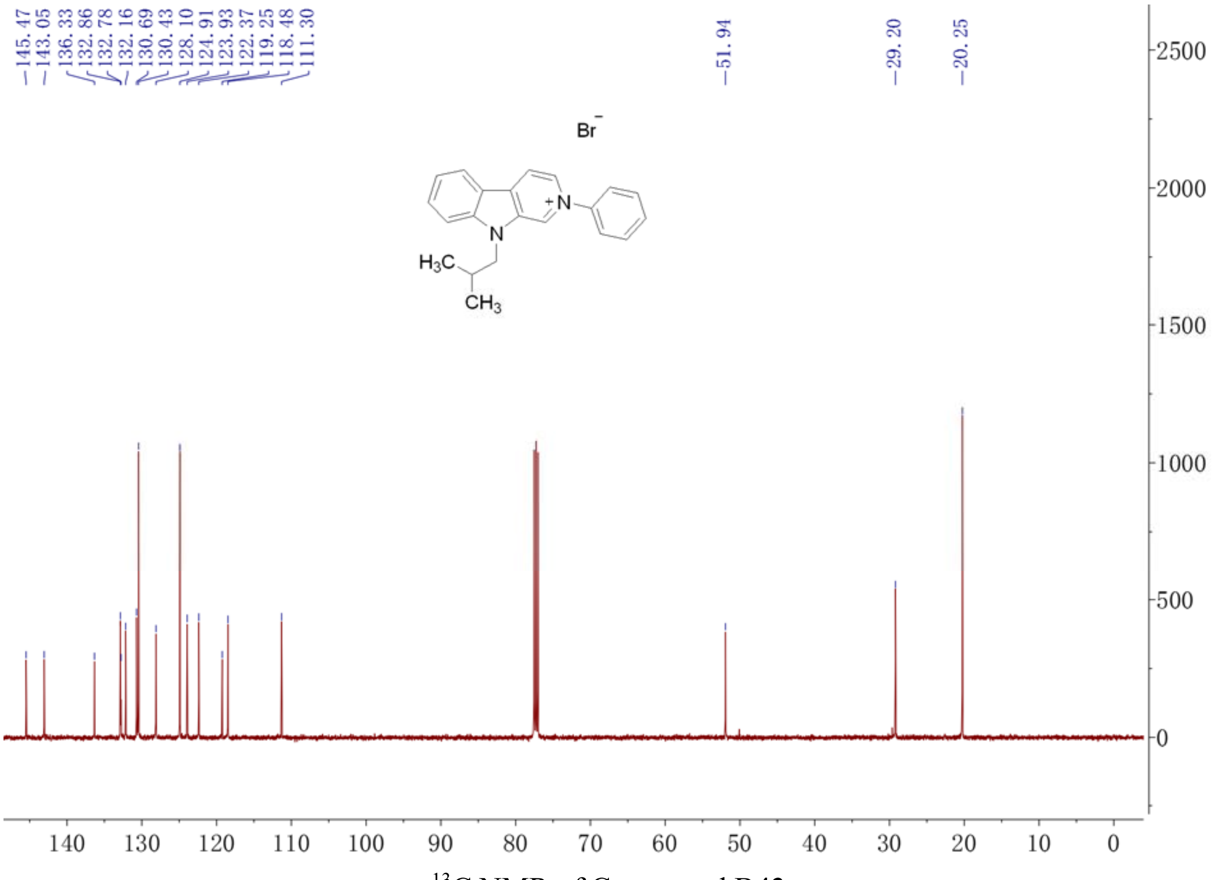

^{13}C NMR of Compound B**43**

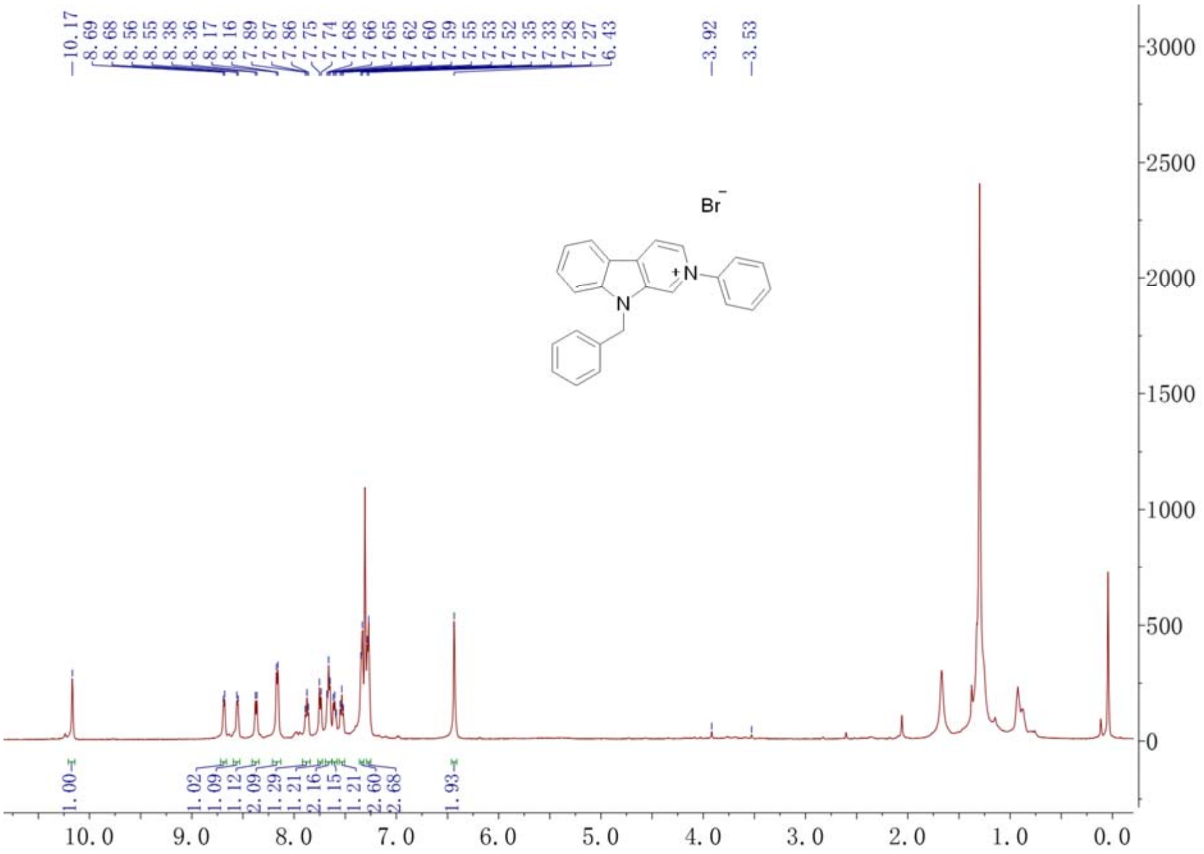

^{1}H NMR of Compound B**44**

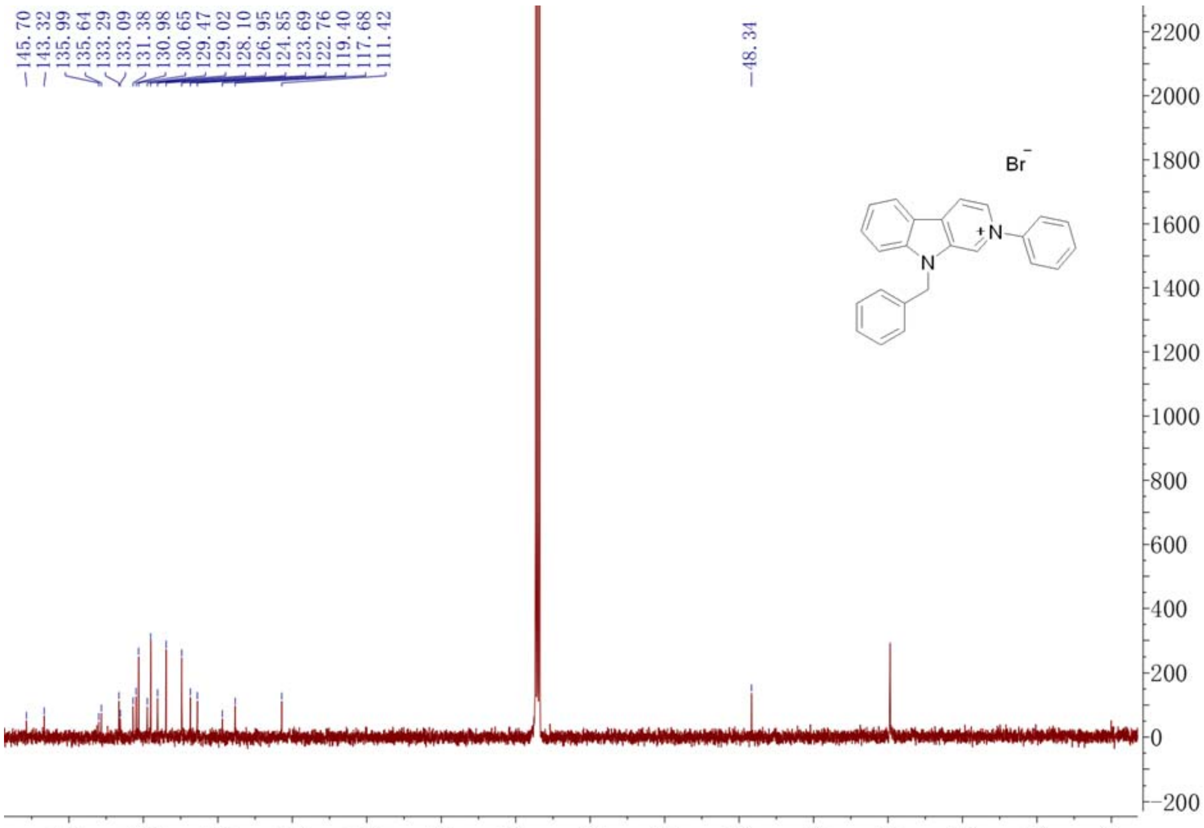

^{13}C NMR of Compound B**44**

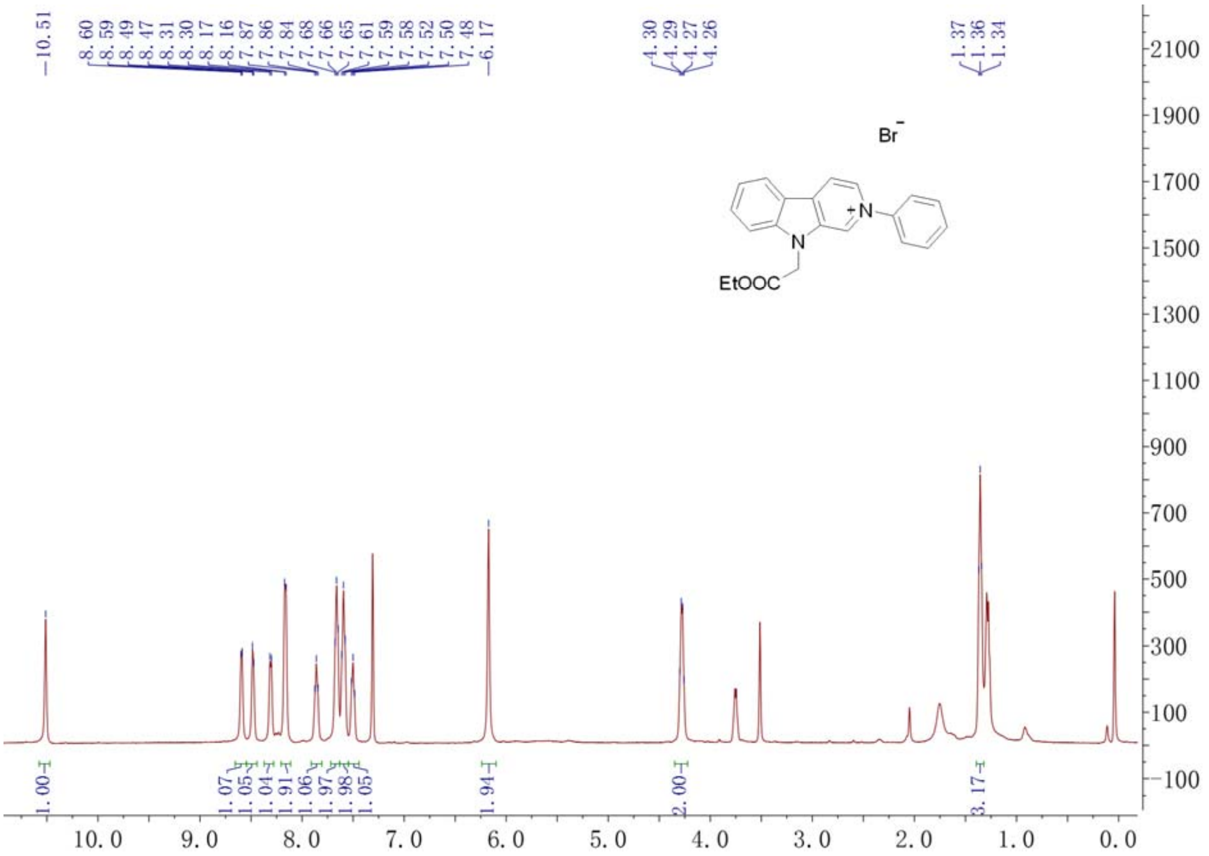

^{1}H NMR of Compound B**45**

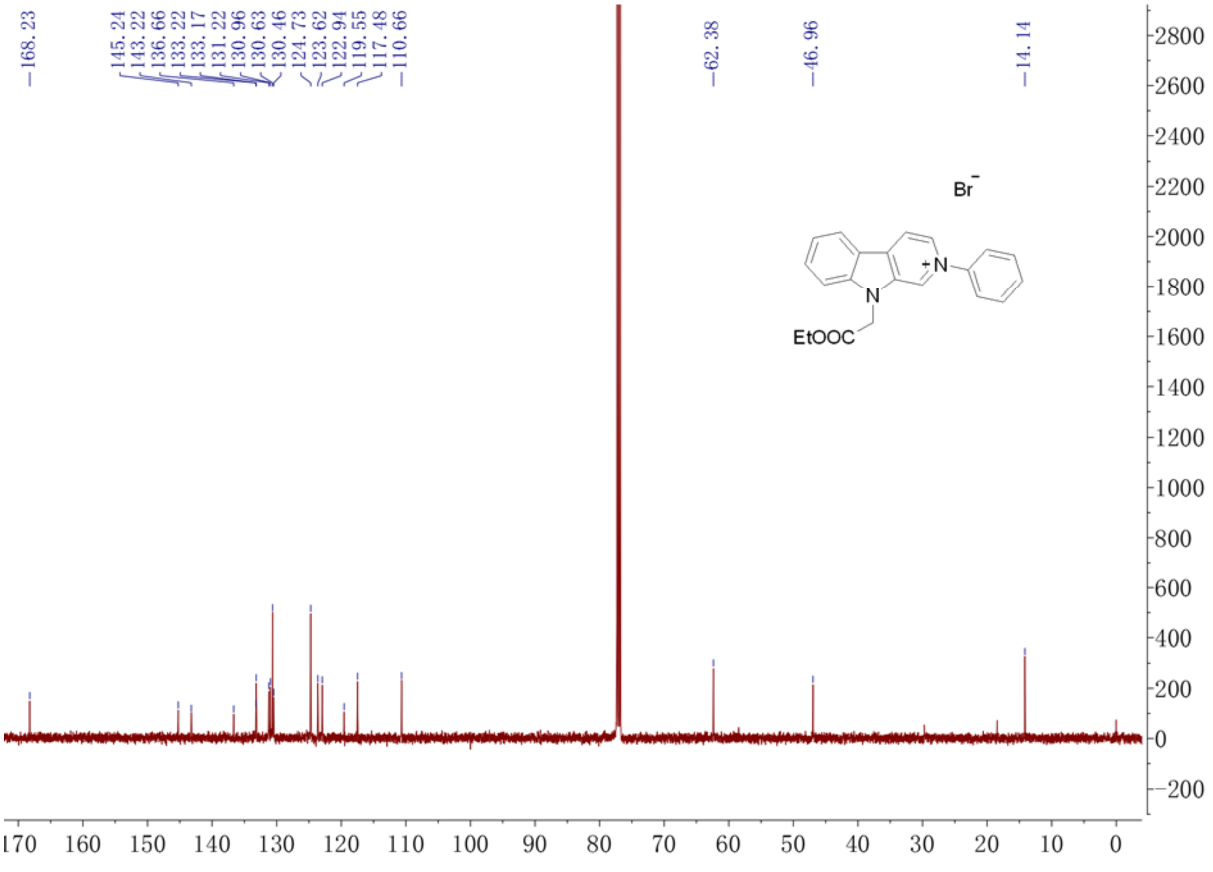

^{13}C NMR of Compound B**45**

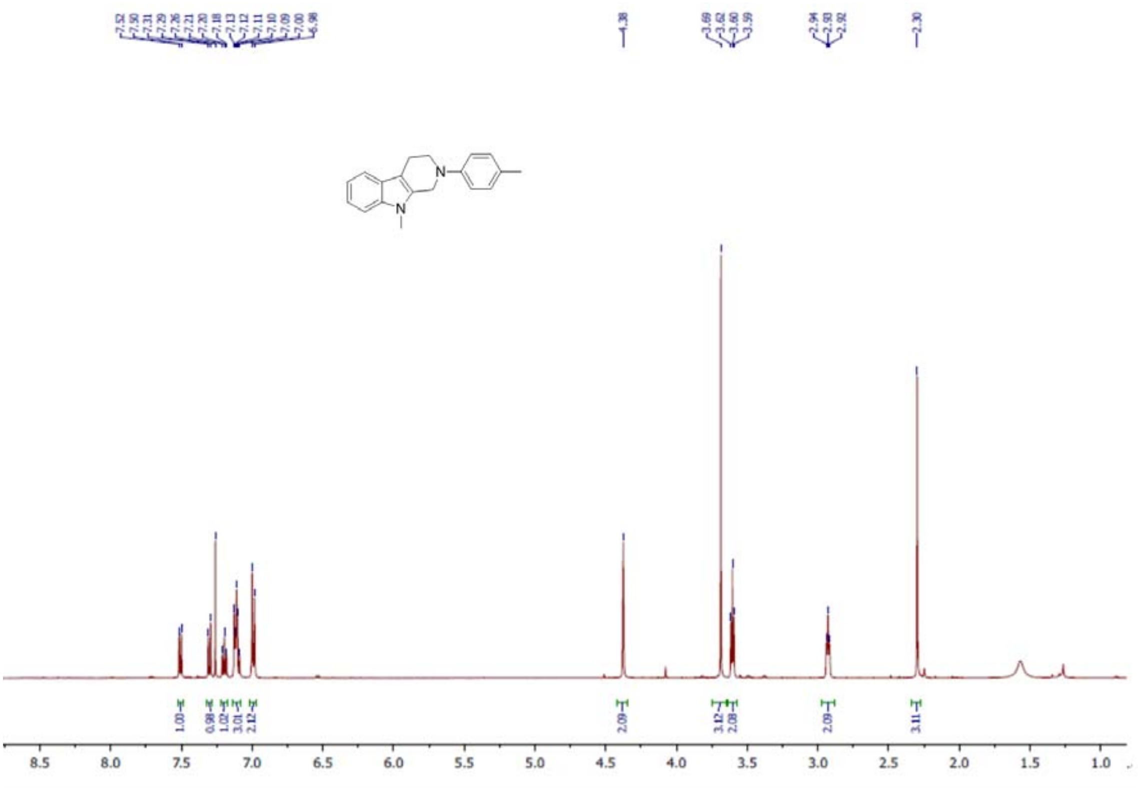

^{1}H NMR of Compound **C1**

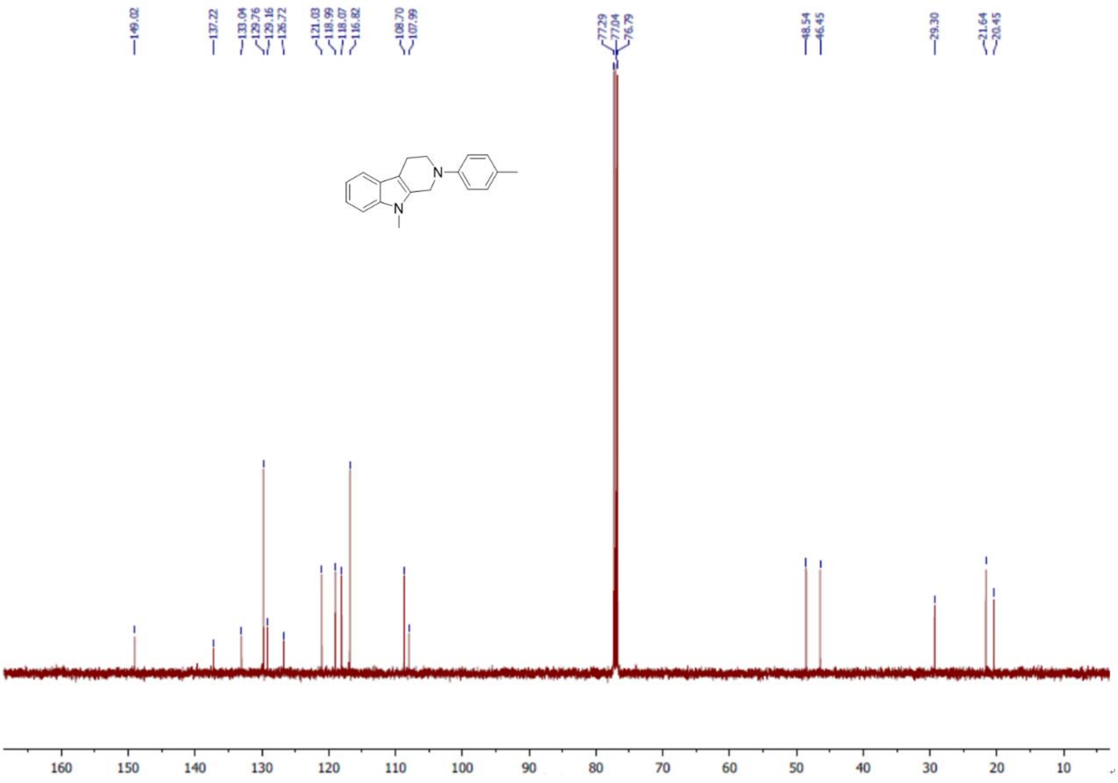

^{13}C NMR of Compound **C1**

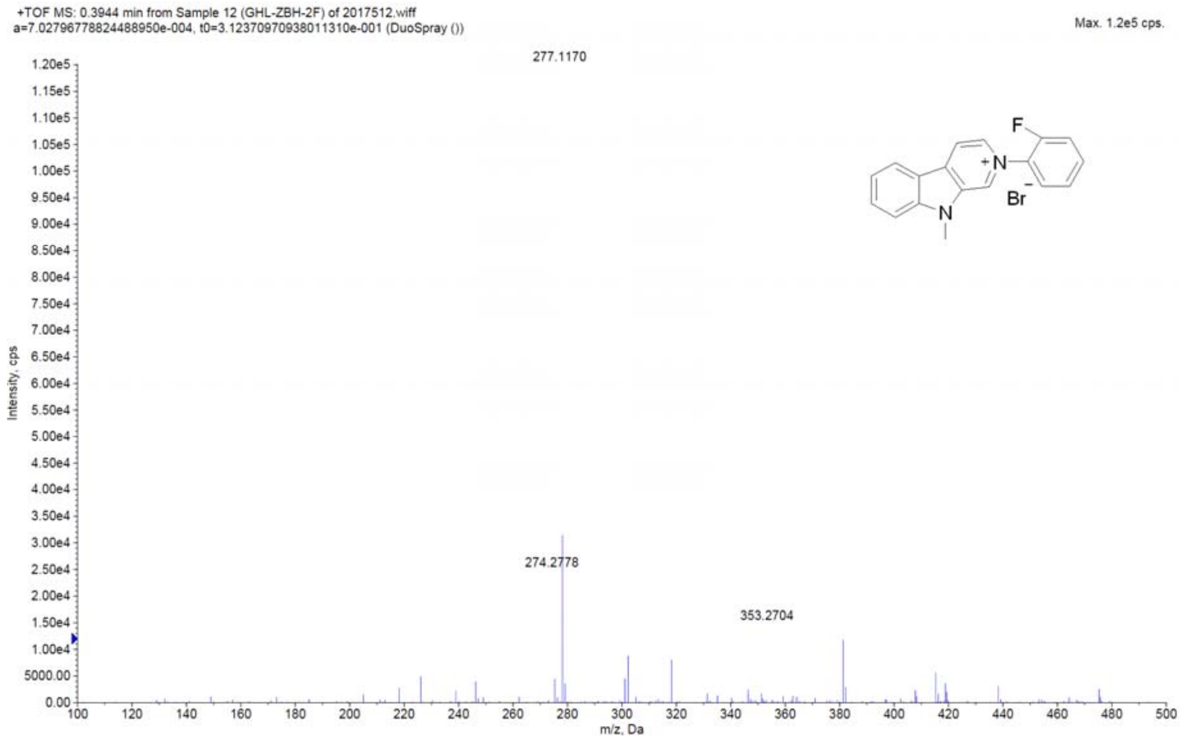

HR-ESI-MS of Compound B**2**

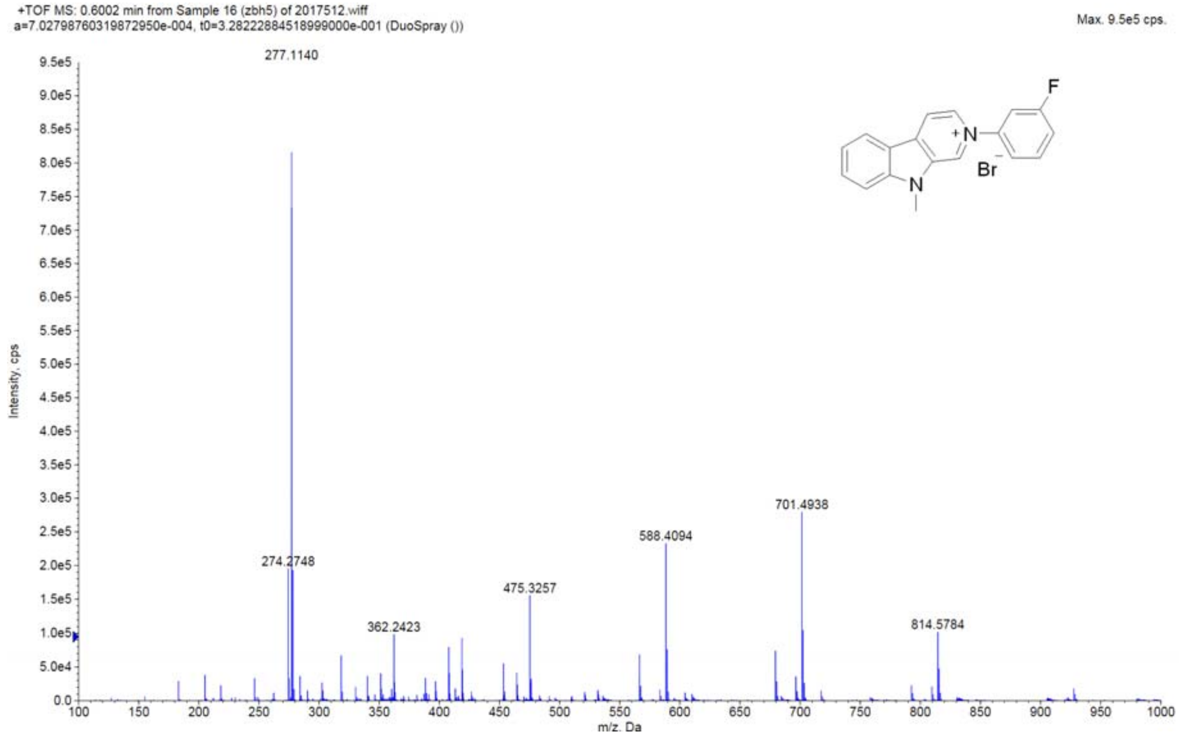

HR-ESI-MS of Compound B**3**

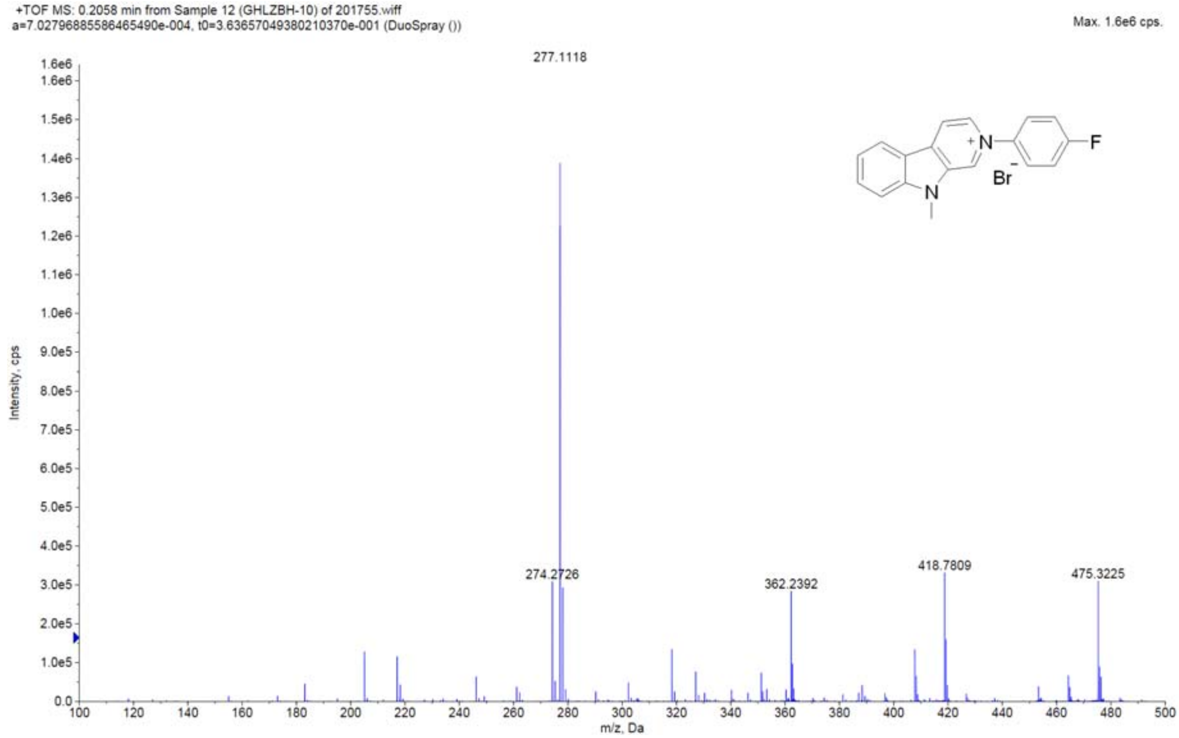

HR-ESI-MS of Compound B**4**

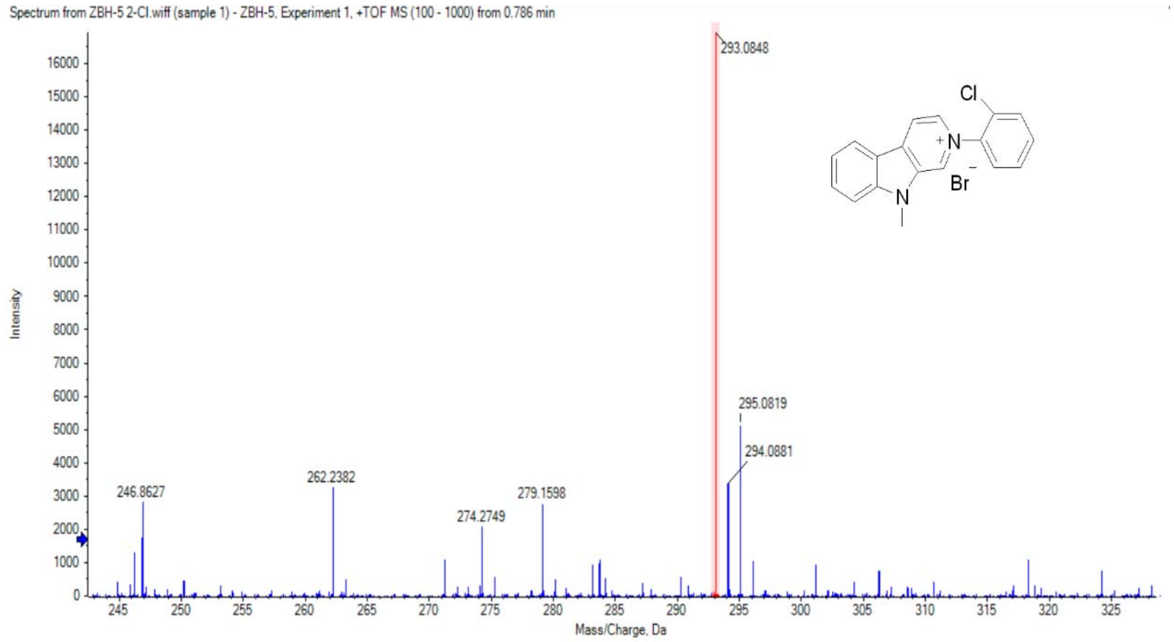

HR-ESI-MS of Compound B**5**

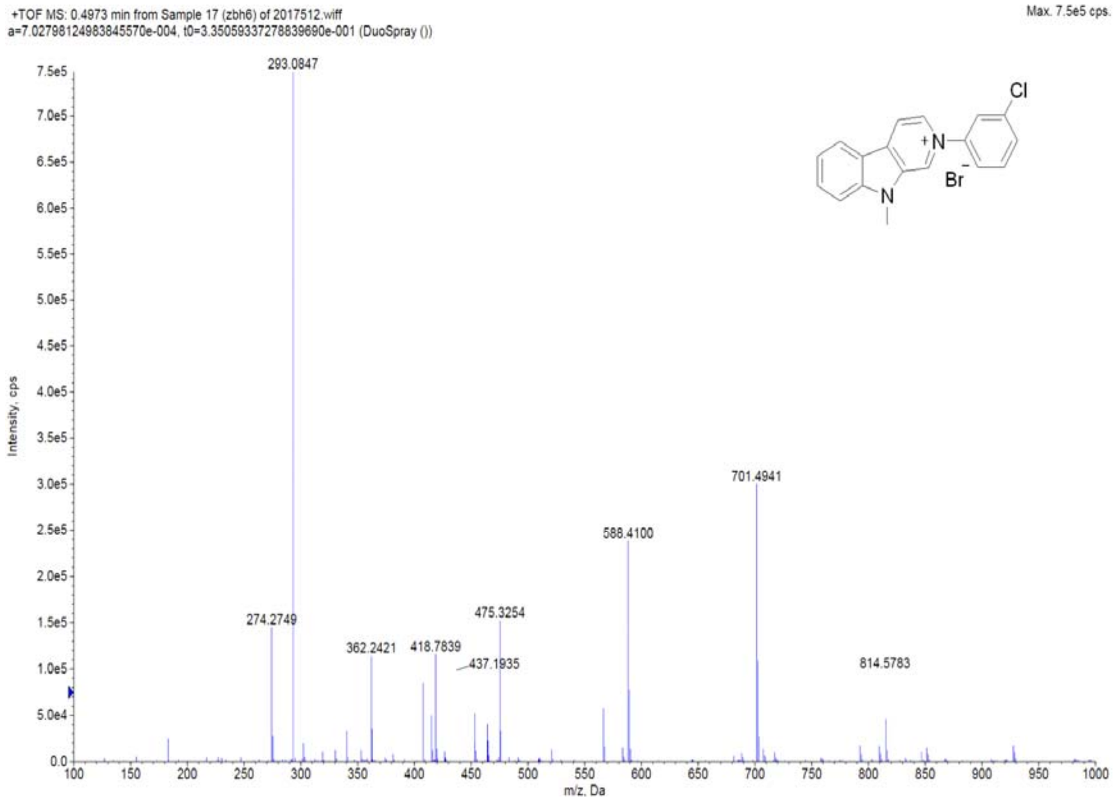

HR-ESI-MS of Compound B**6**

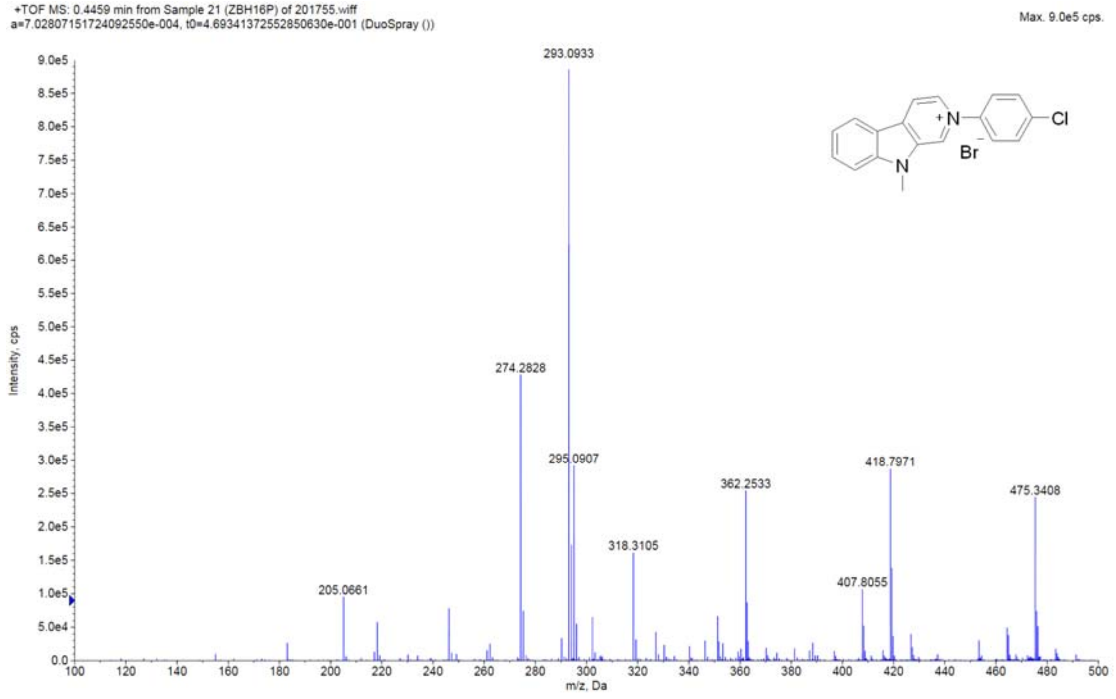

HR-ESI-MS of Compound B**7**

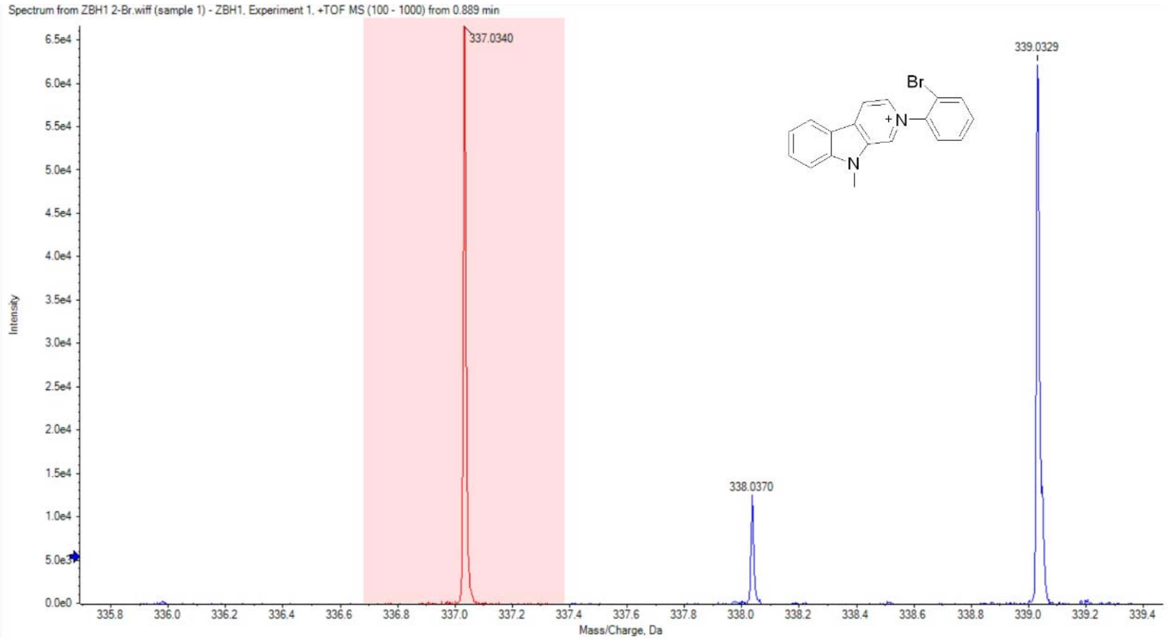

HR-ESI-MS of Compound B**8**

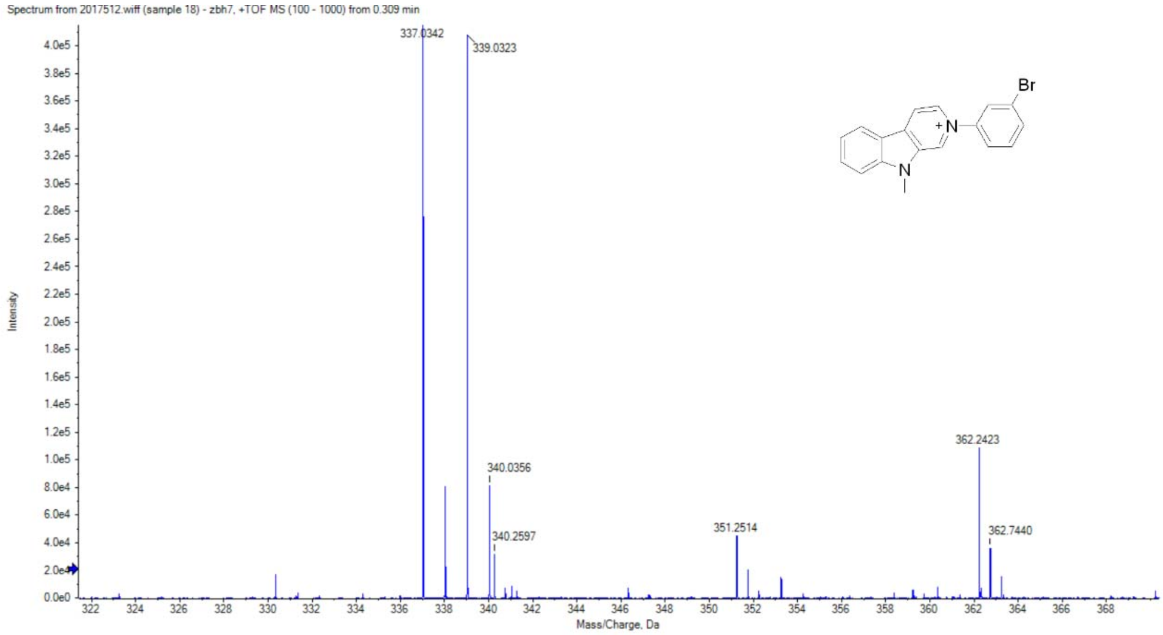

HR-ESI-MS of Compound B**9**

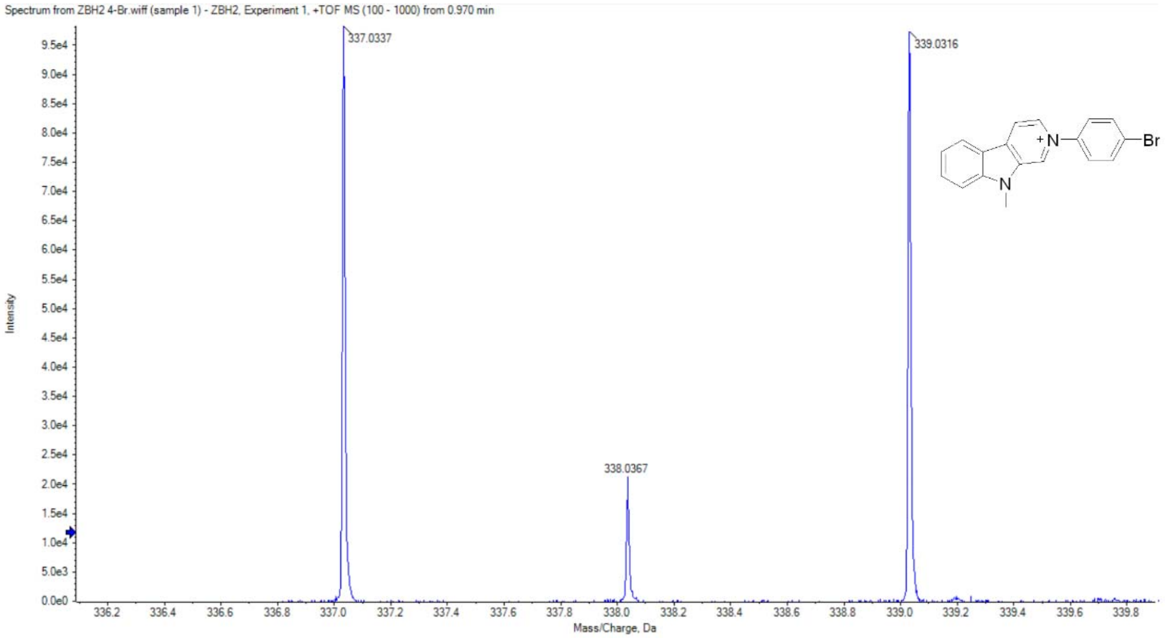

HR-ESI-MS of Compound B**10**

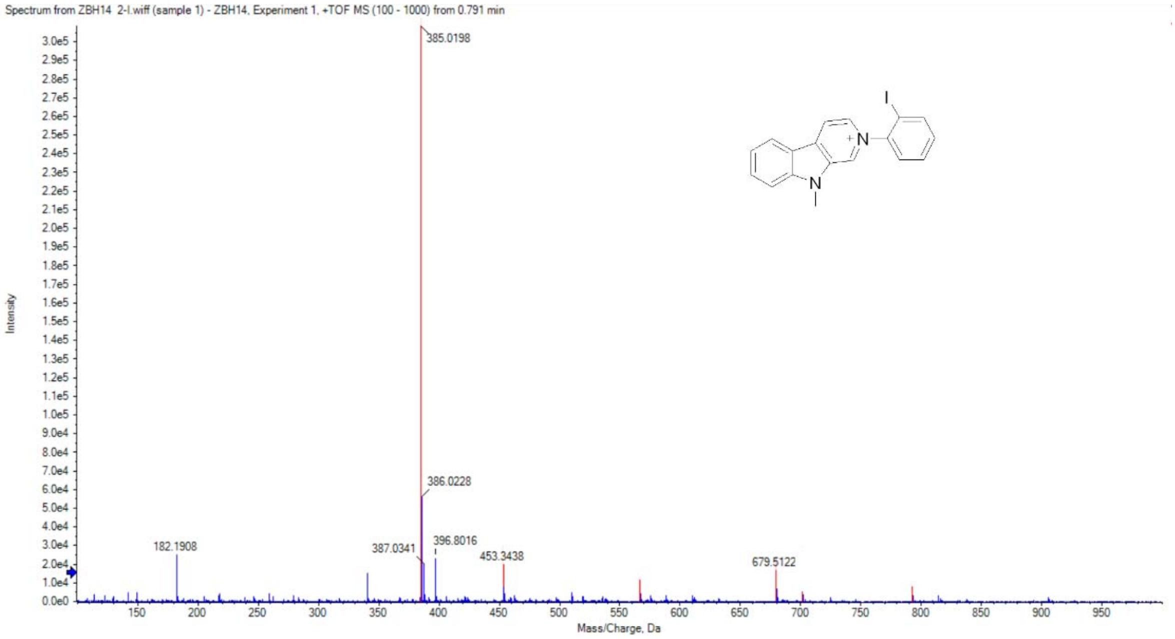

HR-ESI-MS of Compound B**11**

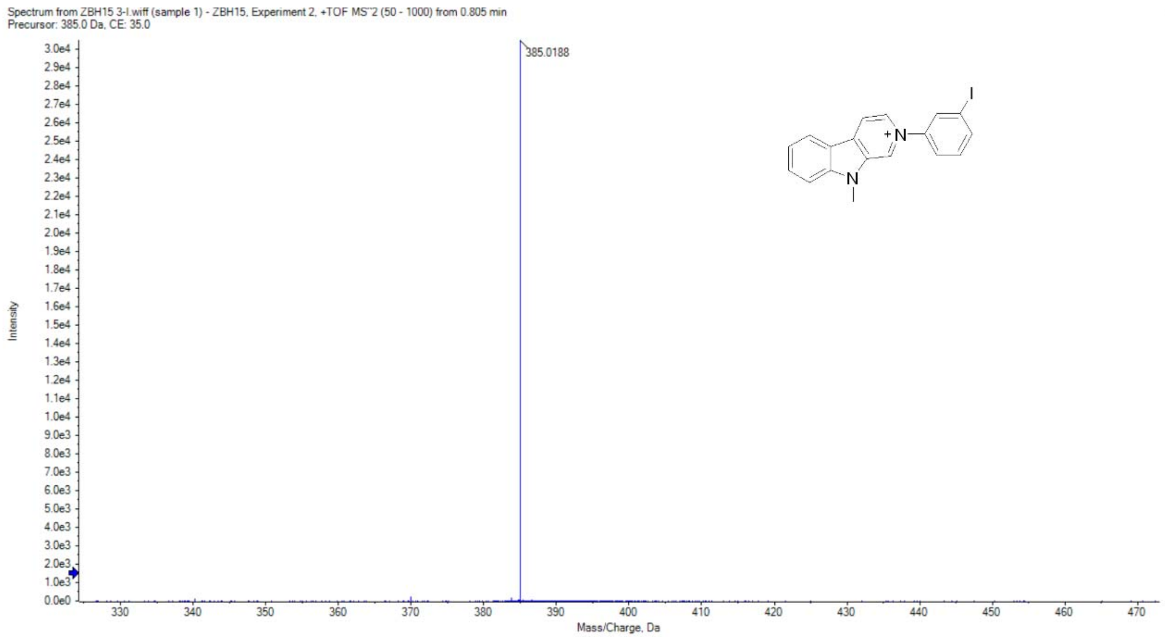

HR-ESI-MS of Compound B**12**

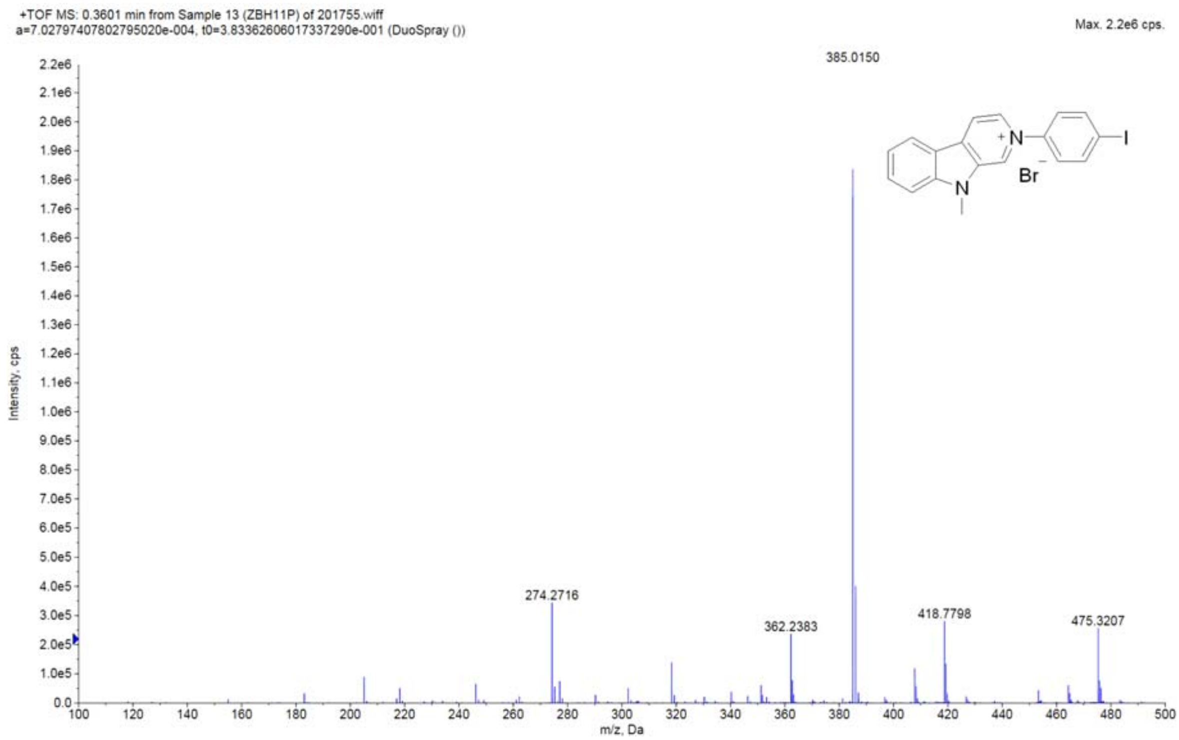

HR-ESI-MS of Compound B**13**

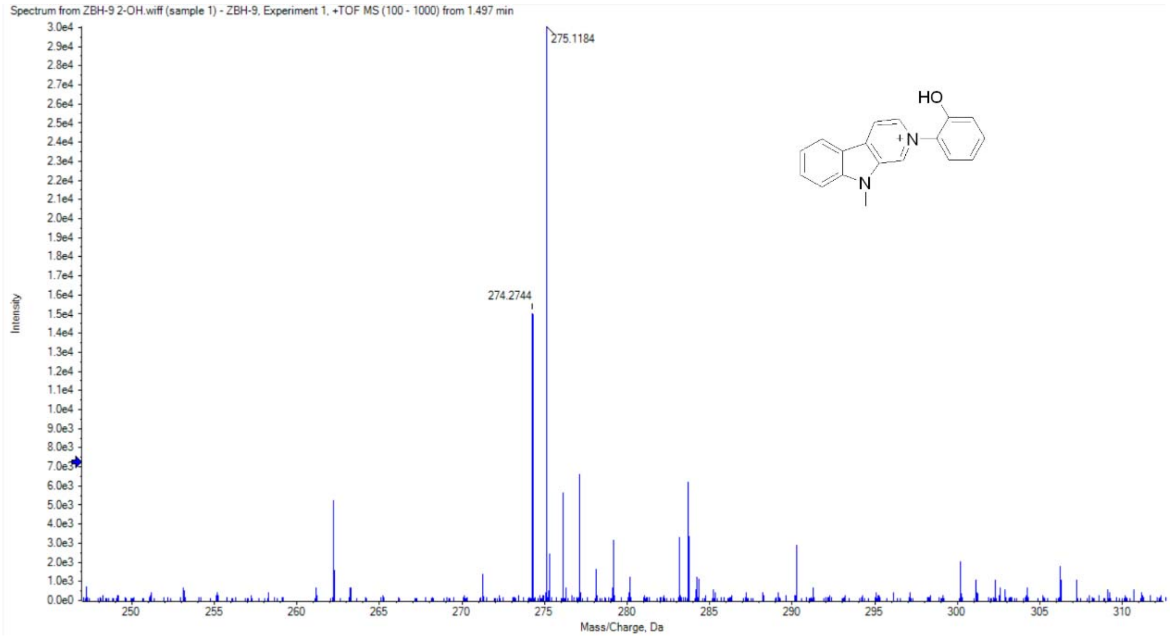

HR-ESI-MS of Compound B**14**

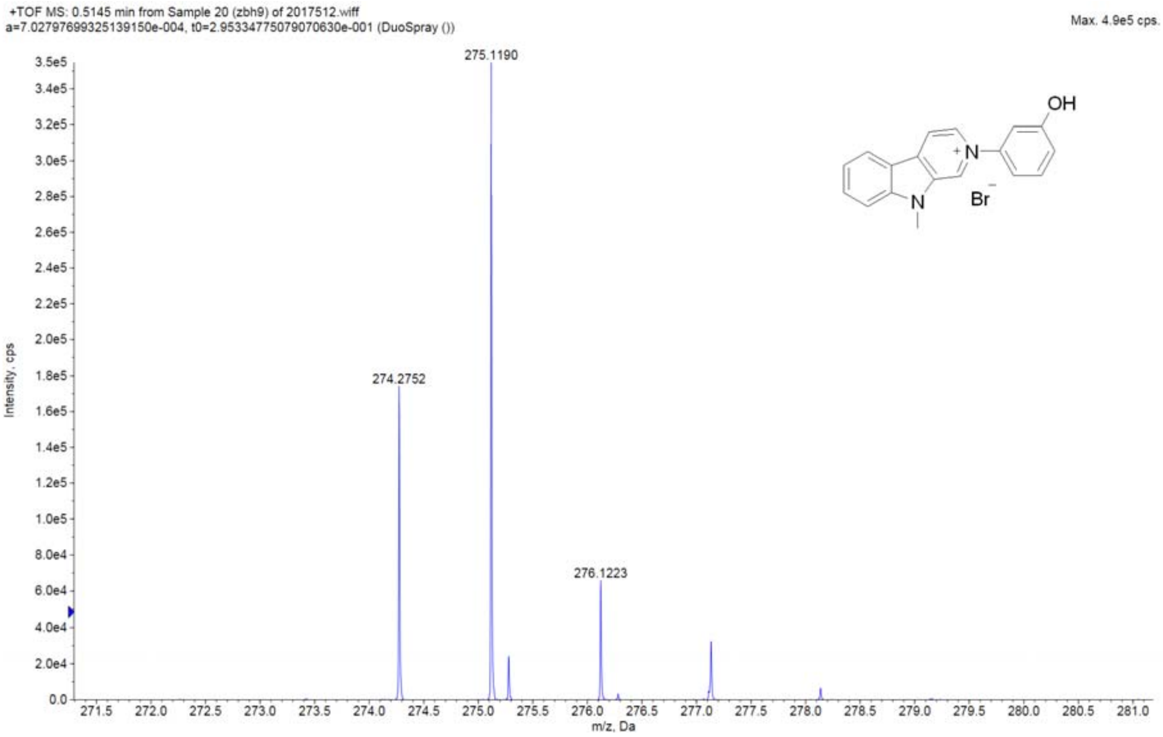

HR-ESI-MS of Compound B**15**

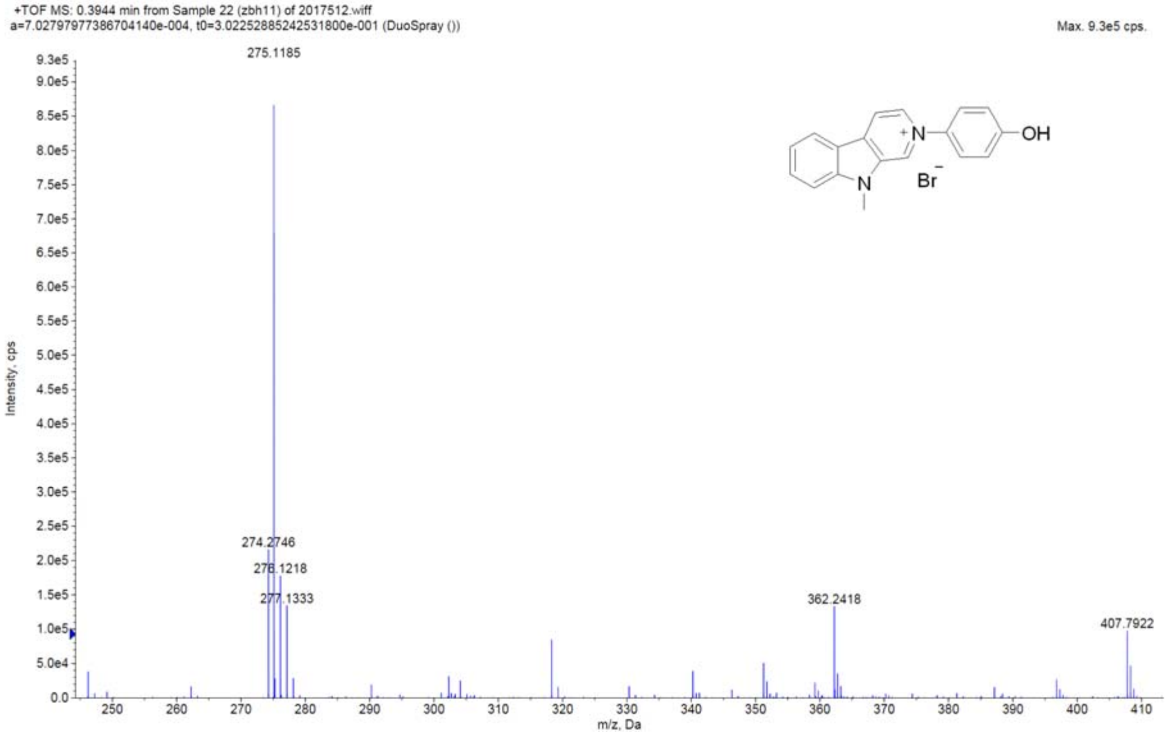

HR-ESI-MS of Compound B**16**

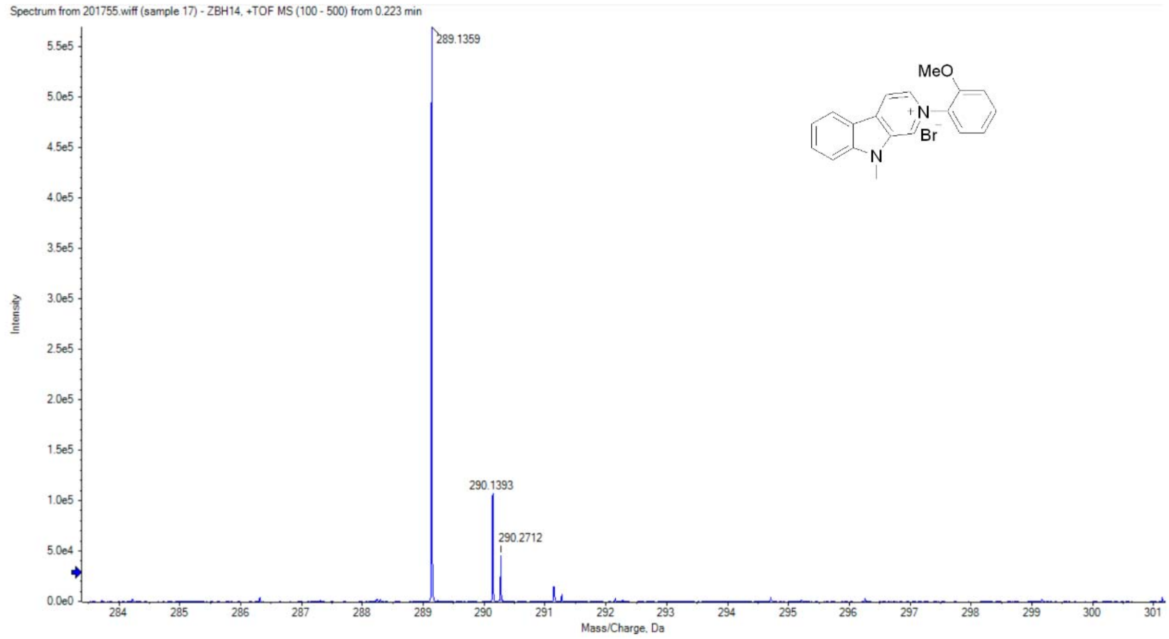

HR-ESI-MS of Compound B**17**

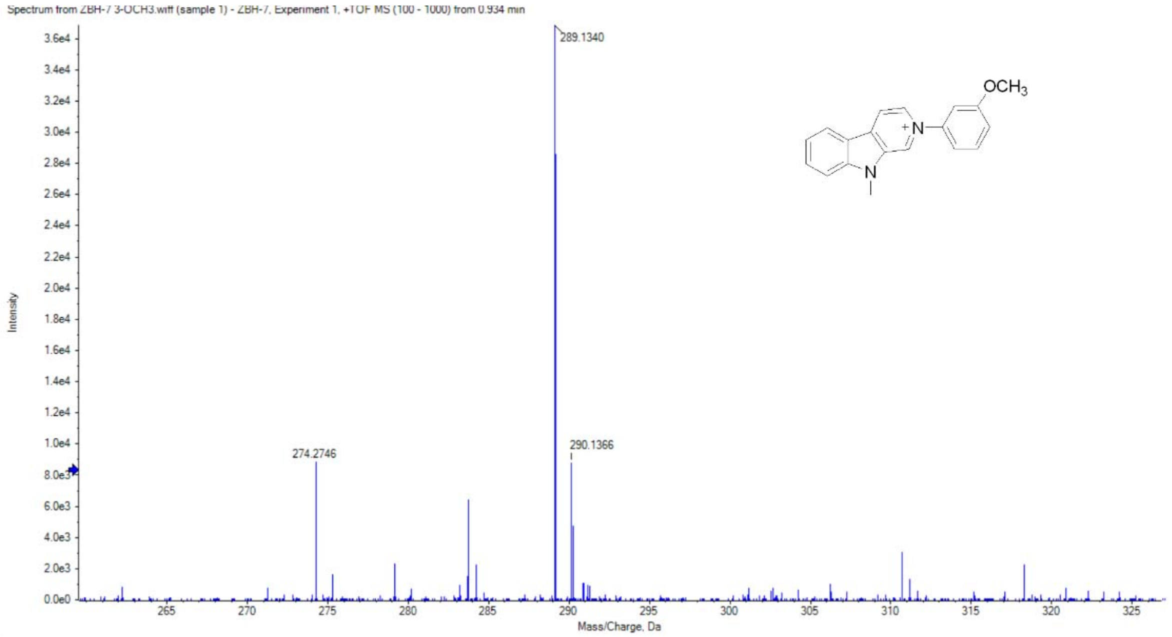

HR-ESI-MS of Compound B**18**

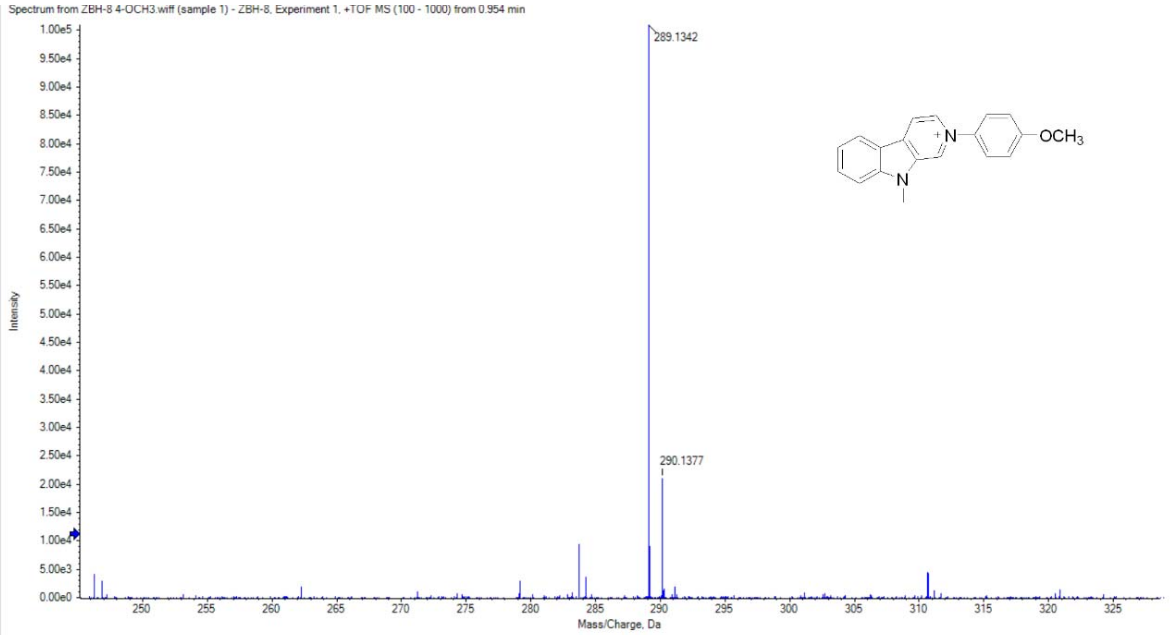

HR-ESI-MS of Compound B**19**

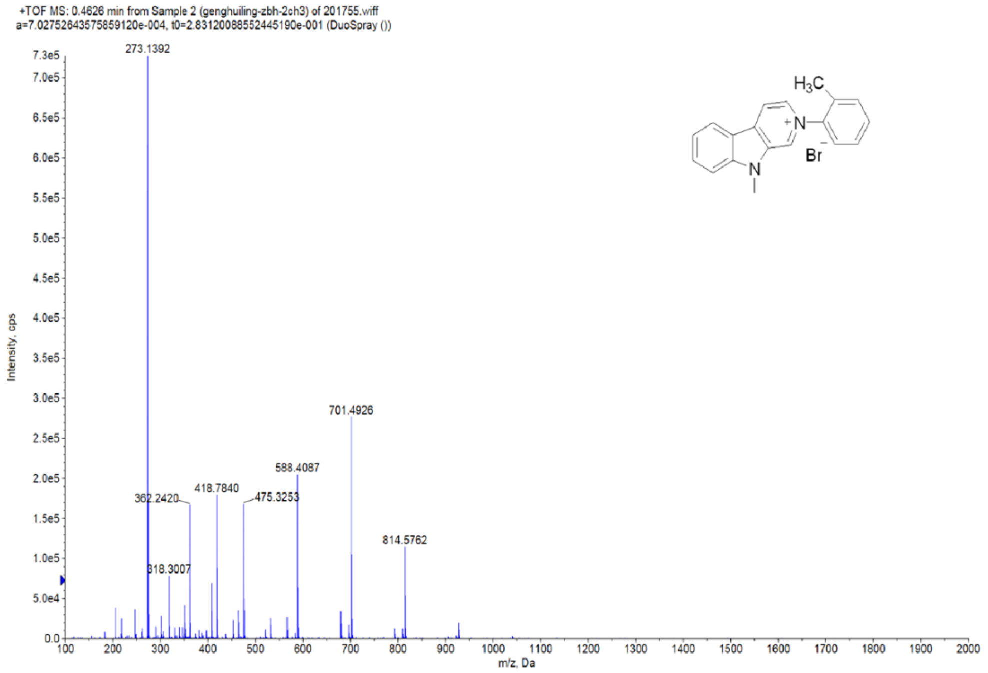

HR-ESI-MS of Compound B**20**

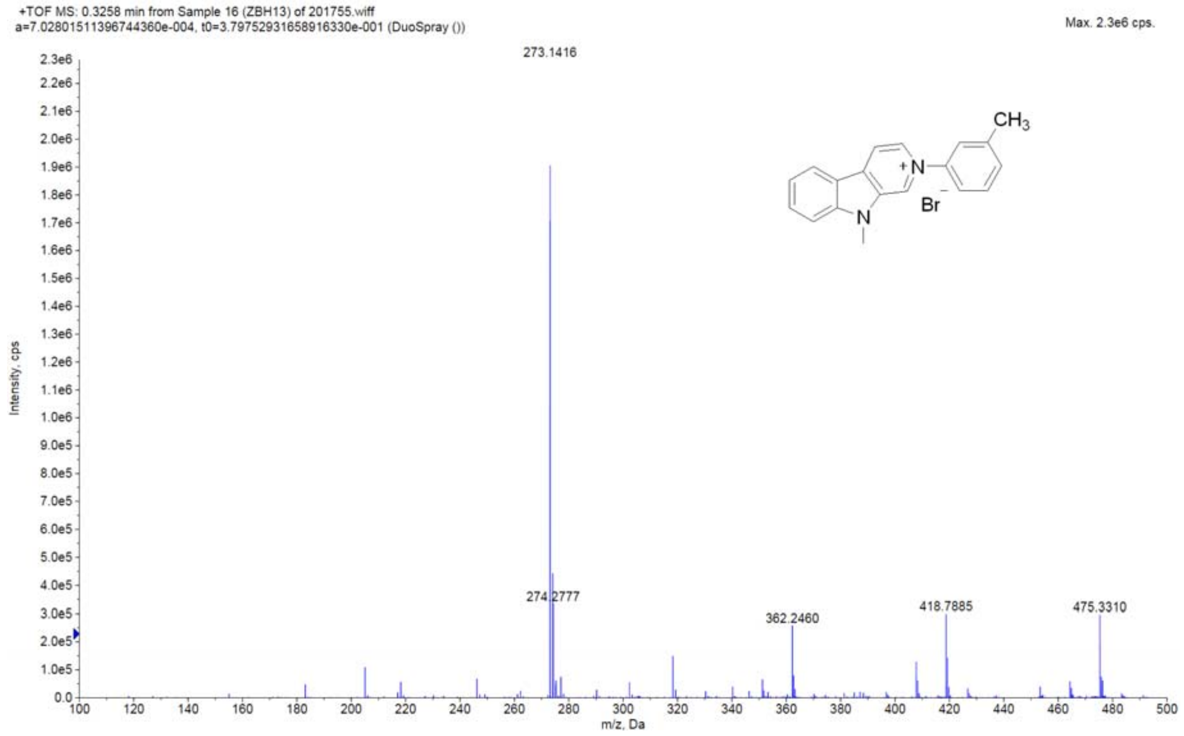

HR-ESI-MS of Compound B**21**

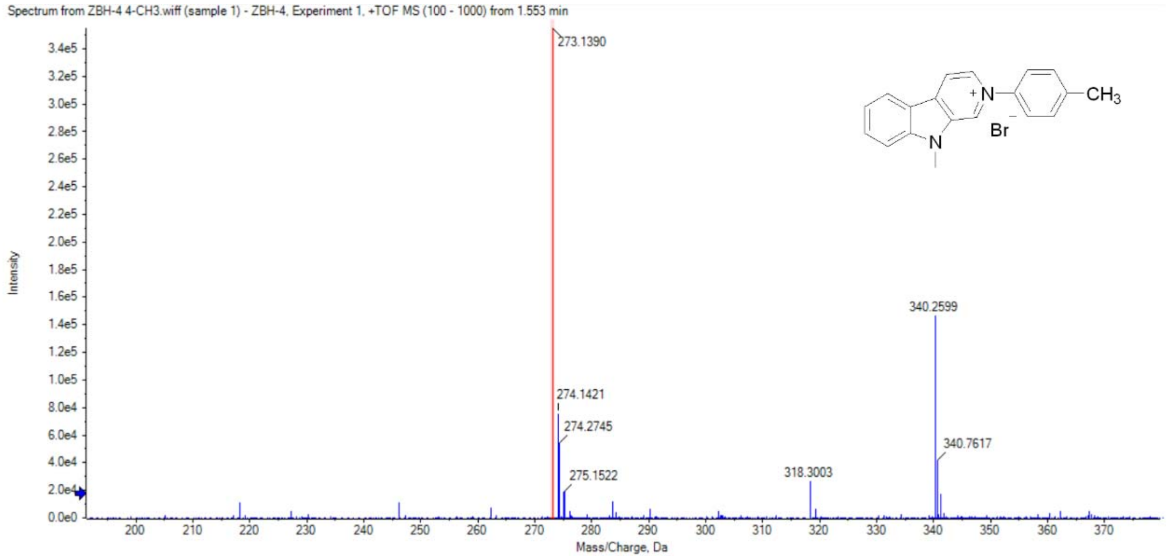

HR-ESI-MS of Compound B**22**

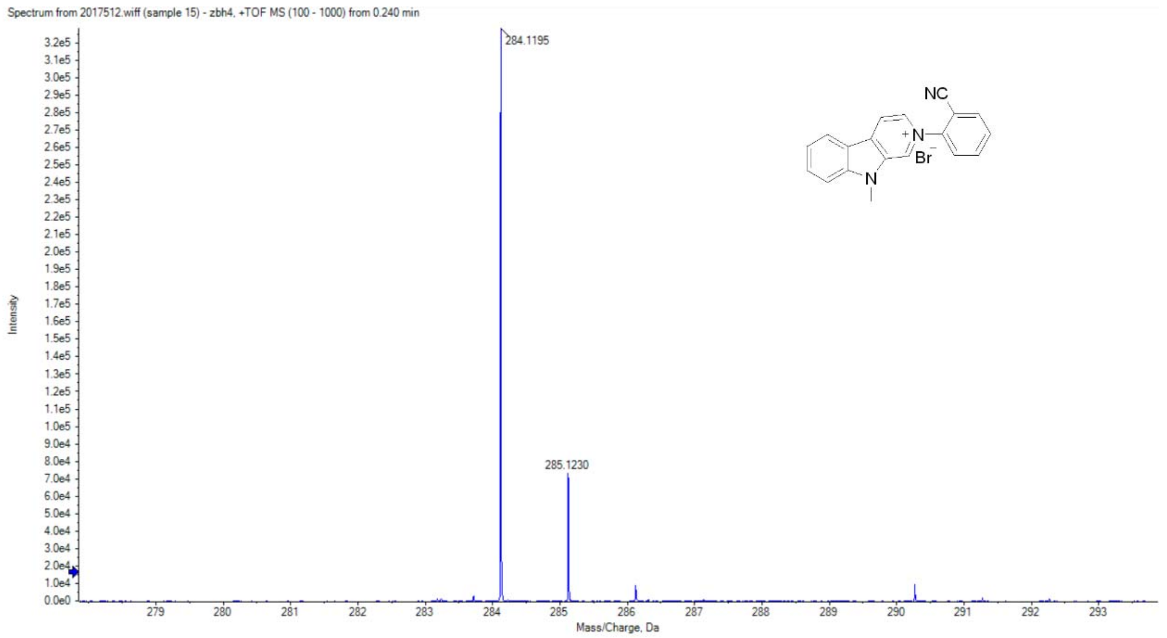

HR-ESI-MS of Compound B**23**

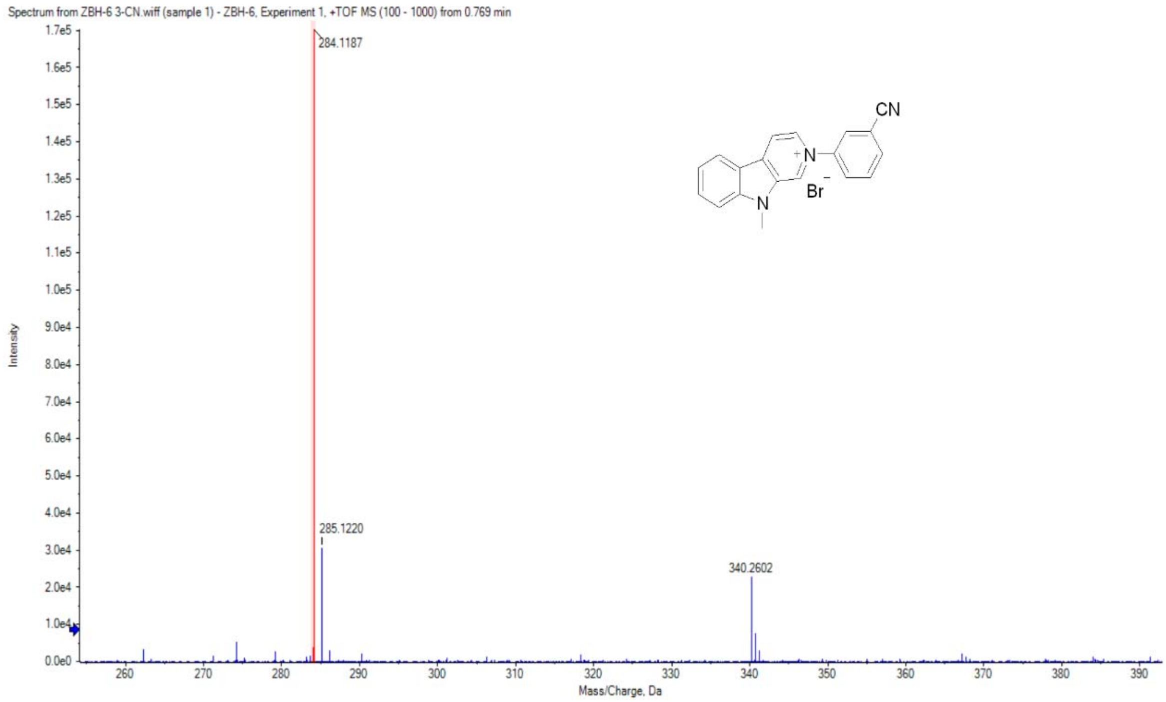

HR-ESI-MS of Compound B**24**

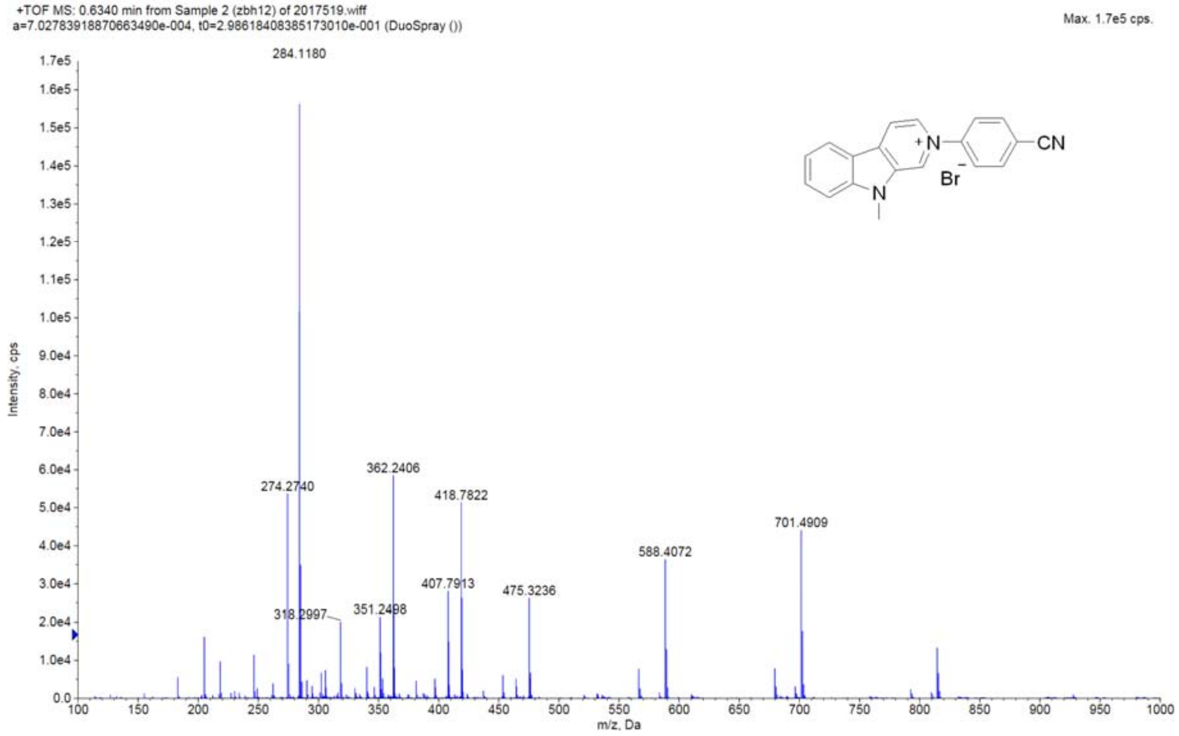

HR-ESI-MS of Compound B**25**

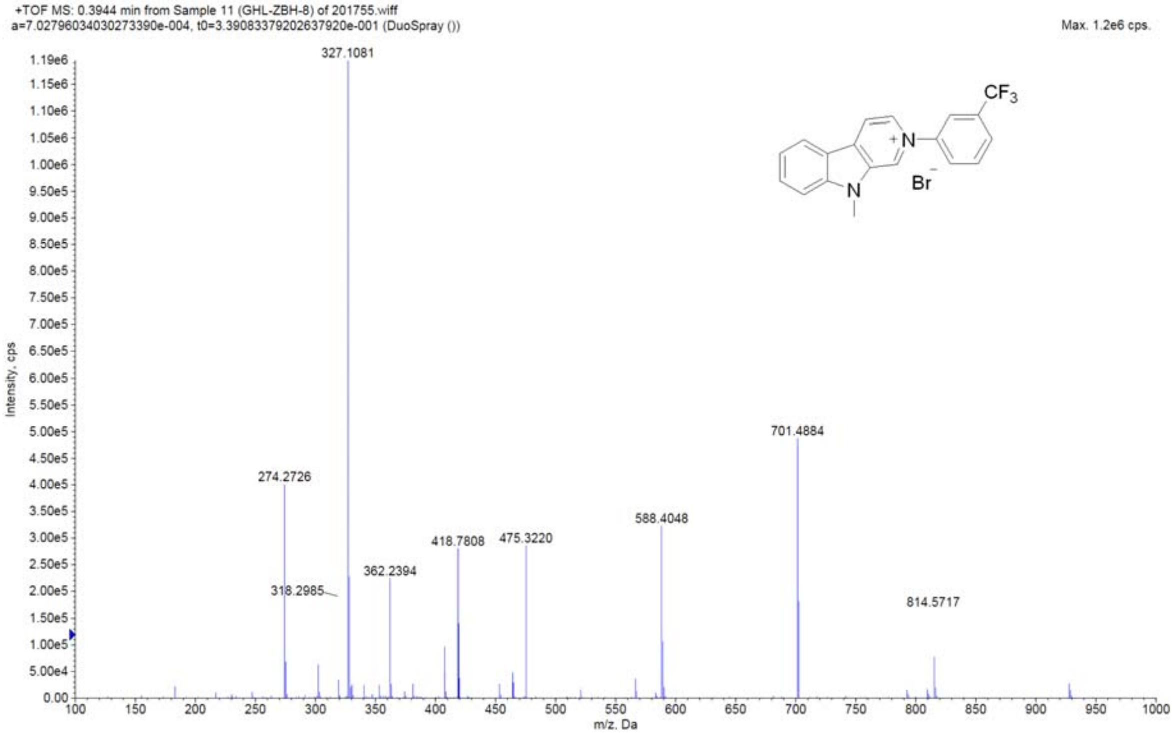

HR-ESI-MS of Compound B**26**

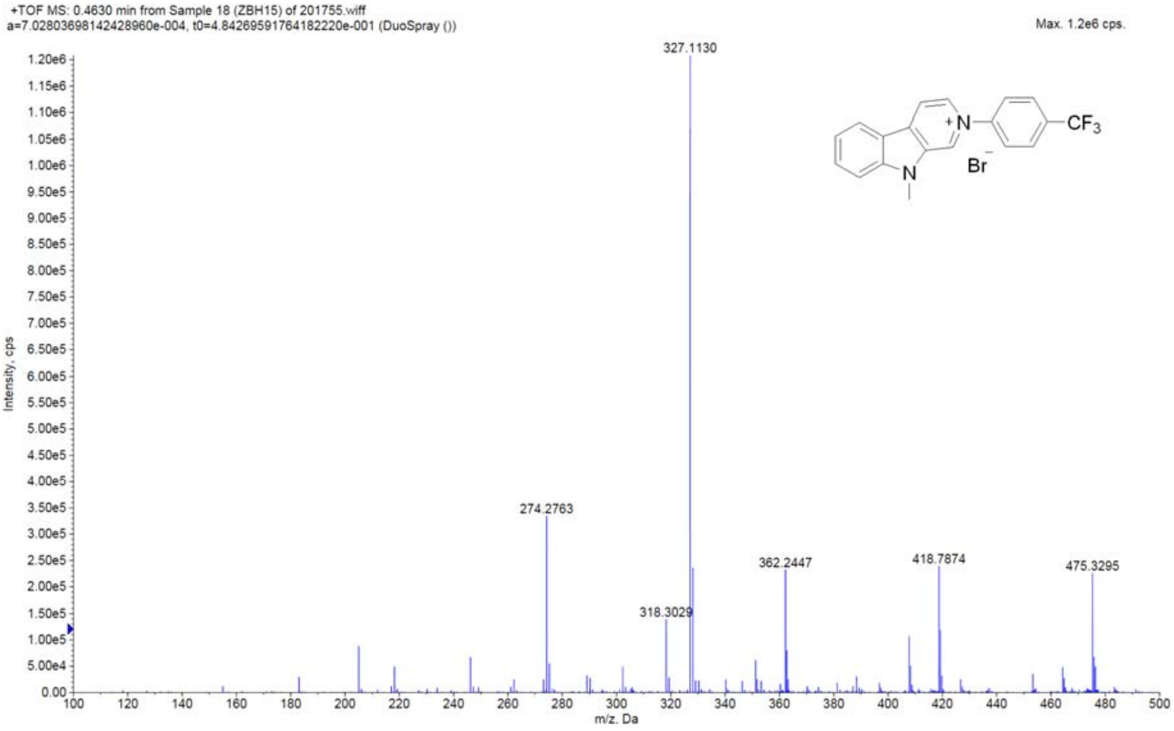

HR-ESI-MS of Compound B**27**

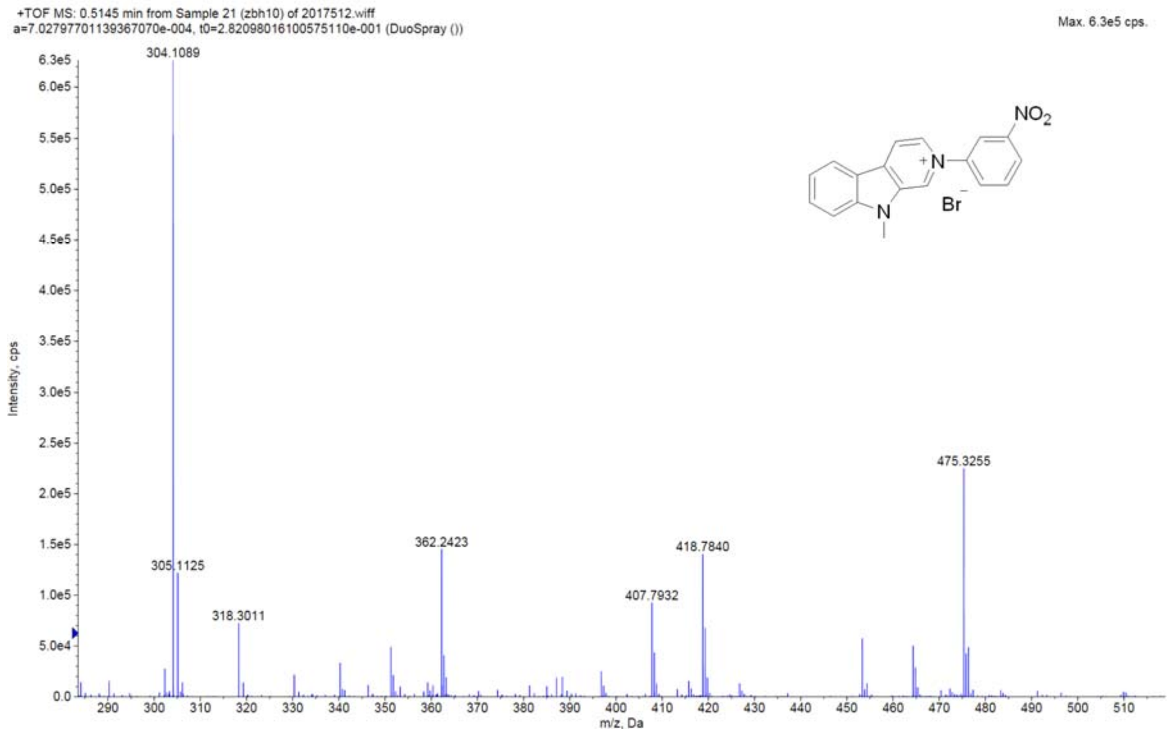

HR-ESI-MS of Compound B**28**

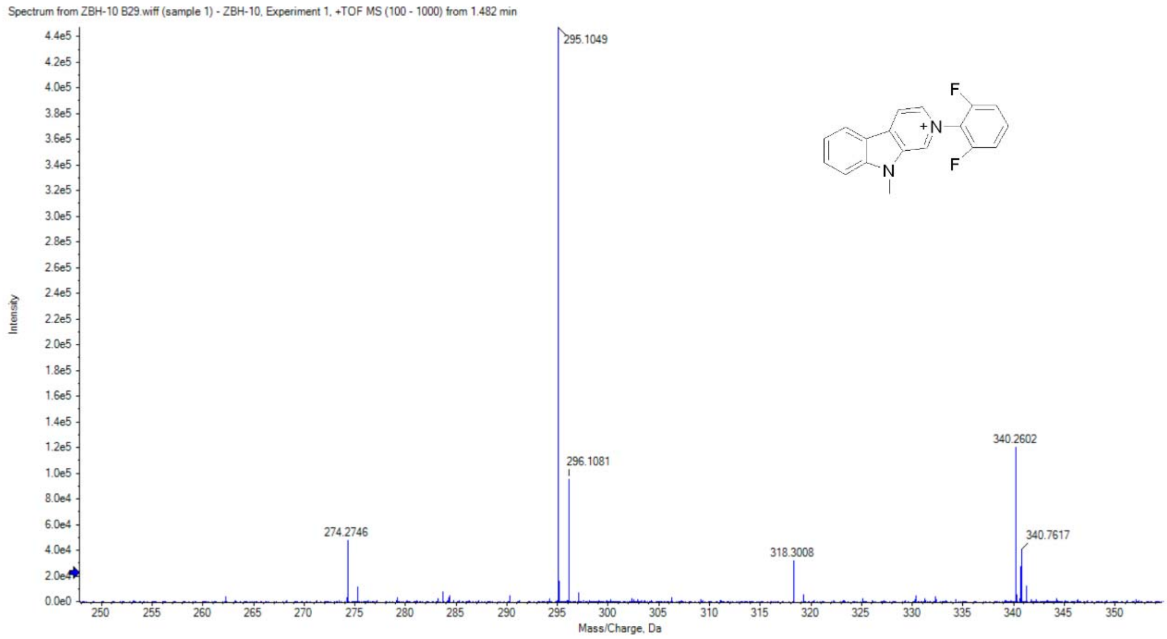

HR-ESI-MS of Compound B**29**

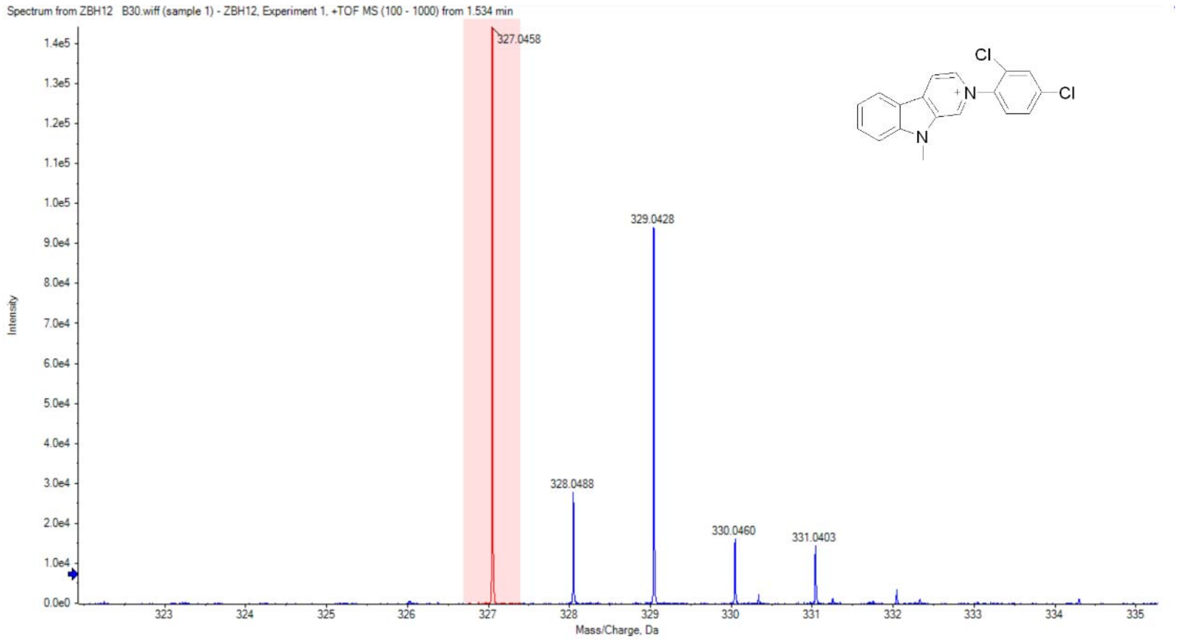

HR-ESI-MS of Compound B**30**

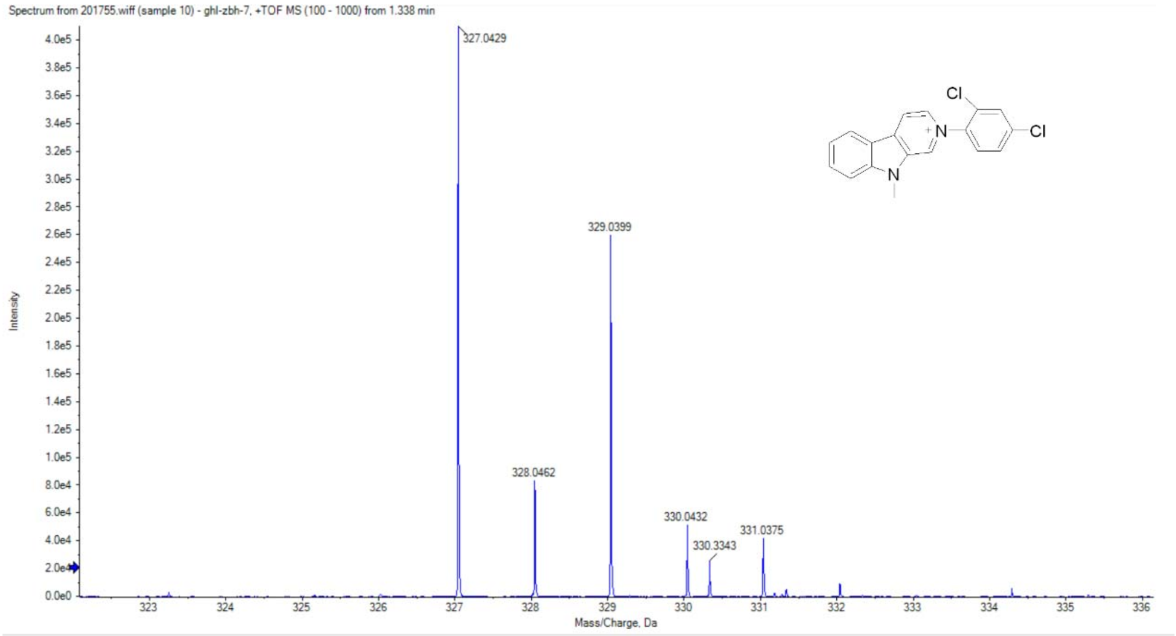

HR-ESI-MS of Compound B**31**

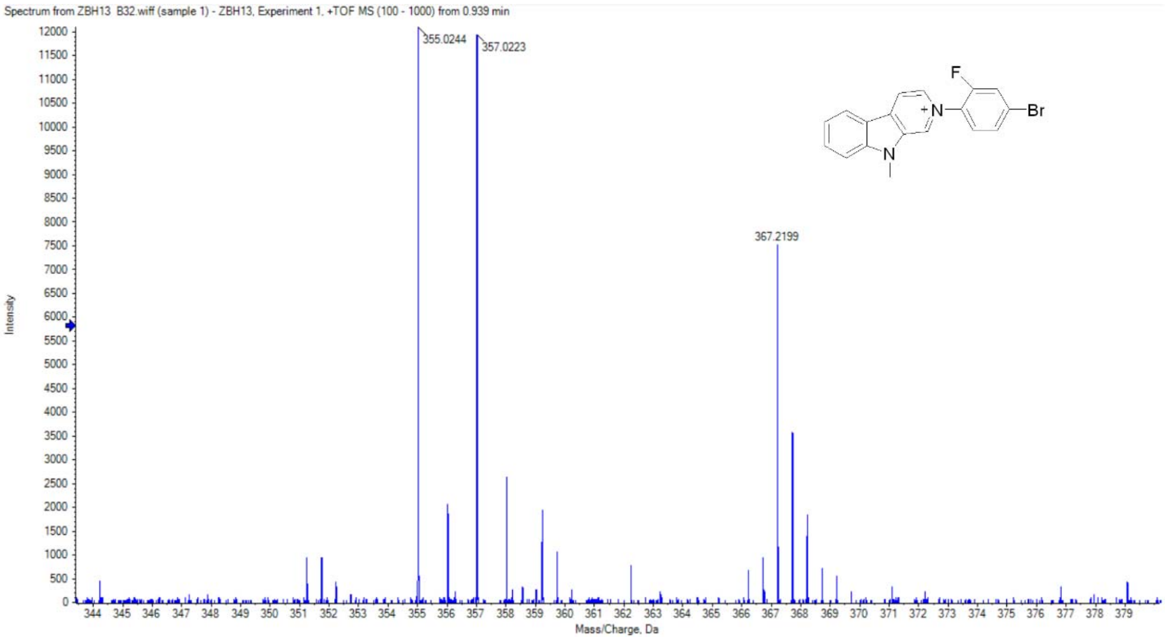

HR-ESI-MS of Compound B**32**

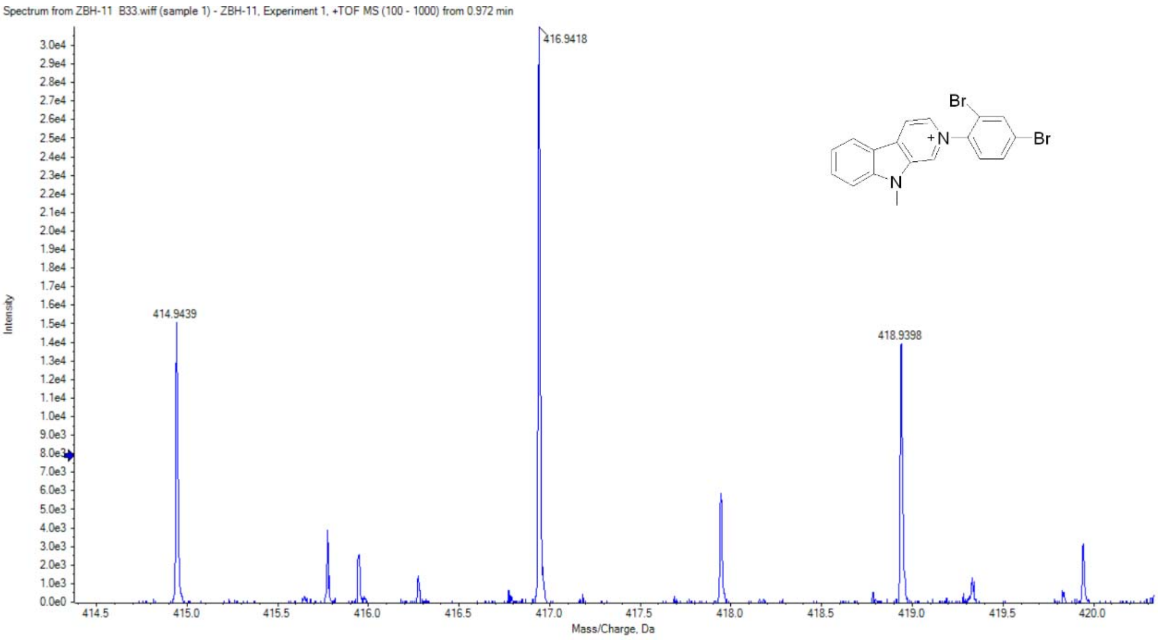

HR-ESI-MS of Compound B**33**

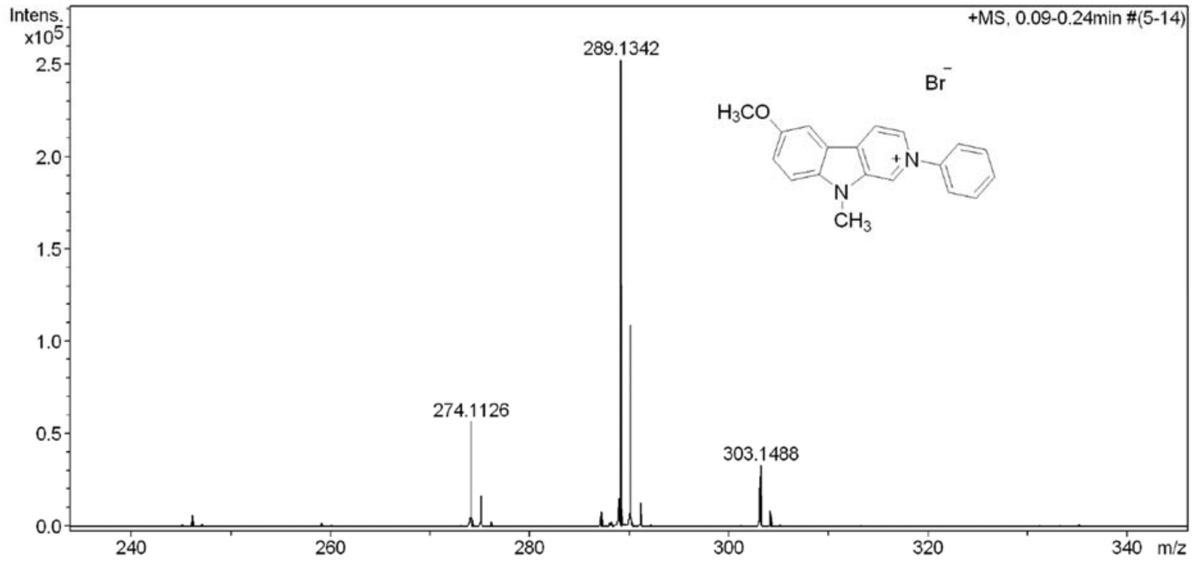

HR-ESI-MS of Compound B**34**

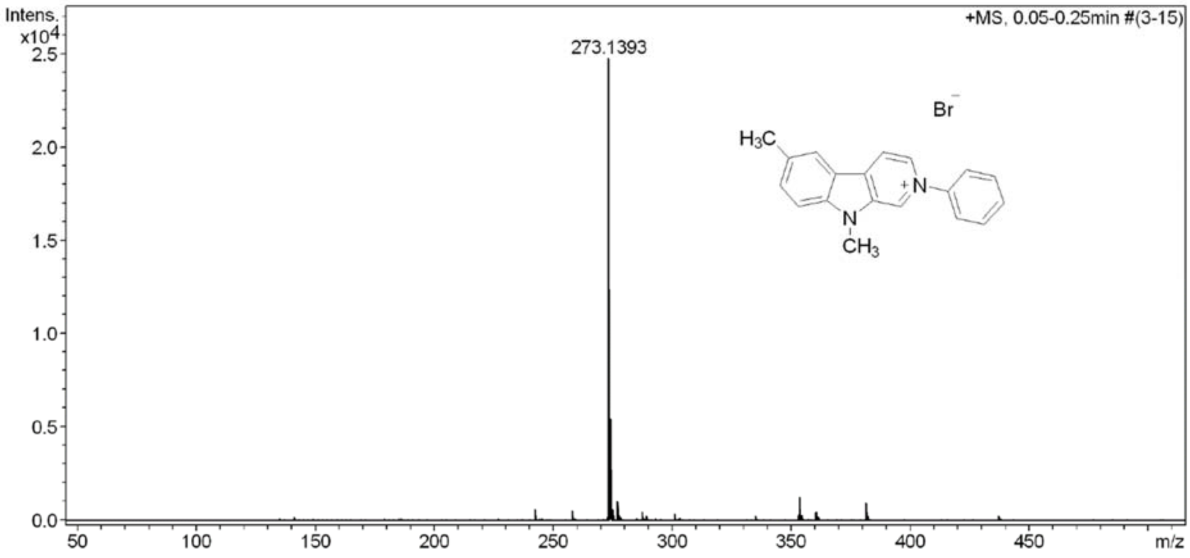

HR-ESI-MS of Compound B**35**

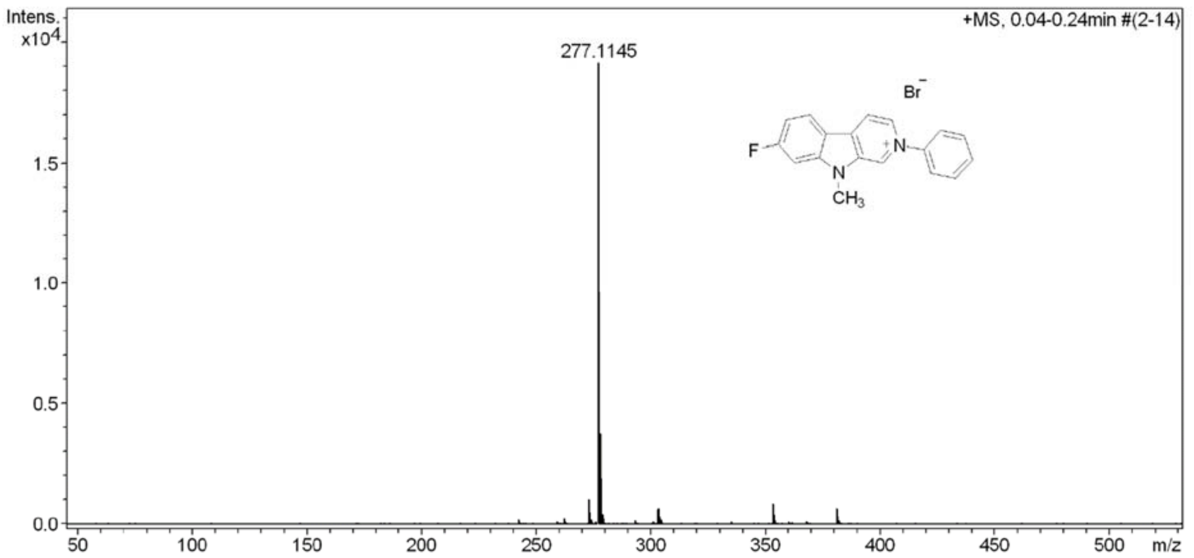

HR-ESI-MS of Compound B**36**

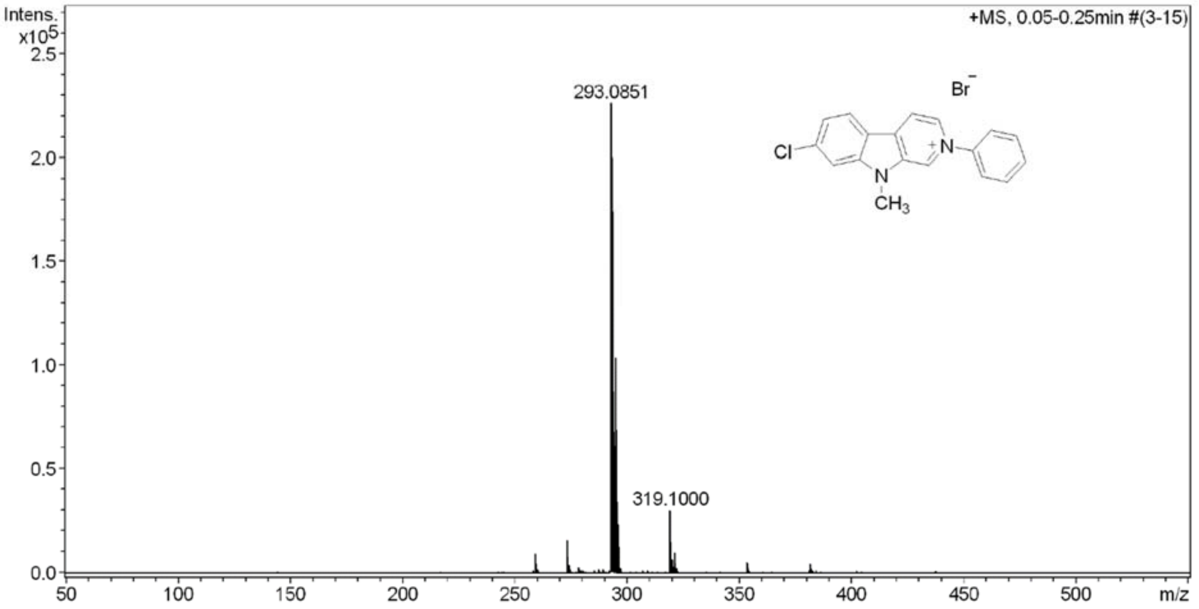

HR-ESI-MS of Compound B**37**

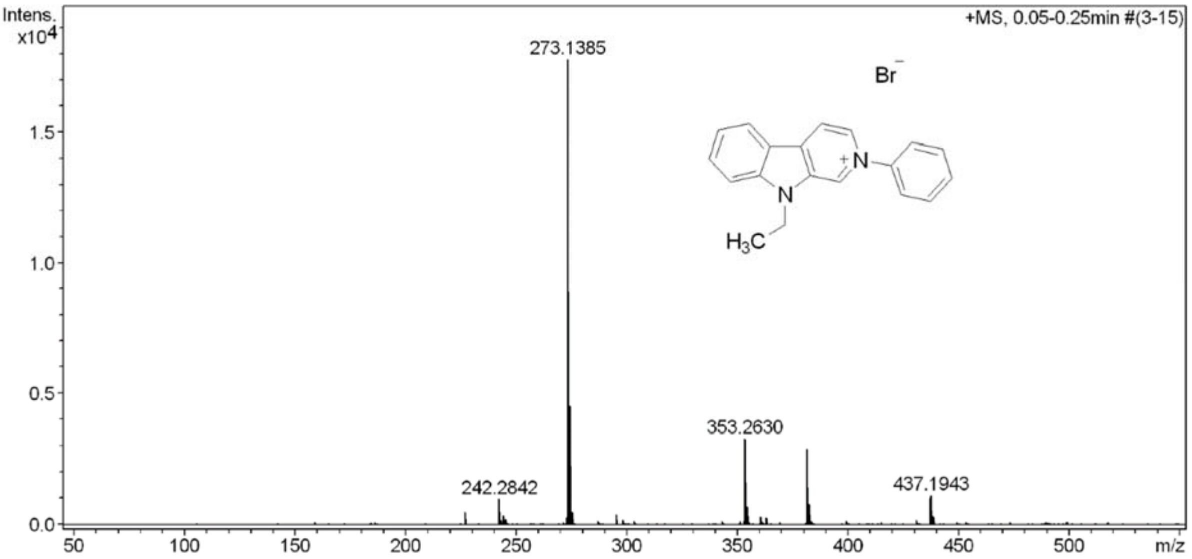

HR-ESI-MS of Compound B**38**

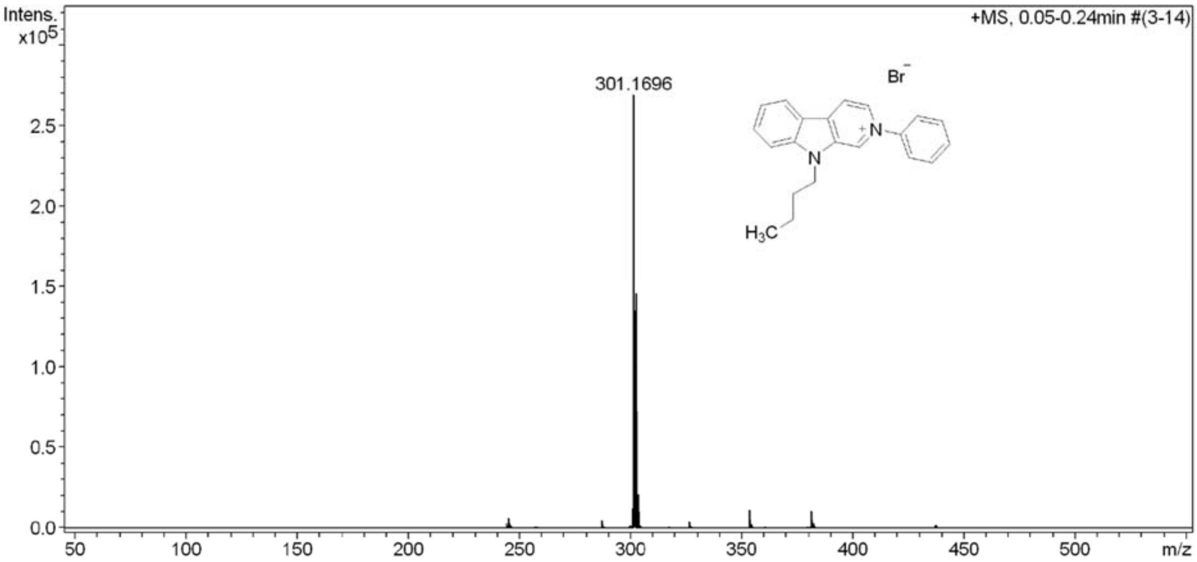

HR-ESI-MS of Compound B**39**

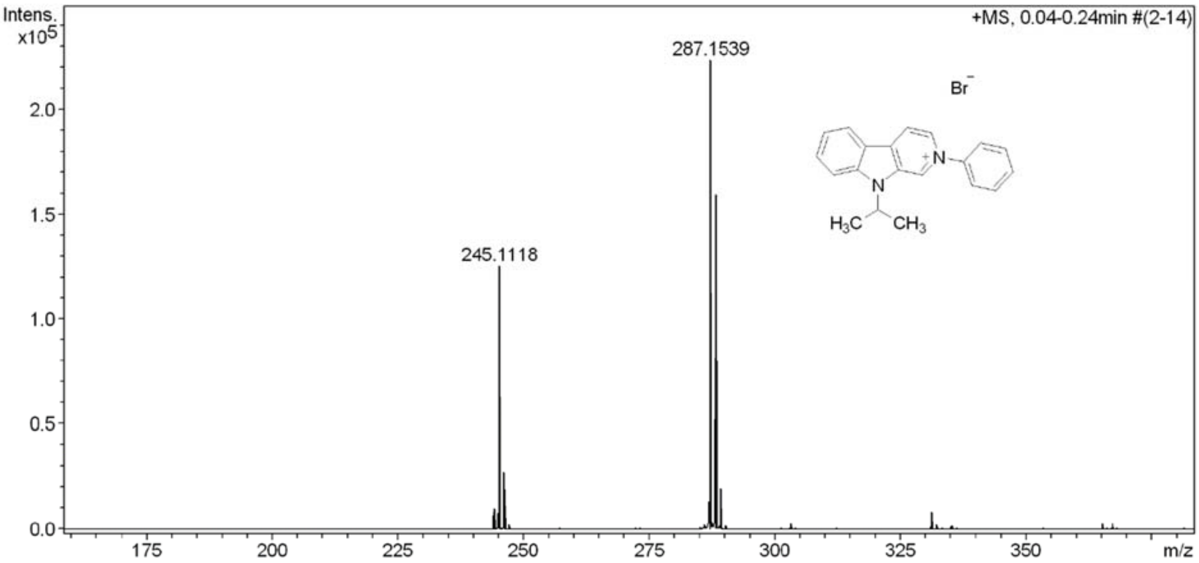

HR-ESI-MS of Compound B**40**

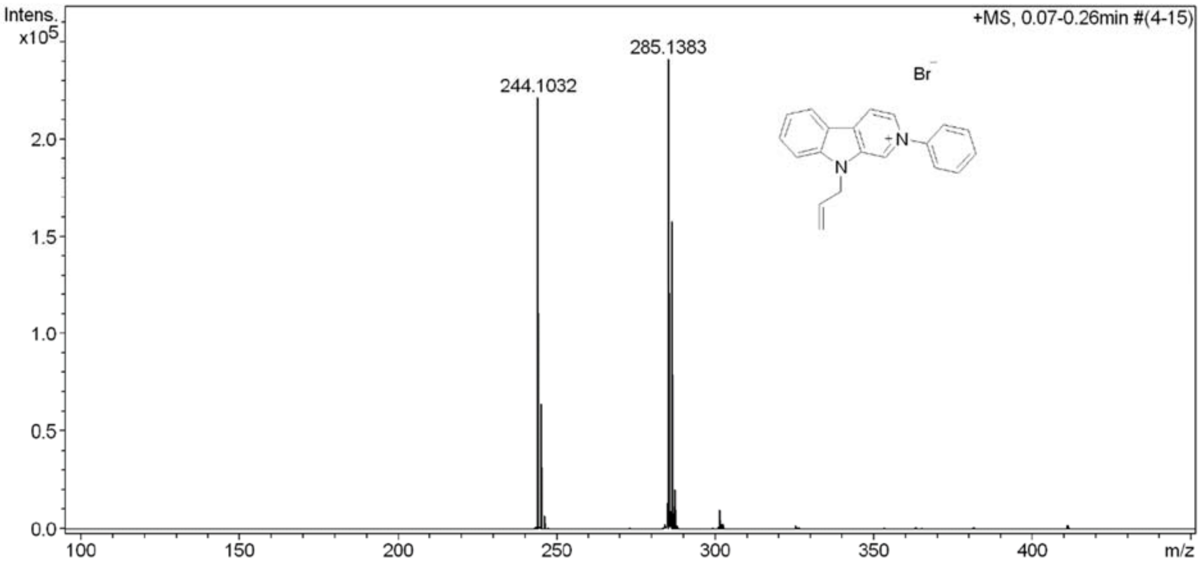

HR-ESI-MS of Compound B**41**

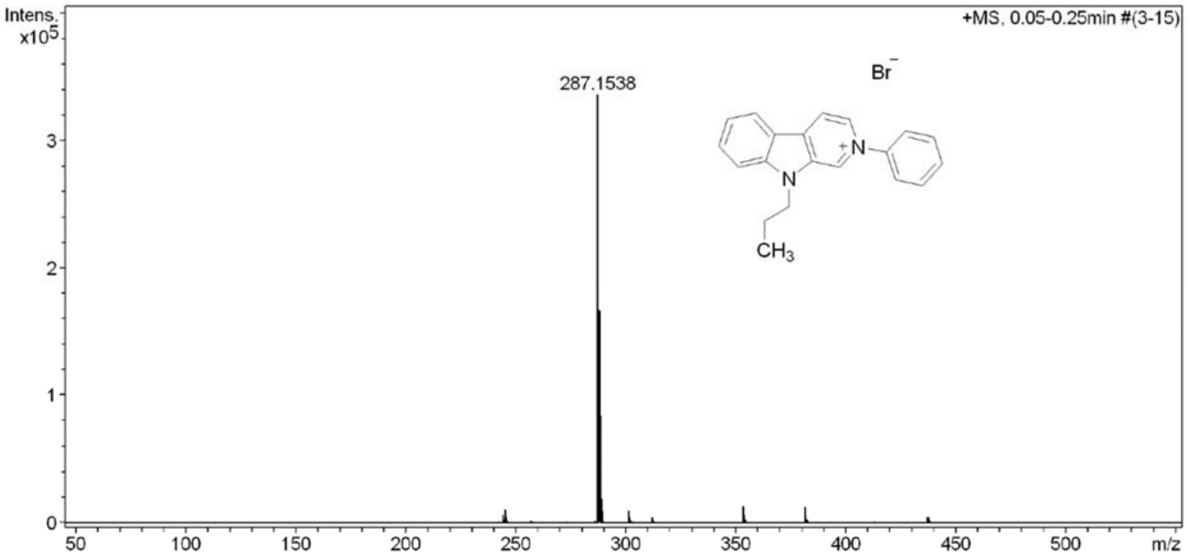

HR-ESI-MS of Compound B**42**

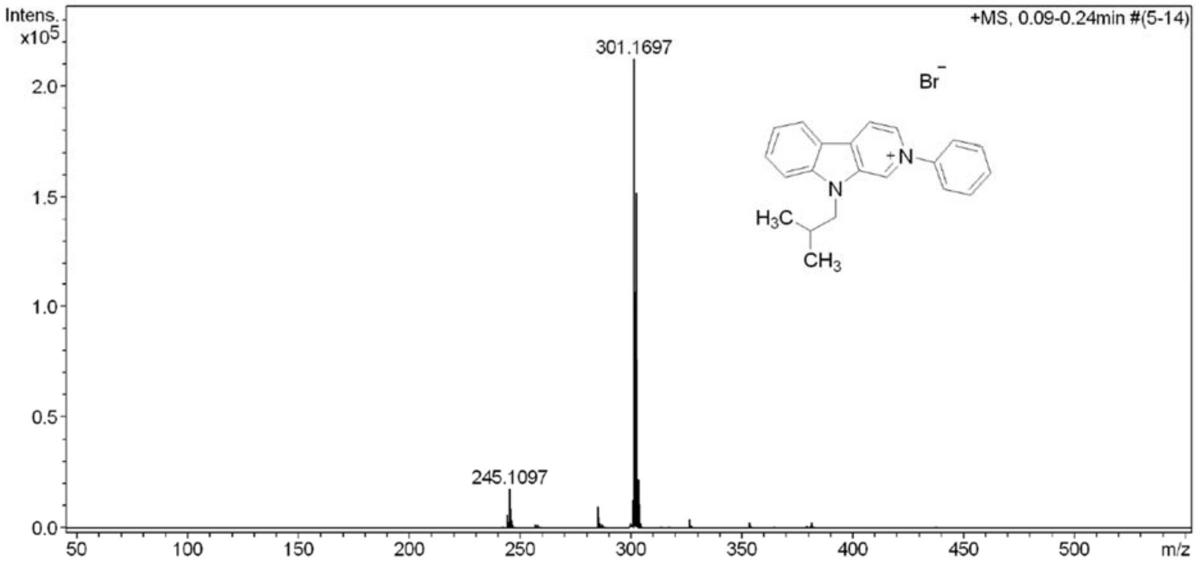

HR-ESI-MS of Compound B**43**

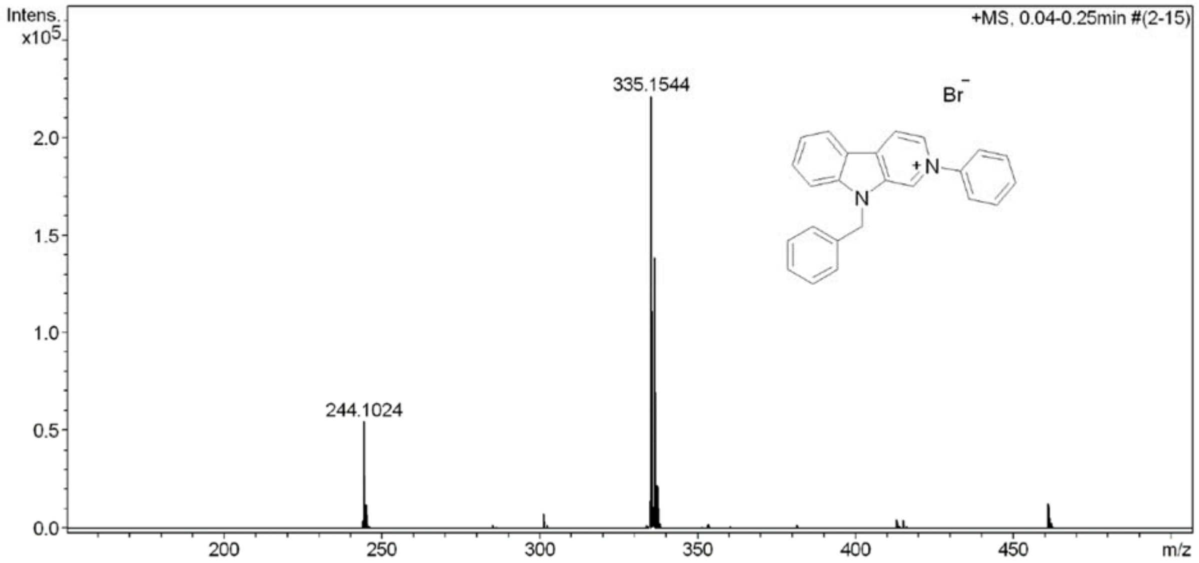

HR-ESI-MS of Compound B**44**

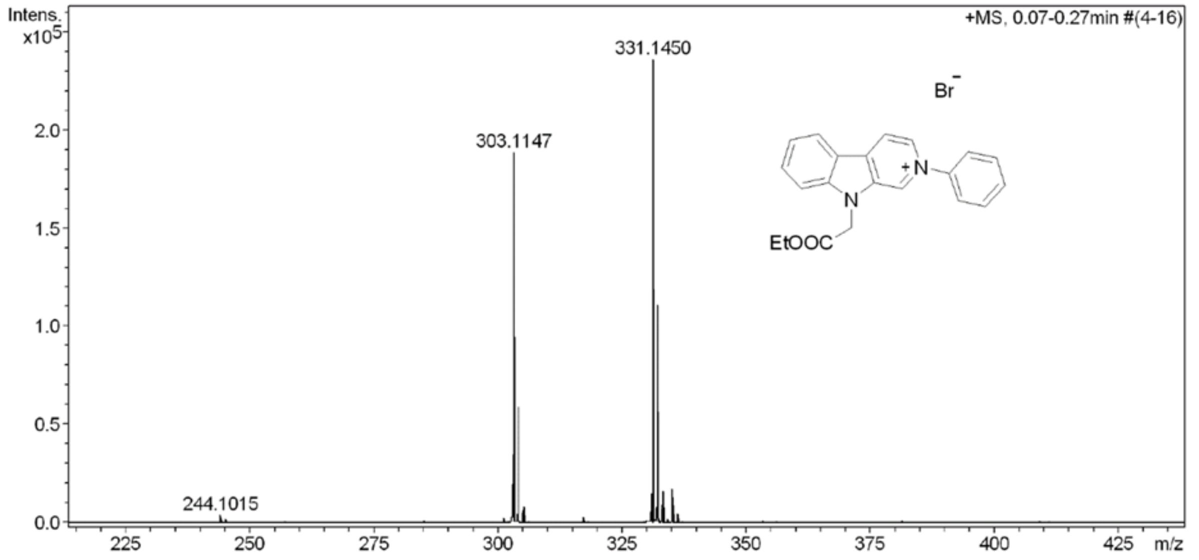

HR-ESI-MS of Compound B**45**

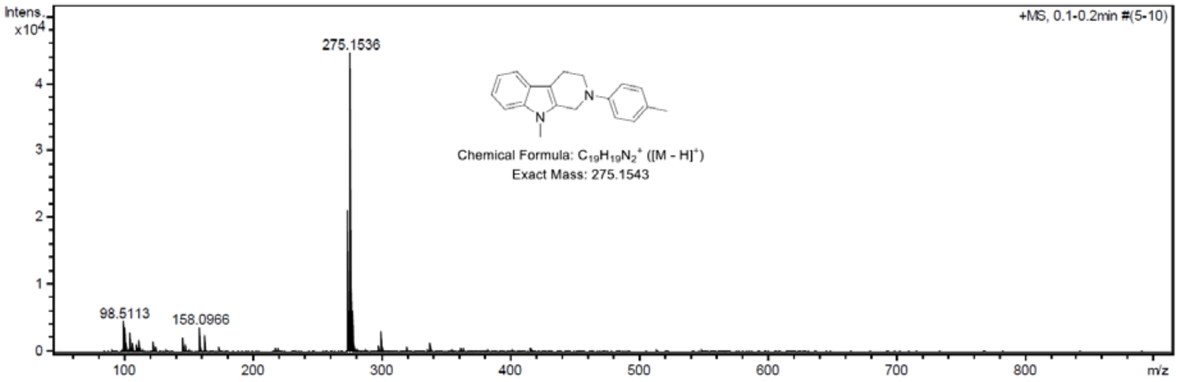

HR-ESI-MS of Compound **C1**

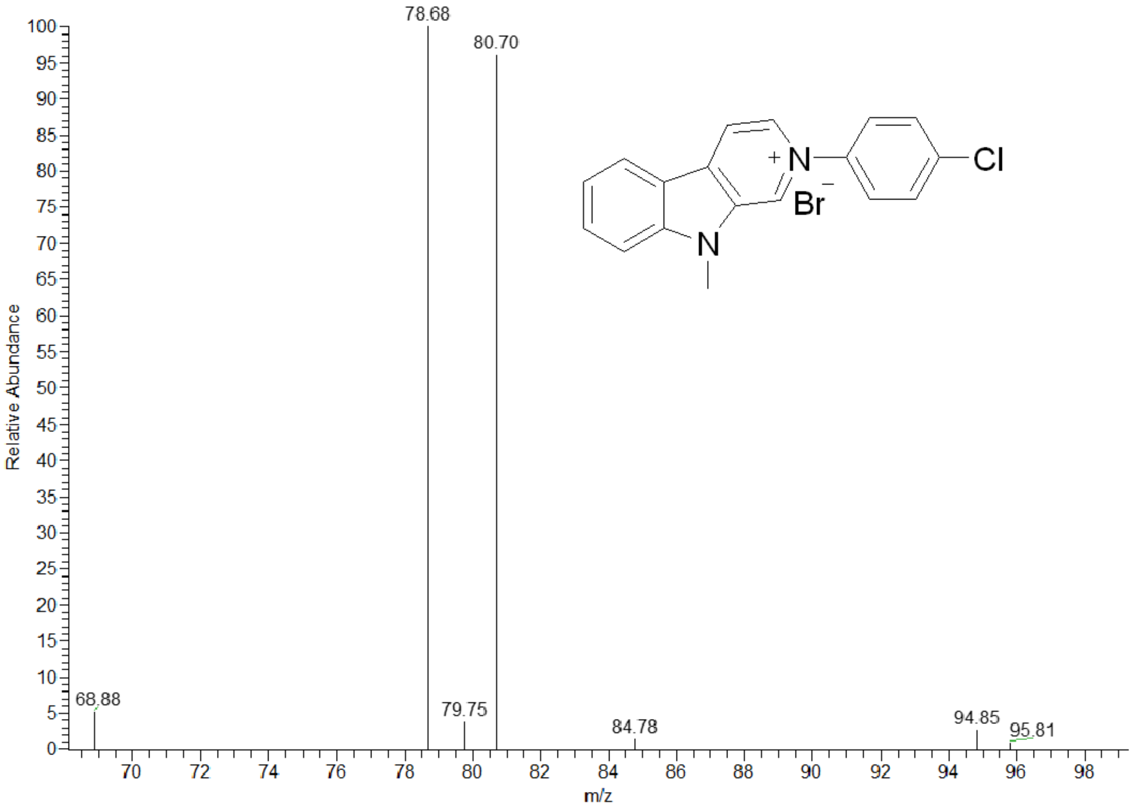

Negative MS of **B7**

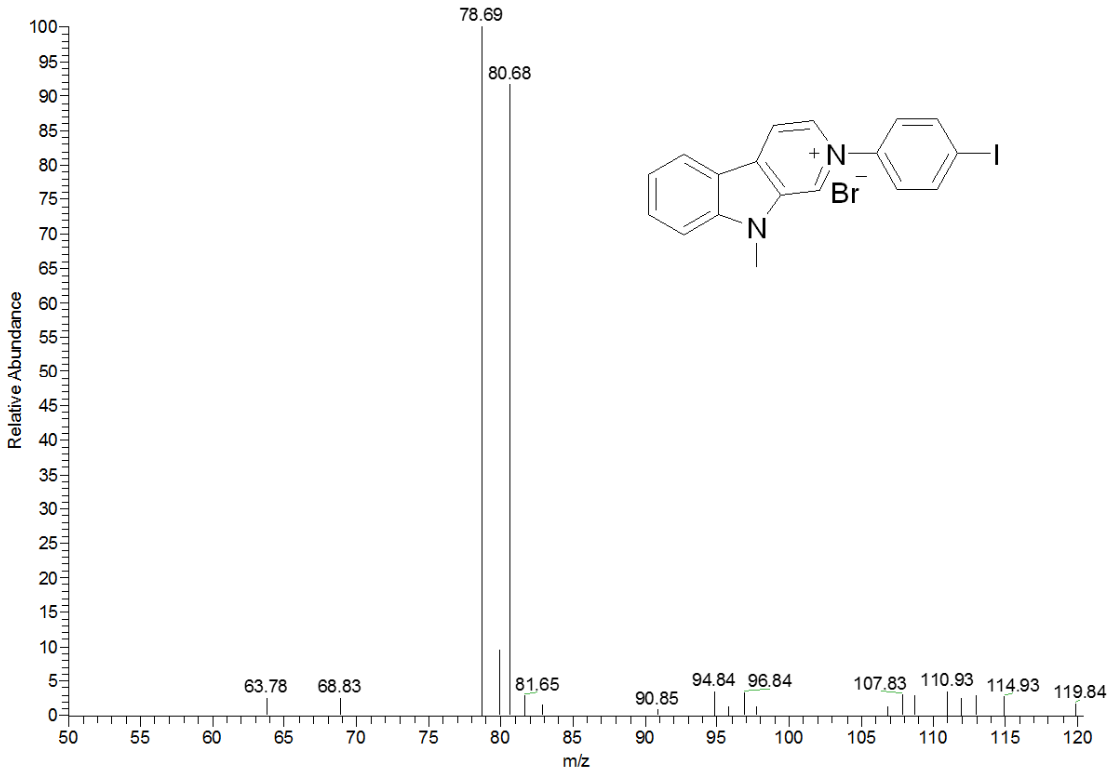

Negative MS of **B13**

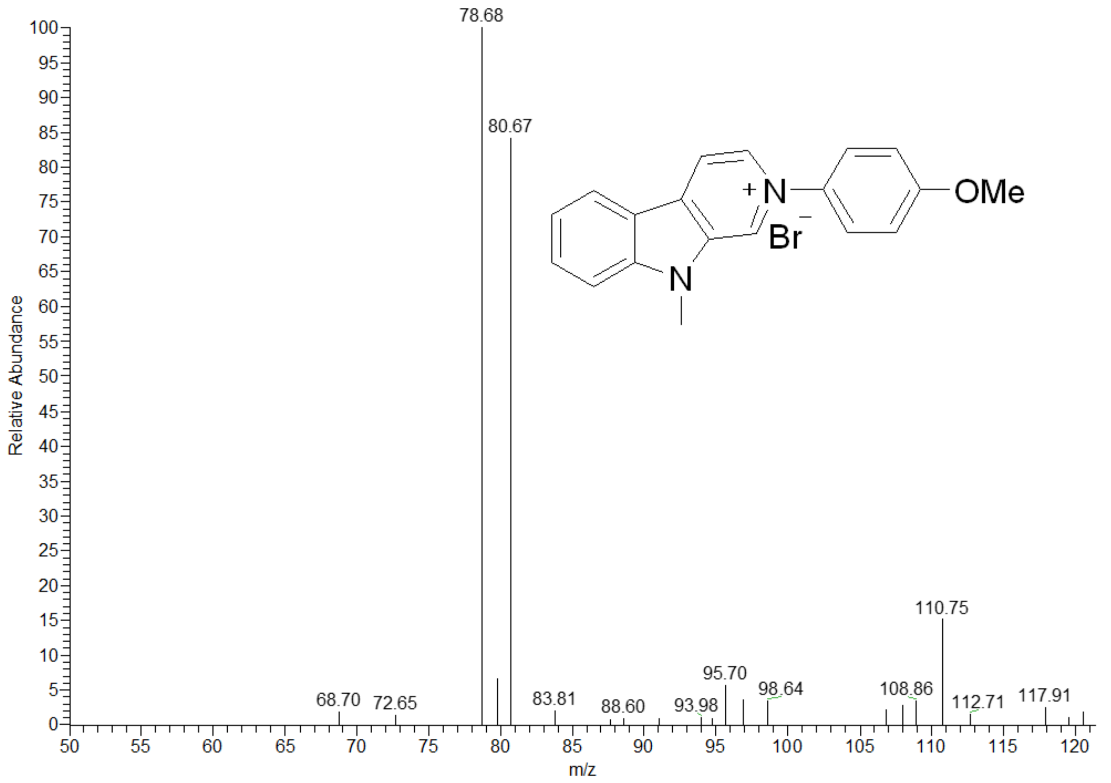

Negative MS of **B19**

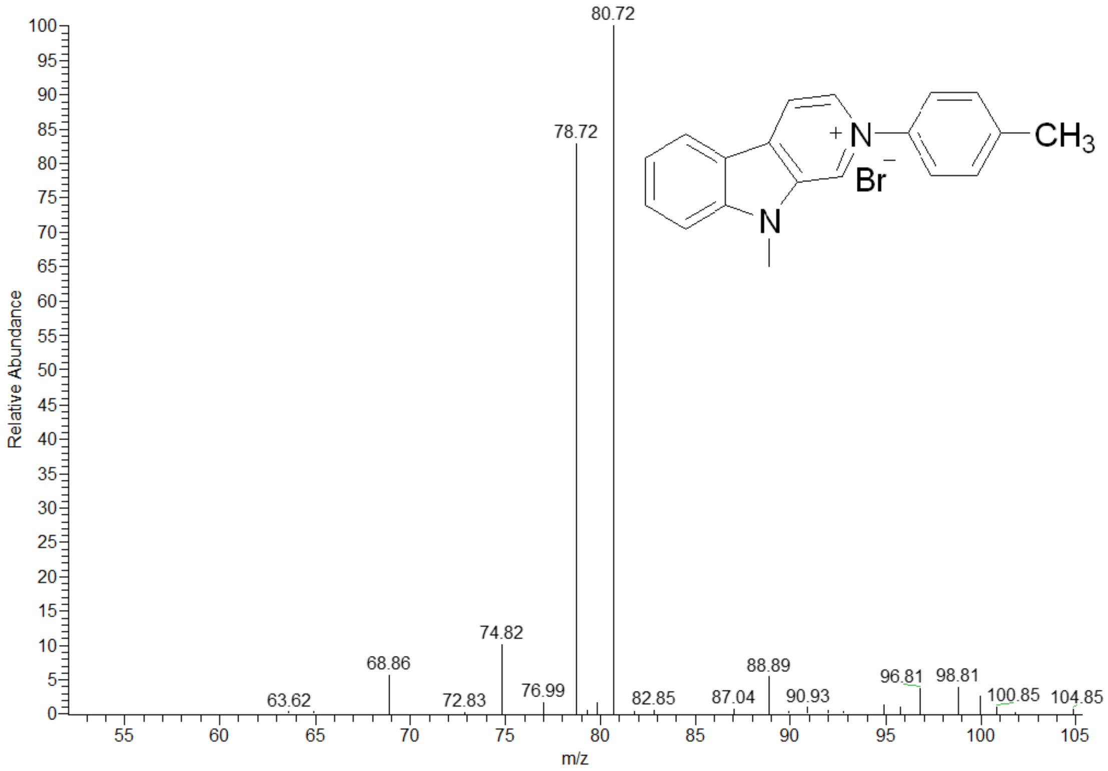

Negative MS of **B22**

Molecular docking studies.

Molecular docking simulations were performed using the software Autodock 4.2 along with AutoDock Tools (ADT 1.5.6) using the hybrid Lamarckian Genetic Algorithm (LGA) as our previous studies[1]. The three dimensional (3D) crystal structures of AChE (PDB code: 4BDT)[2] and BuChE (PDB code: 5K5E)[3] were obtained from the RCSB Protein Data Bank. The standard 3D structure (mol2 format) of all compounds were constructed by using the "SKETCH" option function in SYBYL-X, whose configurations were fully geometry-optimized using a conjugate gradient procedure based on the TRIPOS force field with the Powell conjugate gradient minimization algorithm and a convergence criterion of 0.05 kcal/mol Å. Gasteiger-Hückel charge was used to calculate the partial atomic charges of each structure. The crystal ligand (inhibitor in protein crystal PDB structure) centered maps was defined by the program AutoGrid and the cubic grid box of 40×40×40 Å size (x, y, z) with a spacing of 0.375 Å was built. The docking parameters consisted of setting the population size to 150, the number of evaluations to 2,500,000, the number of generations to 270,000, and the number of top individuals that automatically survive to 20, while the number of docking run was set to 40 with other default values during each docking run. The results of the most favorable free energy of binding were chosen as the resultant complex structures. To test the docking protocol and validate the procedure, we also carried out docking experiments for the crystal ligand (inhibitor, **huprine W**/**6QS**).

References

1. Li, D., Chi, B., Wang, W.-W., Gao, J.-M., Wan, J. Exploring the possible binding mode of trisubstituted benzimidazoles analogues in silico for novel drug design targeting Mtb FtsZ. *Med. Chem. Res.* **26**, 153-169 (2017).
2. Nachon, F., Carletti, E., Ronco, C., Trovaslet, M., Nicolet, Y., Jean, L., Renard, P.-Y. Crystal structures of human cholinesterases in complex with huprine W and tacrine: elements of specificity for anti-Alzheimer's drugs targeting acetyl- and butyryl-cholinesterase. *Biochem. J.* **453,** 393-399 (2013).
3. Dighe, S. N., Deora, G. S., De la Mora, E., Nachon, F., Chan, S., Parat, M.-O., Brazzolotto, X., Ross, B. P. Discovery and structure–activity relationships of a highly selective butyrylcholinesterase inhibitor by structure-based virtual screening. *J. Med. Chem.* **59,** 7683-7689 (2016).

In vitro cytotoxicity assay

Cytotoxic activity of the compounds was determined using MTT assay[1]. Primary cultured goat fetal fibroblasts and primary cultured porcine fetal kidney cells were cultured in the DMEM medium (high glucose) and a mixture culture medium consisting of 50% DMEM (high glucose) and 50% DMEM/F12, respectively, supplemented with 10% fetal bovine serum, penicillin (100 U/mL) and streptomycin (100 μg/mL) at 37 °C in 5% CO_2. The cells at exponential growth phase were seeded into a 96 well plate at 4.8×10^3 cells/200 μL/well and cultured for 24 h. After removal of the medium, the cells in each well were treated with 200 μL solution containing different concentrations of the tested compounds and vehicle controls which only received an equivalent amount of DMSO in quintuplicate for 48 h at 37 ℃. Thirty microliter of MTT solution (5 mg/mL in PBS) was added to each well with μL fresh medium for an additional 4 h. After removing the supernatant, 150 μL of DMSO was added to completely dissolve the formazan crystals that had formed in viable cells in the wells. Finally, the plates were shaken and the absorbance (A) was determined using microplate reader (Bio-Rad 680) at 570 nm. The wells containing the same media as other test wells but no cells were used as blank controls. All the doses were tested in quintuplicate and the experiments were repeated at least three times. The inhibition rates (IRs) were calculated. IC_{50} values and their confidence intervals at 95% probability (95% CI) were calculated by using PRISM software ver. 5.0 (GraphPad Software Inc., San Diego, CA, USA).

References

1. Cao, F.-J., Yang, R., Lv C., Ma, Q., Lei, M., Geng, H.-L., Zhou, L. Pseudocyanides of sanguinarine and chelerythrine and their series of structurally simple analogues as new anticancer lead compounds: cytotoxic activity, structure-activity relationship and apoptosis induction, *Eur. J. Pharm. Sci.* **67,** 45-54 (2015).
